# Directional sensitivity of bone conduction stimulation on the otic capsule in a finite element model of the human temporal bone

**DOI:** 10.1038/s41598-024-64377-x

**Published:** 2024-06-14

**Authors:** Paweł Borkowski

**Affiliations:** Institute of Aeronautics and Applied Mechanics, Faculty of Power and Aeronautical Engineering, Warsaw University of Technology, ul. Nowowiejska 24, 00-665 Warsaw, Poland

**Keywords:** Biomedical engineering, Mechanical engineering, Quality of life

## Abstract

Sound transmission to the human inner ear by bone conduction pathway with an implant attached to the otic capsule is a specific case where the cochlear response depends on the direction of the stimulating force. A finite element model of the temporal bone with the inner ear, no middle and outer ear structures, and an immobilized stapes footplate was used to assess the directional sensitivity of the cochlea. A concentrated mass represented the bone conduction implant. The harmonic analysis included seventeen frequencies within the hearing range and a full range of excitation directions. Two assessment criteria included: (1) bone vibrations of the round window edge in the direction perpendicular to its surface and (2) the fluid volume displacement of the round window membrane. The direction of maximum bone vibration at the round window edge was perpendicular to the round window. The maximum fluid volume displacement direction was nearly perpendicular to the modiolus axis, almost tangent to the stapes footplate, and inclined slightly to the round window. The direction perpendicular to the stapes footplate resulted in small cochlear responses for both criteria. A key factor responsible for directional sensitivity was the small distance of the excitation point from the cochlea.

## Introduction

The primary route of sound transmission to the human ear is air conduction. When it is impaired, and conventional hearing aids or other treatments are ineffective or not recommended, an option for handling conductive and mixed hearing loss is the bone conduction (BC) implant. Despite advanced devices available on the market, there is still a need to minimize medical complications and adverse effects of surgery and improve efficiency^1,2^. During BC stimulation, sounds reach the inner ear by five pathways: inertia of the cochlear fluids, compression of the cochlear walls, middle ear ossicles inertia, sound radiated in the ear canal, and pressure transmission from the cerebrospinal fluid^3,4^. Pressure transmission from the cerebrospinal fluid acts only as the stimulus of bone vibrations, so it does not affect the cochlear fluid directly^5^. Theoretical predictions using the lumped element impedance model of the inner ear indicated that in healthy ears, only the first three pathways played a role in the cochlear excitation^6^. The fluid inertia was dominant, but contributions of the cochlear wall compression and middle ear inertia were only up to 10 dB lower^7^. For an immobilized stapes footplate (SF), starting at 0.4 kHz, the cochlear wall compression was more important than the fluid inertia^6^.

The main goal of BC is to induce vibrations in a small part of the skull called the otic capsule (OC), which is located inside the petrous part of the temporal bone and forms the outer wall of the inner ear. A typical implantation site for BC devices is the mastoid process at the outer skull surface. The hearing mechanisms for bone conduction hearing aids (BCHAs) located at soft tissue (transcutaneous) and directly attached to the bone (percutaneous) are equal for different stimulation sites and driven primarily by bone vibrations^8^. The entire skull vibrates during BC stimulation applied at the mastoid. In experiments on cadaveric heads, the skull moved as a rigid body at low frequencies below 0.4 kHz^9^, and this movement disappeared at high frequencies due to complex modes of natural vibrations^10^. Recent research, supplemented by numerical simulation, showed that skull deformations start even at 0.25–0.5 kHz next to the stimulation site^11^.

The closer an implant is to the cochlea, the lower the energy demand to power the system, and the force giving sufficient excitation level also decreases. Movement of the skull parts distant from the inner ear leads to energy losses. Experiments on dry human temporal bones and skulls showed that acceleration components of the OC vibrations measured during BC stimulation depended on the distance from the stimulation site to the inner ear^12^ and the implant location^13^. Studies on cadaveric human heads^14^ and patients^15^ showed that the results for stimulation on the OC site were several times as good as for implantation on the temporal bone squamous.

Implanting the stimulator directly in the OC is still challenging in clinical practice due to the risk of damage to the labyrinth^16^, so its size must be small. Even for BCHAs intended for the mastoid process, the size of the transducer can make implantation difficult in some cases^17^. Besides, a transcutaneous device designed for application on the outer skull surface implanted close to the cochlea yielded only a moderate increase in the output signal^18^. Hence, the BC stimulation on the OC requires a new and more efficient solution. Decreasing the implant size due to the low energy demand reduces the area of direct stimulation, increasing the stimulation effectiveness by concentrating vibrations next to the cochlea. However, a critical factor may be the directional sensitivity of the OC. Due to the complex anatomical shape of the cochlea and heterogeneous mass and stiffness distributions of the surrounding petrous part of the temporal bone, it is unknown how the stimulation direction affects the cochlear response. So far, the directional sensitivity was not the subject of interest for researchers working on BC devices located on the skull surface, where the stimulation direction was toward the inner ear^19^. Comparison of the two directions of BC stimulation applied to the OC was the aim of a numerical simulation using the temporal bone model^20^. However, whether these two directions correspond to extreme cochlear excitation was unknown.

The audibility measurements related to BCHAs are difficult. New methods include, for example, a sound-insulated skin microphone placed on the forehead^21^. The most reliable criterion for assessing the intensity of cochlear response in conscious patients is the hearing threshold (HT). It correlates well with excitation of the basilar membrane (BM), a part of the Corti organ that sends signals to the brain. The BM vibration depends on the pressure difference between the scala tympani and the scala vestibule, which is measurable on cadavers^22^. There are measurement inaccuracies for BC stimulation due to relative movements between pressure gauges and vibrating bone^23^. Kim et al.^24^ numerically modeled the influence of excitation direction on the BM velocity. The most effective excitation direction was almost perpendicular to the SF and the BM in its initial part (hook region), and the intensity of BM vibration correlated with an antisymmetric component of the intracochlear pressure.

As the BM measurements along the cochlea during BC stimulation are not easy^25^, the cochlear promontory (CP) velocity was the criterion used in experiments and modeling studies. The CP vibrations measured by laser Doppler vibrometer (LDV) on cadaveric heads and HTs in human subjects with a percutaneous BCHA attached to the skull by a headband revealed that the ratio between the HT level and CP vibration intensity depended on the stimulation position^26^. Measurements of BC stimulation also revealed that intracochlear pressures were proportional to the CP movement^27^. The ratios between intracochlear pressures and CP motion above 1 kHz did not depend on the stimulation level, type of BCHA coupling, or stimulation site^28^. Measurements using a 3D LDV showed that CP vibrations in three directions provided better information about HTs than measurements along a single axis^29^. The 3D LDV measurements in guinea pigs included nine criteria for predicting HTs based on the CP velocities^30^. The best criterion was a weighted sum based on three orthogonal directions, and the vibration component along a single direction was the most inaccurate. The dominant vibration direction varied with frequency, and the vibrations along all three axes at high frequencies were almost independent of the BC stimulation direction. Lim et al.^31^ used a numerical model of the human head to compare two implant locations on the skull surface. They pointed out the problem with measuring CP vibrations at low frequencies resulting from the fact that the head rotates when the stimulating force does not pass through its mass center. In turn, at high frequencies, the measurement of a single point at the CP is not representative due to the complex vibration modes of the OC. Moreover, the CP and BM velocities exhibited different trends versus frequency at the medium frequency range of 0.5–3 kHz^32^. Therefore, more than a single criterion based on the CP vibration is required to assess the directional sensitivity of the cochlea, regardless of whether velocity measurements are one or three-directional.

Another measure of the cochlear excitation is the fluid volume in the scala tympani, moving along with the round window (RW) membrane and calculated relative to the bone vibration; in other words, the fluid volume displacement (VD). It reflects the effects of cochlear wall compression, fluid inertia, middle ear ossicles inertia, and the third window (cochlear and vestibular aqueducts, blood vessels, and microchannels). According to^33^, anatomical differences in vestibular aqueduct size affect BC hearing only at low frequencies. Dobrev et al.^5^ also suggested a reduced importance of the third window.

This paper’s hypothesis states that the directional sensitivity of the human cochlea exists and has extremes. The present report aims to search for characteristic directions of BC stimulation. It is impossible to conduct in patients and time-consuming in cadaveric studies. The direction of the most intense excitation is crucial for miniaturizing the BC device intended for implantation on the OC and setting its vibration direction. Conversely, determining the direction that gives the weakest cochlear response is also essential regarding the implant interaction with vocal cord vibrations transmitted through the bone to the inner ear. Numerical simulation seems appropriate for identifying advantageous directions of BC stimulation, studying how they change versus frequency, and applying this knowledge in future LDV measurements. Therefore, the directional sensitivity criteria used in this study included RW vibrations, although the BM gives more reliable information about the cochlear response. The CP can move relative to the RW depending on the direction of BC stimulation, which influences the fluid VD calculation. Hence, in the present study, the criterion of average bone displacement at the RW edge replaced the CP movement. The RW edge and CP vibrations are comparable due to the high OC stiffness. Although considering only one vibration direction perpendicular to the RW is a significant simplification^29^, this criterion corresponds well to the fluid VD.

The model used in the presented study assumed an otosclerosis condition by immobilizing the SF in the oval window. During BC stimulation measurements on cadavers, the fluid VD of the RW membrane did not correlate with the fluid volume of the SF as well as it does for air conduction, and the VD reduction after immobilization of the SF was only moderate^34^. Assuming that the effect of the middle ear ossicles inertia is secondary to the stimulation on the OC and depends on the stimulation direction, it can only complicate the interpretation of the results, which was a reason for its elimination.

This finite element study of the human temporal bone model aims to check how the direction of BC stimulation applied to the OC affects the bone and fluid vibrations at the RW and to evaluate the directional sensitivity of the cochlea by intersecting both criteria.

## Methods

### Finite element model. Boundary conditions in harmonic analysis

The previously developed finite element model of the temporal bone with the inner ear was used^20^. The processing of CT scans resulted in files with geometry in the form of a large set of small triangular surfaces separated from each other. The surfaces defined the bone contour, but the information regarding the heterogeneity of the structure was lost. After loading the files into ANSYS (Release 17.2, ANSYS Inc., Canonsburg, Pennsylvania, United States), the main steps of model creation included defining cross-sections to generate points, connecting them with splines, creating surfaces and volumes, and finally discretizing them using 3-D finite elements. Defining the interface between cortical and trabecular tissues in the temporal bone model was difficult. The method used to determine the region of cortical bone was the selection of subsequent layers of finite elements, starting from the bone contour. The number of layers depended on the site (see Supplementary Graphics S1 online). Based on literature data, tuning of the stiffness, density, and damping properties was done for the RW membrane, BM, stapes, spiral lamina, spiral ligament, annular ligament, and uniformly distributed mass, imitating soft tissues vibrating with the bone^20^. Material properties were isotropic and linearly elastic or viscoelastic, and the BM model was passive.

The temporal bone support included surfaces connecting with the other skull bones, excluding the petro-occipital synchondrosis. The annular ligament ossification imitated the SF otosclerosis, and other middle and outer ear structures were absent. The stimulation site was on the OC surface above the lateral semicircular canal. A concentrated mass of 0.16 g connected to the nodes lying on the OC surface in a circle with a radius of 1.5 mm represented the BC implant so that its natural frequencies did not affect the simulation results. A harmonic force applied to the mass and directed toward the OC had an amplitude of 0.1 N, as in^20^. A set of 17 frequencies based on the one-third octave band: 0.2, 0.3, 0.4, 0.5, 0.63, 0.8, 1.0, 1.25, 1.6, 2.0, 2.5, 3.15, 4.0, 5.0, 6.3, 8, and 10 kHz represented the hearing range. For the presented model, the force amplitude of 0.1 N in the primary direction resulted in the relative displacement of the BM at 1 kHz twice as great as the one obtained for air conduction at the sound pressure level of 80 dB^20^. The temporal bone weighed 28 g and had an associated mass of 25 g, imitating soft tissues (skin and brain), modeled as a surface density of 2.5·10^–3^ g/mm^2^, and uniformly distributed on the outer and inner bone surfaces. While validating the presented model, adjusting the surface density gave the best fit to the experimental data.

### The round window vibrations

**Figure 1:**
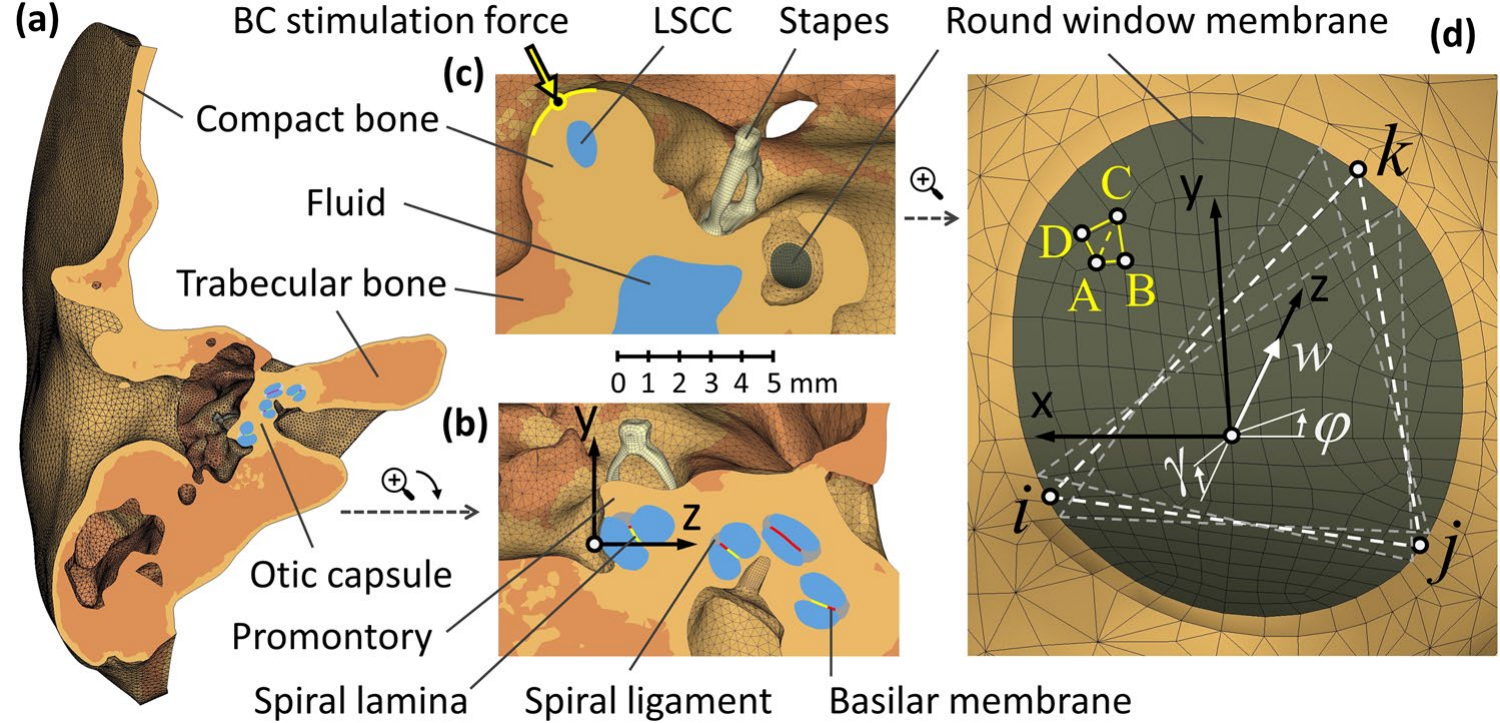
Finite element model: (**a**, **b**) views of the temporal bone section in the *yz-plane*, (**c**) the *xy-plane* section with the bone conduction (BC) force applied to the otic capsule above the lateral semicircular canal (LSCC), (**d**) the *xy-plane* view of the round window (RW). Sixteen triangles *ijk* described the bone vibration at the RW edge (dashed lines show two consecutive triangles). The exemplary triangles ABC and ACD represented the areas used to calculate the fluid volume displacement of the RW membrane.

The movement of the RW edge consisted of a translation *w* along the *z-axis* and two rotations *γ*, *φ* around the *x-* and *y-axes* (Fig. 1). The right-handed coordinate system *xyz* had the origin at the RW center, and its *z*-*axis* was perpendicular to the membrane and directed toward the cochlea. There were 48 nodes lying on the border between the compact bone and the RW membrane. Triplets of nodes on the RW edge defined the vertices *i*, *j*, *k* of *M* = 16 triangles. With the assumption of small rotations, the real and imaginary parts of the vertices displacement along the *z-axis* can be expressed as Eq. (1):

1 $$\left\{ {\begin{array}{*{20}c} {w_{i}^{p} } \\ {w_{j}^{p} } \\ {w_{k}^{p} } \\ \end{array} } \right\} = \left[ {\begin{array}{*{20}c} 1 & {y_{i} } & { - x_{i } } \\ 1 & {y_{j} } & { - x_{j } } \\ 1 & {y_{k} } & { - x_{k } } \\ \end{array} } \right]\left\{ {\begin{array}{*{20}c} {w^{p} } \\ {\gamma^{p} } \\ {\varphi^{p} } \\ \end{array} } \right\};\;\;p = Re, Im.$$

The matrix on the right-hand side in Eq. (1) contained the vertices coordinates, and the displacements on the left were nodal results, so after inverting the matrix, the movement of a single triangle was calculated as Eq. (2):

2 $$\left\{ {\begin{array}{*{20}c} {w^{p} } \\ {\gamma^{p} } \\ {\varphi^{p} } \\ \end{array} } \right\} = \frac{1}{{\left( {y_{k} - y_{i} } \right)x_{j} + \left( {y_{j} - y_{k} } \right)x_{i} + \left( {y_{i} - y_{j} } \right)x_{k} }}\left[ {\begin{array}{*{20}c} {x_{j} y_{k} - y_{j} x_{k} } & {y_{i} x_{k} - x_{i} y_{k} } & {x_{i} y_{j} - y_{i} x_{j} } \\ {x_{k} - x_{j} } & {x_{i} - x_{k} } & {x_{j} - x_{i} } \\ {y_{k} - y_{j} } & {y_{i} - y_{k} } & {y_{j} - y_{i} } \\ \end{array} } \right]\left\{ {\begin{array}{*{20}c} {w_{i}^{p} } \\ {w_{j}^{p} } \\ {w_{k}^{p} } \\ \end{array} } \right\}.$$

The $\overline{w}^{p}$, $\overline{\gamma }^{p}$ i $\overline{\varphi }^{p}$ components averaged over 16 triangles represented the RW edge movement, subtracted from the RW membrane displacement to obtain the fluid VD. Because the RW membrane consisted of hexahedral finite elements, 218 quadrilaterals on its surface were post-processed as *N* = 436 triangles with vertices *A*, *B*, *C* (Fig. 1). The relative displacement of each vertex was expressed as Eq. (3):

3 $${\Delta }w_{s}^{p} = w_{s}^{p} - \left( {\overline{w}_{p} + \overline{\gamma }^{p} \cdot y_{s } - \overline{\varphi }^{p} \cdot x_{s } } \right);\;\;\; s = A, B, C .$$

The area of the triangle projection on the *xy*-plane was calculated as Eq. (4):

4 $$A_{t} = \frac{1}{2}\left( {x_{A} y_{B} + x_{B} y_{C} + x_{C} y_{A} - x_{A} y_{C} - x_{B} y_{A} - x_{C} y_{B} } \right)$$

The fluid VD for a single triangle was its area from Eq. (4) times the average of three values from Eq. (3), as shown in Eq. (5):

5 $$V_{t}^{p} = \frac{{A_{t} }}{3}\left( {{\Delta }w_{A}^{p} + {\Delta }w_{B}^{p} + {\Delta }w_{C}^{p} } \right).$$

The real and imaginary parts of the fluid VD of the RW membrane were summations of the values from Eq. (5) over *N* triangles, as in Eq. (6):

6 $$V^{p} = \mathop \sum \limits_{t = 1}^{N} V_{t}^{p} .$$

The amplitude and phase characteristics of the RW edge displacement and the fluid VD of the RW membrane versus frequency were based on the real and imaginary parts of $\overline{w}^{p}$ and $V^{p}$.

### Extreme directions of BC stimulation

**Figure 2:**
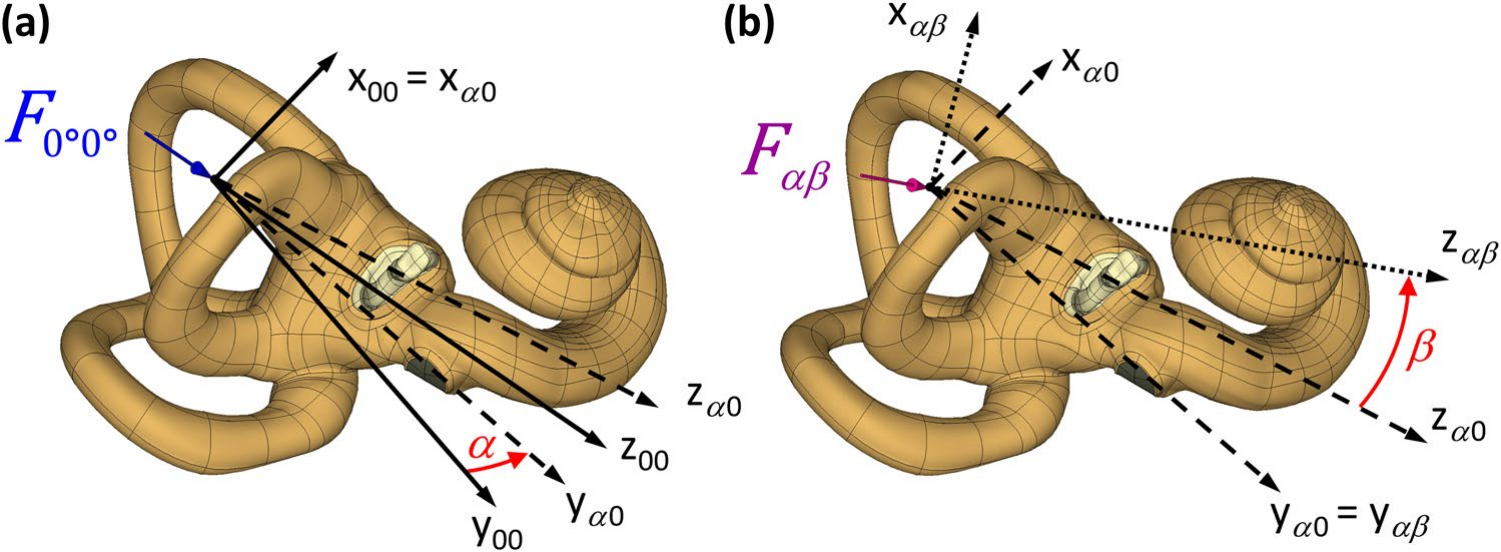
Parametrization of the force direction with *α* and *β* angles: (**a**) the *F*_0°0°_ force indicated the primary direction of bone conduction stimulation (*α* = 0°, *β* = 0°), (**b**) the *F*_*αβ*_ force direction coincided with the *z*_*αβ*_ -*axis*. The stimulation site was at the origin of the coordinate system.

The global coordinate system *ξηζ* of the skull (Fig. 6) had the origin at point (0, 0, 0) and versors: $\hat{\xi }^{T}$ = [1,0,0], $\hat{\eta }^{T}$ = [0,1,0], $\hat{\zeta }^{T}$ = [0,0,1]. The origin of the right-handed coordinate system *x*_00_ *y*_00_ *z*_00_ coincided with the BC stimulation site (Fig. 2) and had coordinates (− 45.92, 6.24, − 25.28 mm) and versors: $\hat{x}_{00}^{T}$ = [0.004, − 0.918, 0.397], $\hat{y}_{00}^{T}$ = [− 0.270, − 0.383, − 0.884], $\hat{z}_{00}^{T}$ = [0.963, − 0.104, − 0.249] defined in the global system. The *α* and *β* angles determined the *F*_*αβ*_ force direction. The primary direction (*α* = 0°, *β* = 0°) lay on the *z*_00_-*axis*. It coincided with the axis of an imaginary cone inscribed in the anterior part of the hole made in the temporal bone during mastoidectomy, as in^20^. The *α* angle described the force rotation around the *x*_00_-*axis*, after which the *y*_00_-*axis* became *y*_*α *0_. Hence, the versors $\hat{x}$_00_ and $\hat{x}$_*α *0_ coincided, and the versors $\hat{y}$_*α *0_ and $\hat{z}$_*α *0_ were products of the rotation matrix and versors of the global system calculated as Eq. (7):

7 $$\hat{y}_{ \alpha 0} = {\mathbf{A}} \hat{y}_{0 0} ;\;\;\; \hat{z}_{ \alpha 0} = {\mathbf{A}} \hat{z}_{00} ,$$

where: $${\mathbf{A}} = \hat{x}_{00} \hat{x}_{00}^{T} \left( {1 - {\text{cos}}\alpha } \right) + {\mathbf{I}} {\text{cos}}\alpha + \left[ {\begin{array}{*{20}c} 0 & { - \hat{x}_{00} \cdot \hat{\zeta }} & {\hat{x}_{00} \cdot \hat{\eta }} \\ {\hat{x}_{00} \cdot \hat{\zeta }} & 0 & { - \hat{x}_{00} \cdot \hat{\xi }} \\ { - \hat{x}_{00} \cdot \hat{\eta }} & {\hat{x}_{00} \cdot \hat{\xi }} & 0 \\ \end{array} } \right]{\text{sin}}\alpha$$, and ${\mathbf{I}}$ was the identity matrix.

The *β* angle described the rotation of the force around the *y*_*α *0_-axis, where the *z*_*α *0_-*axis* became *z*_*α β*_. Hence, the versors $\hat{y}_{ \alpha 0}$ and $\hat{y}_{ \alpha \beta }$ coincided, and the versors $\hat{x}$_*α β*_i $\hat{z}$_*α β*_ were calculated as Eq. (8):

8 $$\hat{x}_{ \alpha \beta } = {\mathbf{B}} \hat{x}_{ \alpha 0} ;\;\;\; \hat{z}_{\alpha \beta } = {\mathbf{B}} \hat{z}_{\alpha 0} ,$$

where: $${\mathbf{B}} = \hat{y}_{ \alpha 0} \hat{y}_{ \alpha 0}^{T} \left( {1 - {\text{cos}}} \right) + {\mathbf{I}} {\text{cos}} + \left[ {\begin{array}{*{20}c} 0 & { - \hat{y}_{ \alpha 0} \cdot \hat{\zeta }} & {\hat{y}_{\alpha 0} \cdot \hat{\eta }} \\ {\hat{y}_{\alpha 0} \cdot \hat{\zeta }} & 0 & { - \hat{y}_{ \alpha 0} \cdot \hat{\xi }} \\ { - \hat{y}_{ \alpha 0} \cdot \hat{\eta }} & {\hat{y}_{ \alpha 0} \cdot \hat{\xi }} & 0 \\ \end{array} } \right]{\text{sin}}\beta {.}$$

The *α* and *β* increments in the simulation were 15° from − 90° to 90° to cover a full range of BC stimulation directions. The vibrations for the pairs (*α* + *π, β* + *π)* had the same amplitudes and reversed phases as those obtained for (*α, − β)*.

The two amplitudes determined the intensity of BC stimulation at a given direction and frequency: $V_{\alpha \beta } \left( f \right)$ denoted the fluid VD of the RW membrane, and $\overline{w}_{\alpha \beta } \left( f \right)$ was the average RW edge displacement along the *z*-direction (Fig. 1). The extreme $V_{max}^{f} , V_{min}^{f} ,{ }\overline{w}_{max}^{f} , \overline{w}_{min}^{f}$ values at a given frequency were used to get normalized values expressed as Eq. (9):

9 $$V_{\alpha \beta }^{n} \left( f \right) = \frac{{V_{\alpha \beta } \left( f \right)}}{{V_{max}^{f} }} ,\;\;\overline{w}_{\alpha \beta }^{n} \left( f \right) = \frac{{\overline{w}_{\alpha \beta } \left( f \right)}}{{\overline{w}_{max}^{f} }} ,$$

and to evaluate the weight coefficients shown in Eq. (10):

10 $$\mu_{V}^{f} = 1 - \frac{{V_{\min }^{f} }}{{V_{\max }^{f} }},\;\;\;\mu _{w}^{f} = 1 - \frac{{\overline{w}_{\min }^{f} { }}}{{\overline{w}_{\max }^{f} { }}} .$$

If the weight coefficients were close to one, the effectiveness of BC stimulation at a given frequency depended on the force direction. If they were close to zero, the stimulation direction did not matter. The effectiveness of BC stimulation in the entire set of 17 frequencies included two criteria of a weighted sum based on values from Eq. (9) and (10) calculated as Eq. (11):

11 $$V_{{{ }\alpha \beta }}^{crit} = \frac{{\mathop \sum \nolimits_{f = 1}^{17} V_{\alpha \beta }^{n} \left( f \right) \cdot \mu_{V}^{f} }}{{\mathop \sum \nolimits_{f = 1}^{17} \mu_{V}^{f} }},\;\;\overline{w}_{{\alpha \beta { }}}^{crit} = \frac{{\mathop \sum \nolimits_{i = 1}^{17} \overline{w}_{\alpha \beta }^{n} \left( f \right) \cdot \mu_{w}^{f} }}{{\mathop \sum \nolimits_{i = 1}^{17} \mu_{w}^{f} }}$$

Finally, the aim was to find pairs of angles (*α, β*) giving extreme values ($V_{max}^{crit}$, $V_{min}^{crit}$, $\overline{w}_{{max{ }}}^{crit}$, $\overline{w}_{{min{ }}}^{crit}$) in the entire frequency range.

## Results

### Criteria of weighted sums

**Figure 3:**
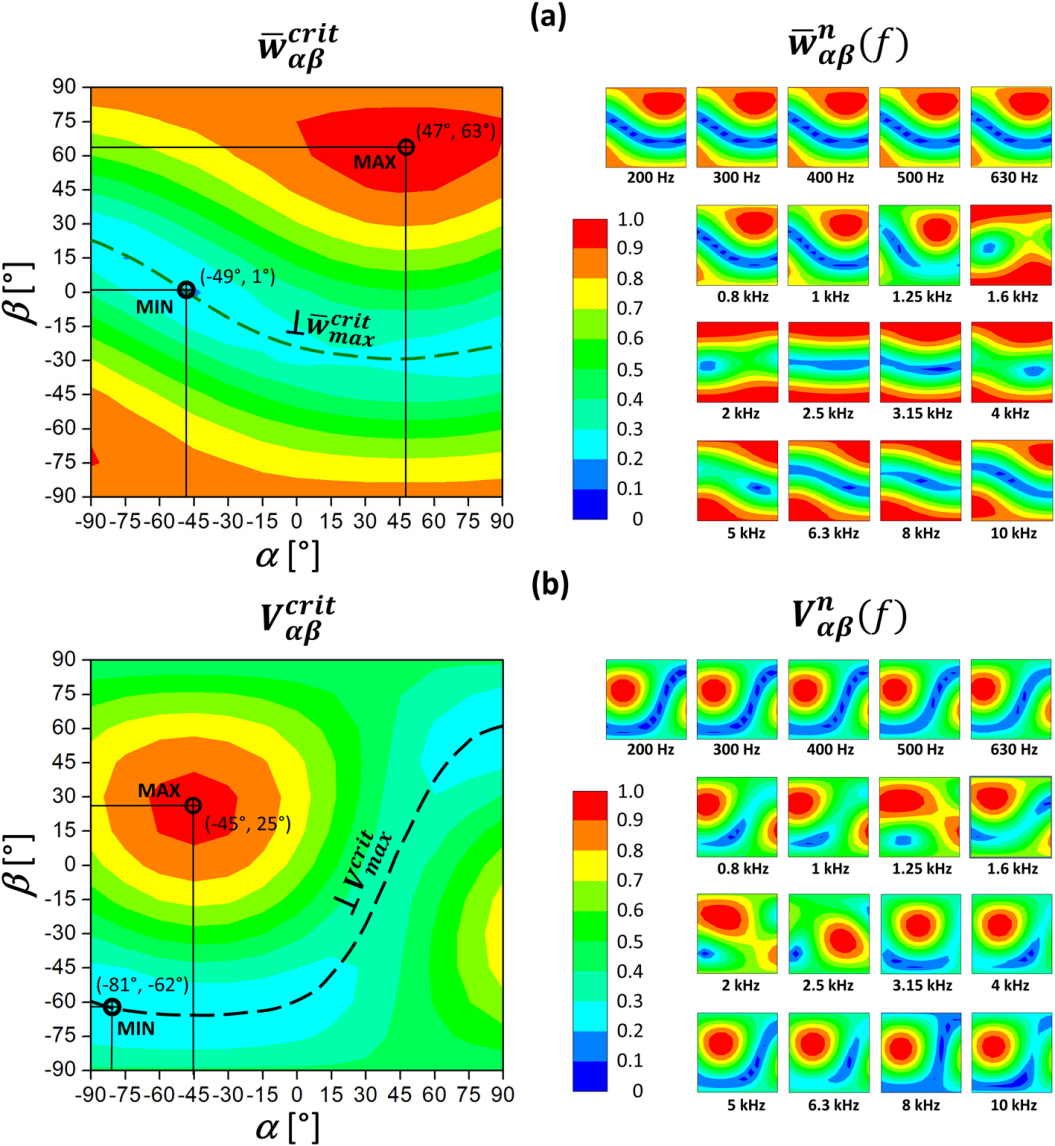
Distributions of two weighted sum criteria for the entire frequency range (left) and normalized amplitudes of the round window (RW) vibrations for 17 frequencies (right). The results shown versus the direction of bone conduction stimulation included (**a**) the average displacement of the RW edge and (**b**) the fluid volume displacement of the RW membrane. Dashed lines denoted directions in a plane perpendicular to the force direction corresponding to the maximum for a given criterion $\overline{w}_{max}^{crit}$ or $V_{max}^{crit}$. The small squares on the right had the same *α* and *β* axes as the two surface plots on the left. The amplitudes in small plots were normalized relative to $V_{max}^{f}$ or $\overline{w}_{max}^{f}$ values, shown in Fig. 5 as lower limits of the upper shaded areas.

The distributions of both weighted sum criteria, according to Eq. (11), resembled fragments of maps containing a hill and a valley with a stream (Fig. 3, left). A single maximum existed for a certain pair of angles *α* and *β*. The BC stimulation in the directions perpendicular to the direction of the maximum yielded small criterion values. These directions for both criteria lay on dashed lines in Fig. 3 (left), containing pairs of angles corresponding to minimum values. Since the angles in the simulation changed every 15°, the accuracy of the extreme directions evaluation was not satisfactory. Thus, the directions corresponding to extremes for each criterion resulted from interpolating the results obtained for pairs (*α* −15°, *β* −15°), (*α*, *β* −15°), (*α* + 15°, *β* −15°), (*α* −15°, *β*), (*α*, *β* ), (*α* + 15°, *β* ), (*α* -15°, *β* + 15°), (*α*, *β* + 15°), (*α* + 15°, *β* + 15°) near the ‘coarse’ extremum for (*α*, *β*), based on the polynomial shape functions of a 9-node Lagrangian surface finite element.

The maximum average displacement of the RW edge according to the criterion of $\overline{w}_{{{ }\alpha \beta }}^{crit}$ occurred for *α* = 47° and *β* = 63° (Fig. 3). The directions in a plane perpendicular to the direction of $\overline{w}_{{max{ }}}^{crit}$(shown as the dashed line in Fig. 3a) yielded the criterion values of less than 30% of $\overline{w}_{{max{ }}}^{crit}$ and 19% for the minimum at angles (− 49°, 1°). Distributions of the normalized displacement $\overline{w}_{\alpha \beta }^{n} \left( f \right)$ were similar up to 1.25 kHz and above 4 kHz to those obtained for the criterion $\overline{w}_{{\alpha \beta { }}}^{crit}$, while for 1.6 and 3.15 kHz they almost did not depend on the *α* angle and reached a maximum for large values of *β*. Since the weighting coefficient $\mu_{w}^{f}$ was between 0.862 (at 2 kHz) and 0.997 (5 kHz), the displacement of the RW edge depended on the stimulation direction.

The maximum value of the fluid VD of the RW membrane according to the criterion of $V_{{{ }\alpha \beta }}^{crit}$ occurred for *α* = − 45° and *β* = 25° (Fig. 3b). Directions in a plane perpendicular to the direction of $V_{max}^{crit}$ resulted in the criterion values of less than 36% of $V_{max}^{crit}$ and dropped to 21% for angles (− 81°, 62°) corresponding to the direction of $V_{min}^{crit}$. The distributions of $V_{\alpha \beta }^{n} \left( f \right)$ and $V_{{{ }\alpha \beta }}^{crit}$ were similar except two frequencies. At 2.5 kHz, the maximum occurred for *α* = 30° and *β* = − 15°, where $V_{ - 45^\circ 25^\circ }^{n}$ was 56% of $V_{30^\circ - 15^\circ }^{n}$. At 3.15 kHz, the maximum occurred for (− 6°, 19°), and $V_{ - 45^\circ 25^\circ }^{n}$ was 83% of $V_{ - 6^\circ 19^\circ }^{n}$. The weighting coefficient $\mu_{w}^{f}$ ranged between 0.862 at 1.25 kHz and 0.989 at 0.2 kHz, so the the stimulation direction mattered in terms of the VD.

### Characteristic BC stimulation directions

**Figure 4:**
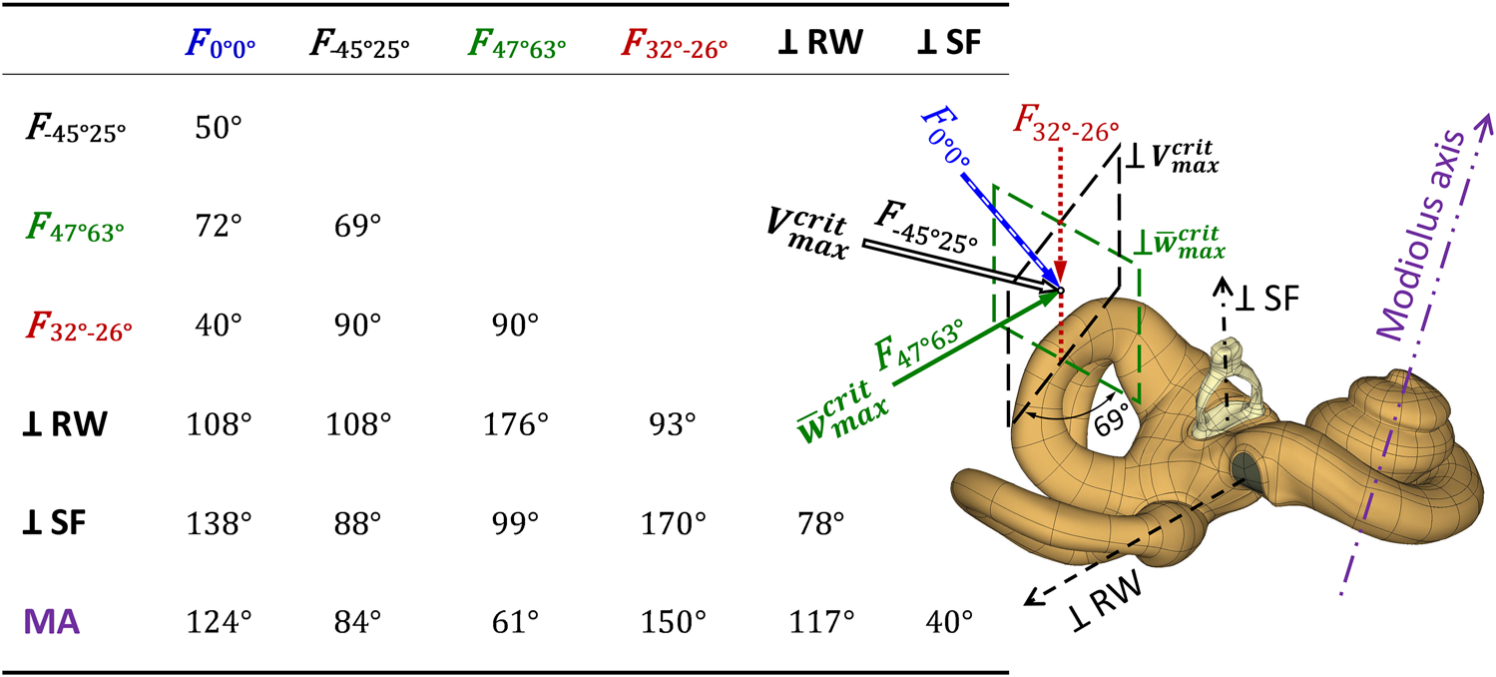
Angles between bone conduction stimulation forces and characteristic directions in the cochlea. 0°0°—the primary direction, $V_{max}^{crit}$ —direction of the $V_{{\alpha \beta { }}}^{crit}$ maximum (− 45°, 25°), $\overline{w}_{{max{ }}}^{crit}$—direction of the $\overline{w}_{{\alpha \beta { }}}^{crit}$ maximum (47°, 63°), 32°–26°—direction of weak vibrations according to both criteria, ⊥RW—direction orthogonal to the round window membrane, ⊥SF—direction orthogonal to the stapes footplate, MA—direction of the modiolus axis.

The two planes perpendicular to the directions corresponded to maximum values of $V_{max}^{crit}$ and $\overline{w}_{{max{ }}}^{crit}$ criteria intersected along the direction defined by *α* = 32° and *β* = − 26° (the dotted line in Fig. 4). The direction of the *F*_*32°−26°*_ force resulted in both small RW edge displacement and the fluid VD and corresponded to the crossing of two dashed lines from Fig. 3. The direction of $V_{max}^{crit}$ (− 45°, 25°) was inclined to the RW surface at 18°, and was almost perpendicular to the modiolus axis and tangent to the SF. The direction of $\overline{w}_{{max{ }}}^{crit}$ (47°, 63°) was nearly perpendicular to the RW. The direction of weak vibrations according to both criteria (32°, − 26°) was roughly perpendicular to the SF.

**Figure 5:**
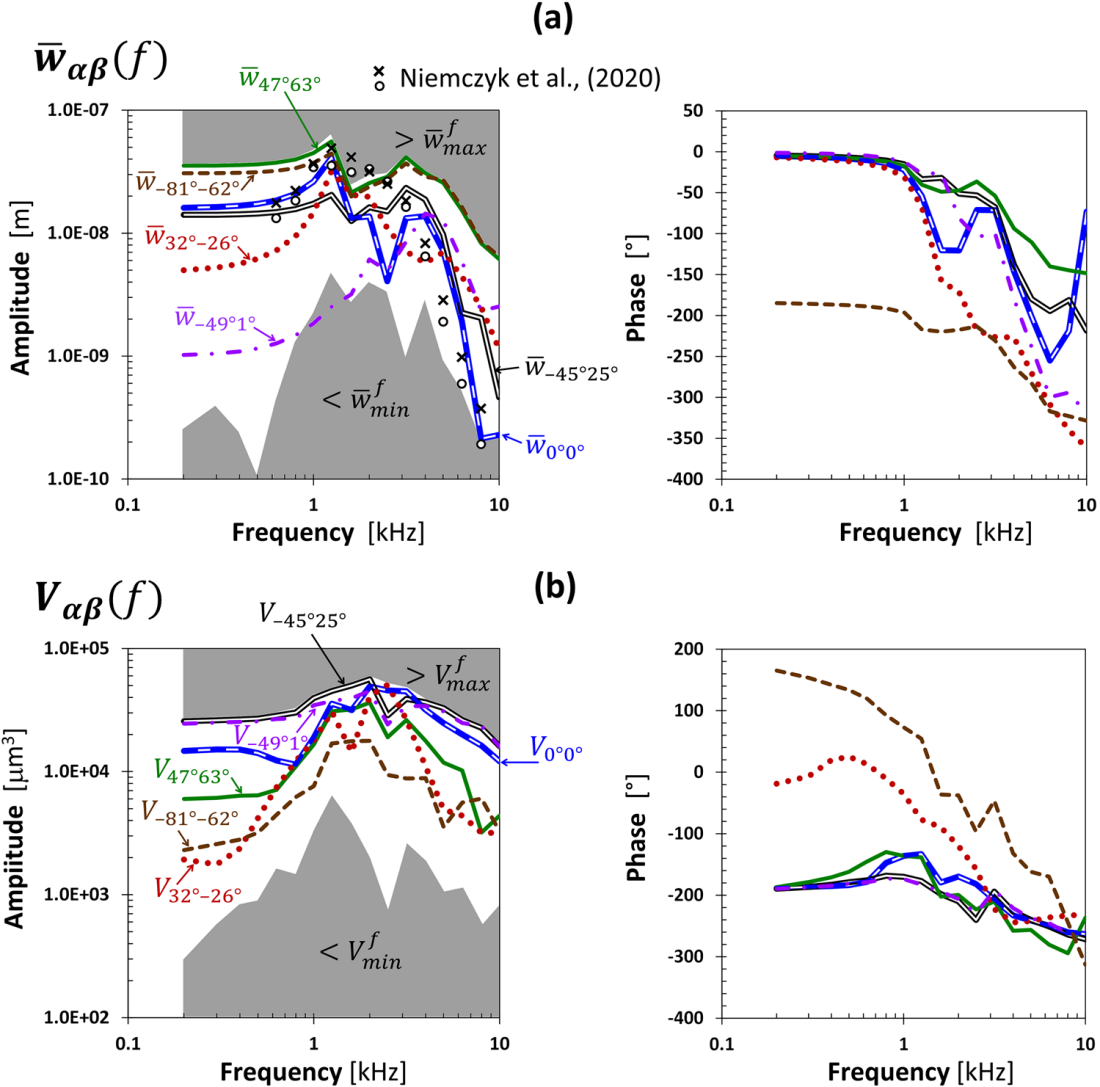
The round window (RW) vibrations for six directions of bone conduction stimulation with a force of 0.1 N versus frequency: (**a**) averaged displacement of the RW edge in the direction perpendicular to its surface, compared with the cochlear promontory vibration measured on cadavers for two stimulation sites at the otic capsule^14^, (**b**) the fluid volume displacement of the RW membrane. Values in shaded areas were outside the extreme ranges.

For the direction of $\overline{w}_{{max{ }}}^{crit}$(47°, 63°), the average displacements of the RW edge corresponded to $\overline{w}_{max}^{f}$ values determined for all directions in the entire frequency range (Fig. 5a). The amplitudes of the RW edge displacements for the directions (0°, 0°) and (− 45°, 25°) at frequencies below 0.63 kHz and for 1.6, 2, 4, 5, and 6.3 kHz were similar, although several times as low as those obtained for the $\overline{w}_{{max{ }}}^{crit}$ direction. The vibration amplitudes of the RW edge for the primary direction (0°, 0°) were comparable to the CP vibrations measured on cadavers^6^. The RW edge displacements for the direction (32°, − 26°) were at low frequencies an order of magnitude lower than those obtained for the direction (47°, 63°); comparable for 1, 1.25, and 1.6 kHz, and few times as low at higher frequencies. The direction corresponding to the minimum $\overline{w}_{{min{ }}}^{crit}$ (− 49°, 1°) belonged to the plane perpendicular to the $\overline{w}_{max}^{crit}$ direction (Fig. 4), so the OC vibrated mainly in the direction tangent to the RW, and the displacements below 2 kHz were several times as small as those obtained for the direction (32°, − 26°). The angle between the directions (− 81°, − 62°) and (47°, 63°) was 158°; hence, the vibrations of the RW edge for both directions had opposite phases, and the amplitudes were comparable. The influence of the resonant frequencies was evident at 1.25 and 3.15 kHz.

**Figure 6:**
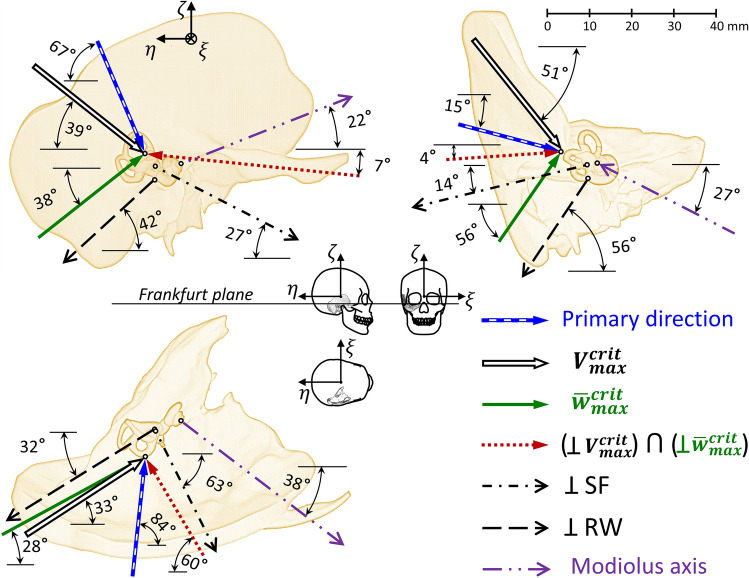
Characteristic directions of bone conduction stimulation assuming the stapes otosclerosis: primary (0°, 0°), $V_{max}^{crit}$ —maximum of the fluid volume displacement (VD) of the round window (RW) membrane (− 45°, 25°), $\overline{w}_{{max{ }}}^{crit}$—maximum RW edge displacement (47°, 63°), $\left( { \bot V_{max}^{crit} } \right) \cap \left( {\bot \overline{w}_{{max{ }}}^{crit} } \right)$—weak vibrations in terms of RW edge and VD (32°, − 26°). Characteristic directions of the cochlea included the modiolus axis and directions orthogonal to the stapes footplate (SF) and the RW membrane.

For the direction of $V_{max}^{crit}$ (− 45°, 25°), the fluid VD of the RW membrane was close to the maximum $V_{max}^{f}$ values obtained from all directions in the entire frequency range, except 2.5 and 3.15 kHz (Fig. 5b). The angle between the directions (− 49°, 1°) and (− 45°, 25°) was 24°, which resulted in similar values of VD amplitudes and phases for both directions. The fluid VD for the primary direction (0°, 0°) was 1.3–2 times as low as for (− 45°, 25°), except at 2.5 and 3.15 kHz, where the results were greater. From 0.8 to 2 kHz, the results for the directions of maximum RW edge vibrations (47°, 63°) and primary (0°, 0°) were similar. Outside this range, the fluid VD for the direction of $\overline{w}_{{max{ }}}^{crit}$(47°, 63°) was a few times as low. For the direction of weak vibrations in terms of two criteria (32°, − 26°), the VD amplitudes were the lowest; however, an order of magnitude higher than the minimum $V_{min}^{f}$ values, and the VD and RW edge phases were similar at low frequencies. The phase for (32°, − 26°) was at low frequencies out of phase relative to the others. Results for this direction were like those obtained for the direction corresponding to the minimum $V_{min}^{crit}$ (− 81°, − 62°) except for the range from 2 to 4 kHz. At 2 and 2.5 kHz, results for (32°, − 26°) were nearly the same as for the primary direction (0°, 0°). Above 3.15 kHz, VD in the directions (− 45°, 25°), (− 49°, 1°), and (0°, 0°) had a few times as high amplitudes compared to the other three directions. Figure 6 shows the four directions of BC stimulation and characteristic directions of the cochlea based on versors (Eqs. 7, 8) projected on the sagittal, coronal, and transverse skull planes. The *ξζ* plane of the skull coordinate system passed through the styloid process, and the *ξη* plane was parallel to the Frankfurt plane. The two projections sufficiently describe the force direction as the intersection of two planes perpendicular to the projection and containing the vector.

## Discussion

**Figure 7:**
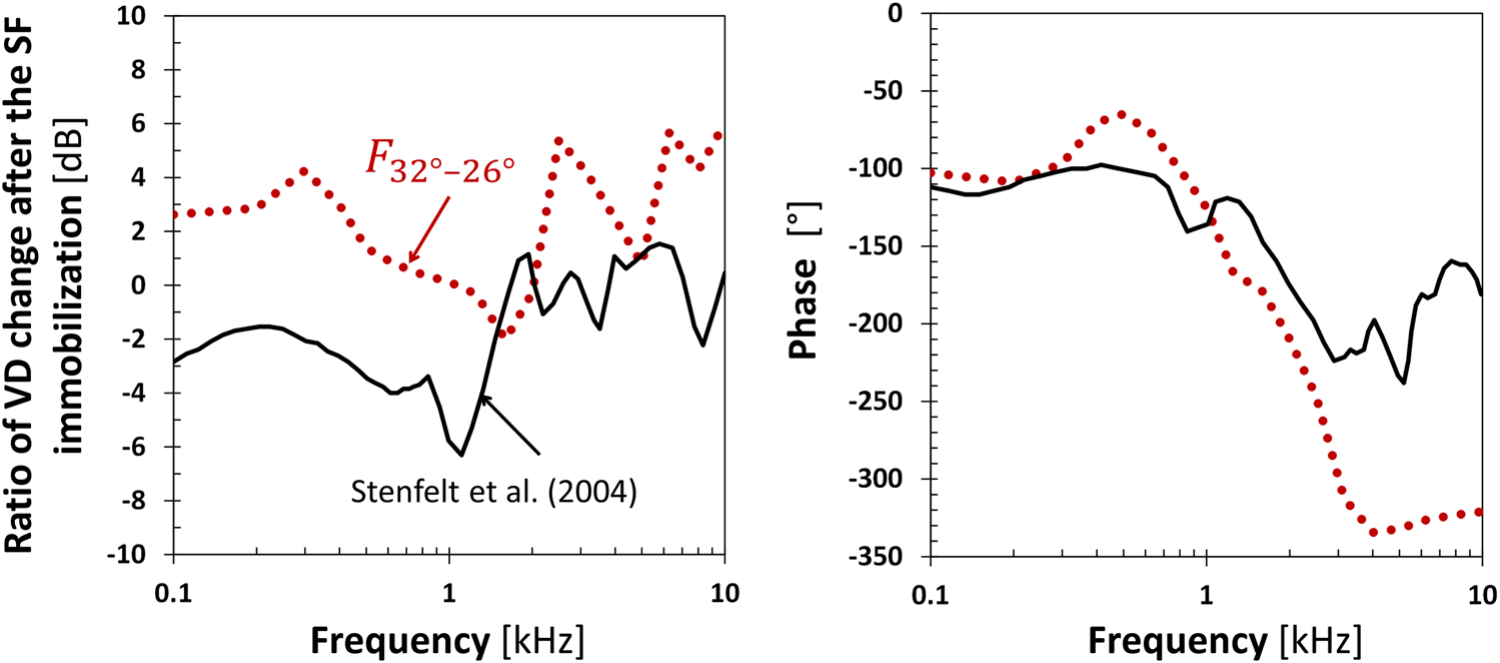
The change of the fluid volume displacement (VD) of the round window membrane after its immobilization in the oval window. The change was relative to a movable stapes without the incus and malleus. The excitation direction was roughly perpendicular to the stapes footplate (32°, − 26°). Results from the presented finite element model compared with the experimental data^34^.

Numerical simulations revealed that the cochlea is sensitive to the direction of stimulating force in the case of BC stimulation on the OC. A key factor was the small distance of the excitation point from the cochlea because of nonuniform distributions of mass and stiffness in the petrous temporal bone. However, observations of a real cochlea may provide quantitatively different results due to simplifying assumptions in the presented numerical simulation. The first was a step change in stiffness at the border between the compact and trabecular bones in the presented model. Since the compact tissue surrounding the fluid space in the cochlea has almost uniform material properties, the effect of compression of the cochlear walls depends little on whether the change in bone tissue density with distance from the OC is abrupt or gradual. This assumption could influence the OC vibration modes and modify the *α* and *β* angles describing specific BC stimulation directions. The second simplifying assumption was the stapes otosclerosis, where the cochlear wall compression dominates^6^. In the case of BC stimulation with force acting close to the cochlea, isolating the effect of cochlear walls compression by eliminating the secondary effect, i.e., the middle and outer ear dynamics, seemed justified, also considering that the importance of BC increases in the case of conduction system pathologies. This assumption did not affect the vibration of the RW edge but influenced the fluid VD of the RW membrane. The experiment on dry temporal bones^34^ showed that the VD at low frequencies for an immobilized stapes (glued footplate) was slightly lower than for a movable stapes (with removed other ossicles). After immobilization of the SF, the VD reduction was only a few dB below 1.5 kHz and less than 2 dB for higher frequencies. Similar differences also occurred in the presented finite element model (Fig. 7). However, the change was positive for otosclerosis, which indicated that in the case of BC stimulation on the OC and a movable stapes, compression of the cochlear walls resulted in squeezing the fluid through the oval window and thus deteriorating the VD of the RW membrane. The stimulation site in^34^ was the inner side of the temporal bone, so the OC moved more as a rigid body. The fluid movement due to its inertia was more significant for a movable SF, yielding worse results for a glued footplate. The VD phases were comparable in the frequency range up to 3.15 kHz, and the differences for high frequencies were due to the temporal bone fixation in the presented model.

**Figure 8:**
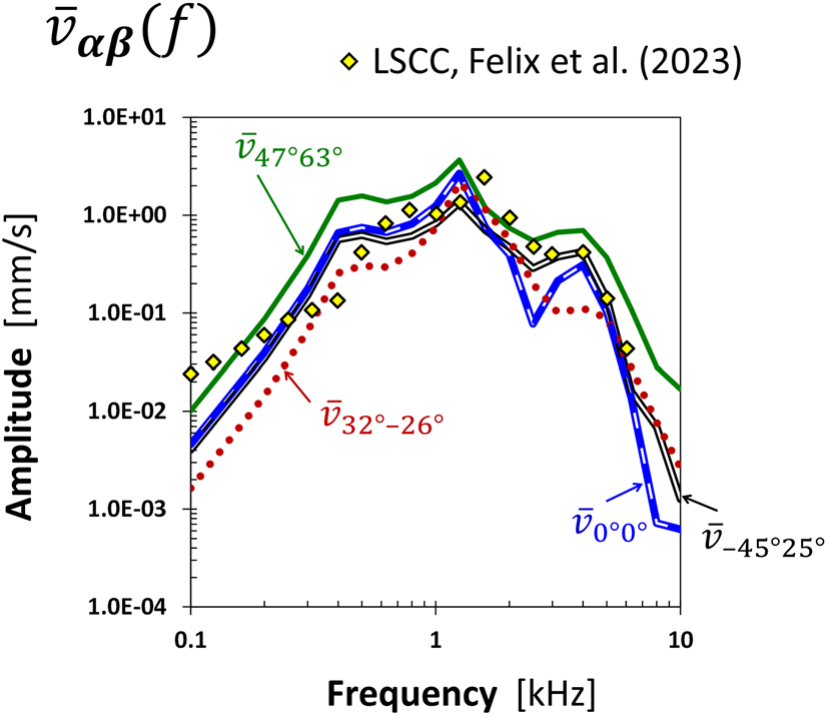
The averaged round window edge velocity in the direction perpendicular to the round window. Four directions of bone conduction stimulation compared with the cochlear promontory vibration measured on cadavers for the same stimulation site above the lateral semicircular canal (LSCC)^23^. The forces in the presented finite element model tuned to the values generated in the experiment by the B81 transducer.

Vibrations of the RW edge and at the CP are similar due to the high stiffness of the compact bone. The differences between the numerical simulation results and the experimental data (Fig. 5) were inevitable, because the human head in^14^ lay on a pillow and was excited by an additional loading system. In the presented model, fixation of the bone boundary excluded rigid body movement, which depends on the stimulation direction and is meaningful at low frequencies. Because the experiment^14^ started at 630 Hz, it needed to clarify how the temporal bone support in the presented model affected the OC vibration at lower frequencies. The effect of bone fixation was visible as differences between the experimental CP velocities obtained from studies on human heads^23^ and the RW edge vibration velocities at low frequencies (Fig. 8). The BC force applied in the simulation was set as 2.5/2.83·10^–6^·10^OFL(*f*)/20^ N to correspond to the force generated by B81 vibration transducer driven by a stepped sine wave with 2.5 V_pp_ voltage in the experiment, taking the output force level (OFL−[dB re 1μN]) of B81 driven by a sinusoidal wave with an amplitude of 1 V_rms_ measured on an artificial mastoid^35^. The RW edge vibration velocities in the presented model had similar slopes for frequencies below 400 Hz regardless of the stimulation direction. The experimental curve had a significantly lower slope caused by decreasing rigid body movement with increasing frequency (heads lay in a silicone elastomer block during the test). Since the graphs were similar above 400 Hz, the bone fixation in the presented model affected the results mainly for low frequencies.

The discrepancy between extreme intensities of the RW edge vibration and the fluid VD resulted from the unique shape of the cochlea and nonuniform distributions of mass and stiffness in the petrous part. Additionally, a single direction represented the RW edge displacement, while the VD came from the spatial movement of the OC. The maximum vibrations of the RW edge occurred for the stimulation direction (47°, 63°) that coincided with the *z*-*axis*. This direction was perpendicular to the plane containing the forces which gave small bone vibrations in the direction perpendicular to the RW. Therefore, displacements in tangent directions *x* and *y* were of greater importance for that plane. Measurements using a 3D LDV revealed that a single point on the CP had an ellipsoidal trajectory during BC stimulation^36^.

The averaged displacements of the RW edge $\overline{w}_{\alpha \beta } \left( f \right)$ depended more on the *β* angle, while the effect of *α* was not as significant. Since the *z* and *x*_00_ axes were nearly parallel (Figs. 1, 2), rotation of the force vector by any *α* angle and *β* = 0° set the *F*_*α0*_ force perpendicular to the *z*-*axis* and induced the OC vibrations primarily in the direction tangent to the RW, so that $\overline{w}_{\alpha 0^\circ } \left( f \right)$ displacements were small (Fig. 3a). Conversely, for any *α* angle and *β* values of ± 90°, the force vector coincided with the *x*_00_-*axis*, so that the OC vibrated more in the direction perpendicular to the RW, yielding significant $\overline{w}_{\alpha \pm 90^\circ } \left( f \right)$ values. Favorable interaction of the cochlear wall compression and fluid inertia effects, resulting in an intense VD of the RW membrane, were evident for the stimulation direction corresponding to $V_{max}^{crit}$ (− 45°, 25°). In comparison, for the directions (32°, − 26°) and (47°, 63°), the OC vibrated more as a rigid body up to 1.25 kHz. The primary direction (0°,0°) yielded good VD values and was almost parallel to the plane of the lateral semicircular canal. According to^8^, this direction was the safest regarding the risk of OC damage when screwing the BC implant into a bone.

As described in^4^, in the case of BC stimulation placed on the skull surface, translational and rotational vibrations occurred in three directions, and none was dominant. Vibrations travel from the stimulation site to the skull for BC stimulation on the OC. They depend on the natural vibrations of the temporal bone, and the direction of force is essential. Moreover, the proximity of the excitation force causes a decrease in bone vibration amplitudes as the distance from the stimulation site increases. As it is known, the amplitude of forced vibrations is greater if the excitation direction corresponds to an eigenvector. Depending on the vibration mode, compression of the cochlea along one axis interacts with stretching or squeezing along the other axes, which affects the stimulation intensity. Hudde et al.^37^ performed a modal analysis of the human head model to analyze spatial deformations of the cochlea. Below the frequency of 5 kHz, the compressional displacements of the OC were 25 dB as low as the translational ones, and above 5 kHz, they were about 10 dB as low.

The first natural vibrations in the presented model occurred around 0.38 kHz, when the petrous temporal bone rotated relative to the jugular process, and the OC was next to the rotation center; hence, the RW edge vibrations and the fluid VD were relatively small at low frequencies and influenced by the force direction. Stenfelt^7^ analyzed vibrations of the inner ear, assuming the movement of the bone surrounding the cochlea as a longitudinal wave. During BC stimulation on the OC, more complex deformation due to nonuniform mass and stiffness distributions inside the petrous temporal bone affects the cochlear fluid movement. Consequently, the directional sensitivity influences the VD value of the RW membrane. Compression of the cochlear walls at low frequencies occurred because the vibration amplitudes were decreasing with increasing distance from the excitation point. The vibration modes responsible for the increase of the RW edge displacement at frequencies close to 1.25 kHz (Fig. 5a) were observed as translational and rotational movements of the OC relative to the jugular process, coupled with compression-extension of the cochlea and deflection of the apex of the petrous part (see Supplementary Video S2 online). At 1.25 kHz, the RW edge vibration amplitudes were almost independent of the stimulation direction, except for (− 45°, 25°) and (− 49°, 1°), where the displacement components in directions tangent to the RW were much greater than the component along the z-axis.

Similarly, the VD amplitude at 1.25 kHz was almost unaffected by the stimulation direction, except for the direction of $V_{max}^{crit}$ (− 45°, 25°), where the cochlear wall compression and fluid inertia effects were the most significant (Fig. 5b). The frequency of 1.25 kHz was close to the first resonant frequency of a skull given in^34^. For the primary direction (0°, 0°) at 2.5 kHz, there was a rotational movement of the OC relative to the point close to the RW, associated with a significant decrease of the RW edge displacement (Fig. 5a) and caused by anti-resonance between 1.25 and 3.15 kHz. In contrast, the fluid VD did not drop due to the intense deformation of the cochlear walls (Fig. 5b). The increase of the RW edge displacement around 3.15 kHz for (− 45°, 25°), (0°, 0°), and (47°, 63°) directions corresponded to the next vibration modes. The VD amplitudes for directions (47°, 63°) and (32°, − 26°) at 3.15 kHz had similar levels, despite slightly different modes. Additionally, compression of the cochlear walls at high frequencies depended on the phase differences between points of the OC. It was evident as a traveling wave in the labyrinth, vestibule, and cochlea, causing the fluid to be alternately squeezed out and pushed.

The direction of weak vibrations (32°, − 26°), obtained by crossing both criteria of a weighted sum, corresponded to the direction of piston-like stapes movement existing for a healthy annular ligament. However, the ligament in the presented model was stiff (otosclerotic condition) and did not influence the results, being a fragment of the bony labyrinth. According to^38^, which listed Békésy and Bárány postulates, the anatomy and physiology of the middle ear limit the sensitivity of the hearing organ to one’s voice because vibrations of the vocal cords transmitted to the inner ear through BC may distort external signals received by air conduction. In the presented model, the force direction perpendicular to the SF resulted in weak amplitudes of the fluid VD at low and high frequencies (Fig. 5b) where the OC moved along the force direction. For medium frequencies, the direction of the OC movement changed due to the influence of eigenvectors, and the fluid VD amplitudes increased. Therefore, shaking the cochlea perpendicularly to the SF resulted in weak fluid vibrations. The differences between the movable and immobilized stapes were only a few dB (Fig. 7). Hence, the bone vibrations evoked by the vocal cords in the direction perpendicular to the SF yield weak VD amplitudes, only moderately affecting the fluid VD produced by the moving stapes in the case of a healthy annular ligament. However, vibrations evoked by vocal cords in other directions can give greater VD amplitudes, interfering with external signals received by air conduction, so this phenomenon needs further research.

Interestingly, directions of the most intense fluid VD according to $V_{max}^{crit}$ (− 45°, 25°) and the highest BM velocity obtained by Kim et al.^24^ were almost tangent to the SF. The latter direction corresponded to the pair of angles (− 65°, − 45°) in the presented model, where the fluid VD was only 35% of $V_{max}^{crit}$ (Fig. 3b). The angle between both directions was 75°. The plausible reason was the exclusion of bone compression by setting a high Young's modulus in^24^ and the fact that fixation of the SF simulated an otosclerosis condition in the presented model. It indicated that fluid inertia is more significant than the cochlear wall compression in the case of a movable stapes.

For BCHAs intended for the skull surface, the stimulation direction was usually perpendicular to the implantation site. For the mastoid process, e.g., as for the implant (g) in^19^, the stimulation direction corresponded to *α* = 30° and *β* = 10° in the presented model. This pair of angles yielded significant RW edge vibrations of 60% $\overline{w}_{max}^{crit}$ (Fig. 3a) and moderate fluid VD of 45% $V_{max}^{crit}$ (Fig. 3b). However, these results apply to the BC implant mounted on the skull surface and screwed into the OC, where the housing isolates vibrations from the skull surface.

Another issue related to directional sensitivity is transcranial attenuation (TA), resulting in diverse levels of signals on the ipsilateral and contralateral sides of the skull. According to the human cadaver study^39^, the highest contralateral transmission of bone vibrations occurred at the intermediate frequency range with the contralateral BC stimulation direction. TA varies between stimulation sites, and its high value is advantageous in the case of unilateral conductive hearing loss^40^. On the other hand, low TA can improve the contralateral routing of sound for single-side deafness, as observed for two commercially available BC hearing aids^41^. Moreover, cross-head transmission is undesirable for binaural BC stimulation^42^ and requires cancellation systems to improve the spatial perception of sound^43^. Assuming the small size of the BC implant and low energy demand, the stimulation on the OC should result in a high TA value, considering that merely shifting the stimulation site towards the cochlea increases the difference between vibration levels on the ipsilateral and contralateral sides^4^. Thus, the optional use of small implants on both sides of the head, placed close to the inner ear and not causing vibrations of the entire skull, may benefit speech understanding in difficult acoustic conditions and improve sound source localization.

Knowledge of directional sensitivity may enable the development of BC devices for implantation close to the OC. Miniaturization of the implant reduces the vibration energy and extends the battery life. Placing the implant under the outer skull surface reduces the risk of inflammatory response. Due to the deep location, small size, and low stimulating force, attaching the implant to the bone using an adhesive is worth considering to avoid invasive procedures on the OC.

## Conclusions

Numerical simulations have shown that the BC stimulation direction affects the cochlear wall compression and fluid inertia effects in the entire hearing range. The results concern the finite element model with a supported temporal bone boundary and an otosclerotic condition on the stapes footplate. The intensity of the fluid VD of the RW membrane was not proportional to the bone vibration amplitude at the RW edge in the direction perpendicular to the window. The force direction corresponding to the most intense excitation of the fluid VD of the RW membrane was a) inclined at a slight angle to the RW membrane, b) roughly perpendicular to the modiolus axis, c) almost tangent to the SF. As expected, the force direction perpendicular to the RW generated the best vibrations of the RW edge in this direction. However, the fluid VD of the RW membrane for the same direction was not maximal.

The direction corresponding to the minimum of the fluid VD was at a slight angle to the direction perpendicular to the RW. In contrast, the direction giving the minimum vibrations of the RW edge was at a slight angle to the direction corresponding to the VD maximum. The direction perpendicular to the SF yielded small vibrations of the RW edge in the direction perpendicular to the window and weak fluid VD amplitudes. The primary direction of stimulation, optimal from the surgeon's point of view, was the direction of moderate RW edge vibrations and high amplitudes of the fluid VD.

The discrepancies between the RW edge vibration in the direction perpendicular to the window and the fluid VD indicated that intensities of the fluid and bone excitations do not coincide. Checking the influence of the force direction on a 3D movement of the RW edge and the excitation intensity of the BM and the spiral lamina inside the cochlea requires further research, assuming the extreme directions determined in this work.

### Supplementary Information

Supplementary Information 1.

Supplementary Information 2.

## Data Availability

The datasets generated during and/or analyzed during the current study are available from the corresponding author on reasonable request.
