# Supplementary material for: Directional sensitivity of bone conduction stimulation on the otic capsule in a finite element model of the human temporal bone
Source: Sci Rep. 2024 Jun 14;14:13768. doi: 10.1038/s41598-024-64377-x (PMC11178818; doi:10.1038/s41598-024-64377-x)
Supplement: Supplementary file 1 — Supplementary Information 1. [file 41598_2024_64377_MOESM1_ESM.pptx]

## Slide 1
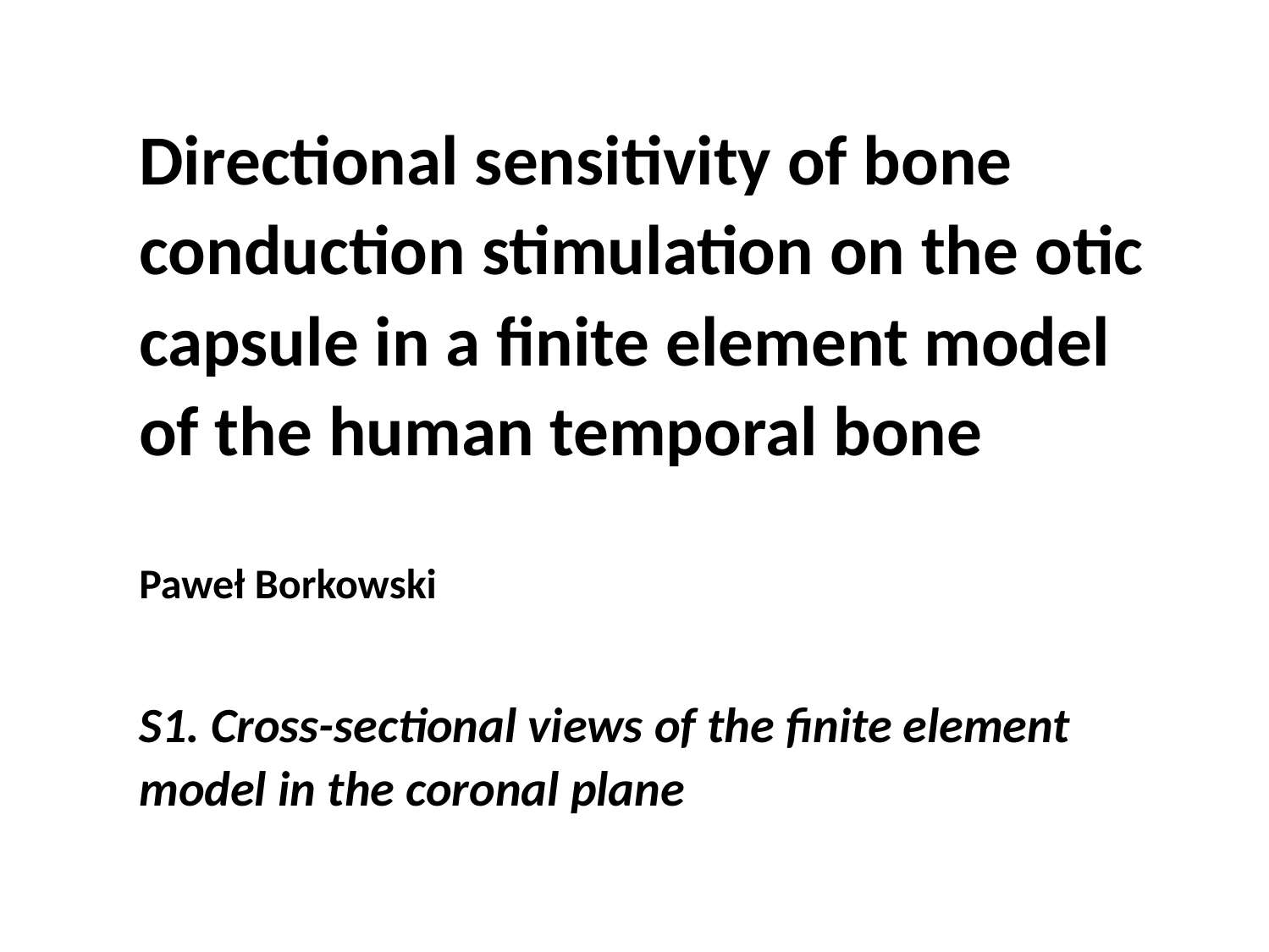

Directional sensitivity of bone conduction stimulation on the otic capsule in a finite element model of the human temporal bone
Paweł Borkowski
S1. Cross-sectional views of the finite element model in the coronal plane

## Slide 2
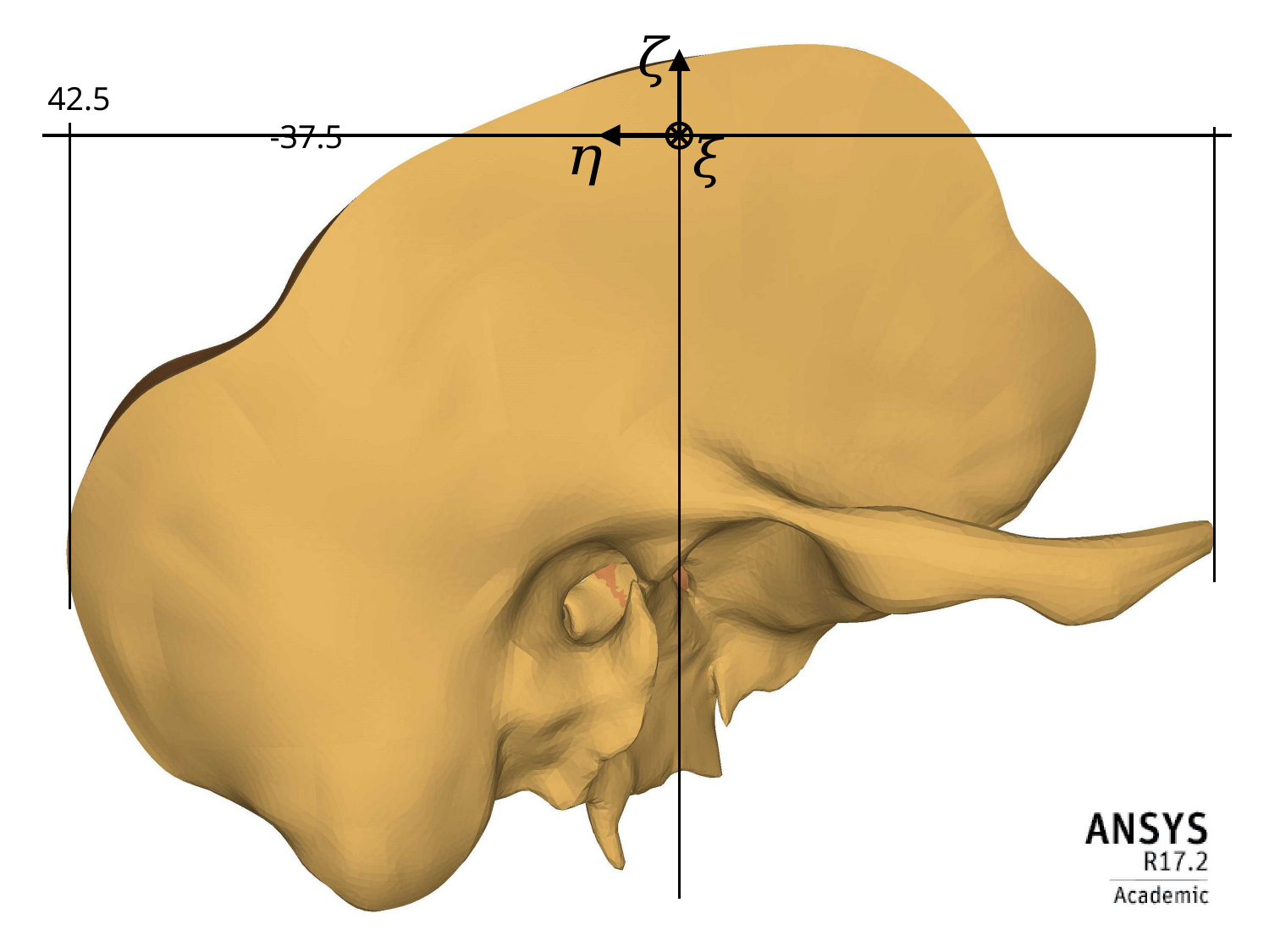

42.5 -37.5

## Slide 3
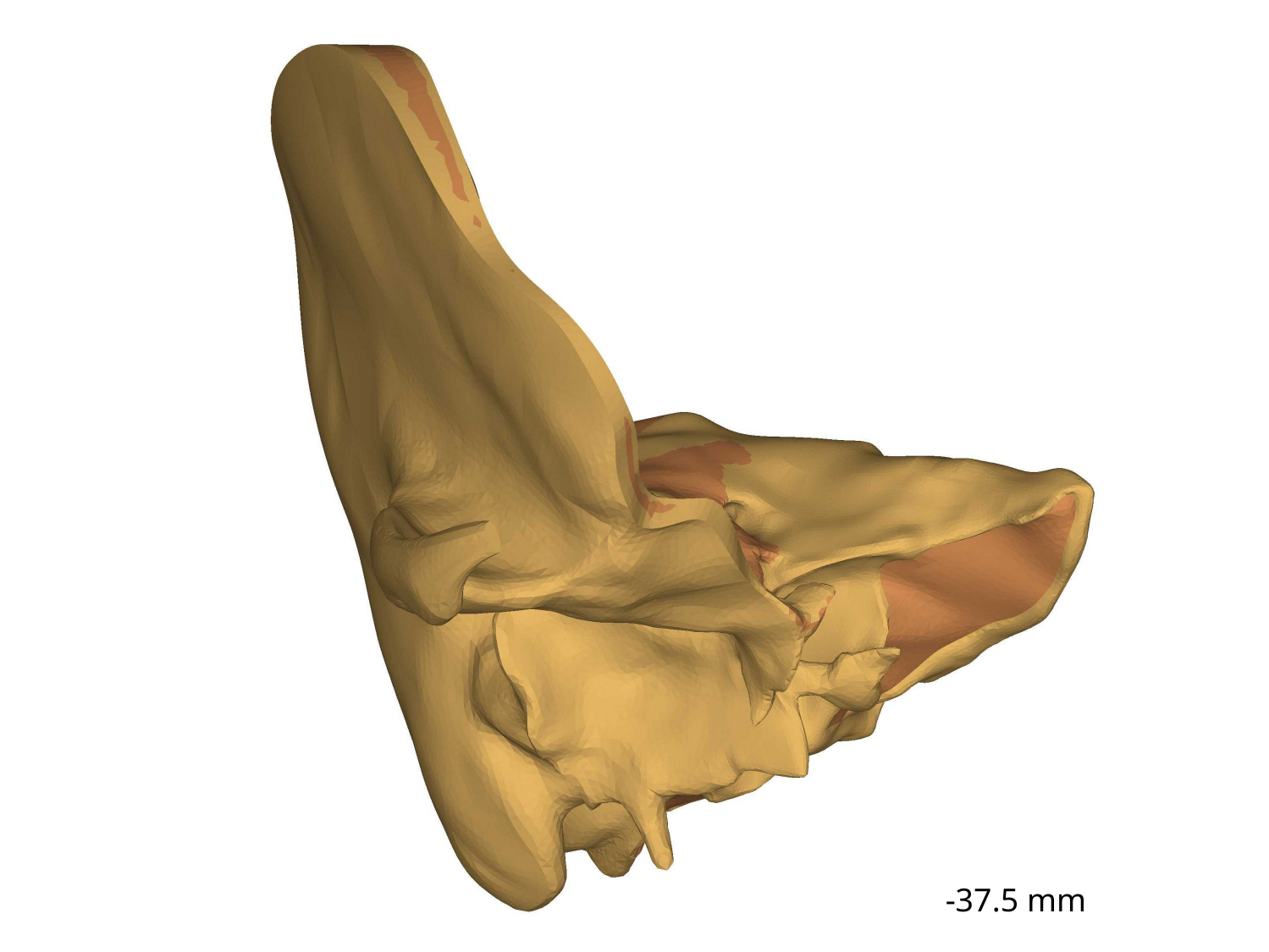

-37.5 mm

## Slide 4
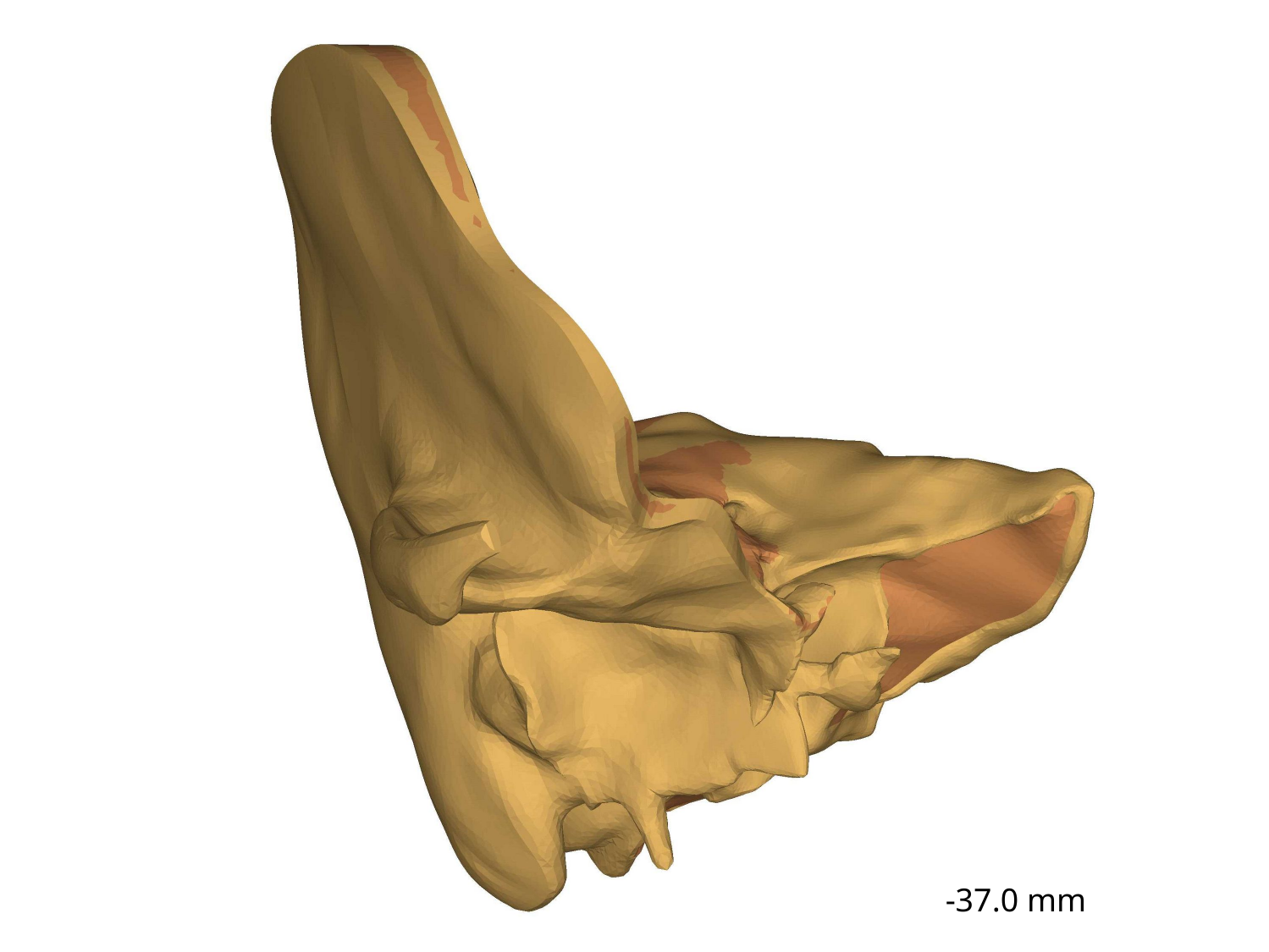

-37.0 mm

## Slide 5
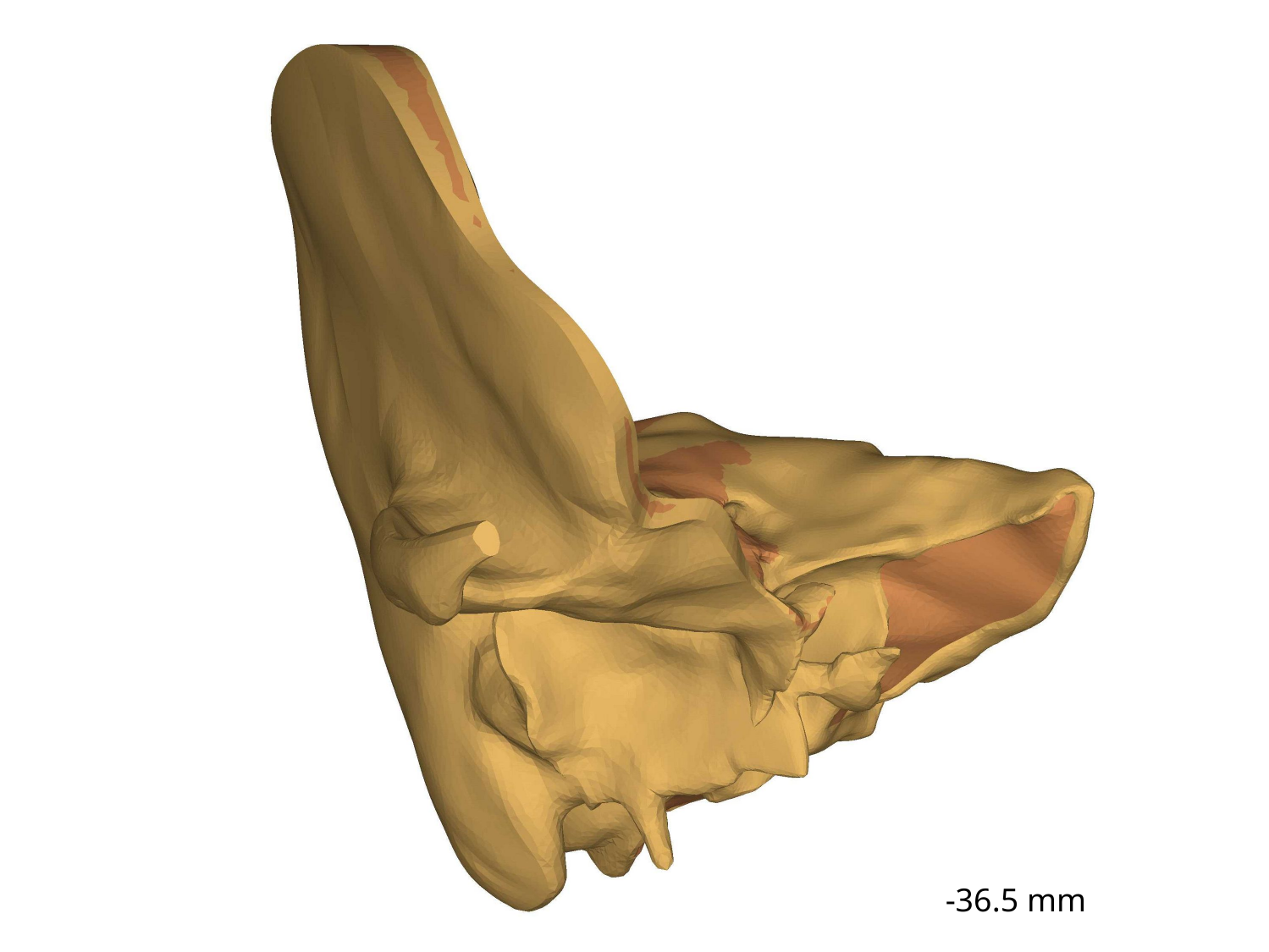

-36.5 mm

## Slide 6
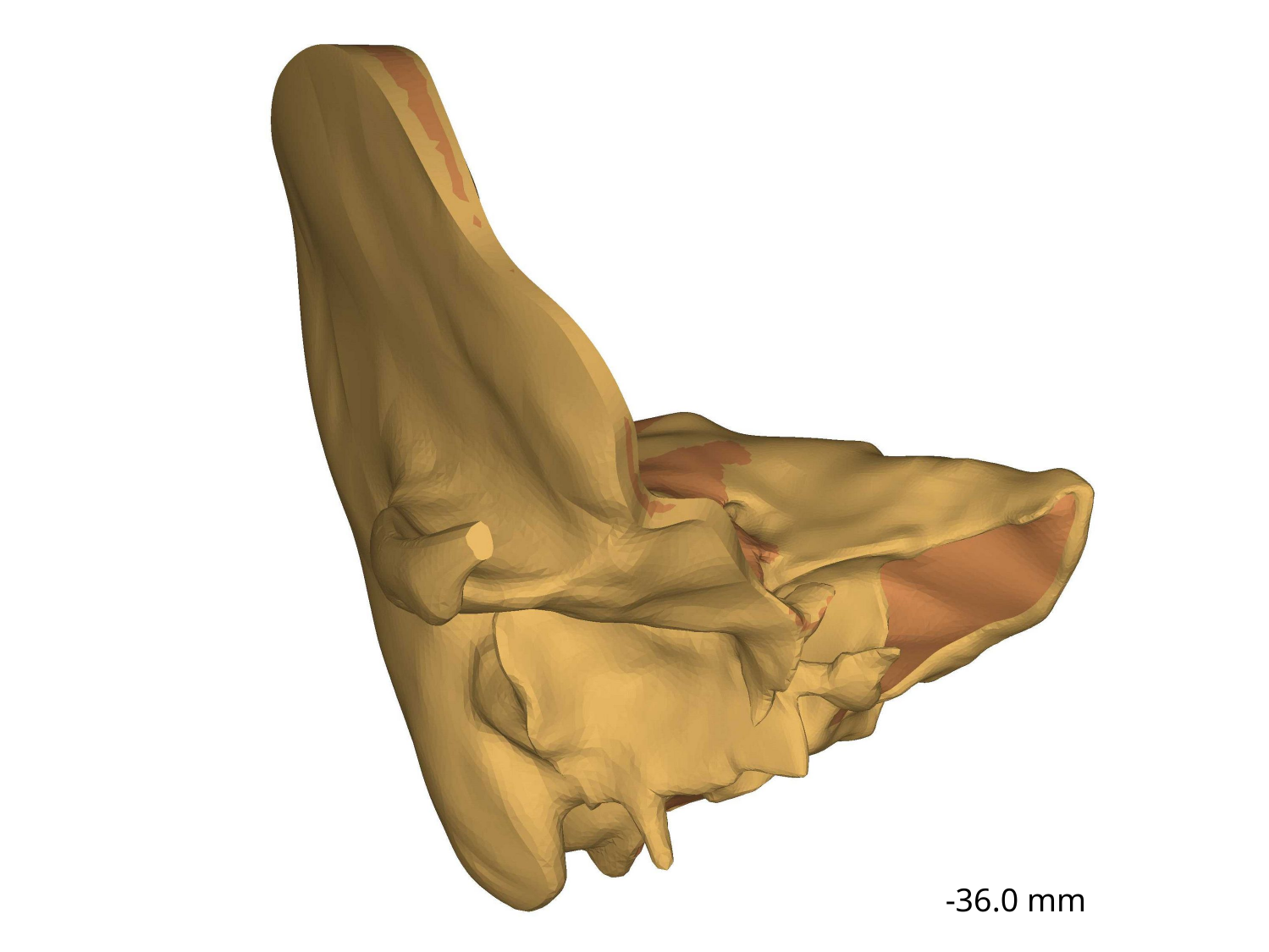

-36.0 mm

## Slide 7
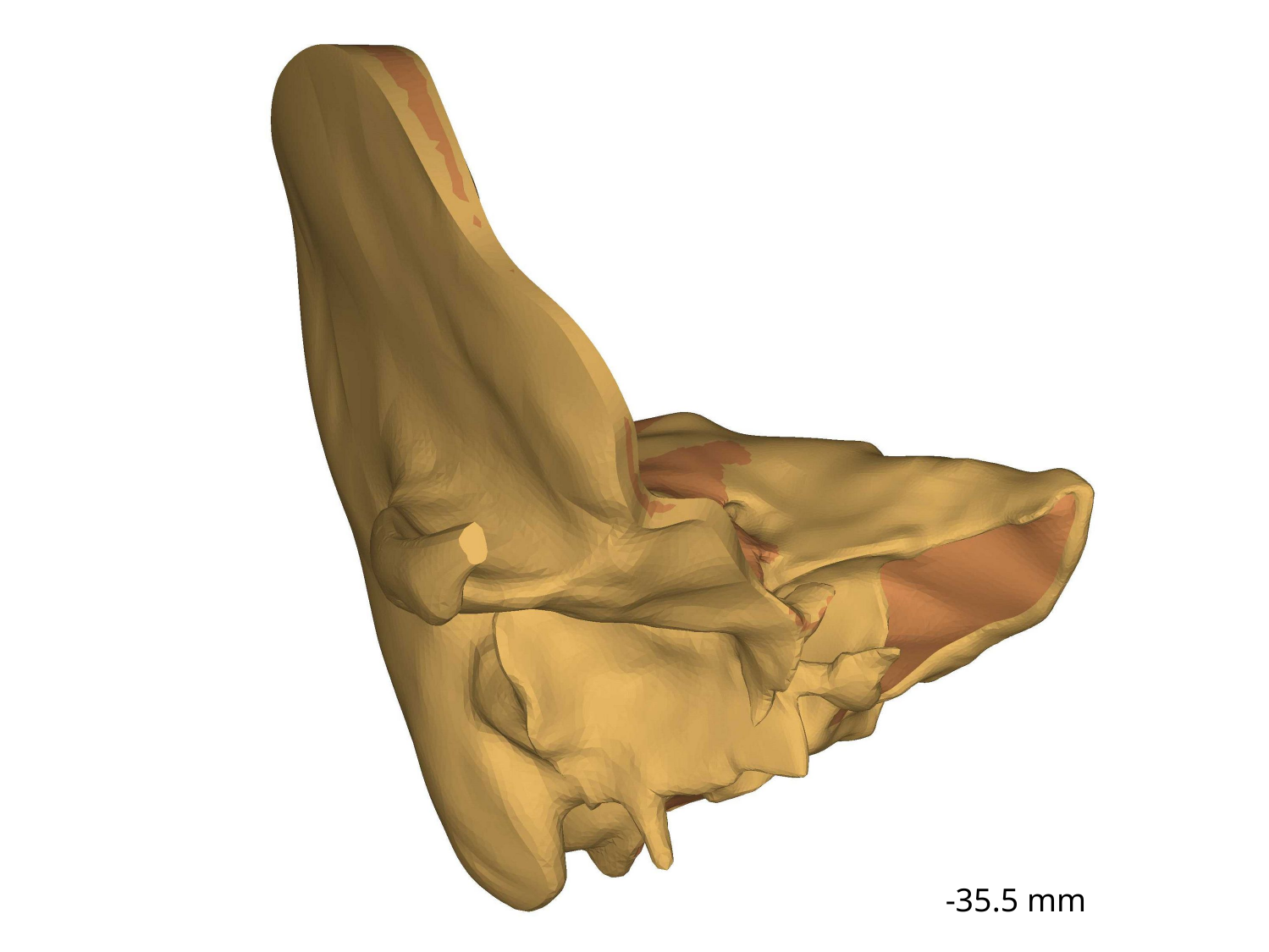

-35.5 mm

## Slide 8
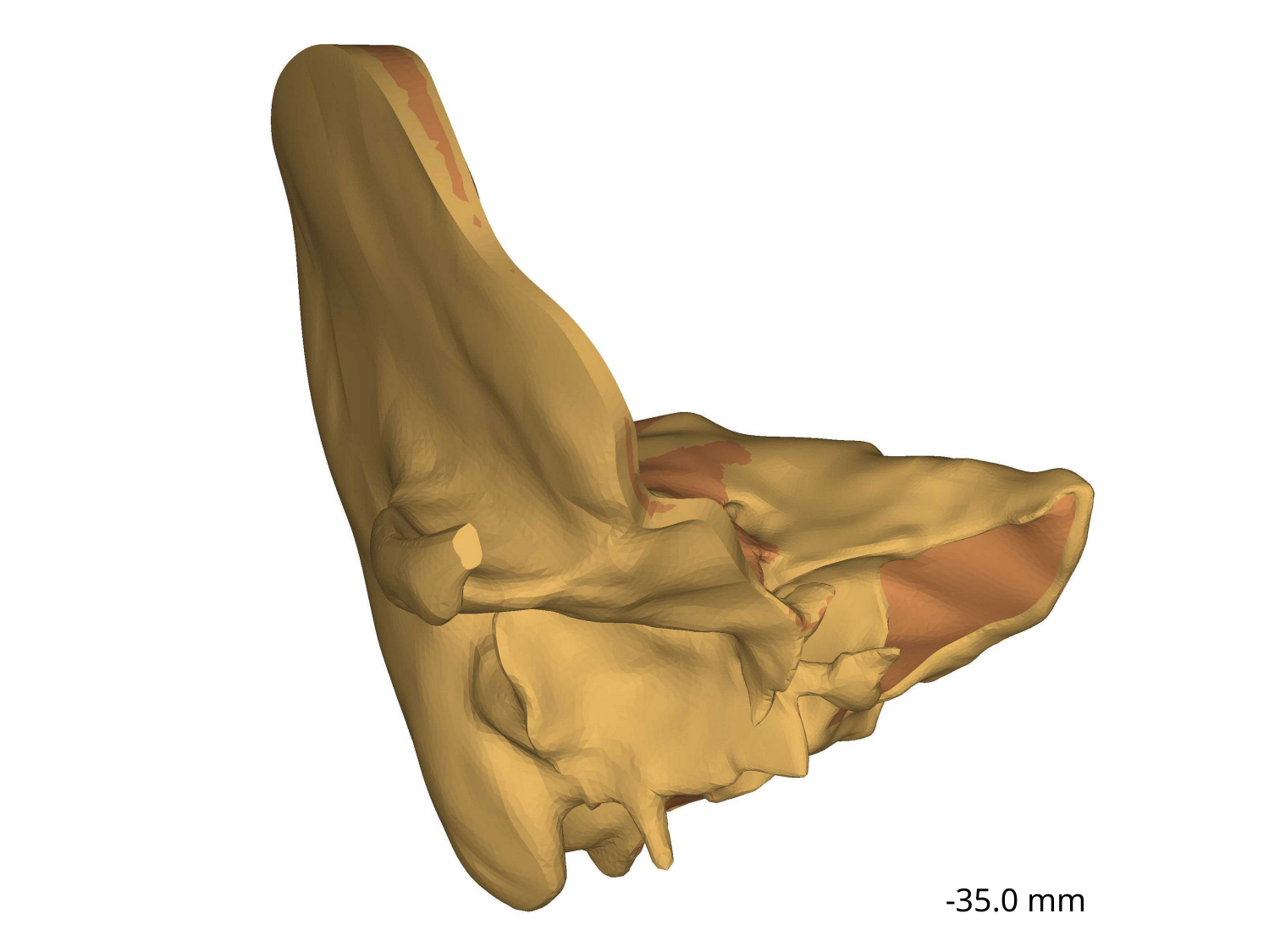

-35.0 mm

## Slide 9
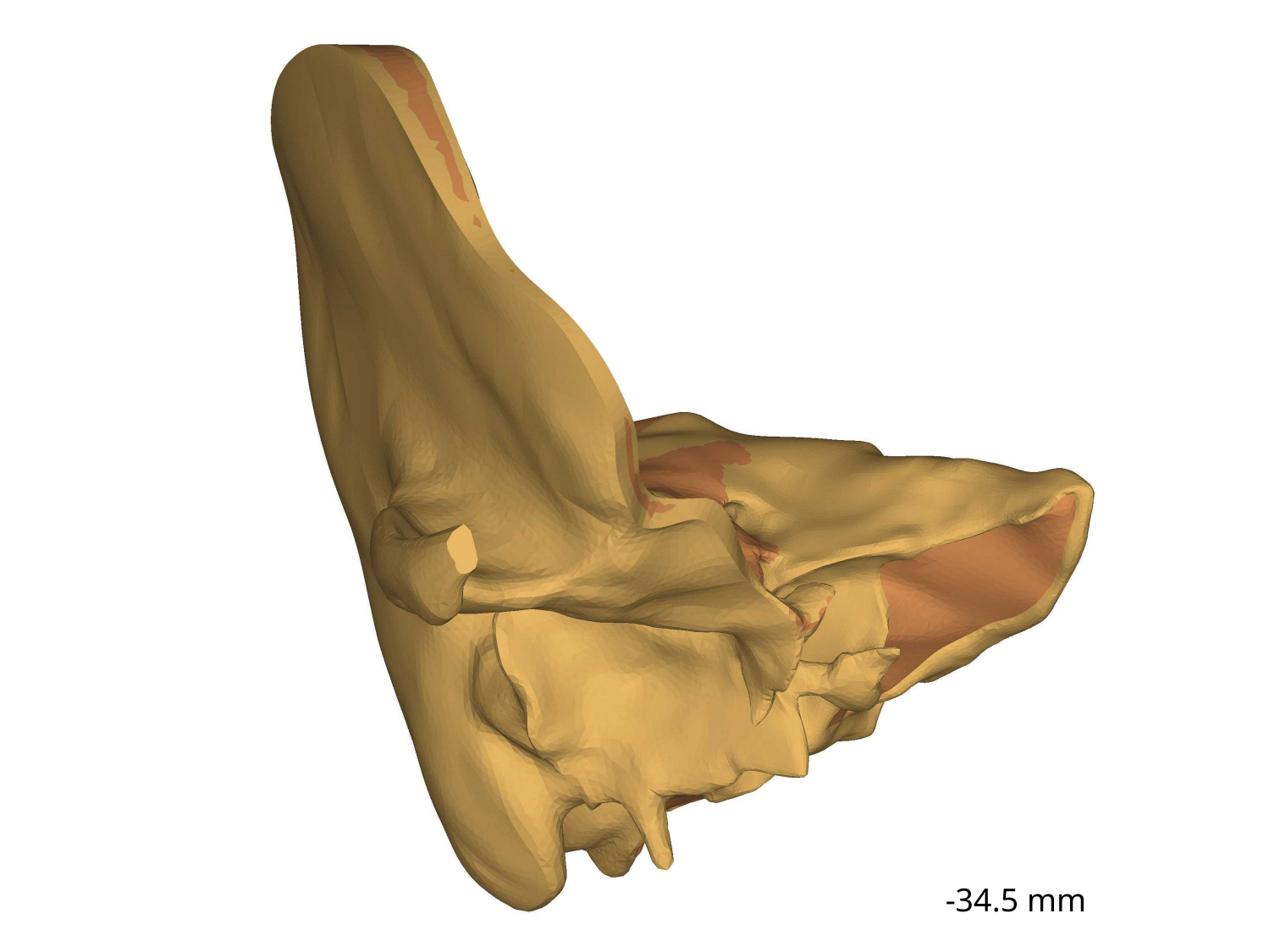

-34.5 mm

## Slide 10
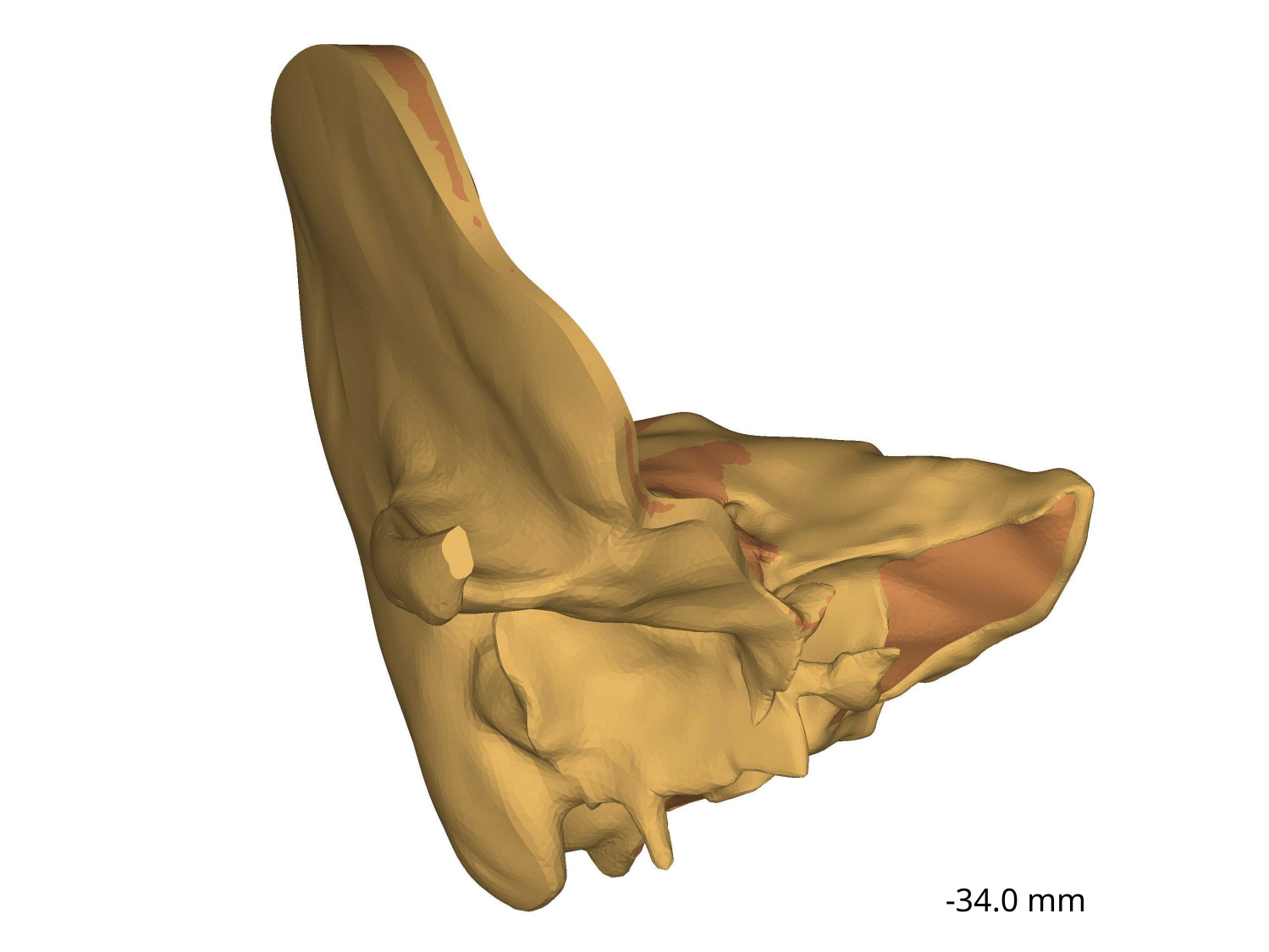

-34.0 mm

## Slide 11
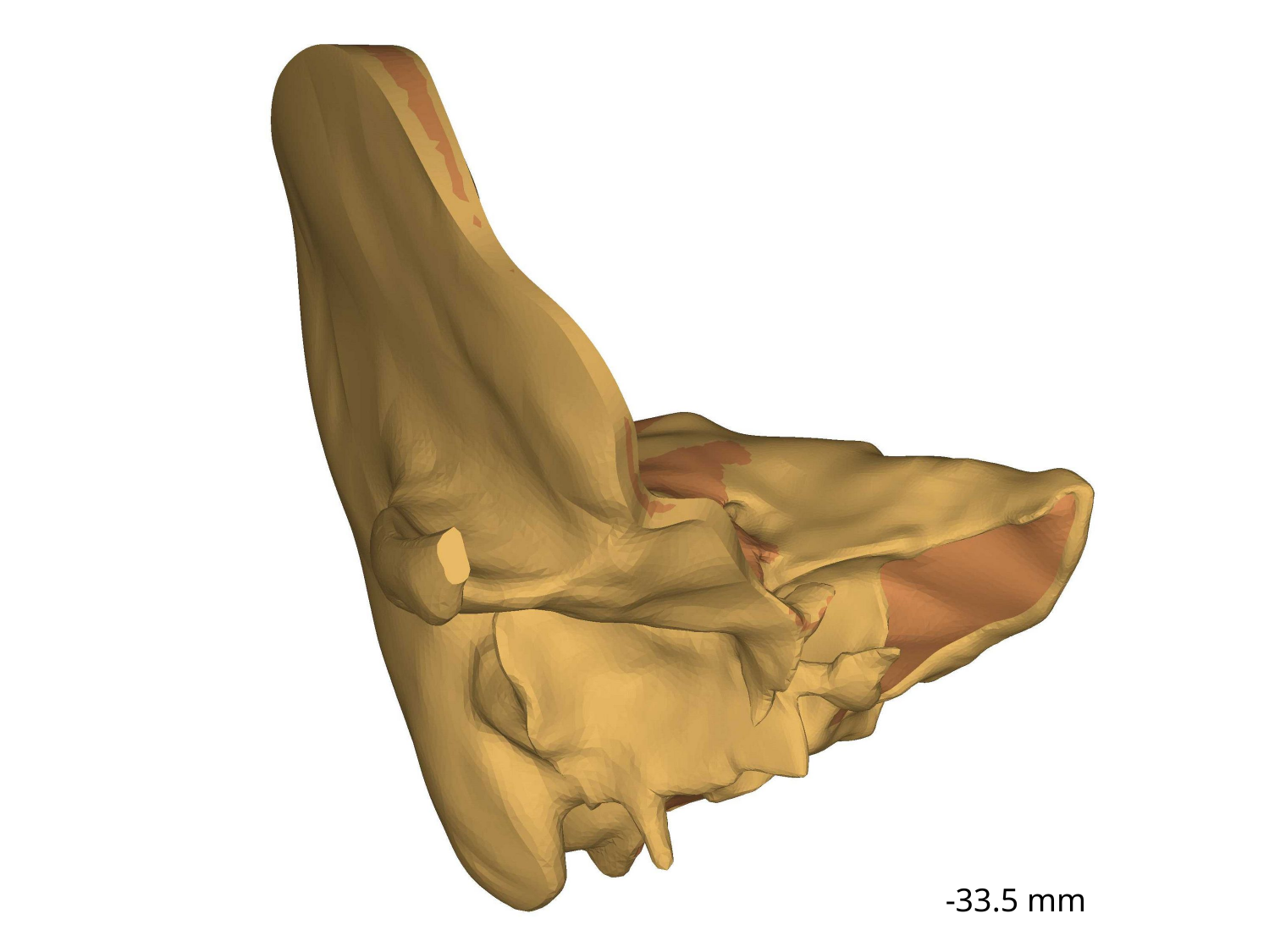

-33.5 mm

## Slide 12
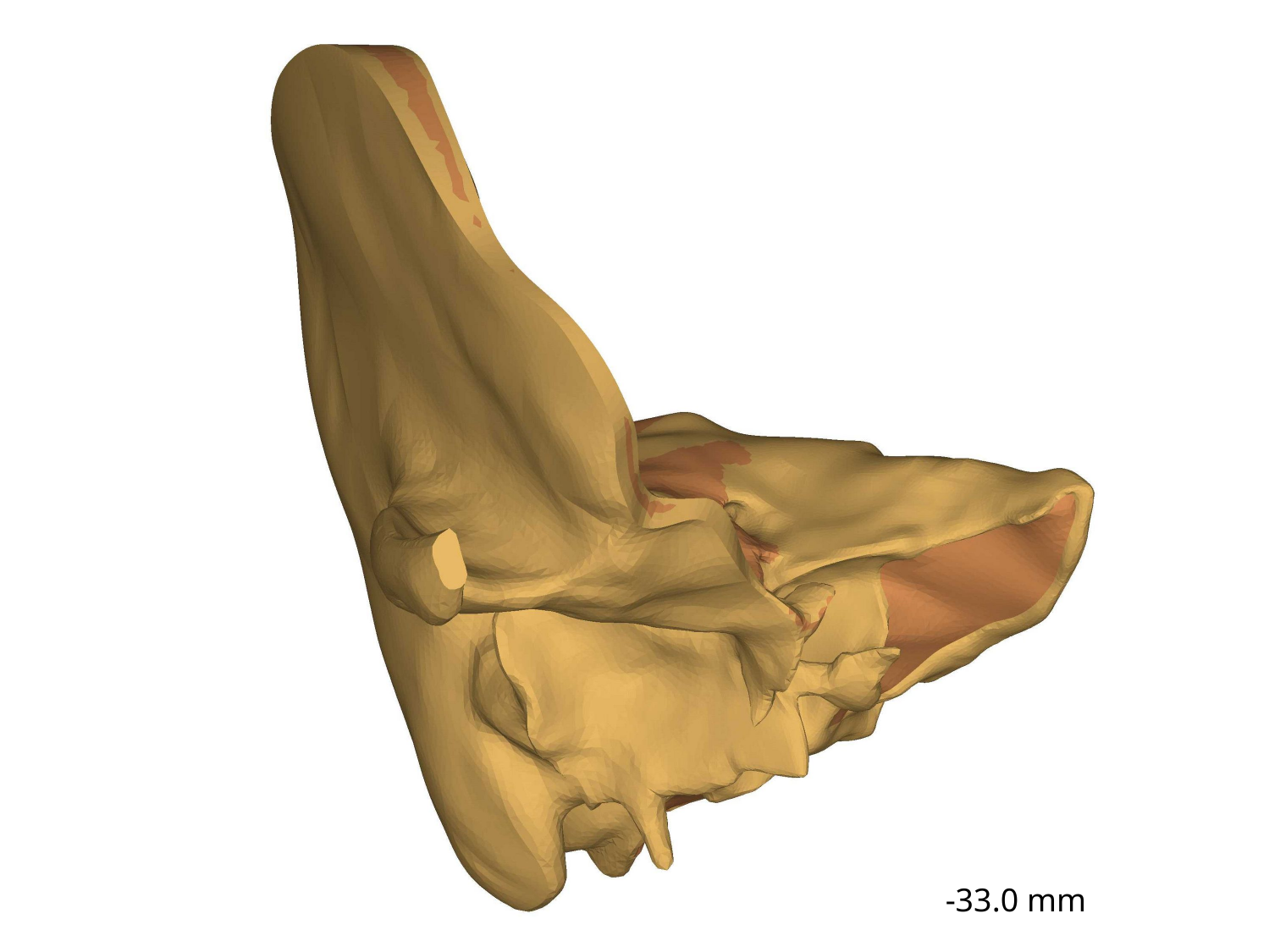

-33.0 mm

## Slide 13
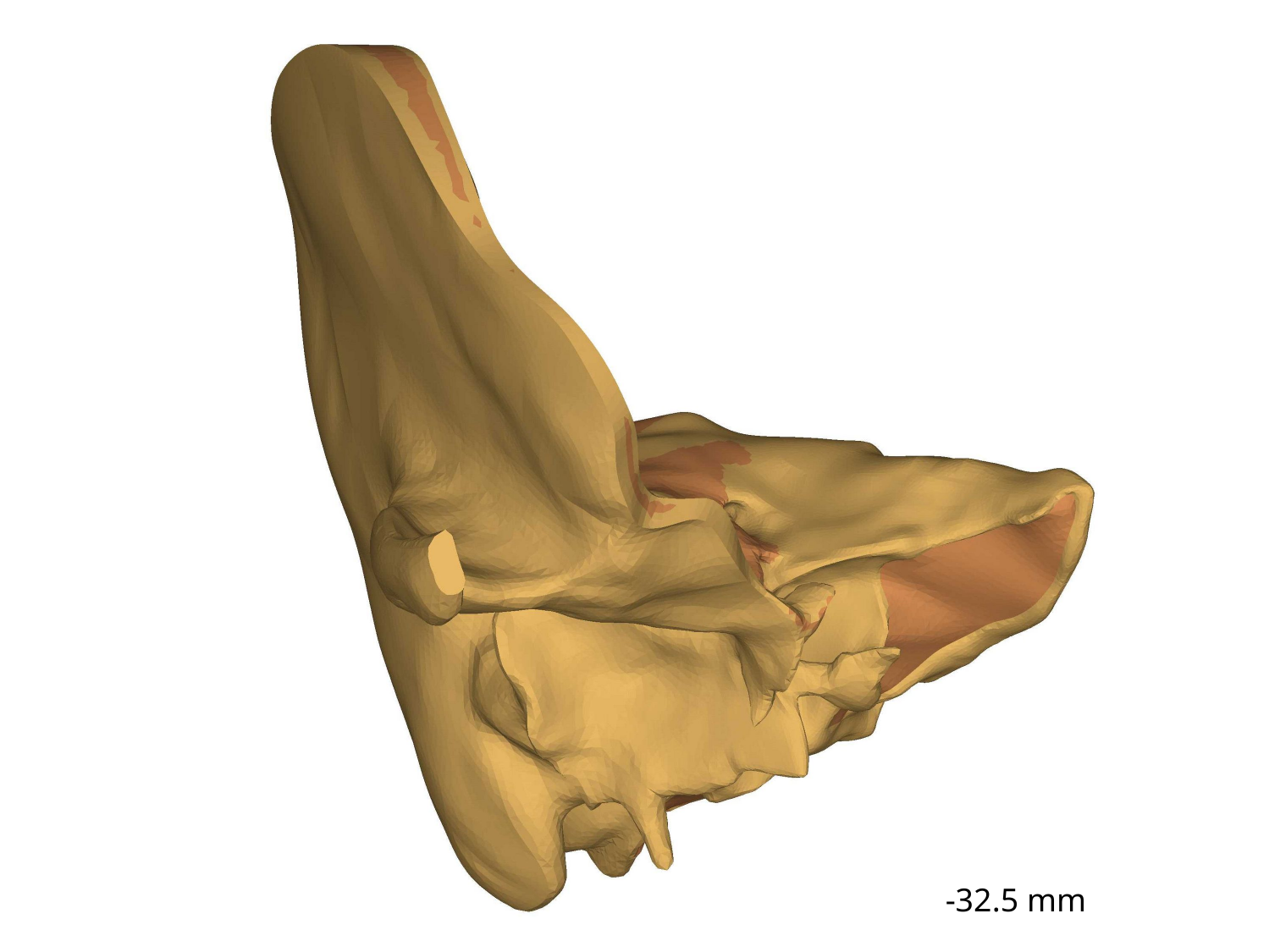

-32.5 mm

## Slide 14
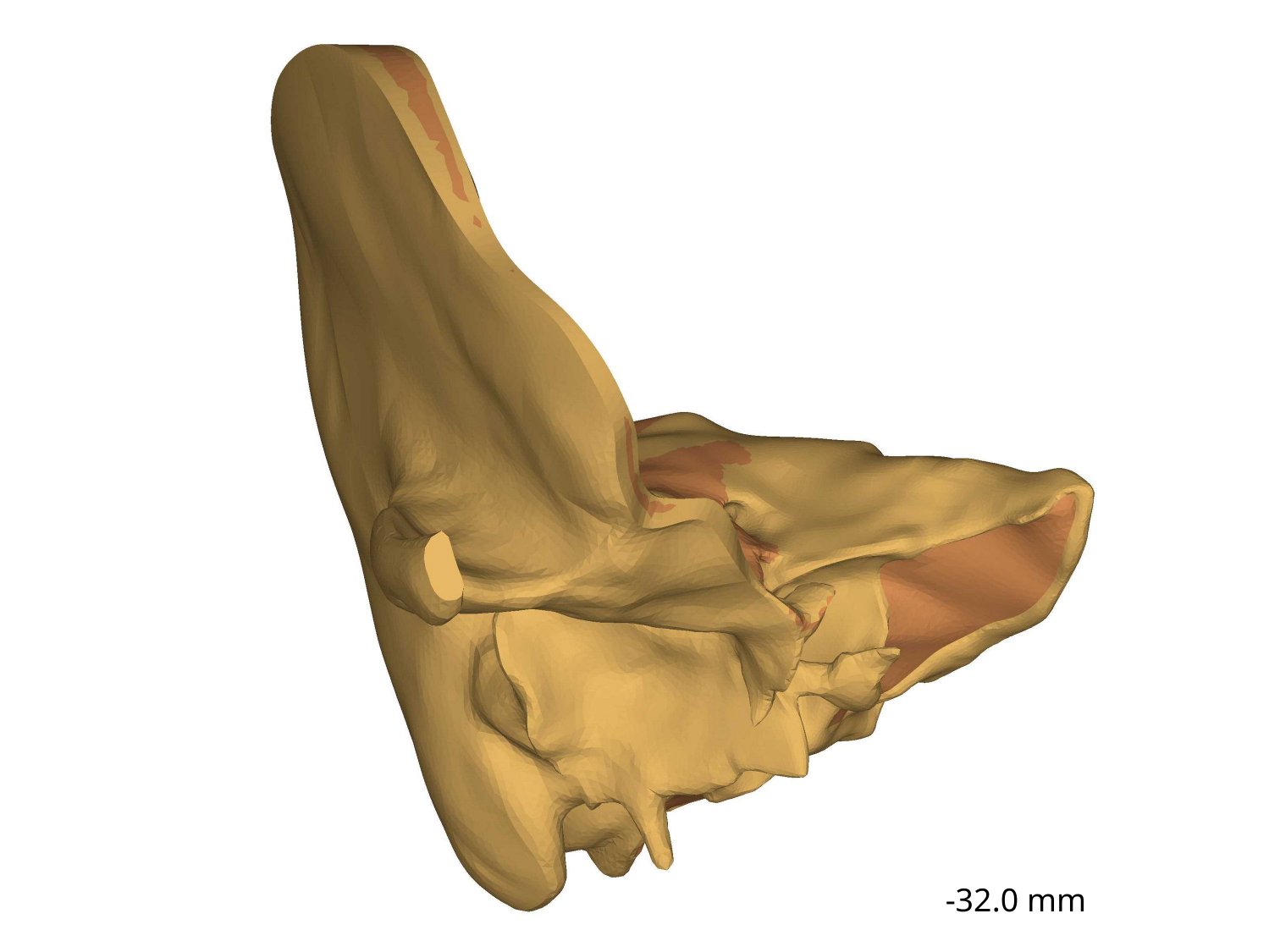

-32.0 mm

## Slide 15
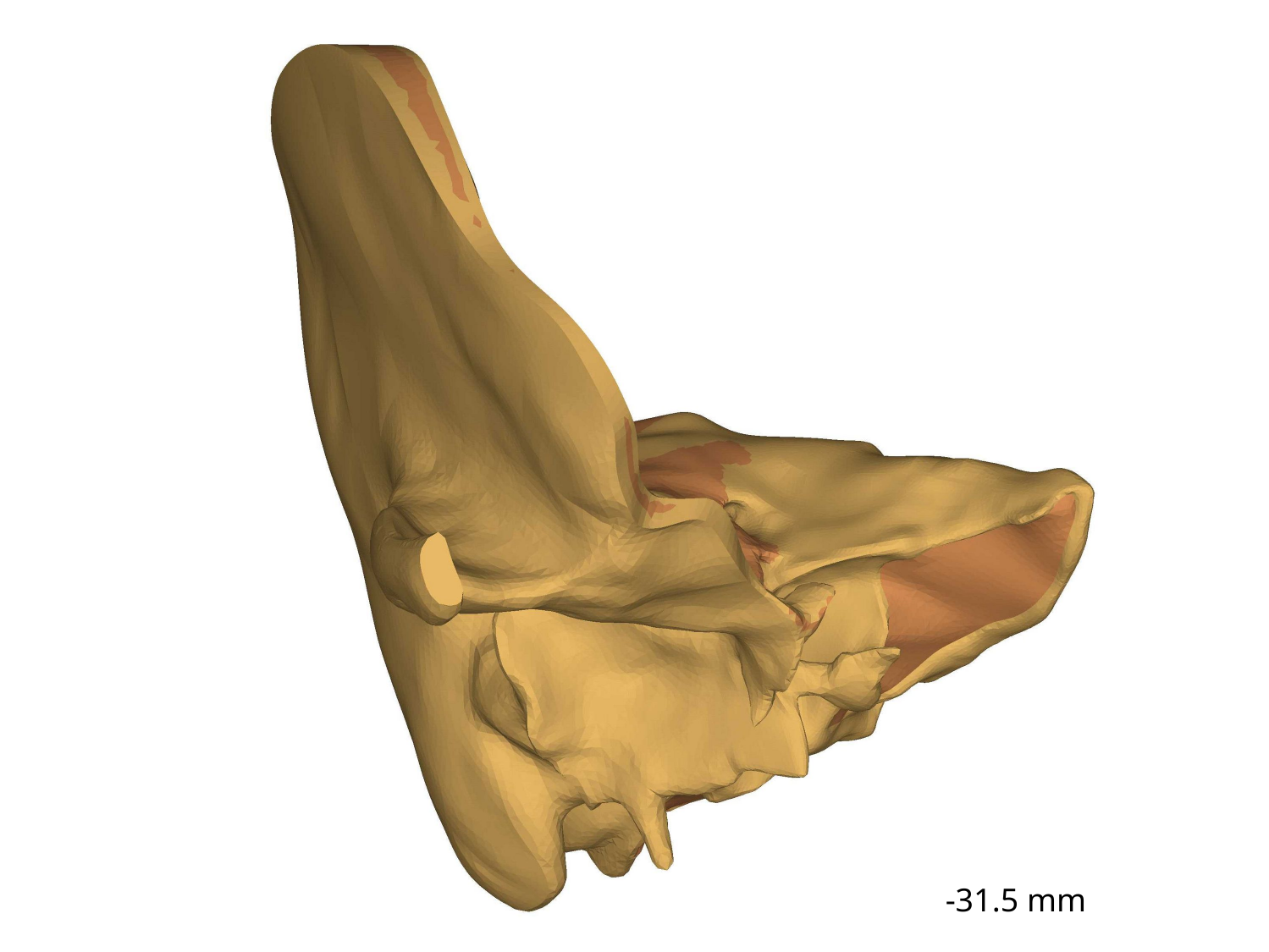

-31.5 mm

## Slide 16
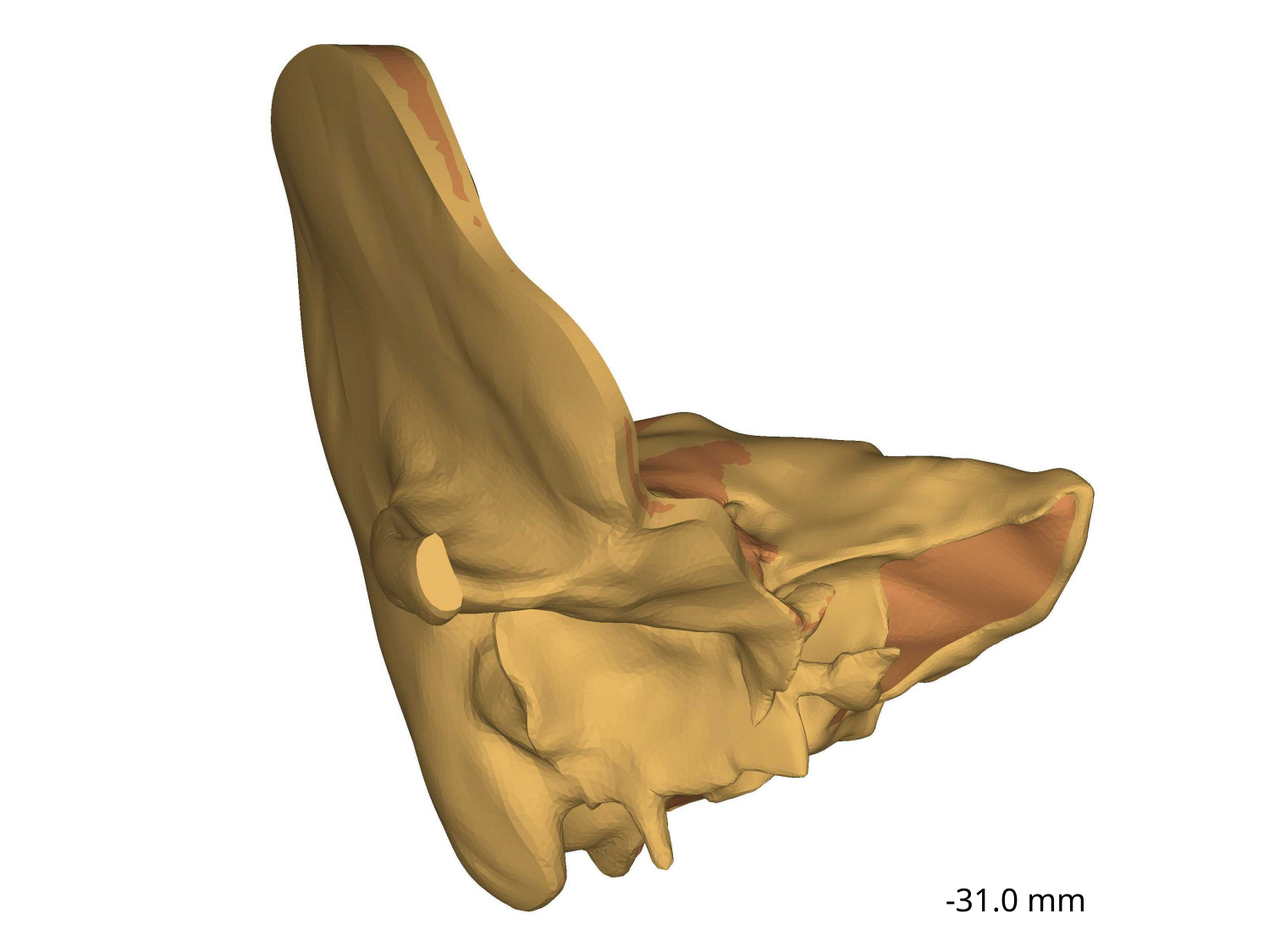

-31.0 mm

## Slide 17
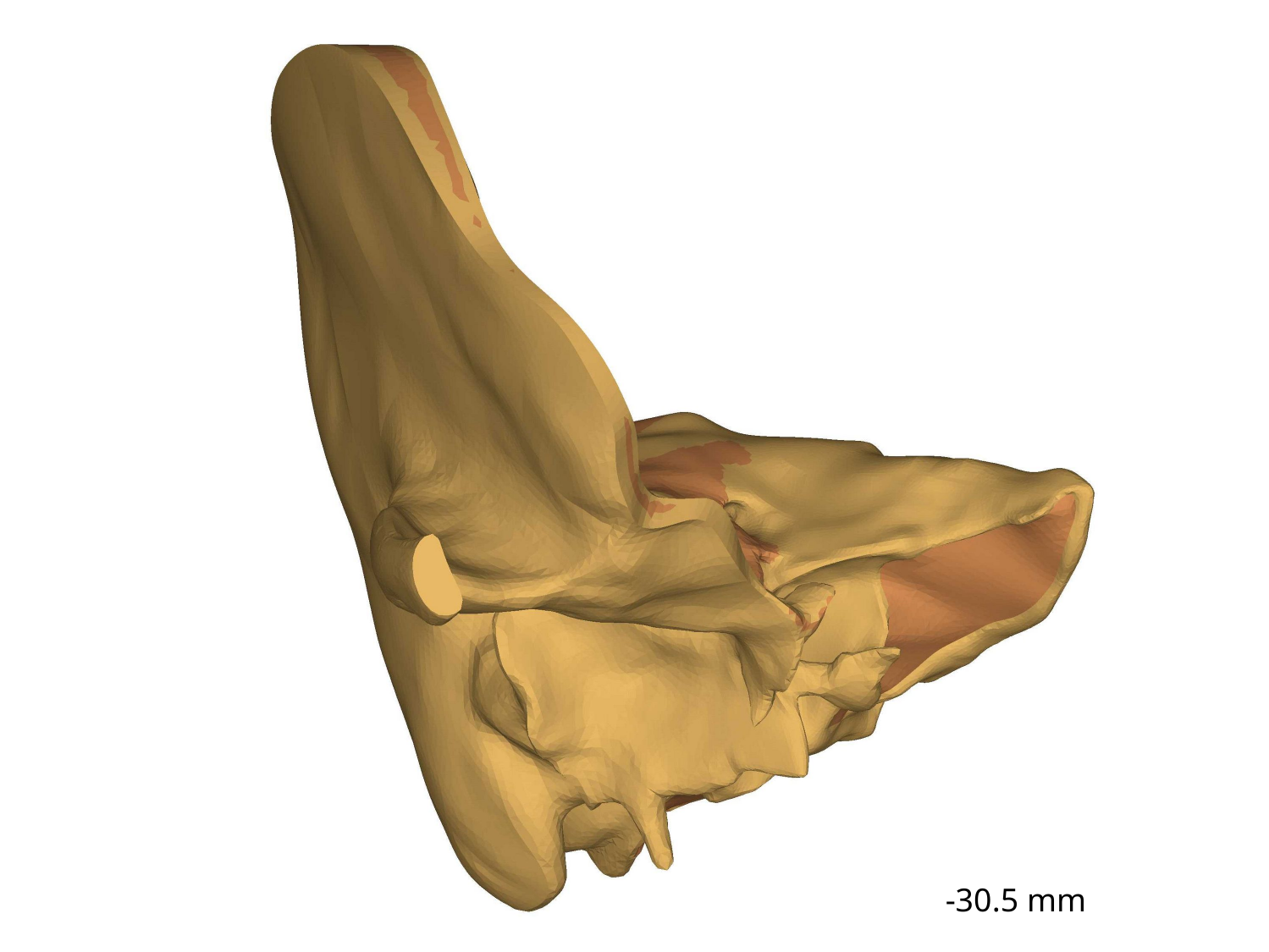

-30.5 mm

## Slide 18
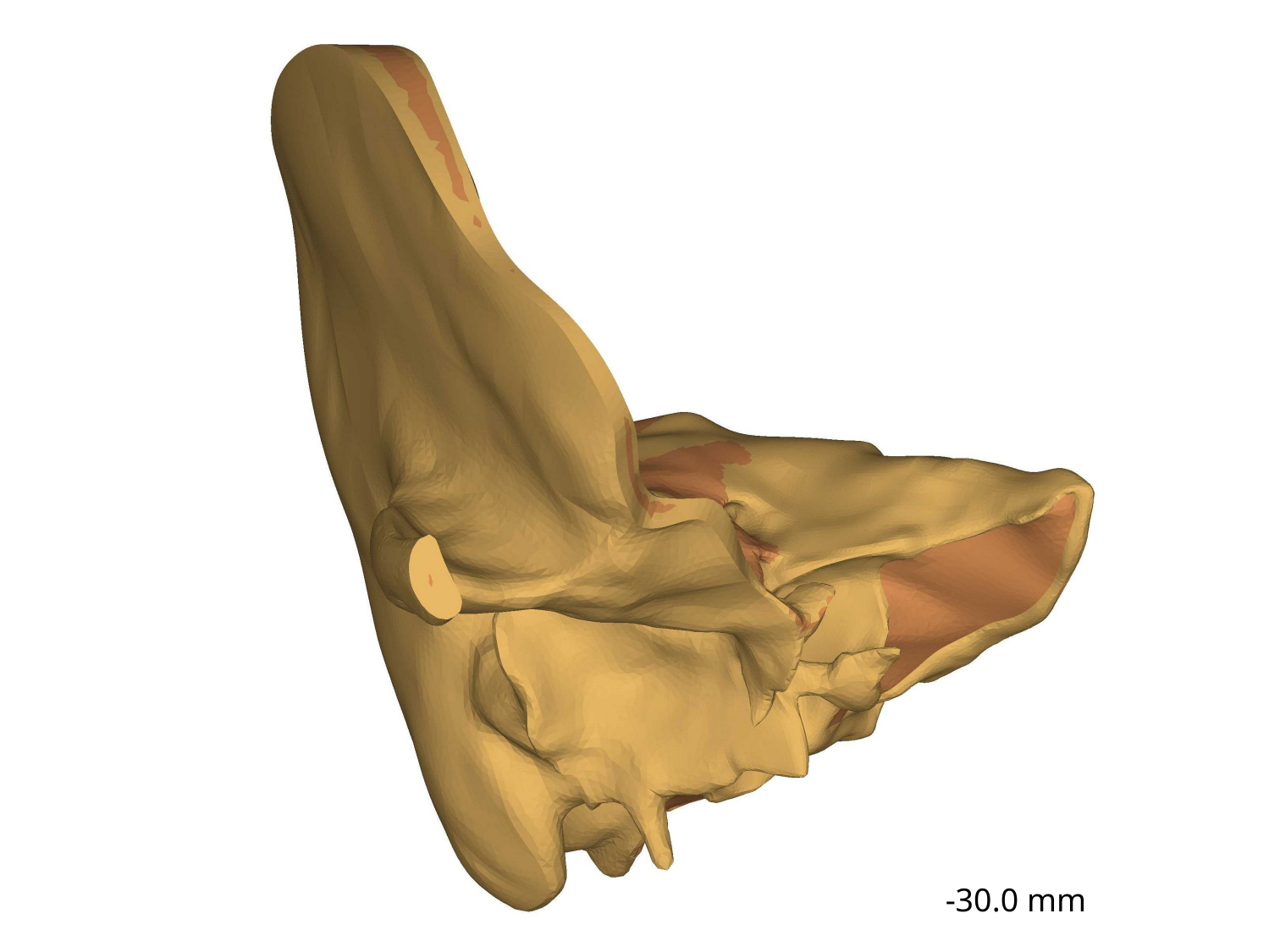

-30.0 mm

## Slide 19
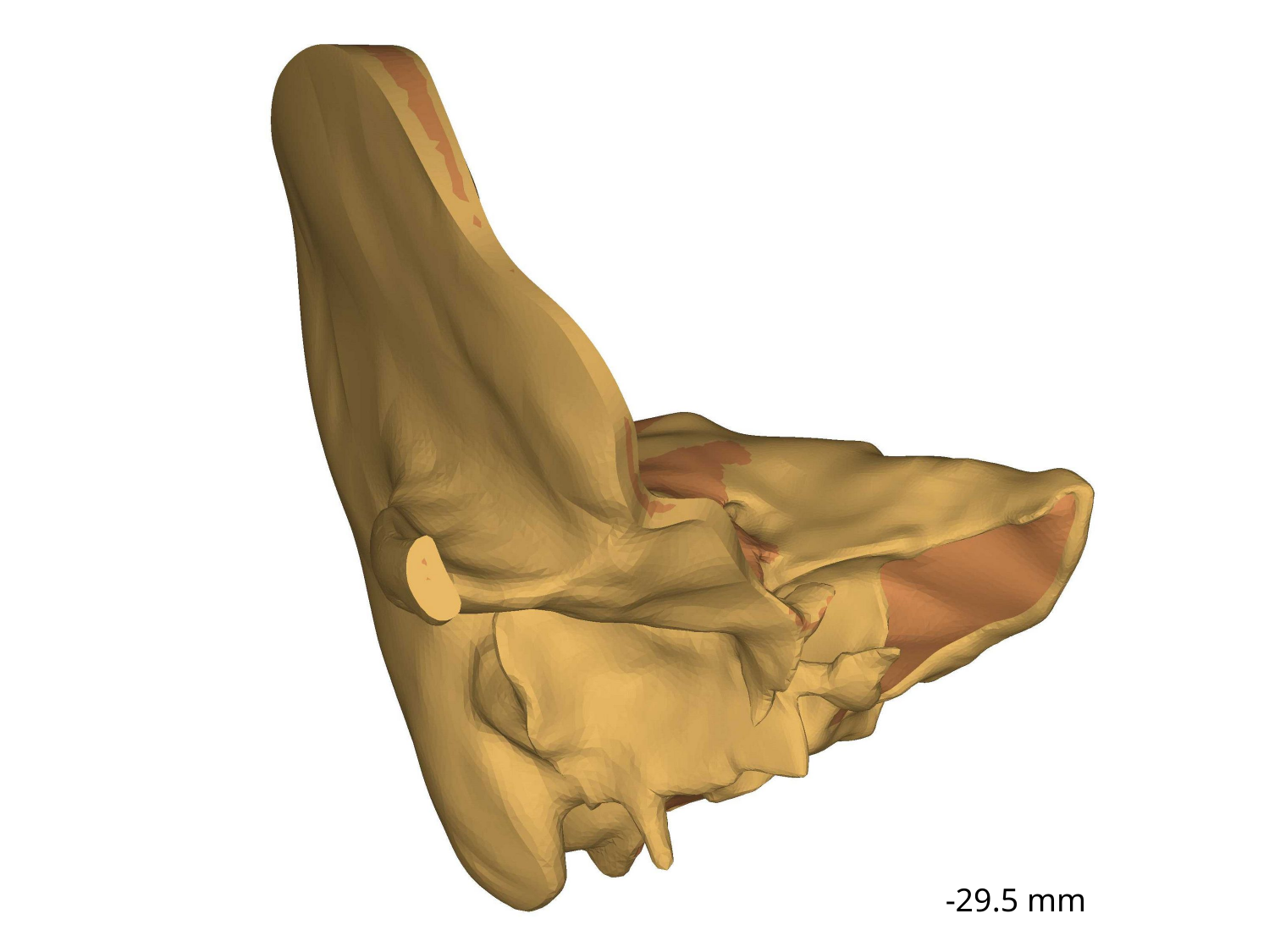

-29.5 mm

## Slide 20
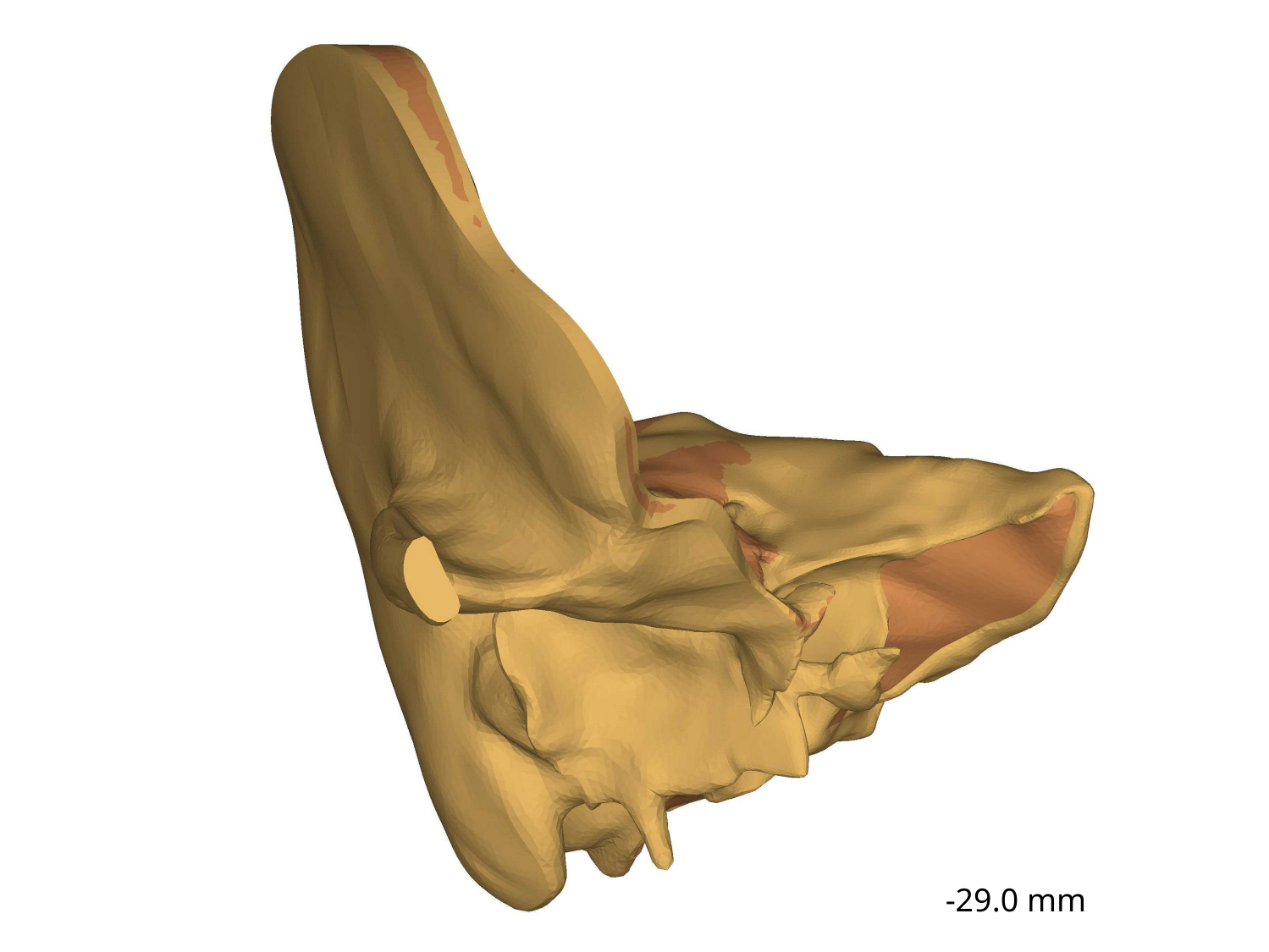

-29.0 mm

## Slide 21
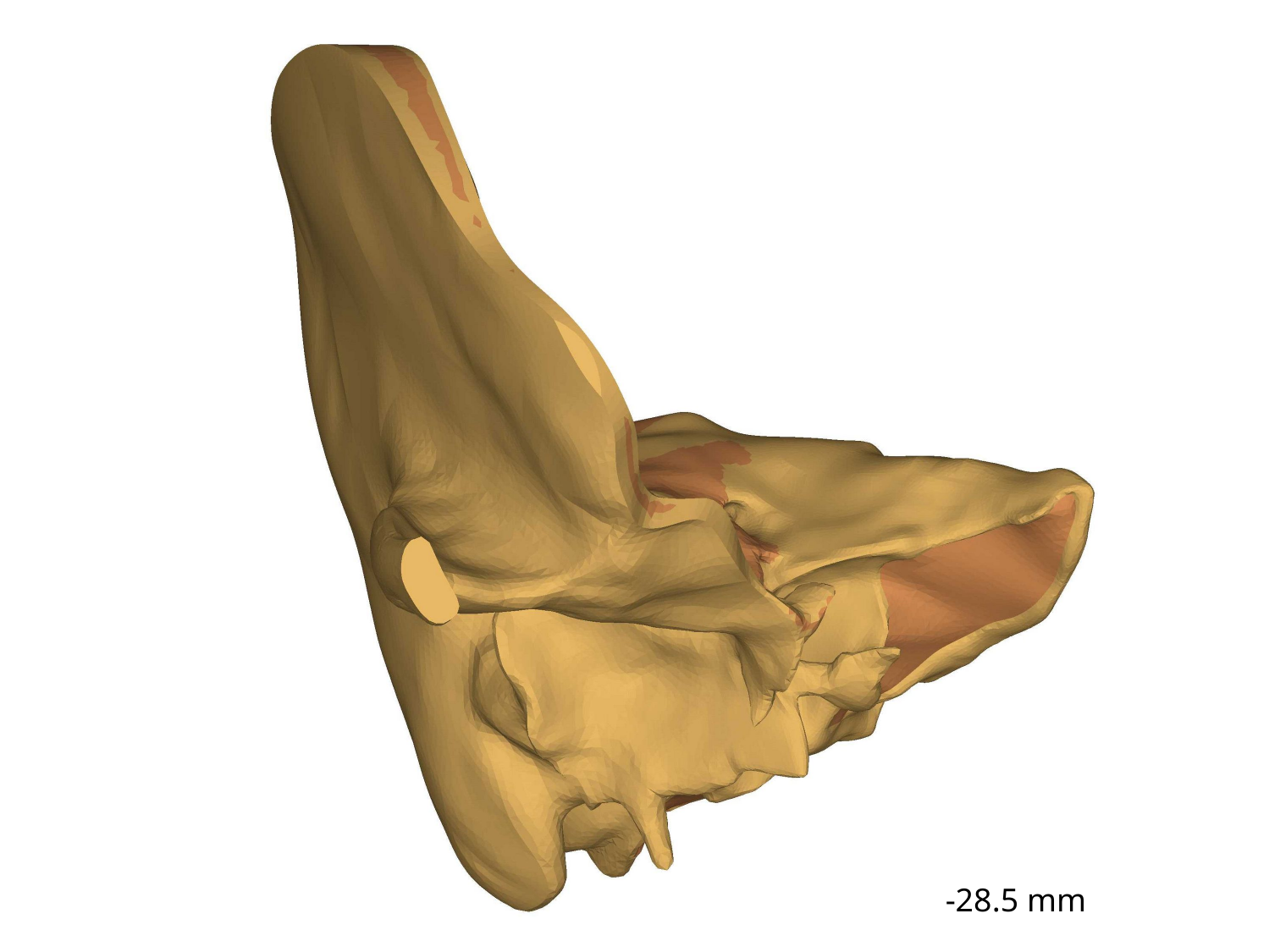

-28.5 mm

## Slide 22
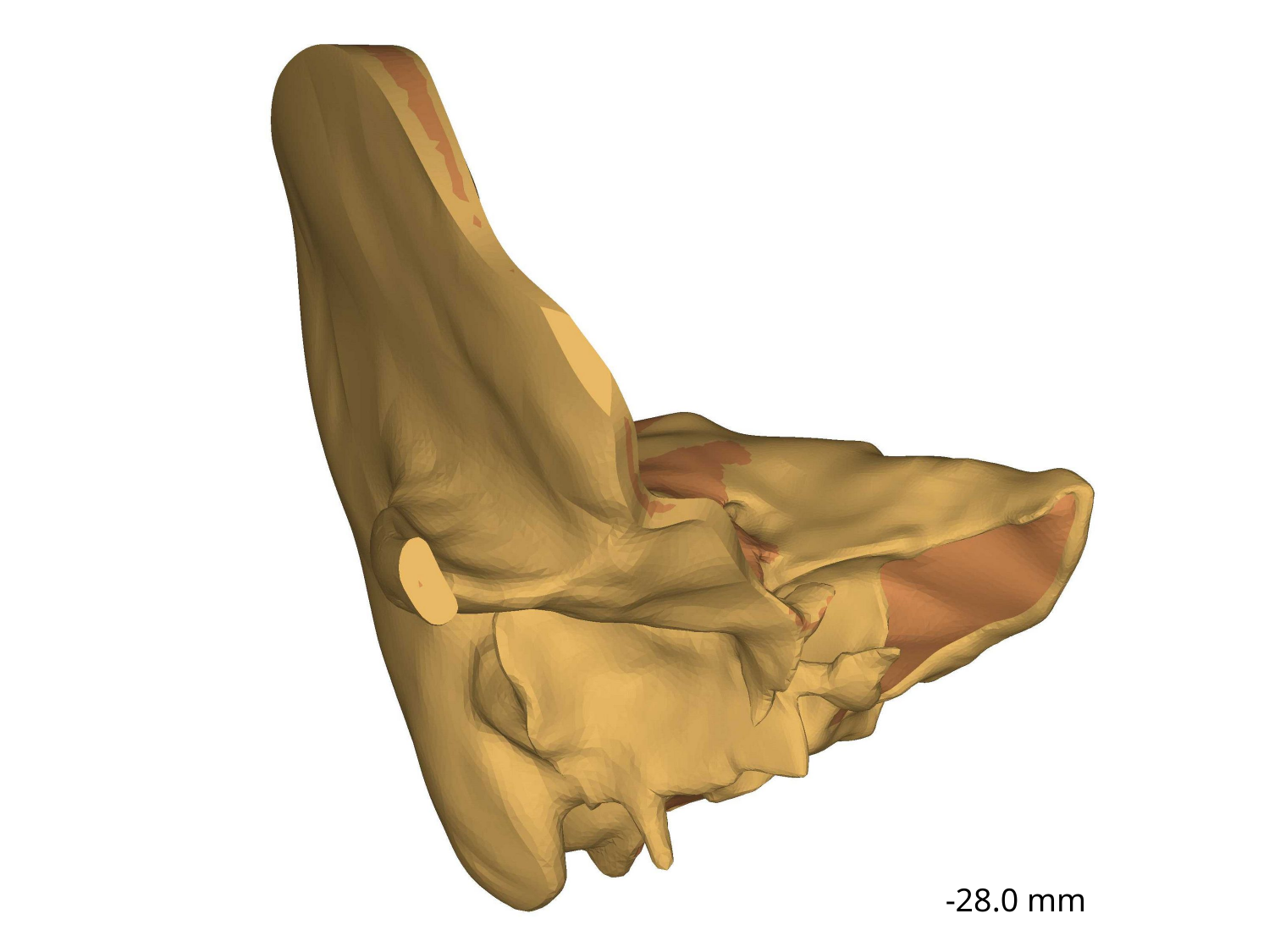

-28.0 mm

## Slide 23
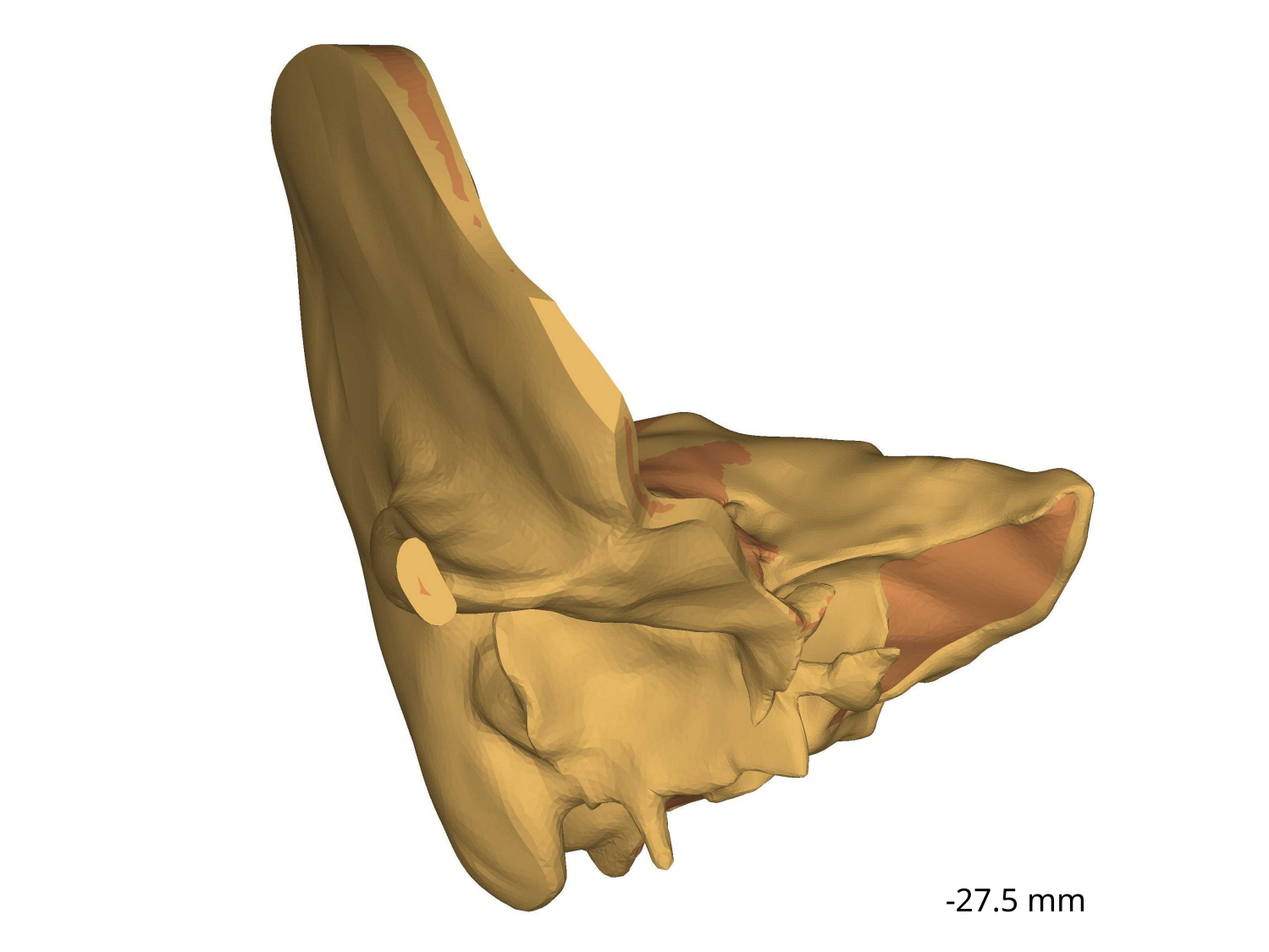

-27.5 mm

## Slide 24
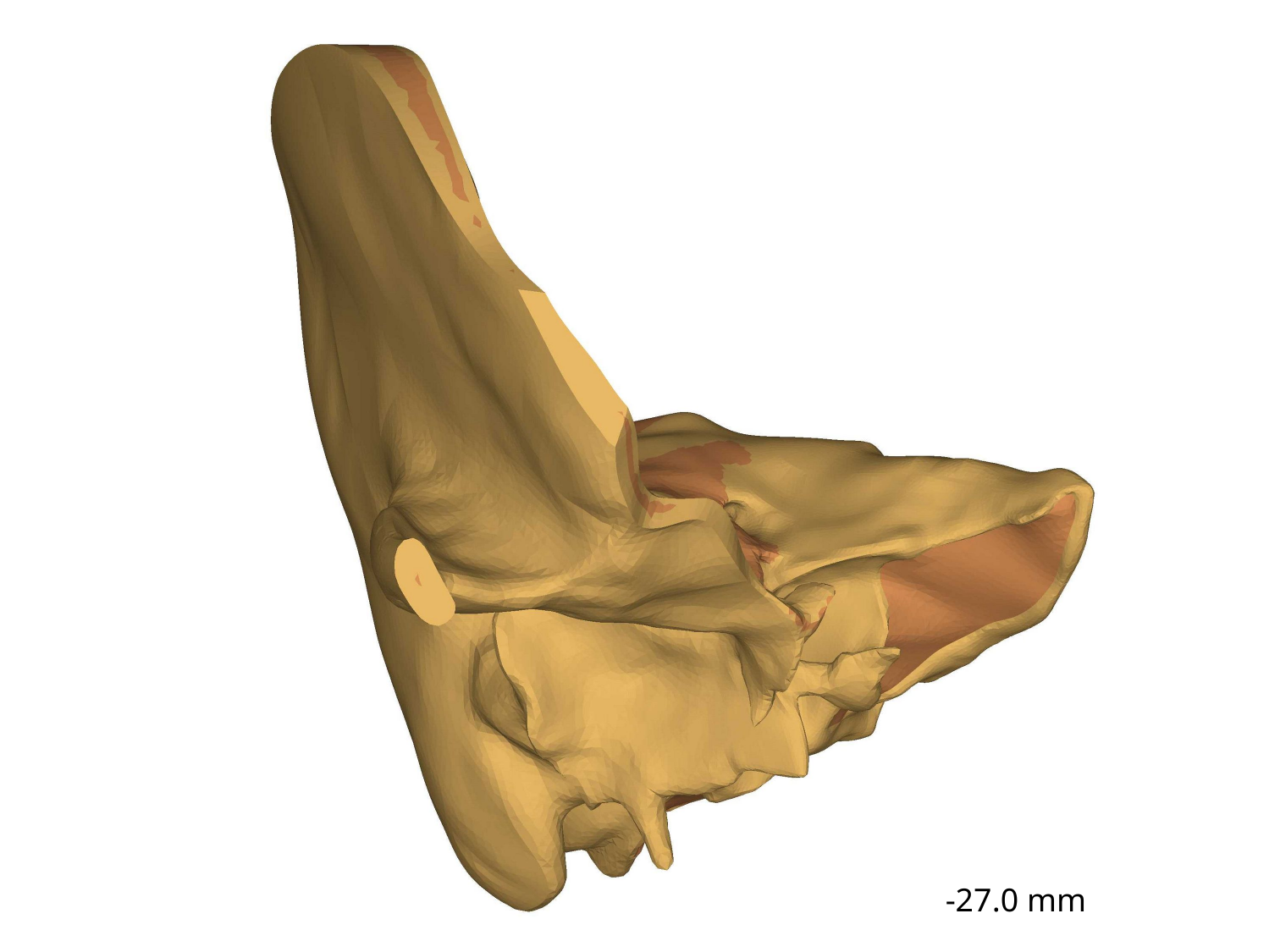

-27.0 mm

## Slide 25
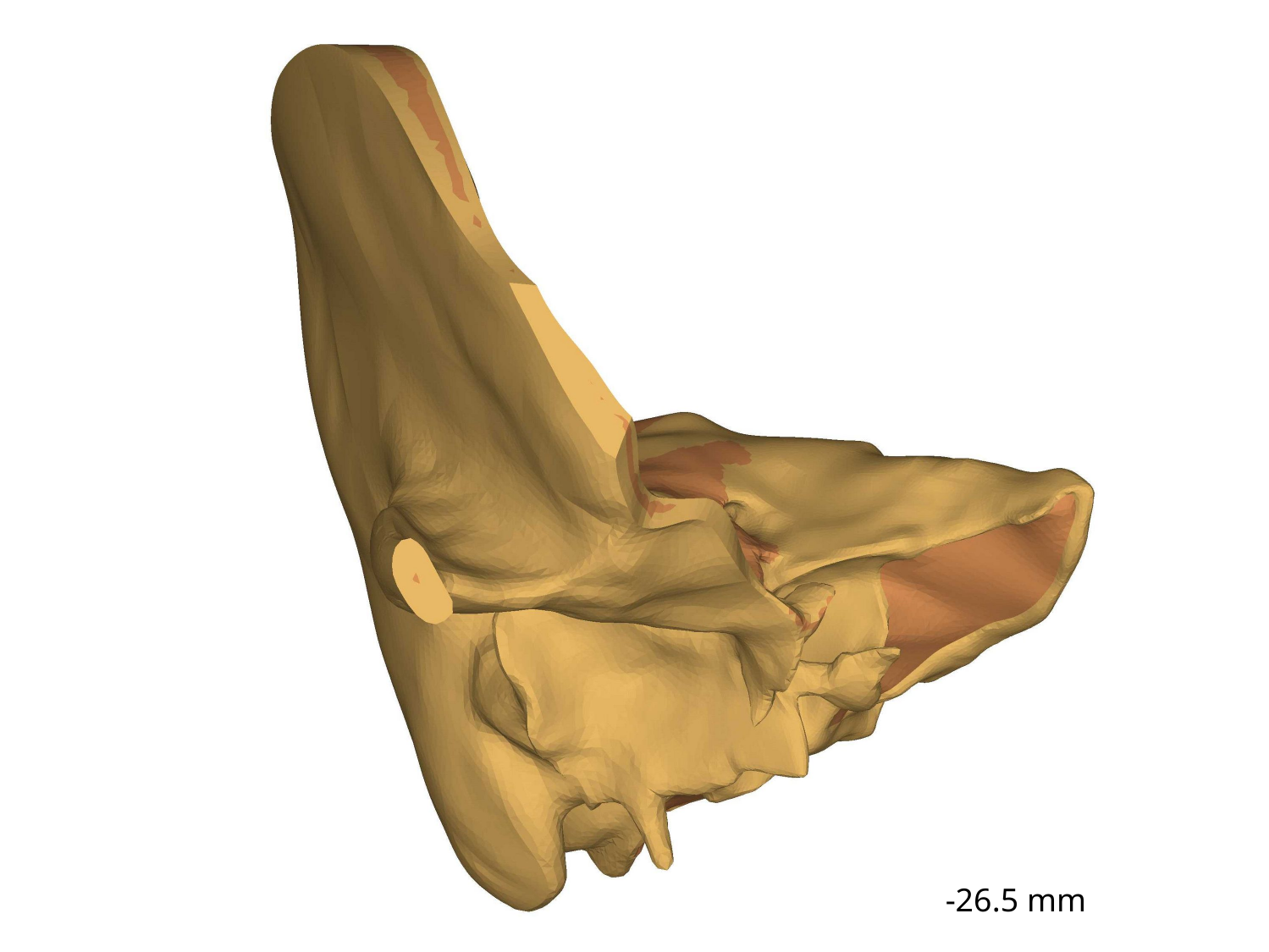

-26.5 mm

## Slide 26
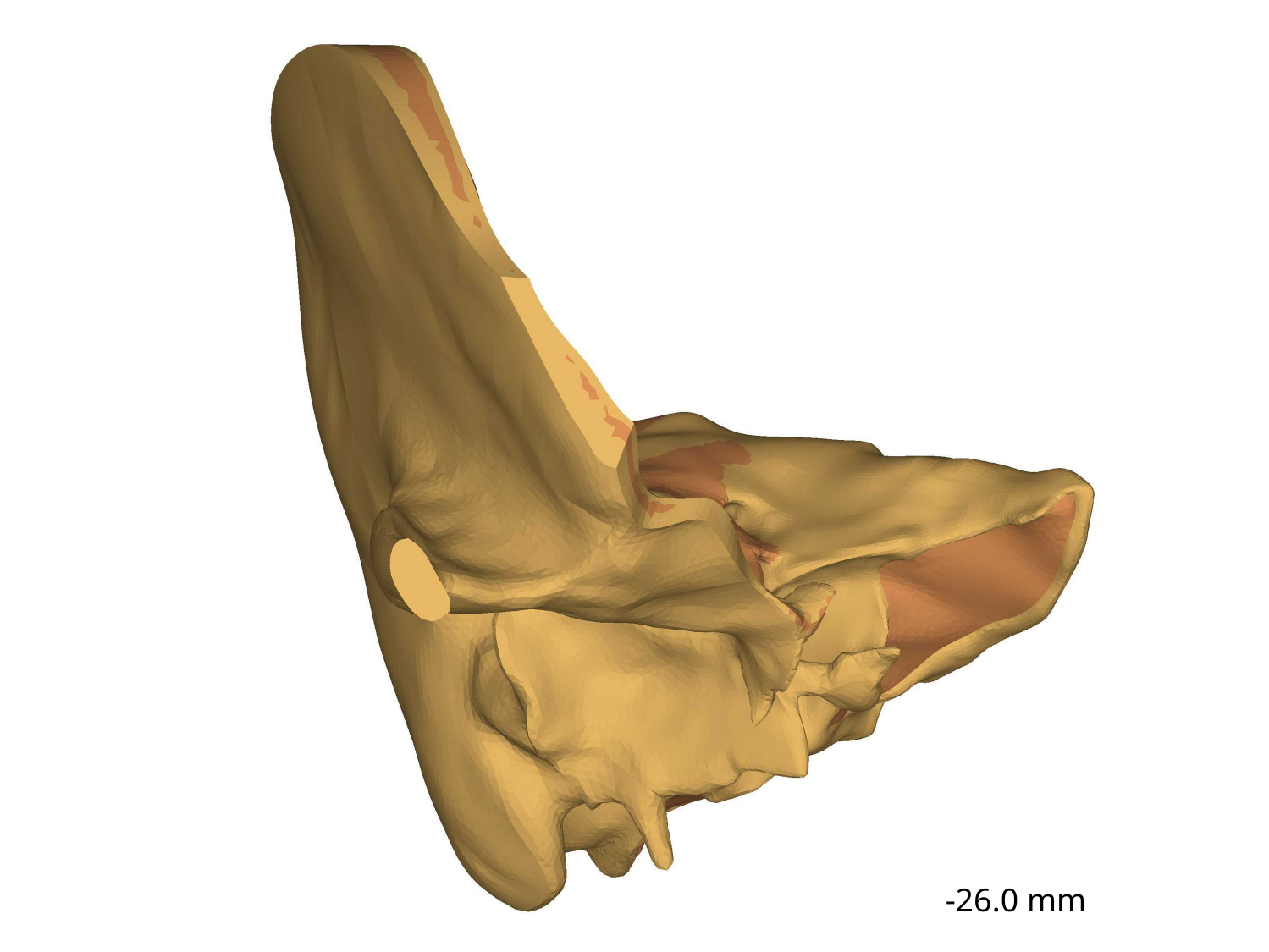

-26.0 mm

## Slide 27
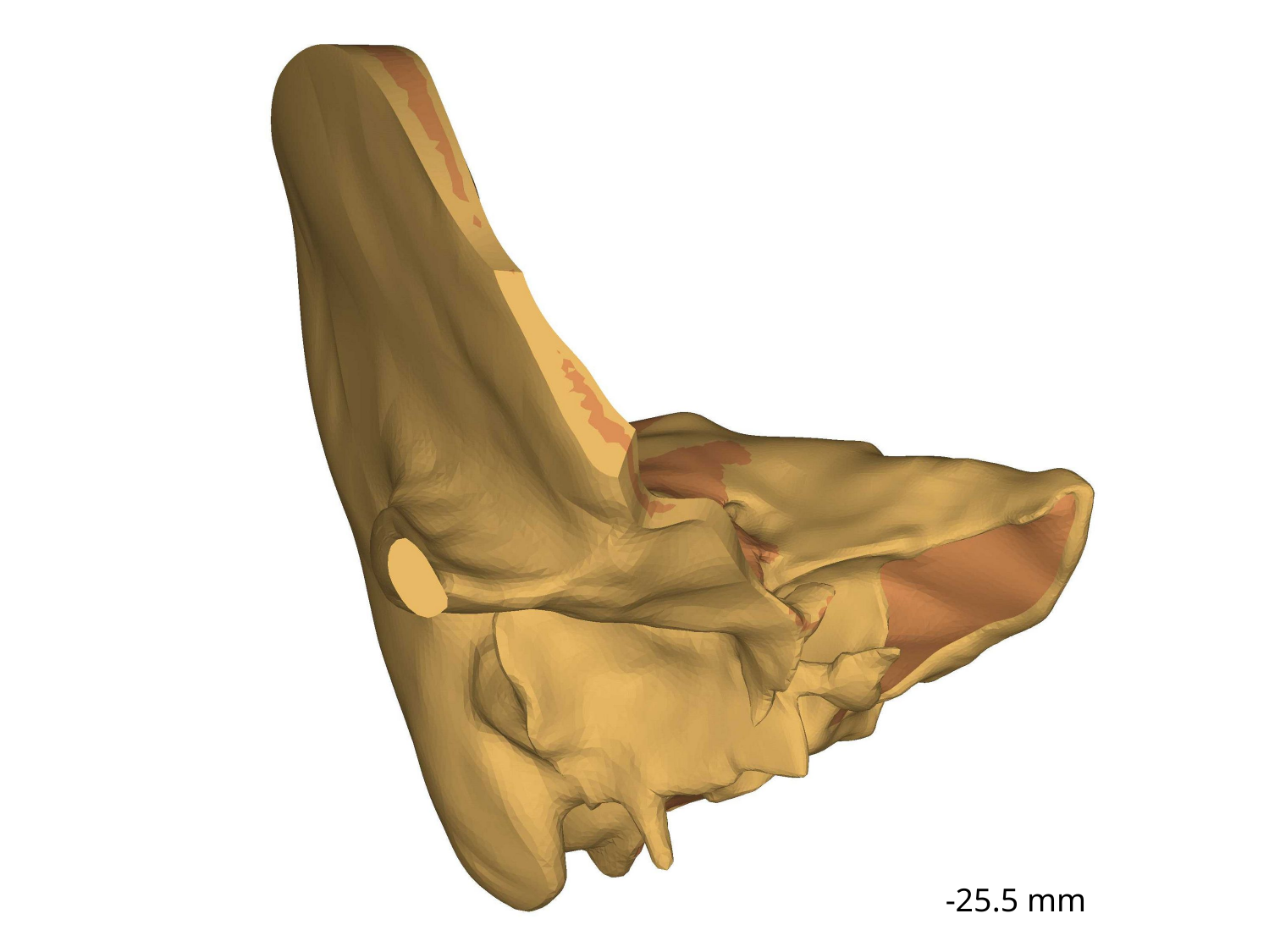

-25.5 mm

## Slide 28
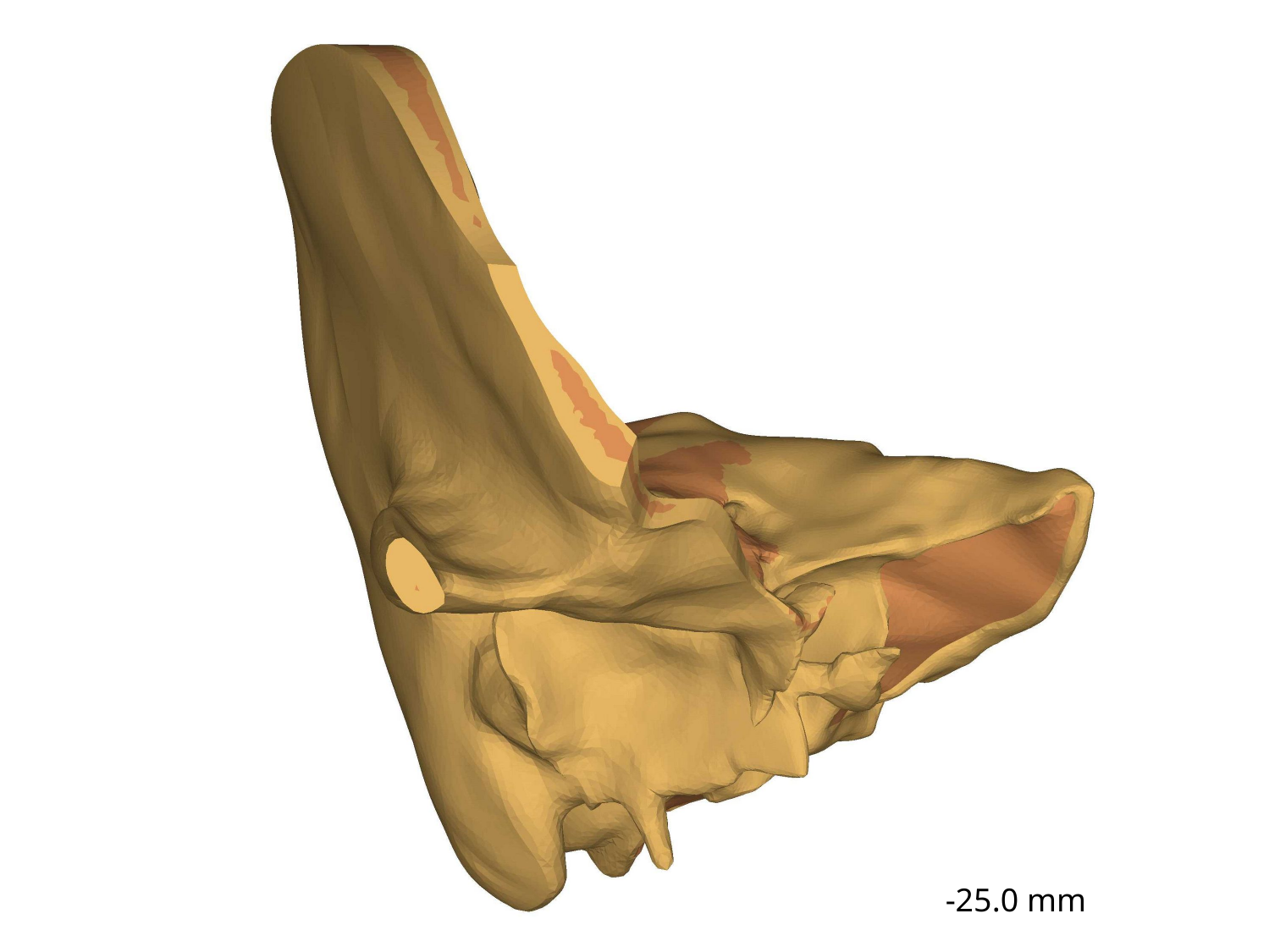

-25.0 mm

## Slide 29
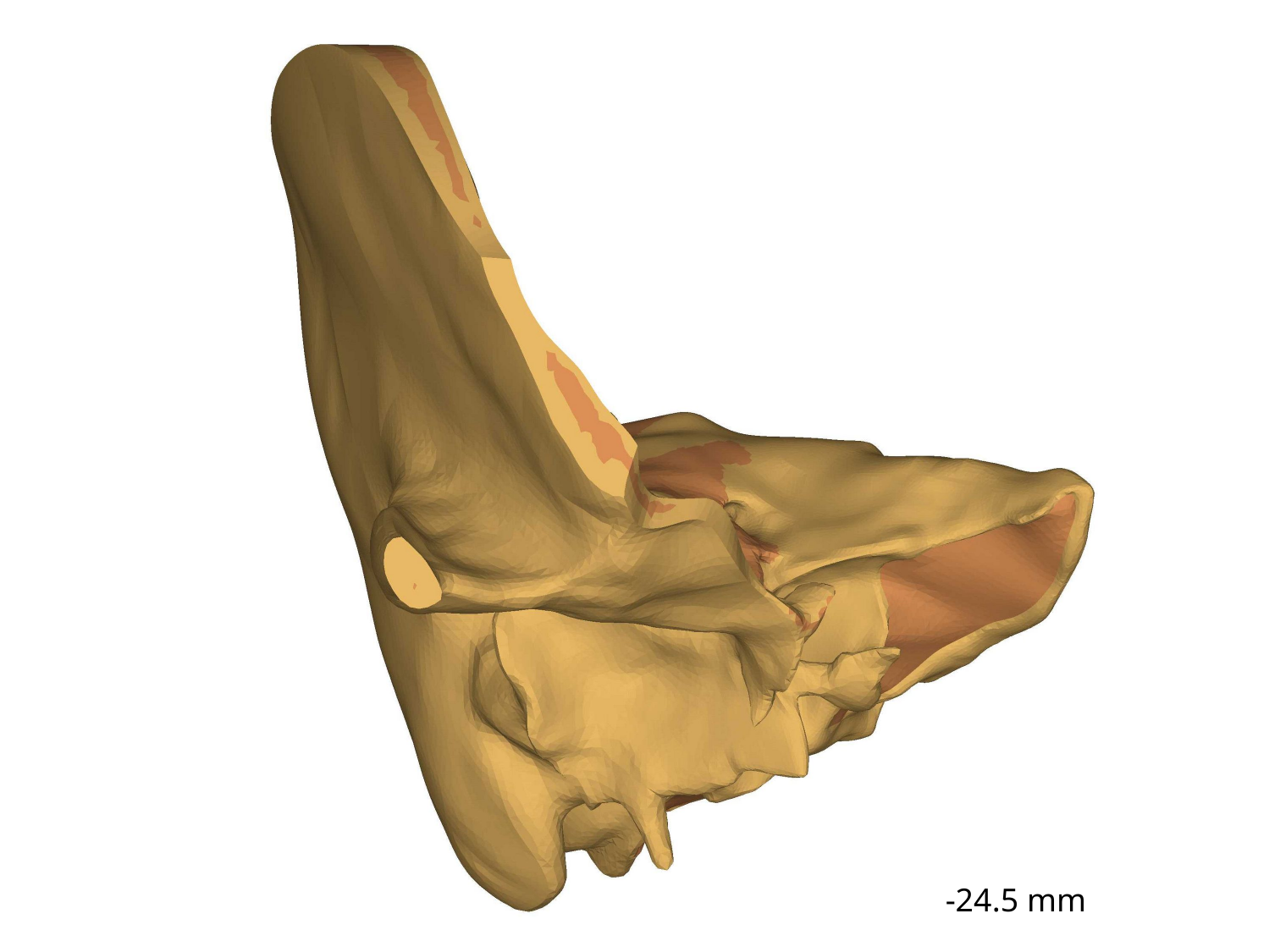

-24.5 mm

## Slide 30
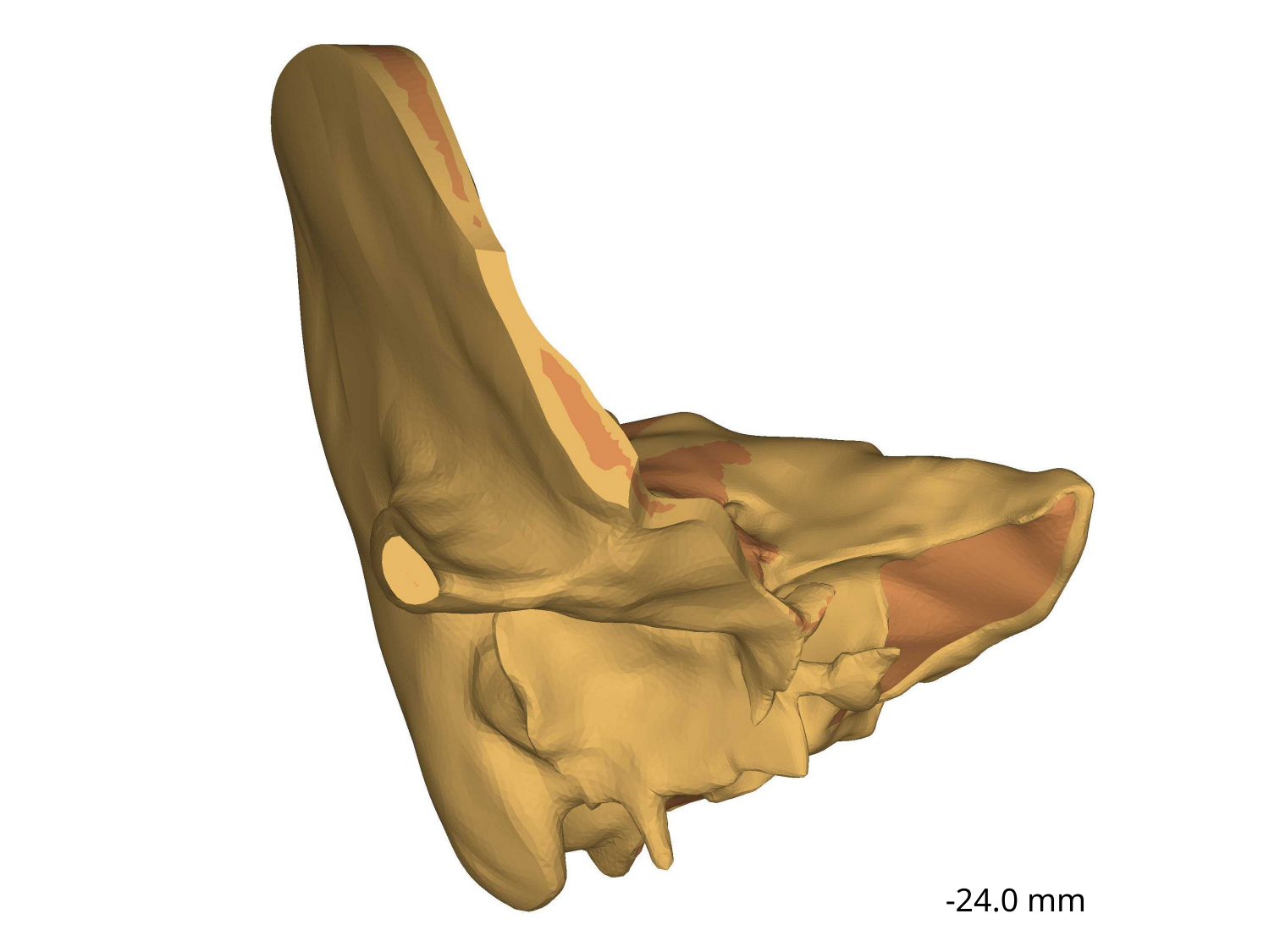

-24.0 mm

## Slide 31
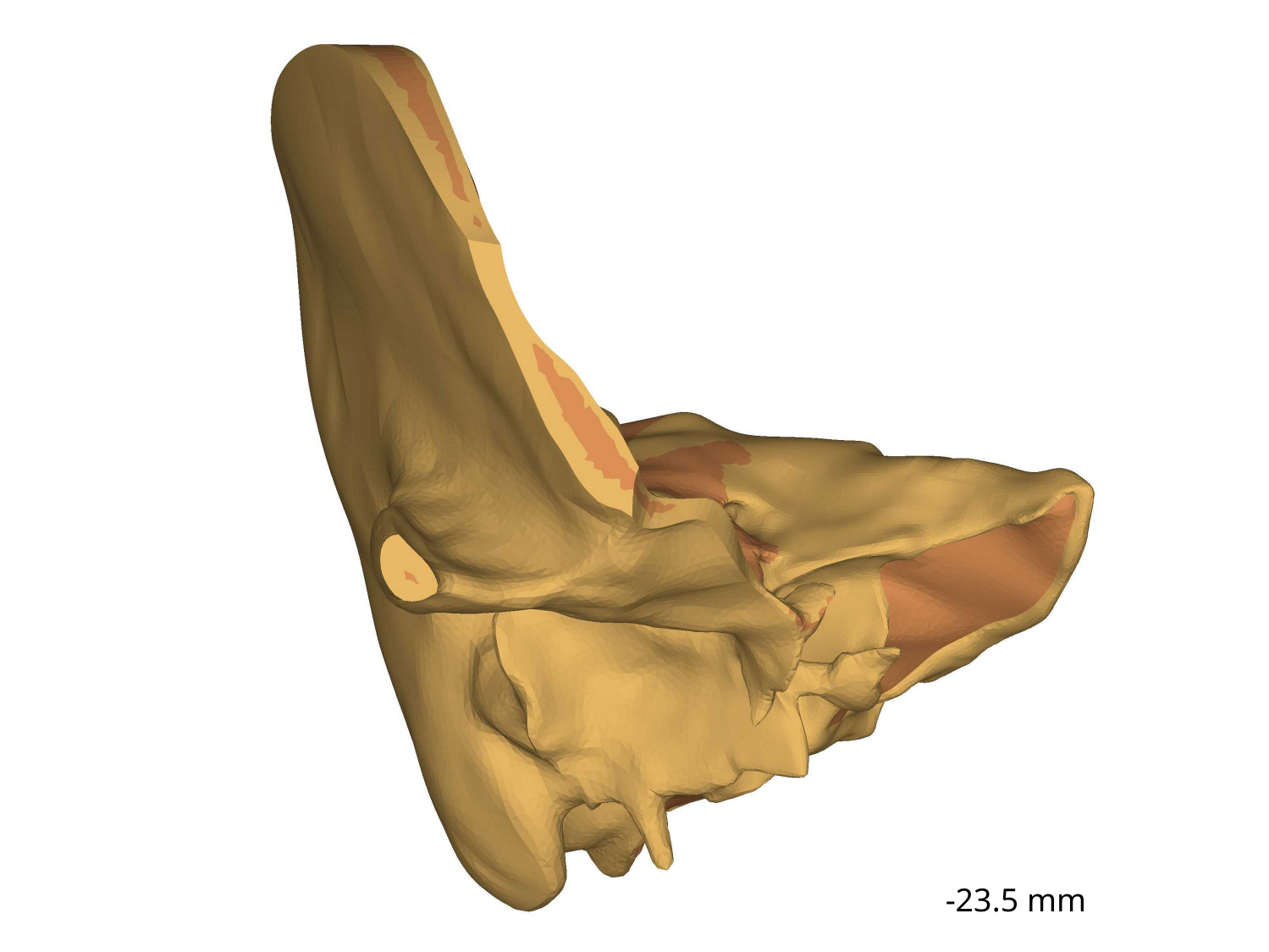

-23.5 mm

## Slide 32
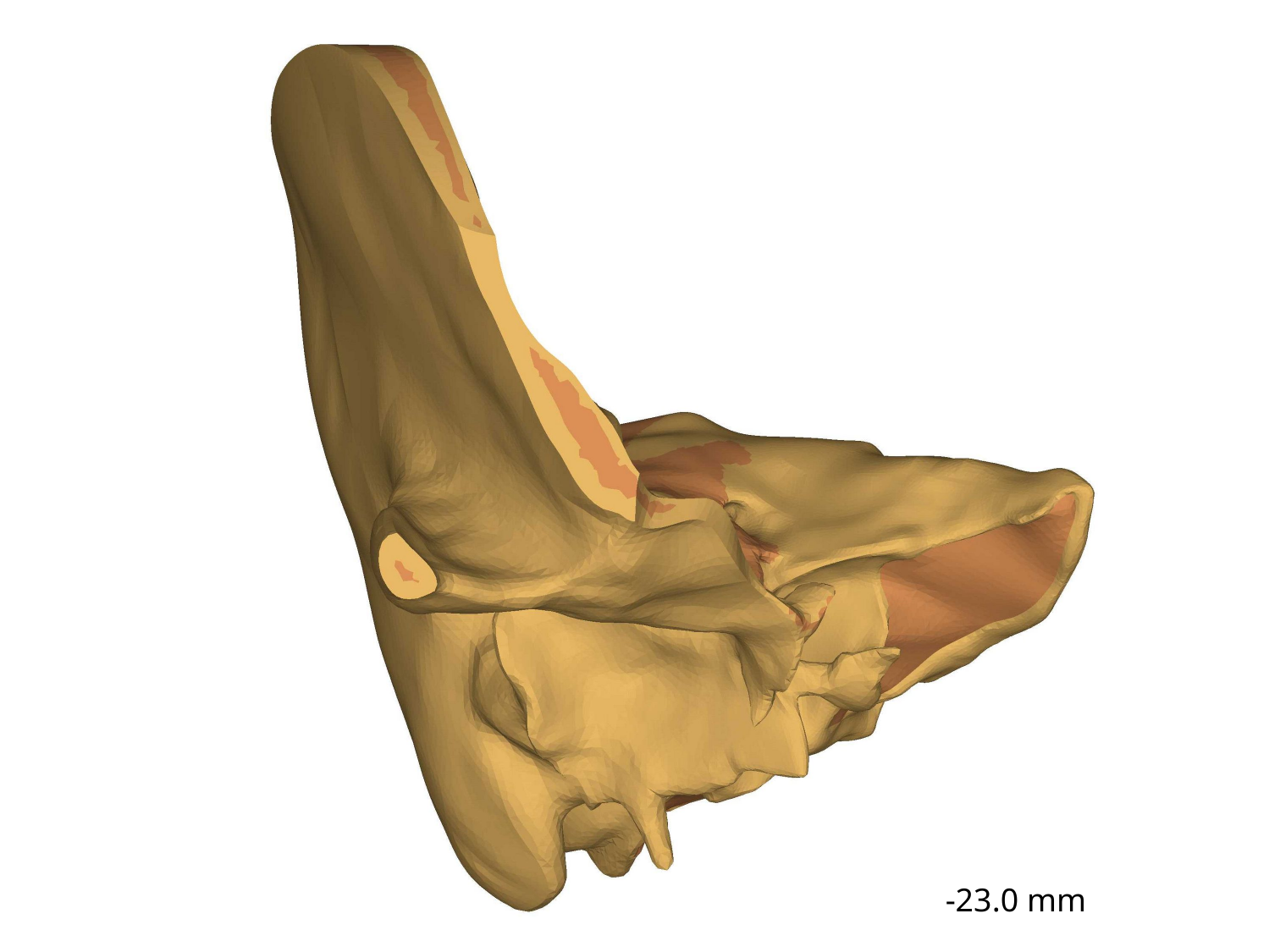

-23.0 mm

## Slide 33
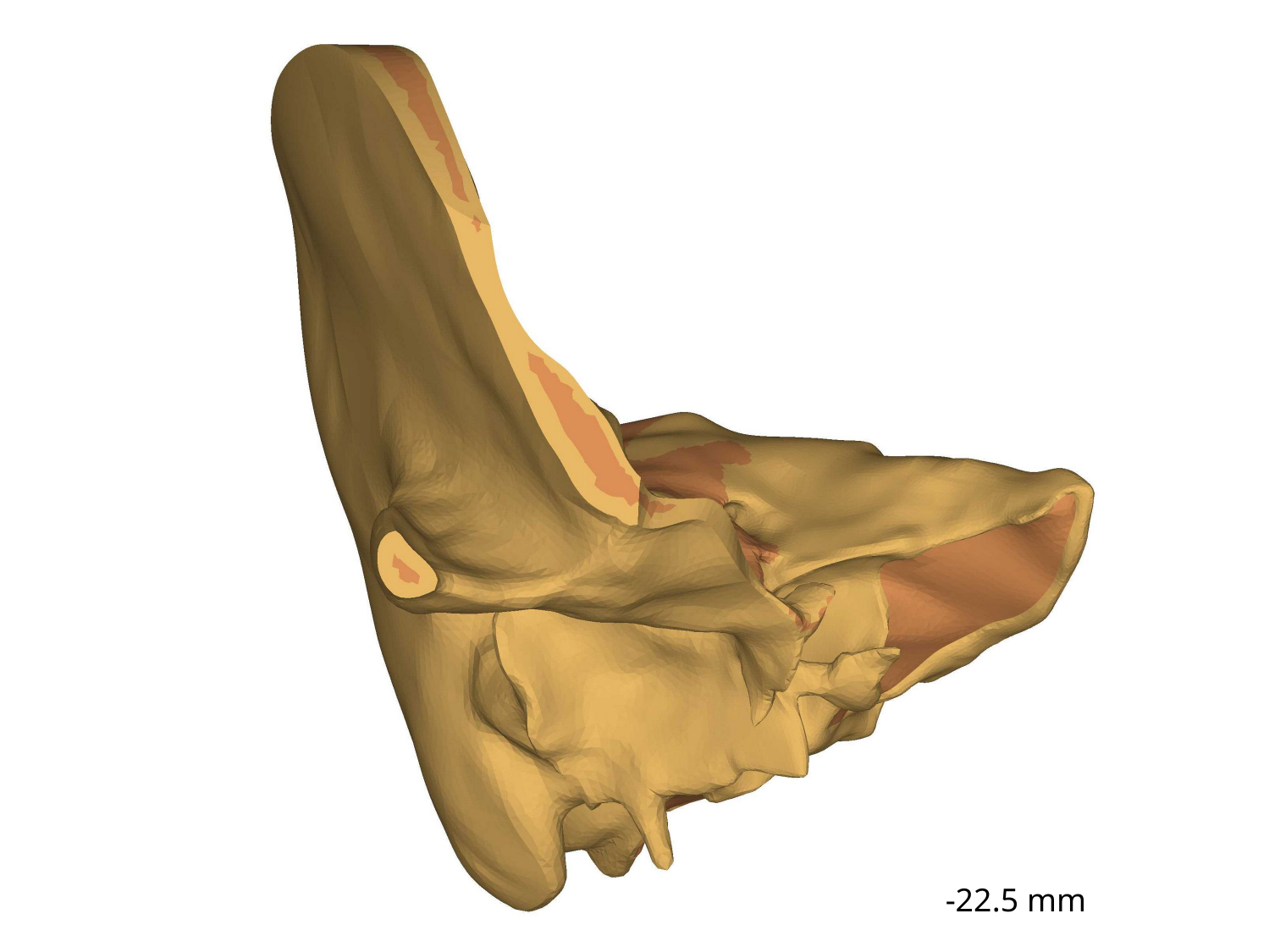

-22.5 mm

## Slide 34
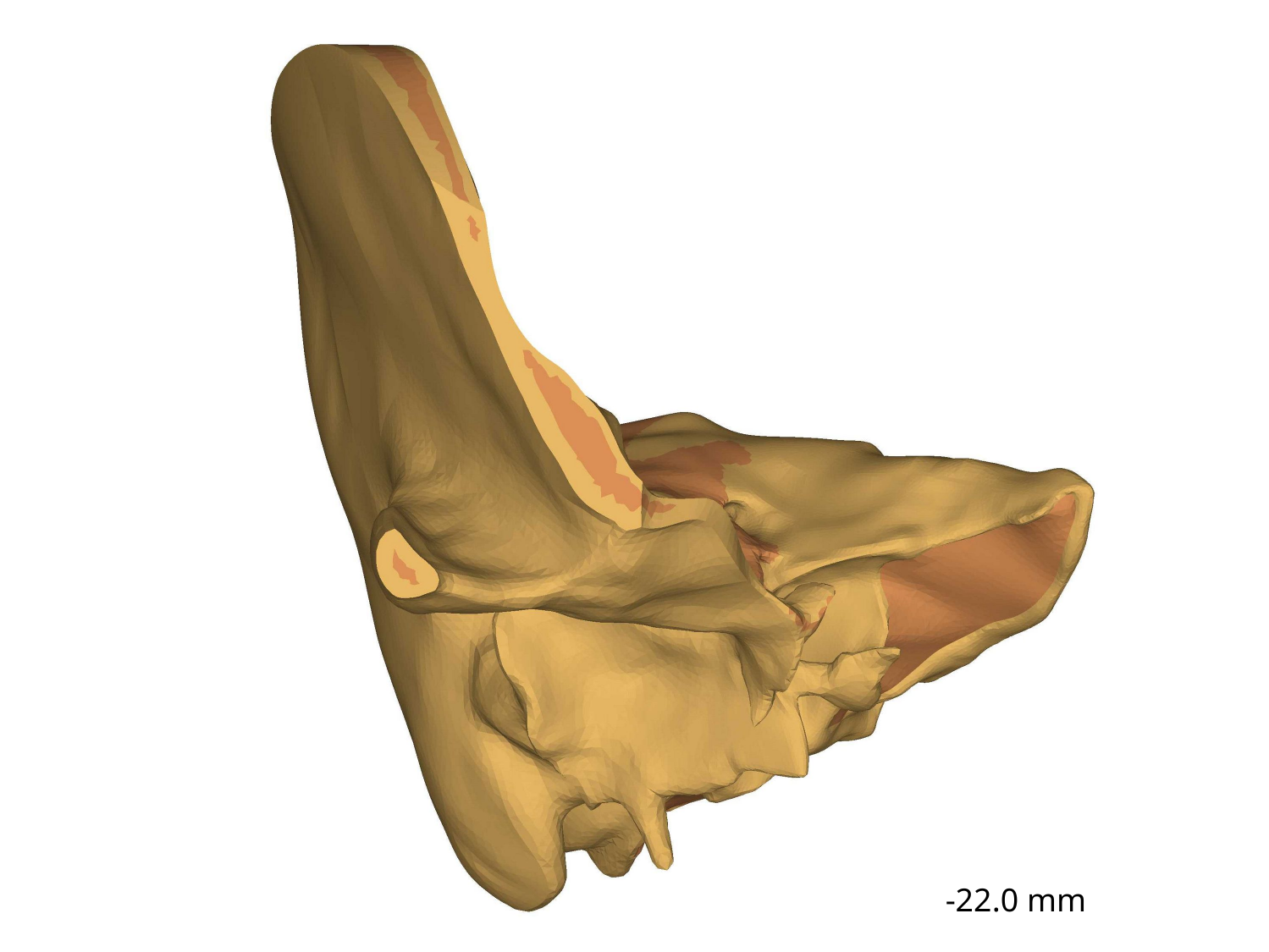

-22.0 mm

## Slide 35
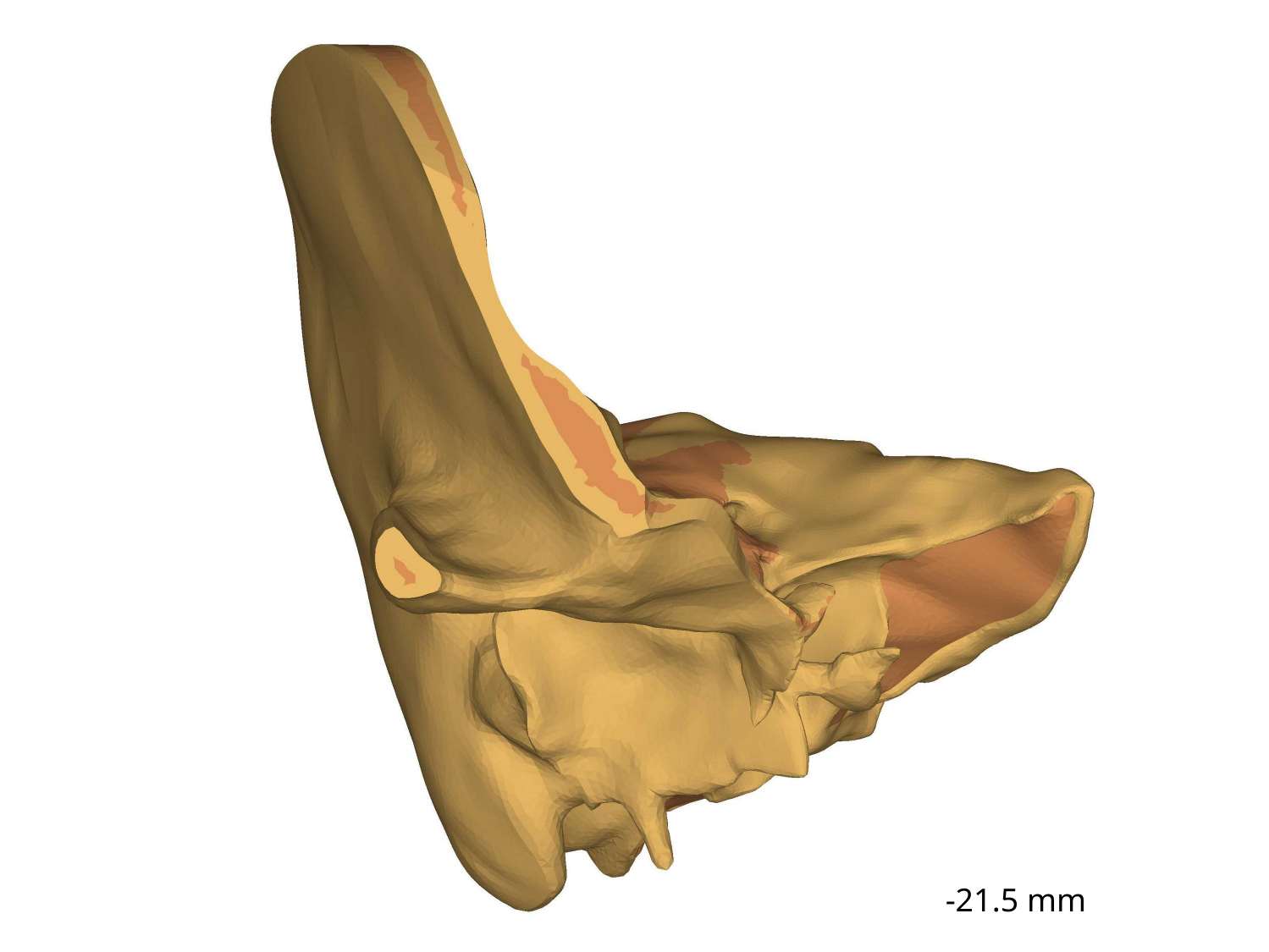

-21.5 mm

## Slide 36
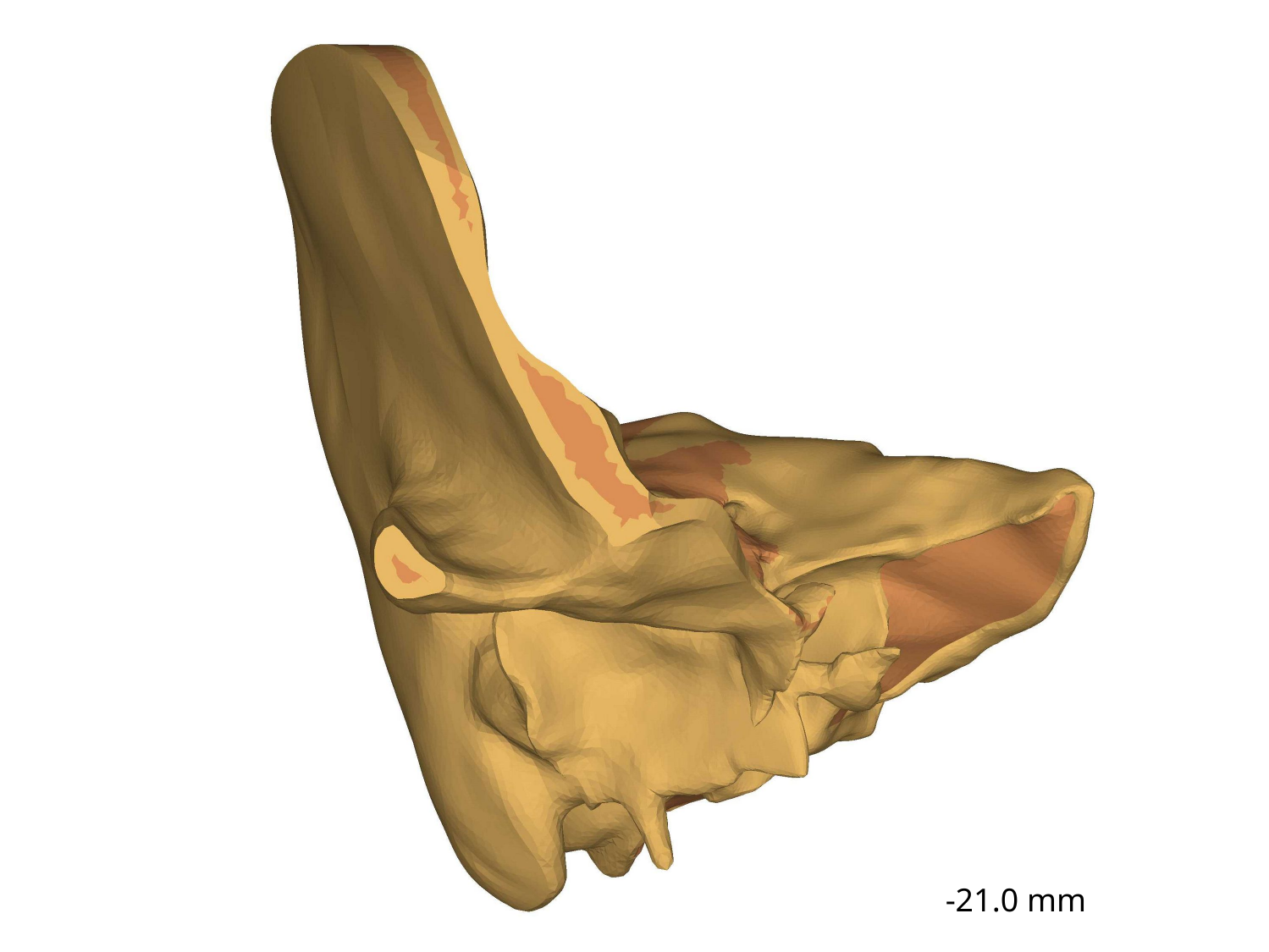

-21.0 mm

## Slide 37
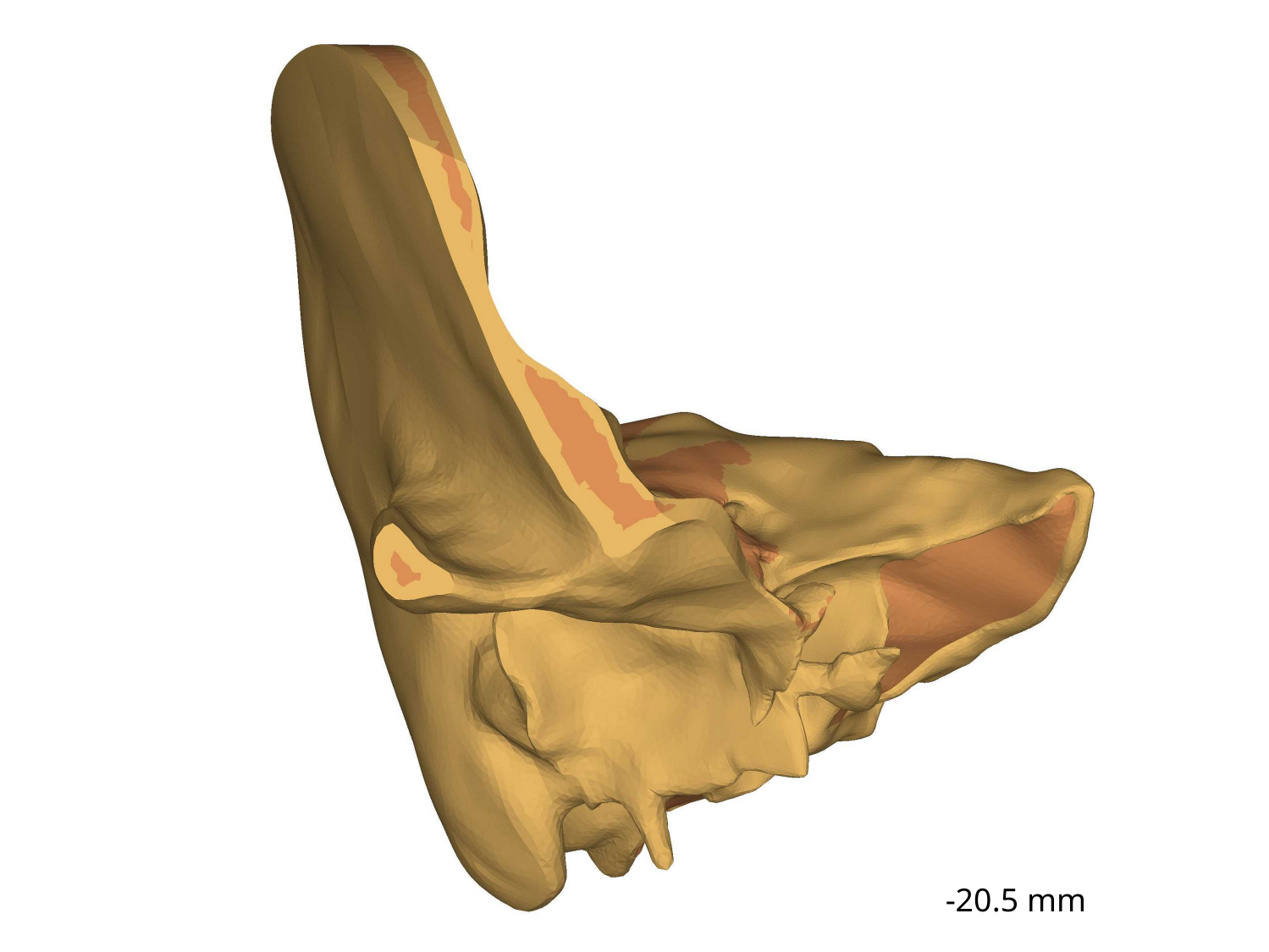

-20.5 mm

## Slide 38
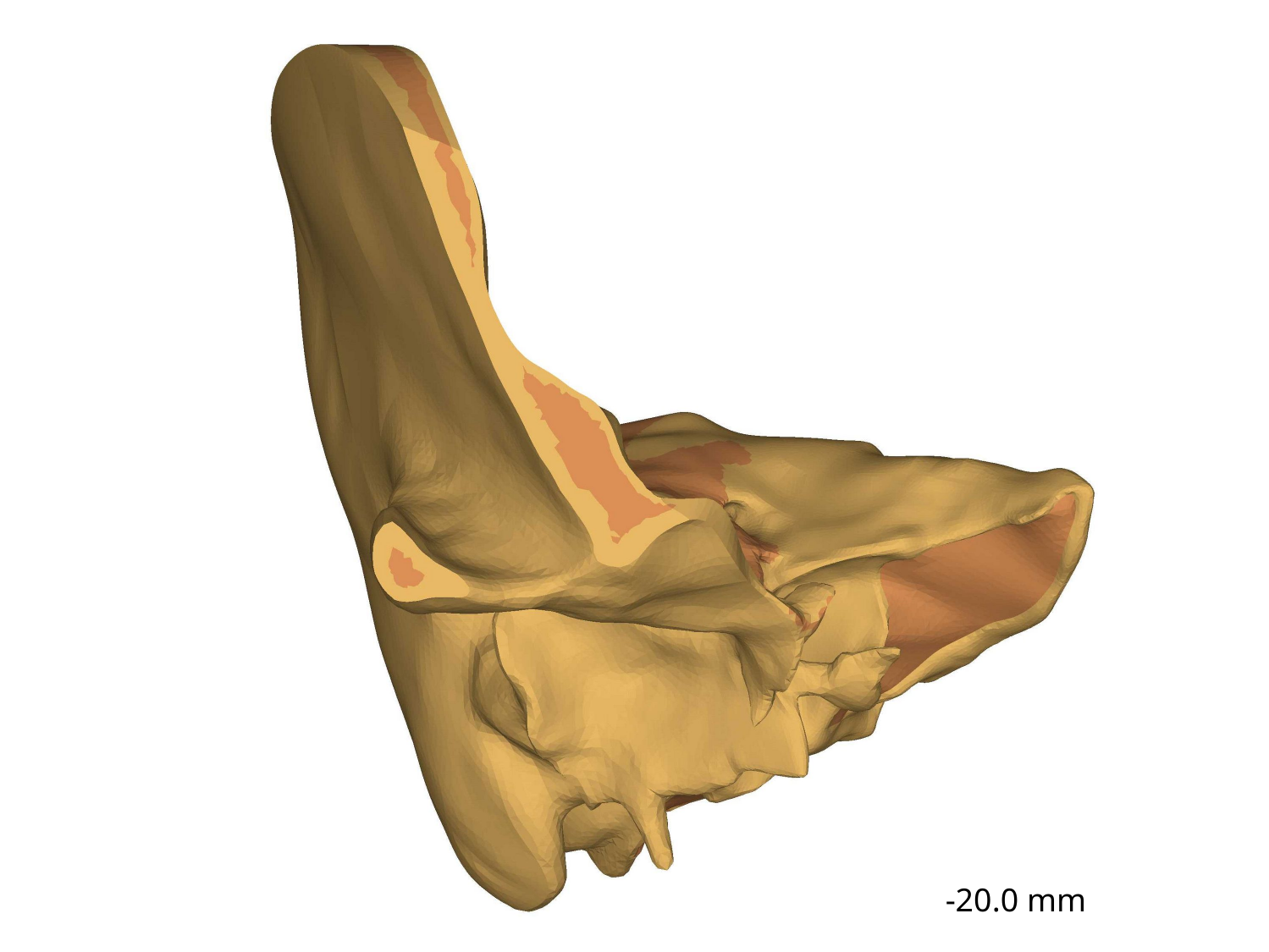

-20.0 mm

## Slide 39
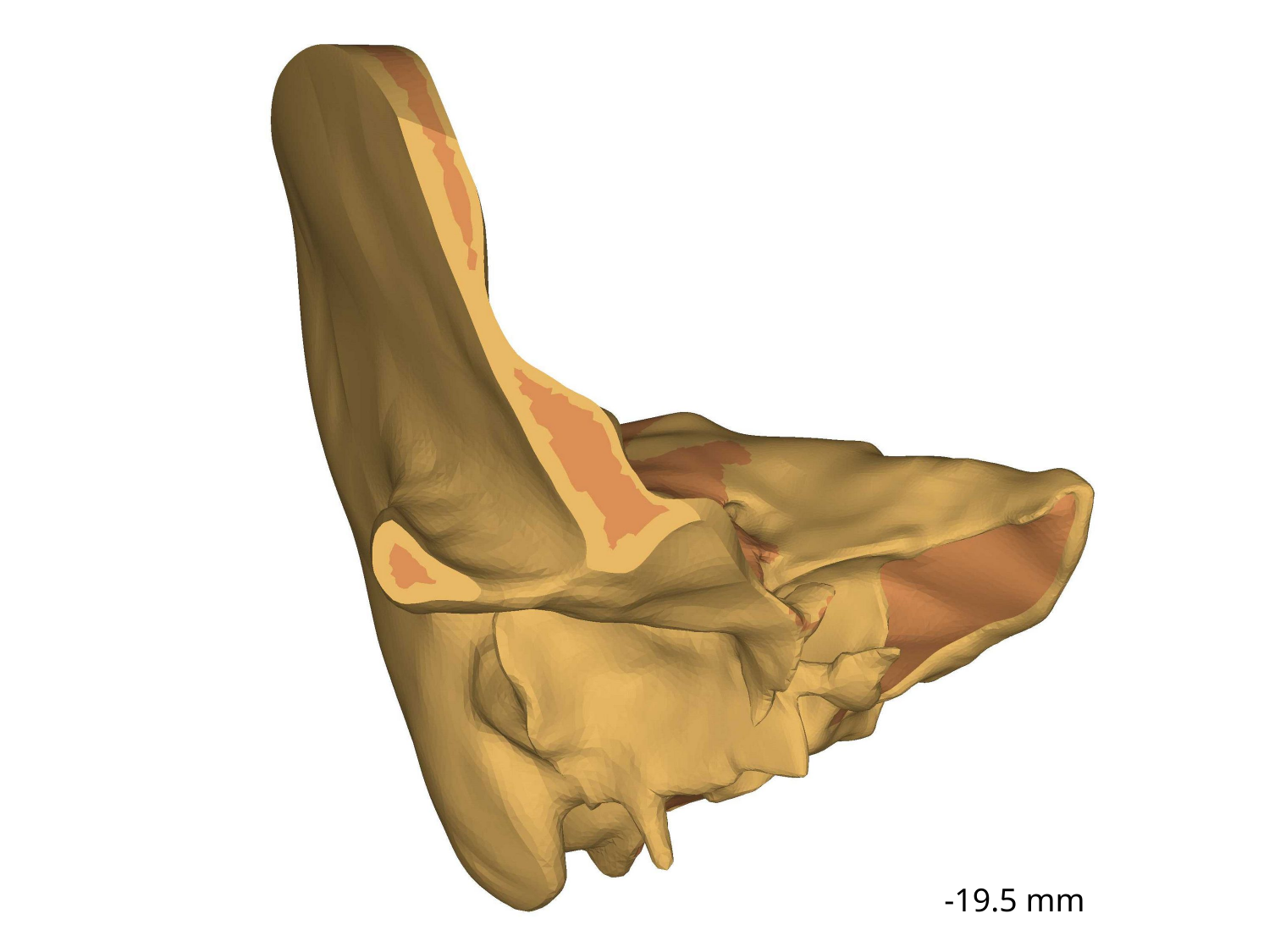

-19.5 mm

## Slide 40
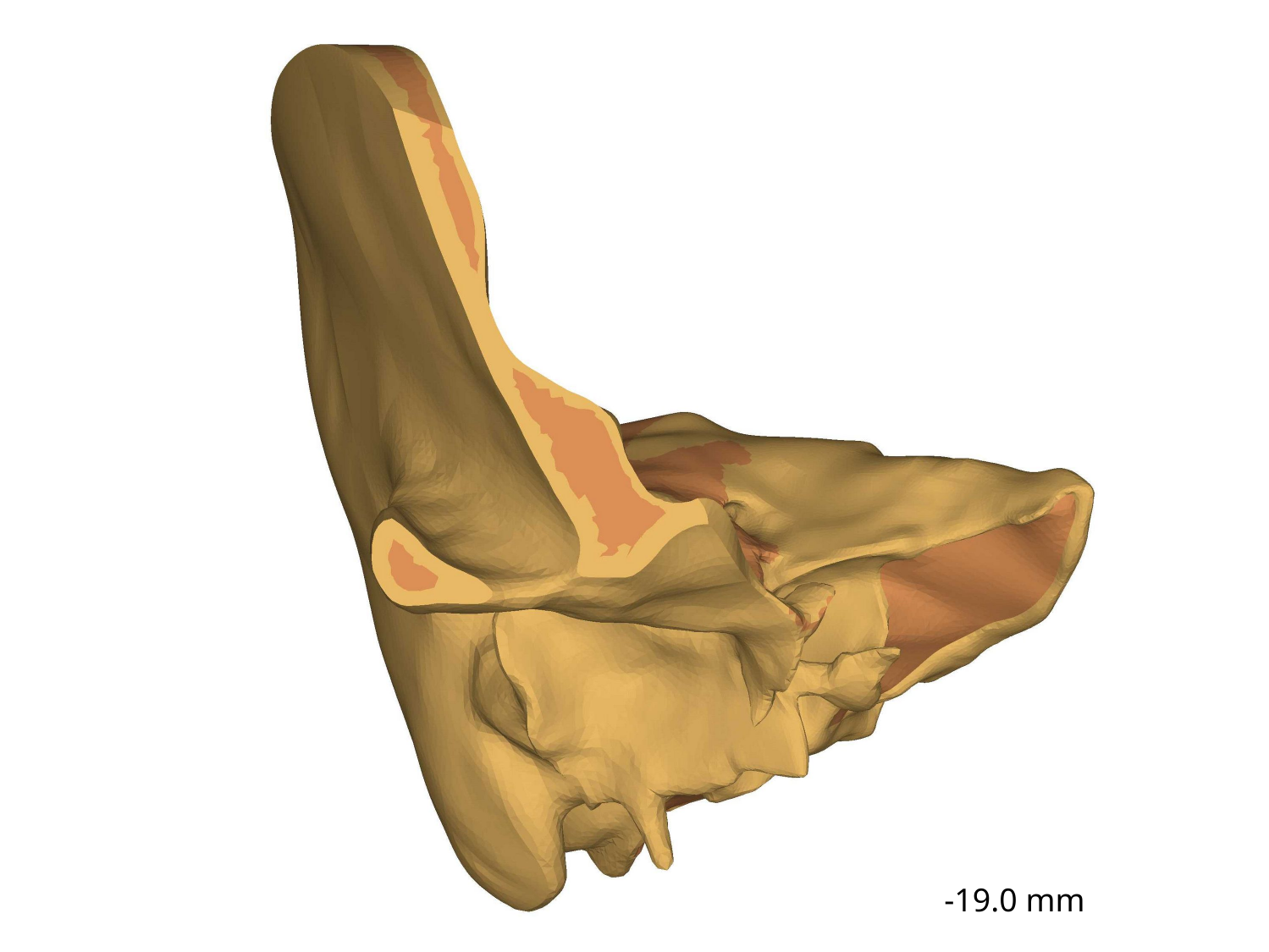

-19.0 mm

## Slide 41
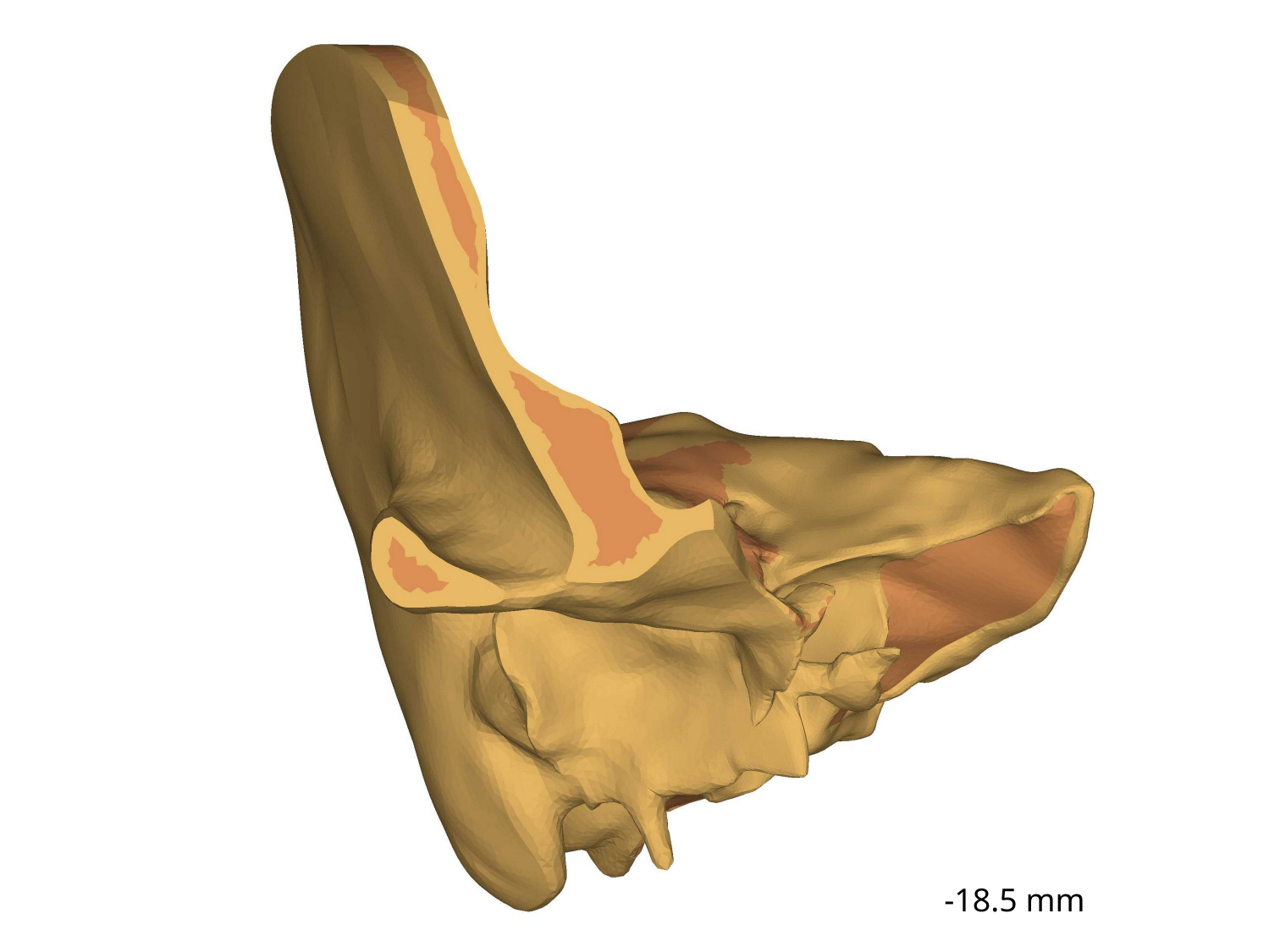

-18.5 mm

## Slide 42
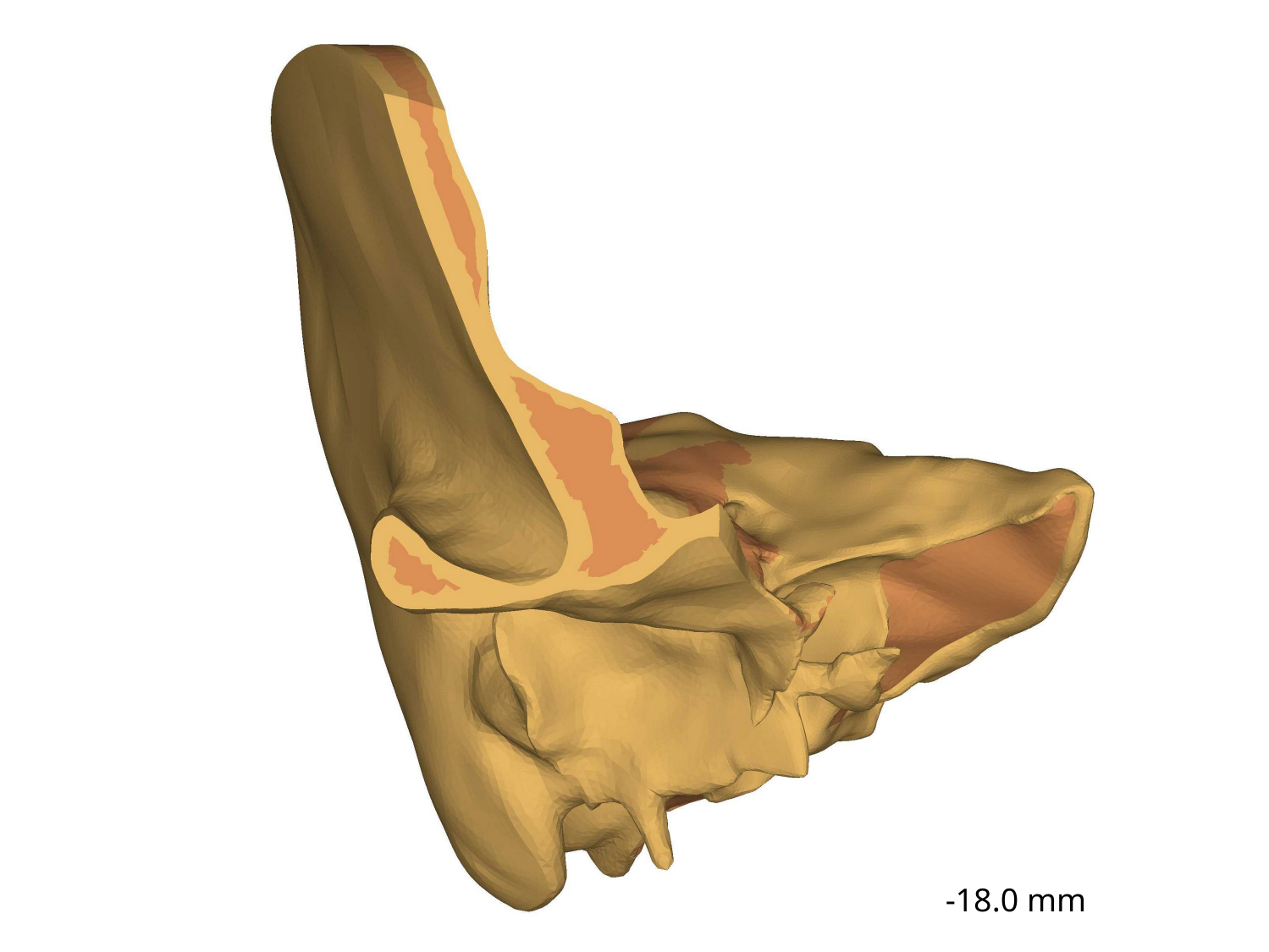

-18.0 mm

## Slide 43
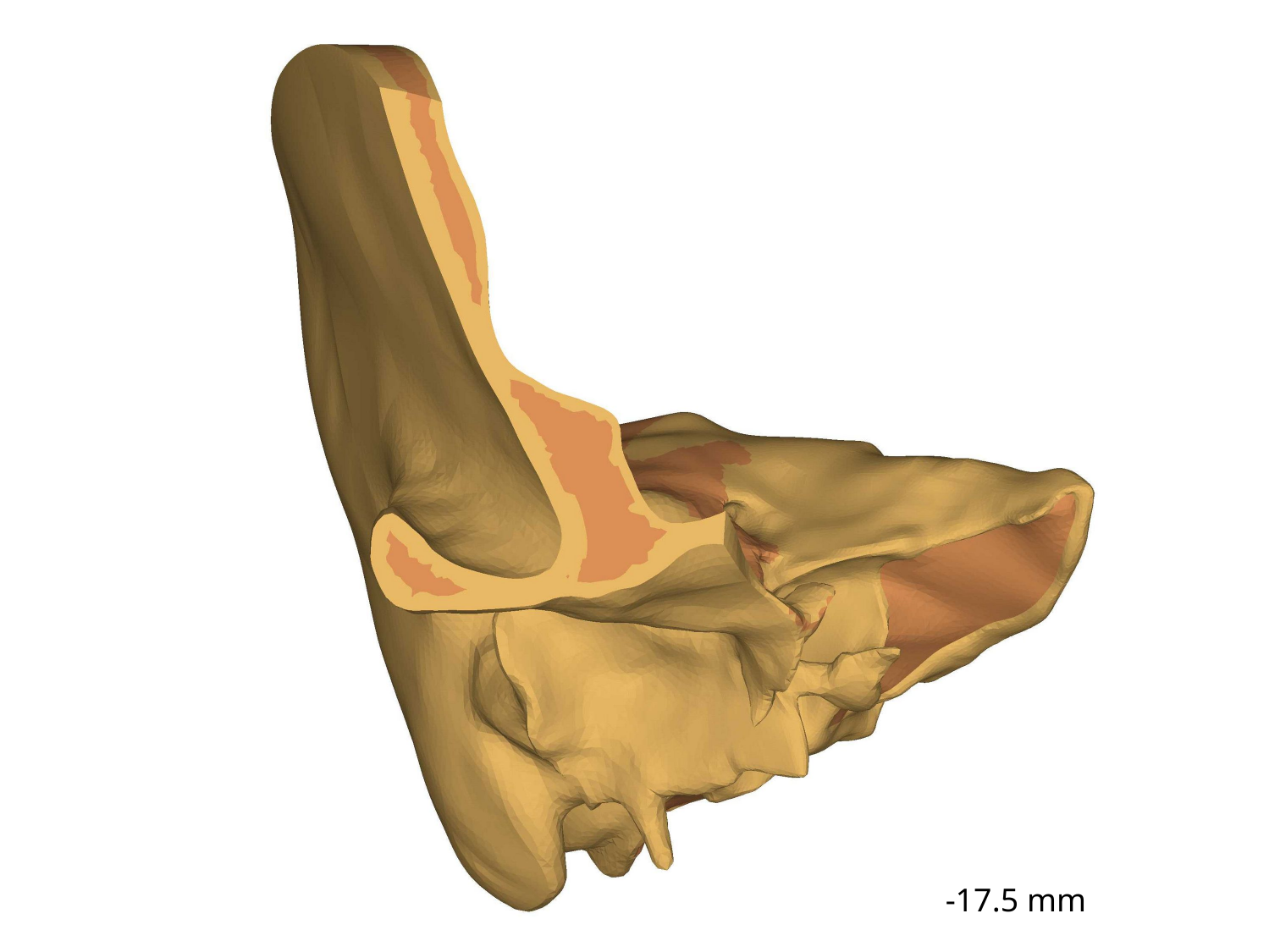

-17.5 mm

## Slide 44
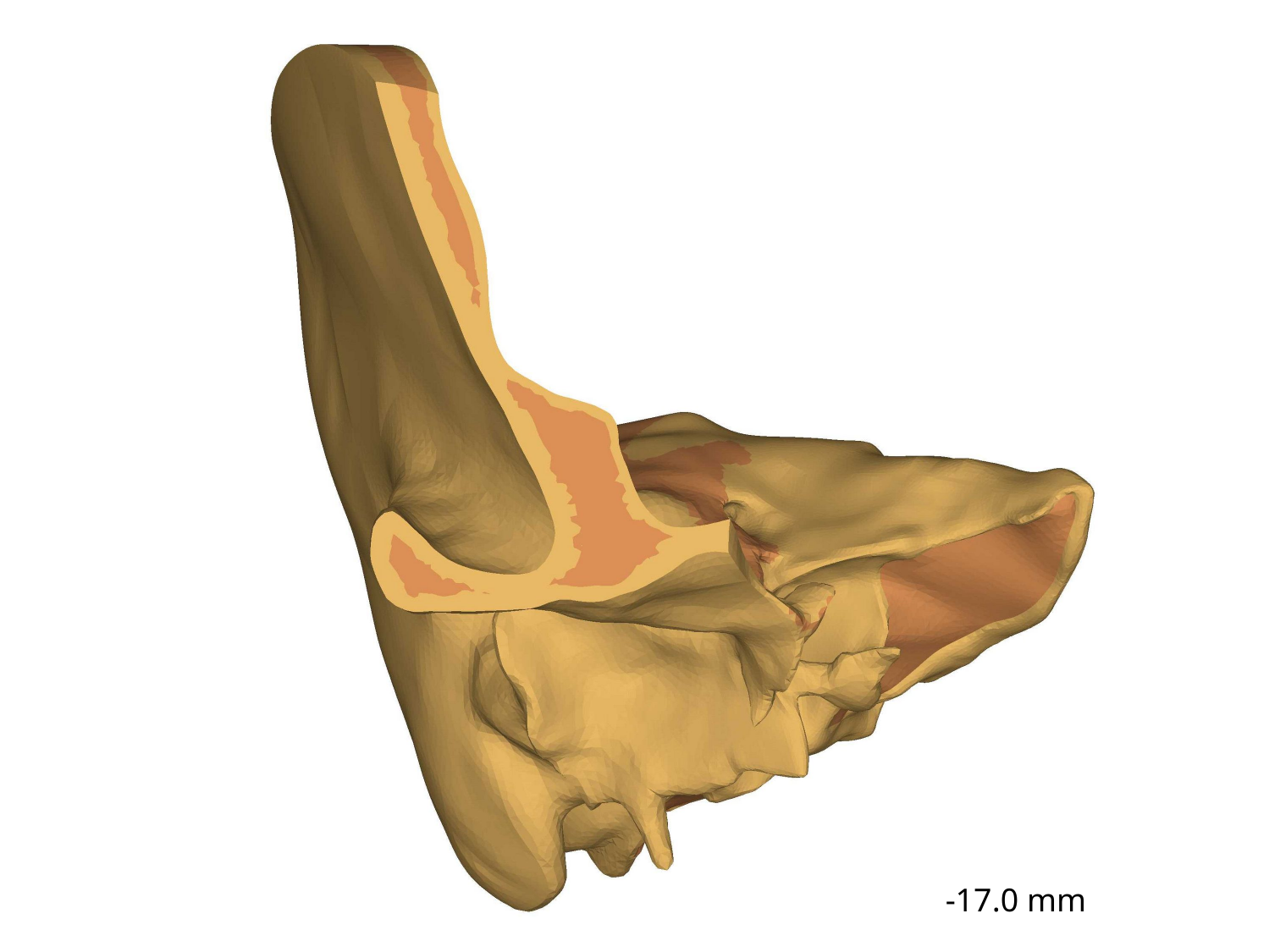

-17.0 mm

## Slide 45
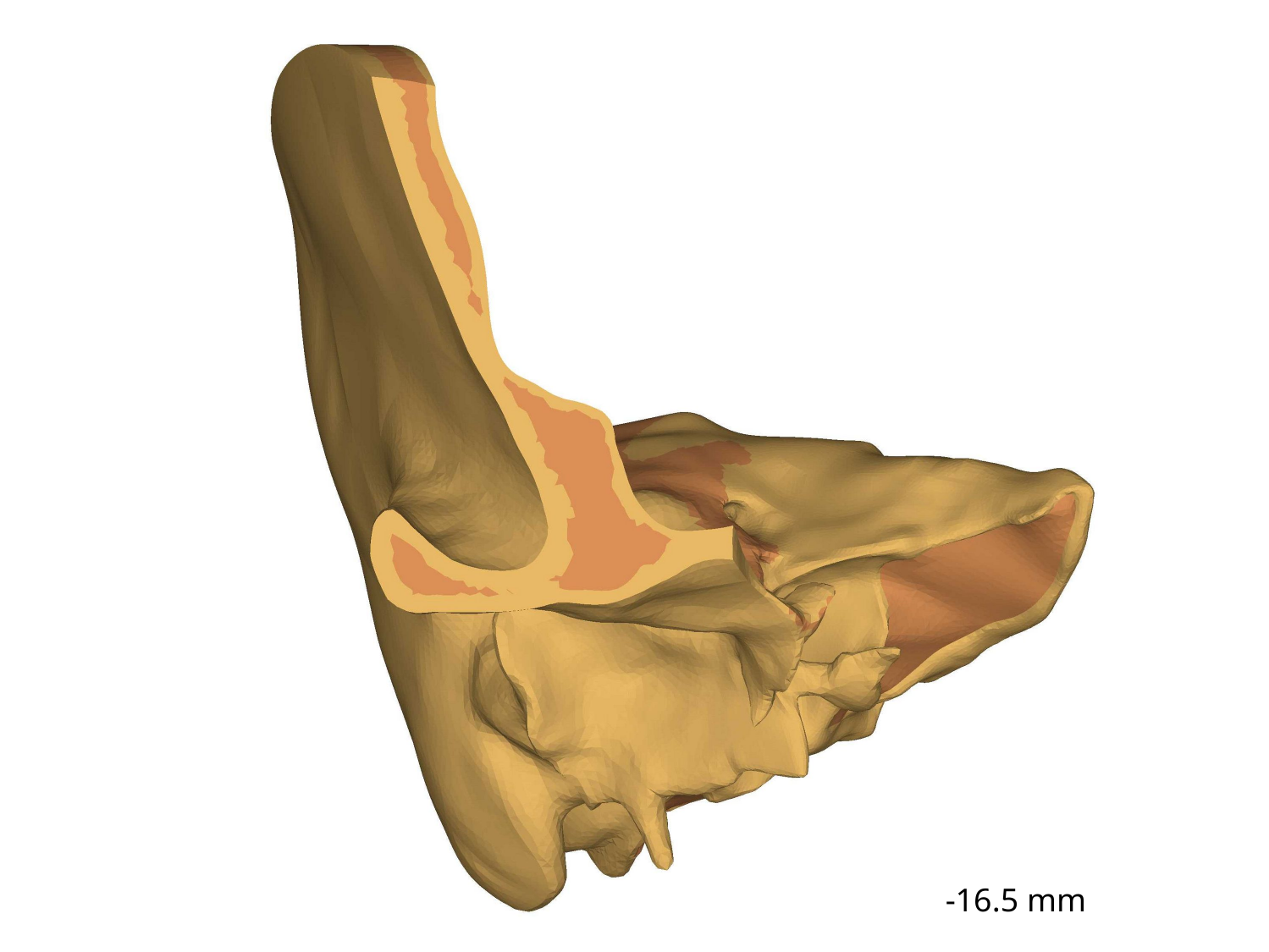

-16.5 mm

## Slide 46
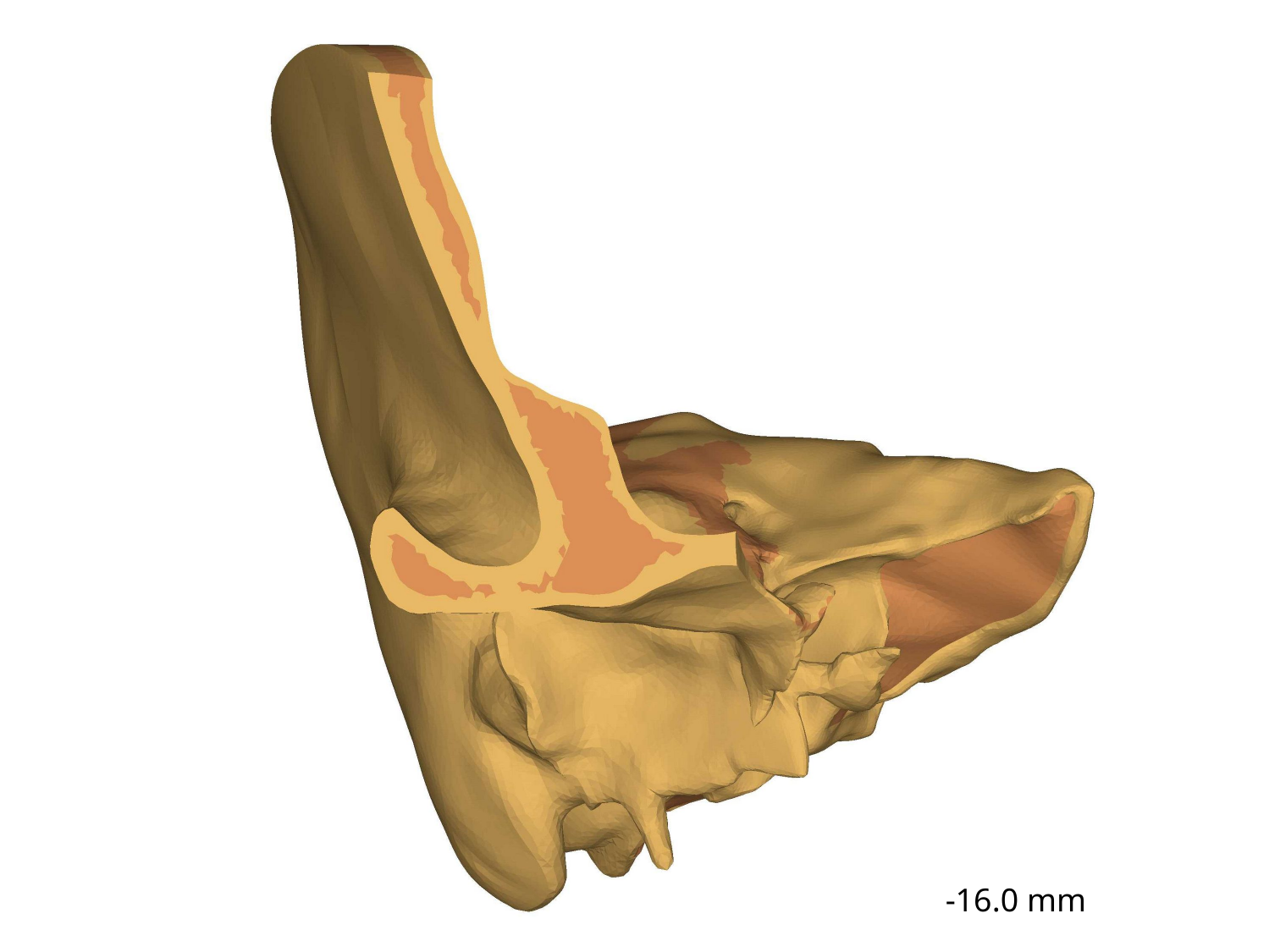

-16.0 mm

## Slide 47
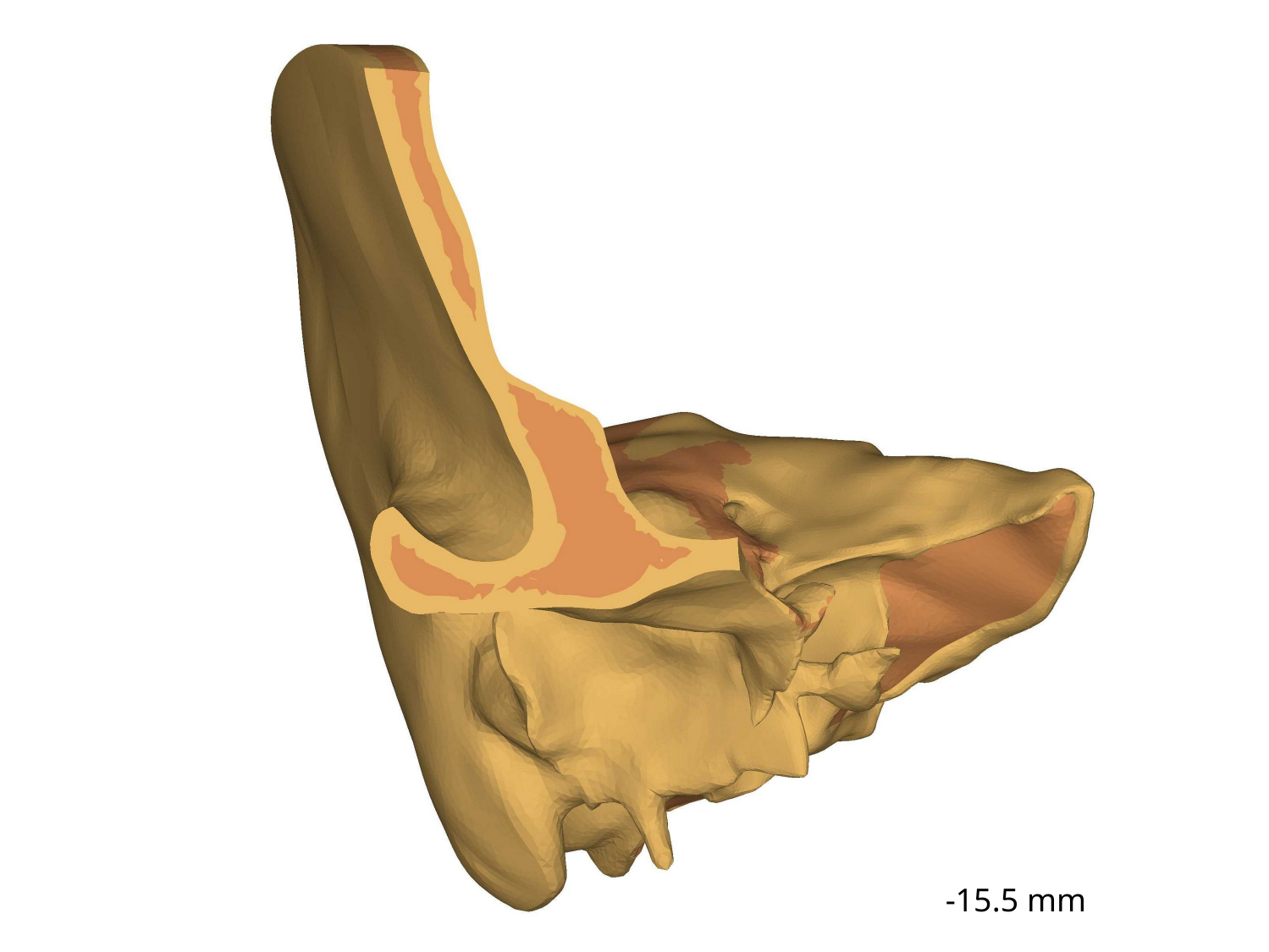

-15.5 mm

## Slide 48
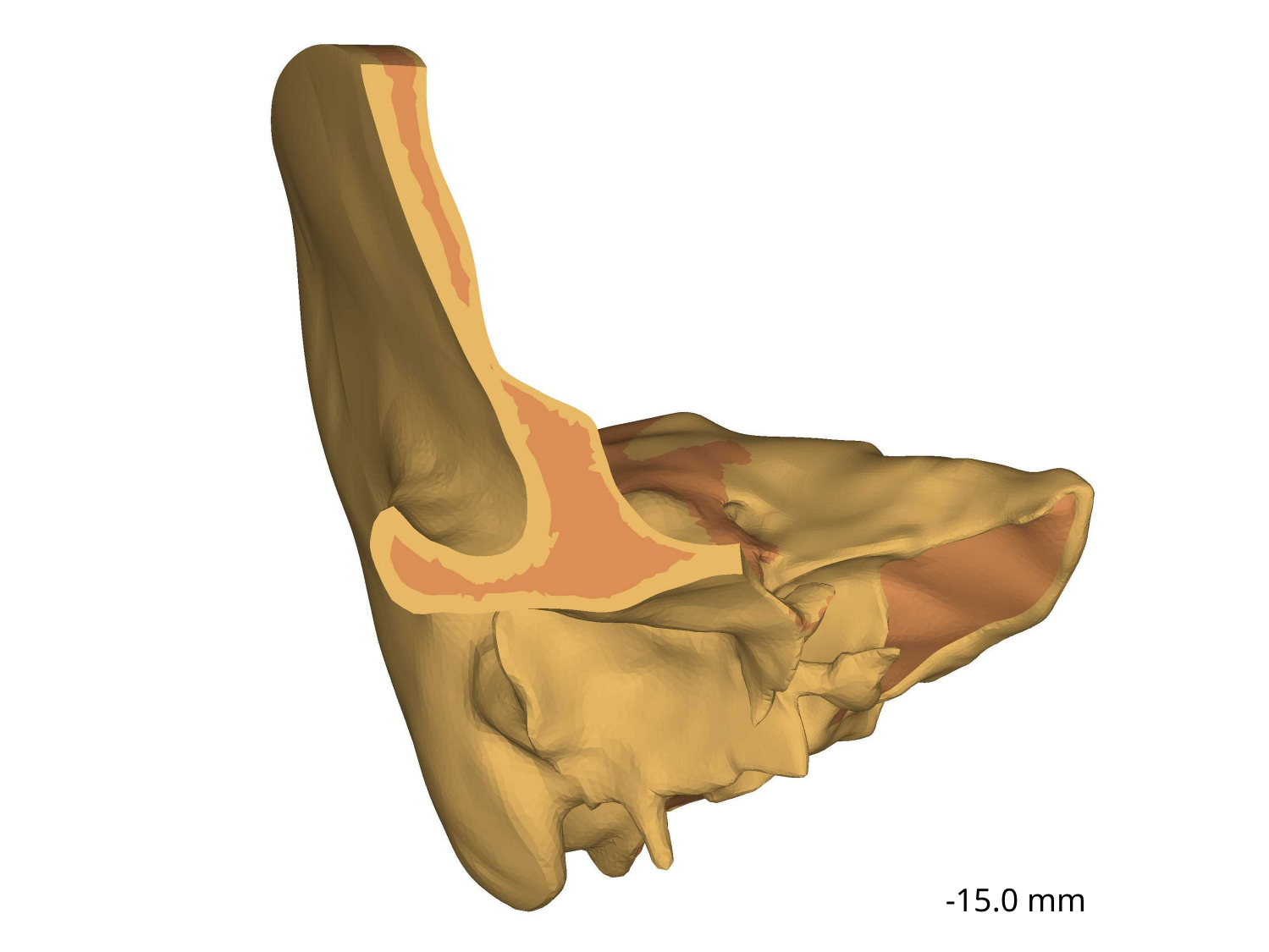

-15.0 mm

## Slide 49
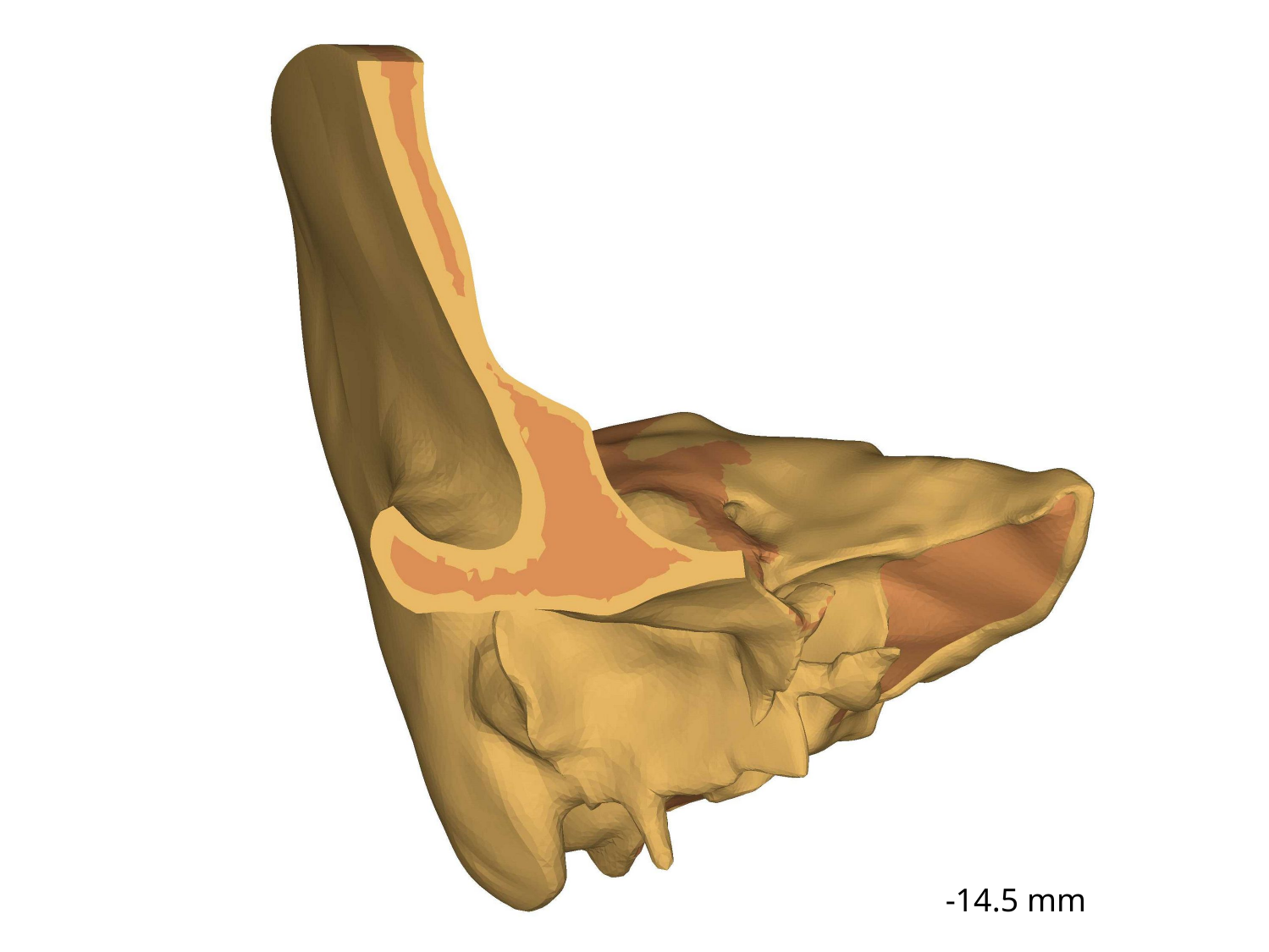

-14.5 mm

## Slide 50
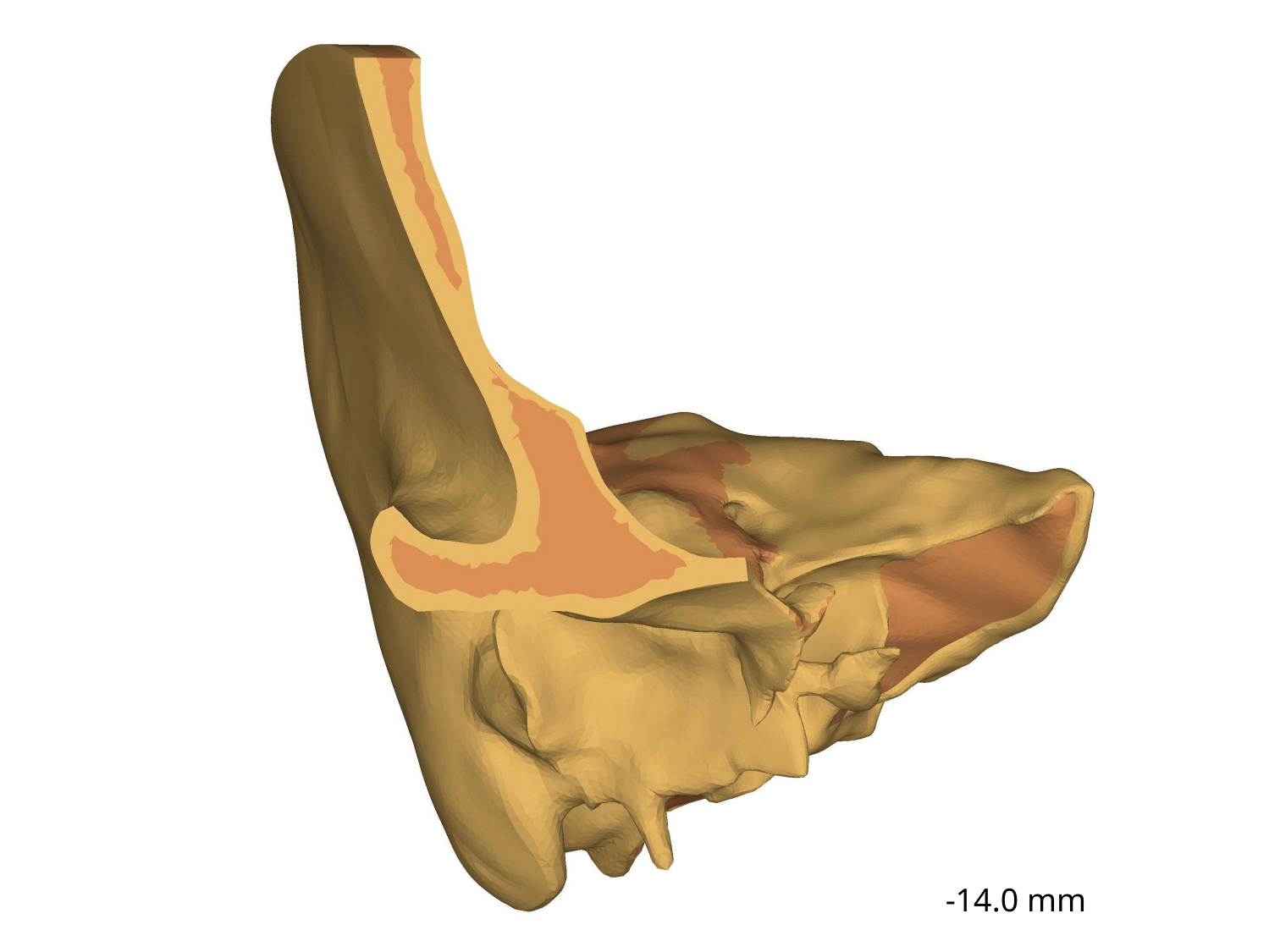

-14.0 mm

## Slide 51
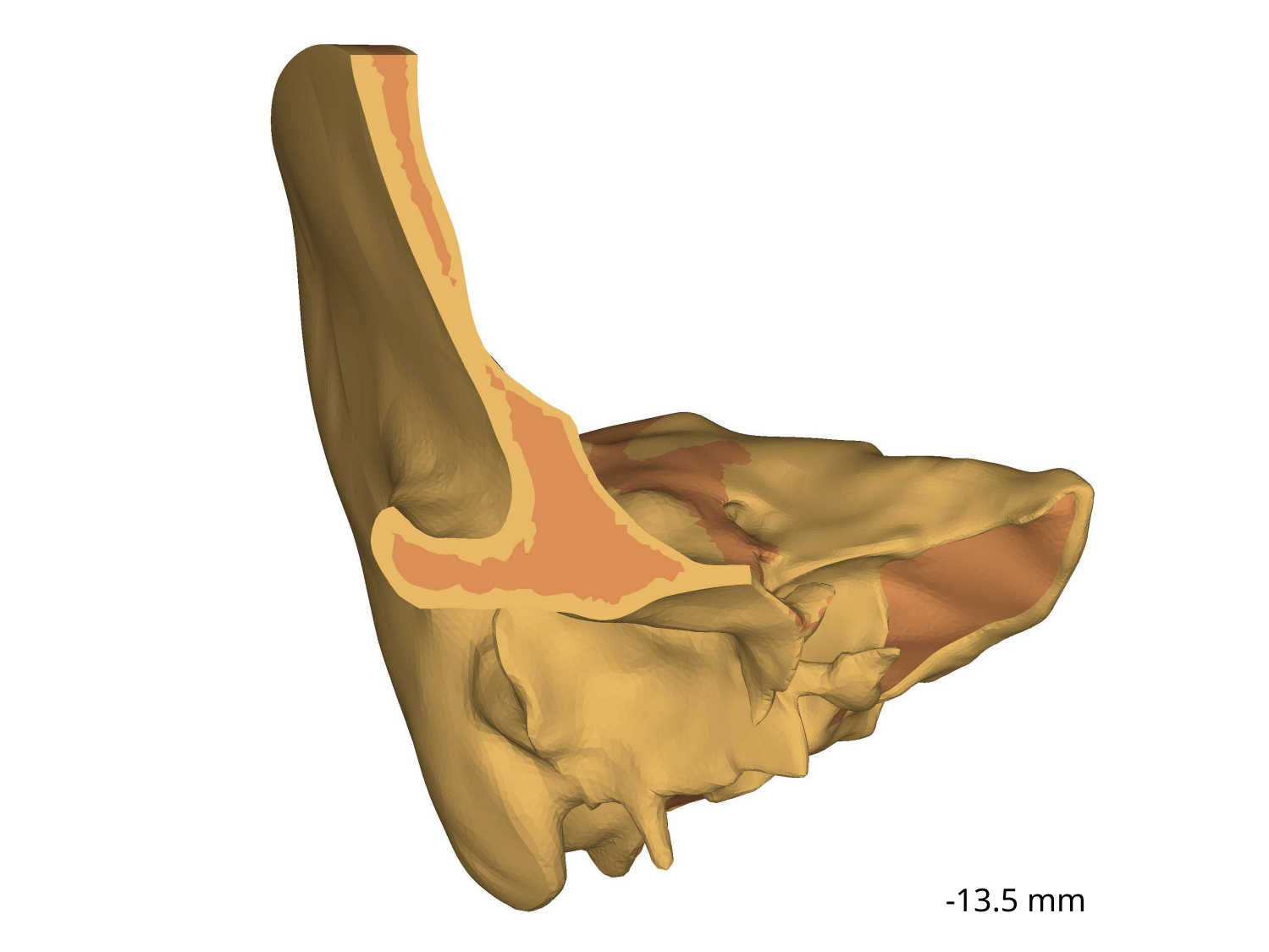

-13.5 mm

## Slide 52
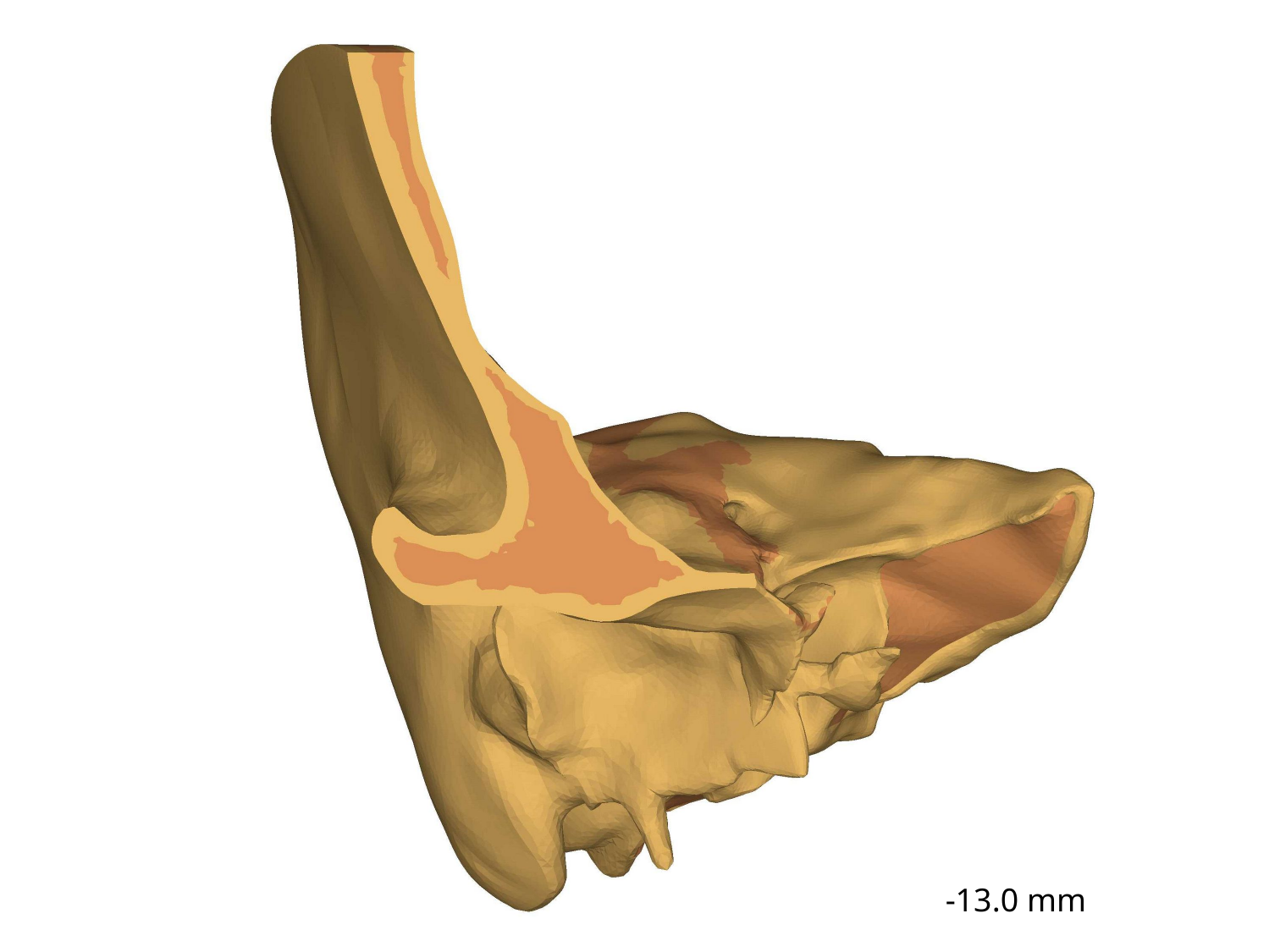

-13.0 mm

## Slide 53
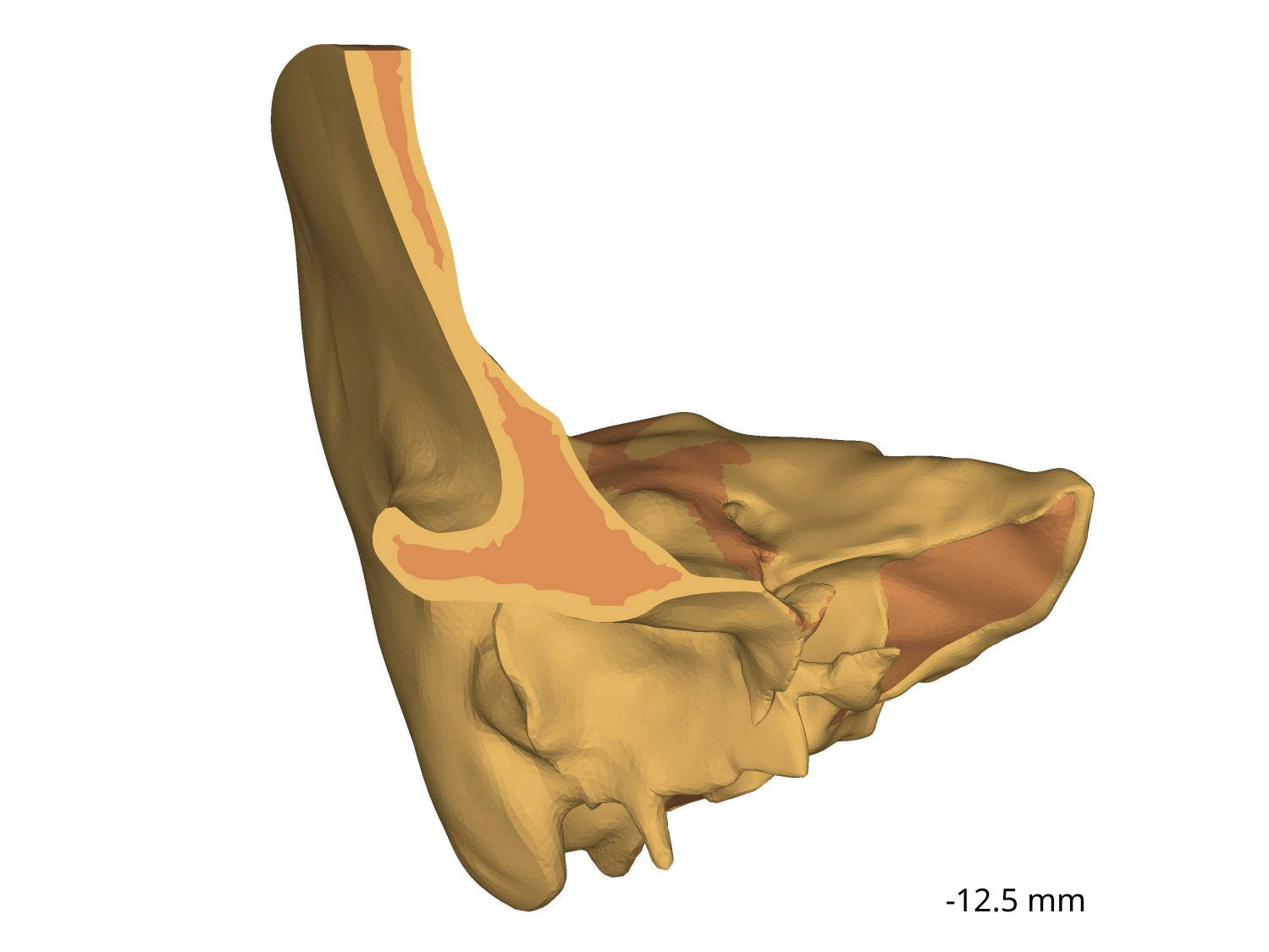

-12.5 mm

## Slide 54
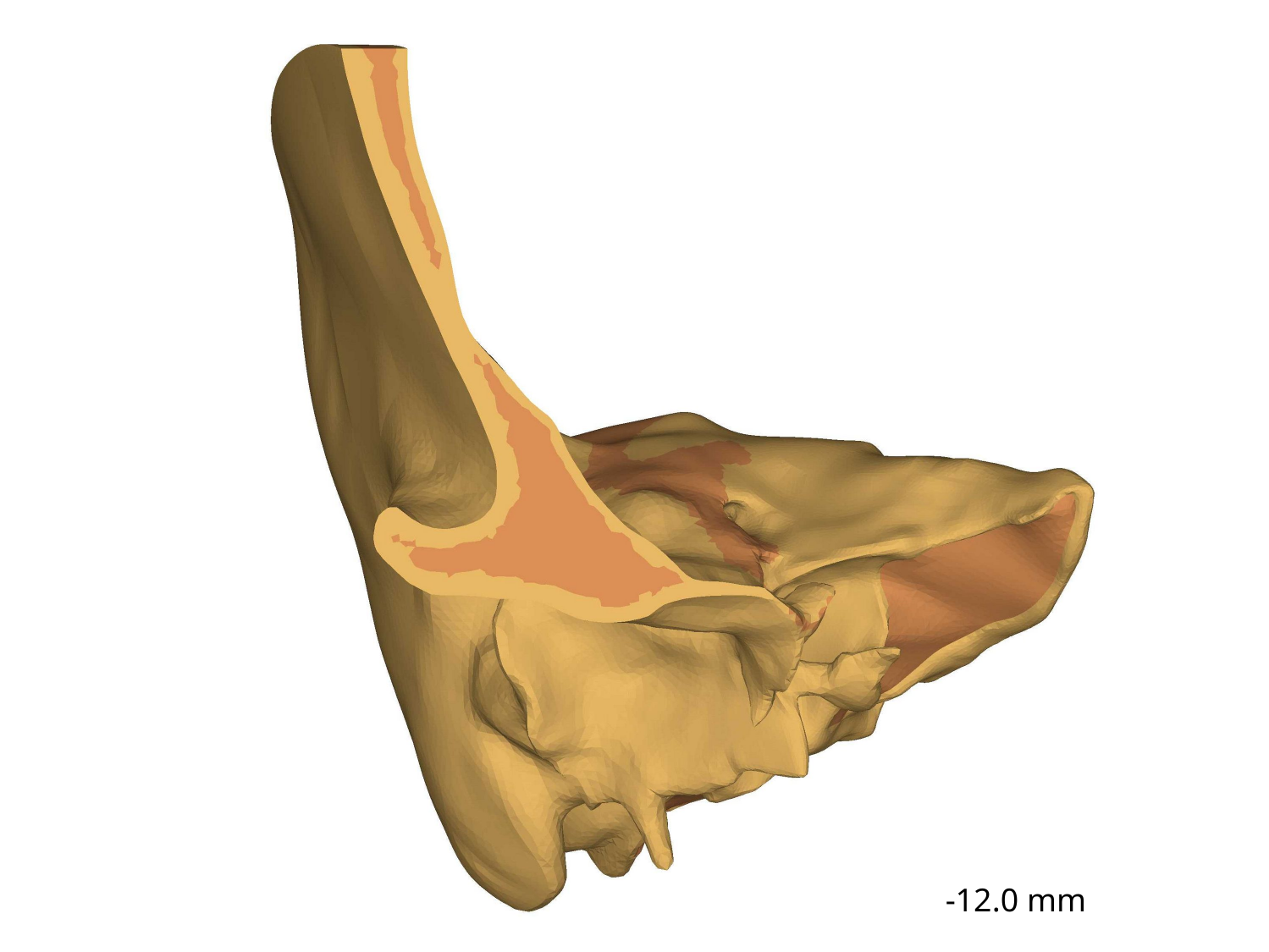

-12.0 mm

## Slide 55
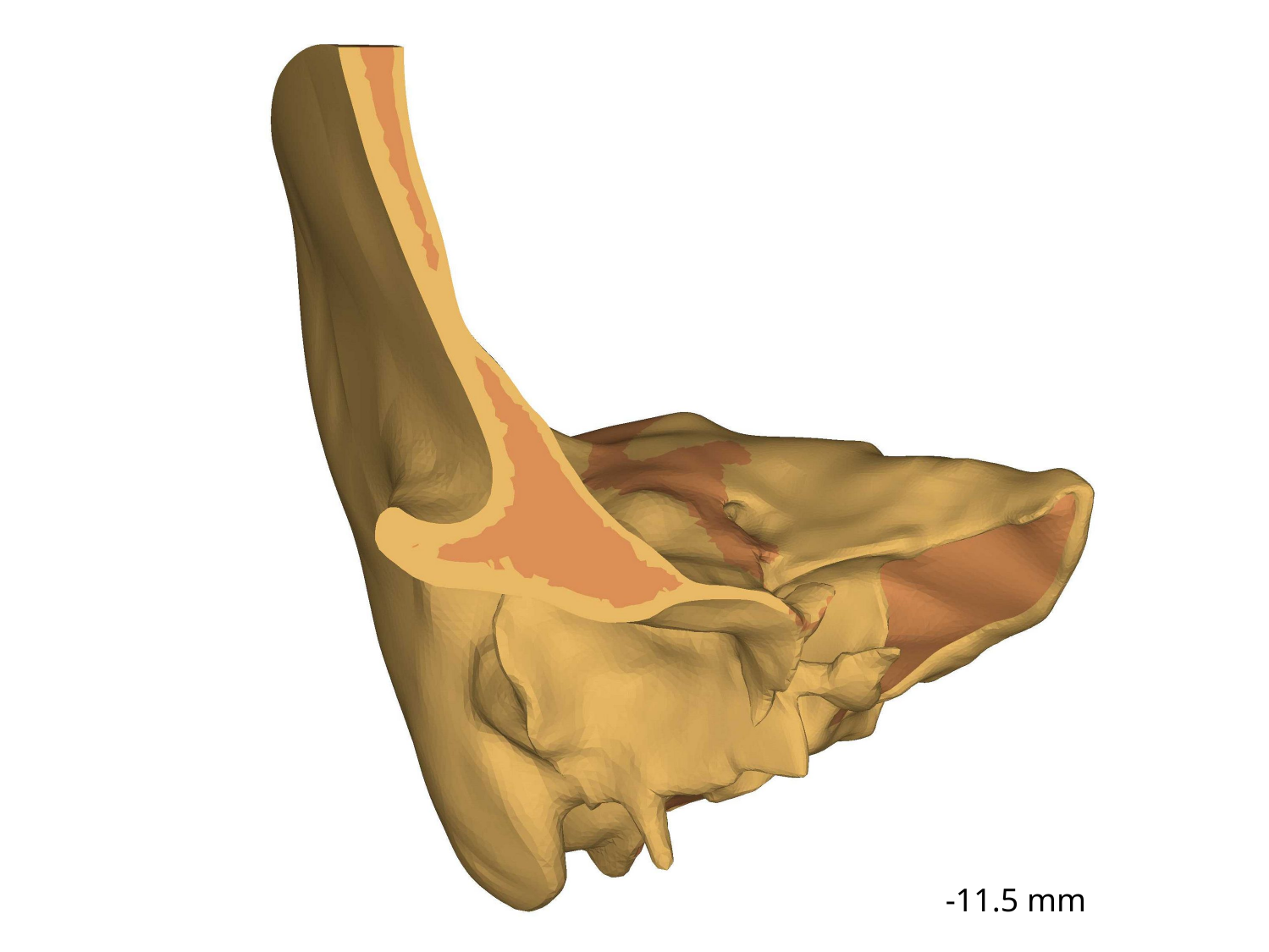

-11.5 mm

## Slide 56
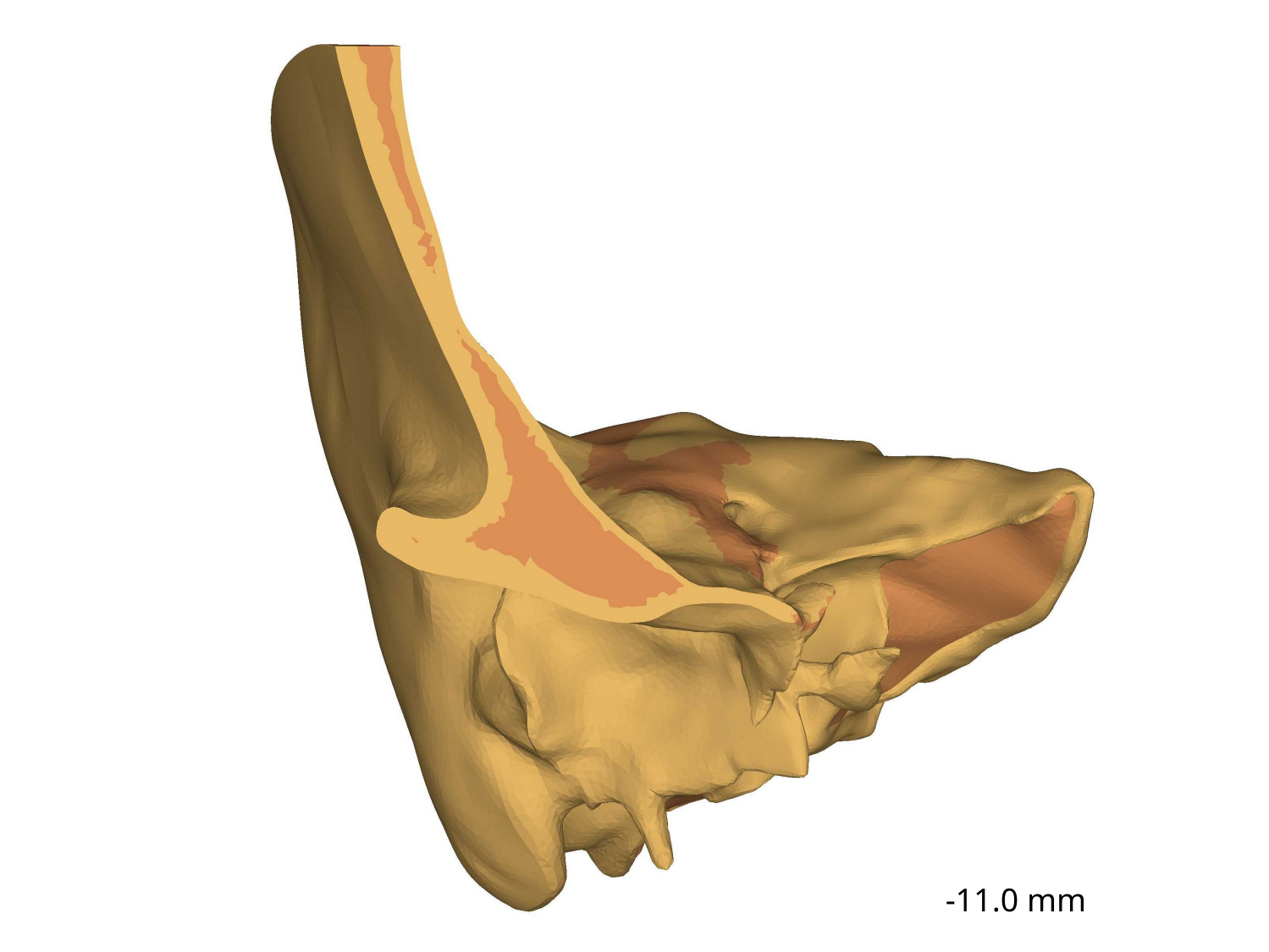

-11.0 mm

## Slide 57
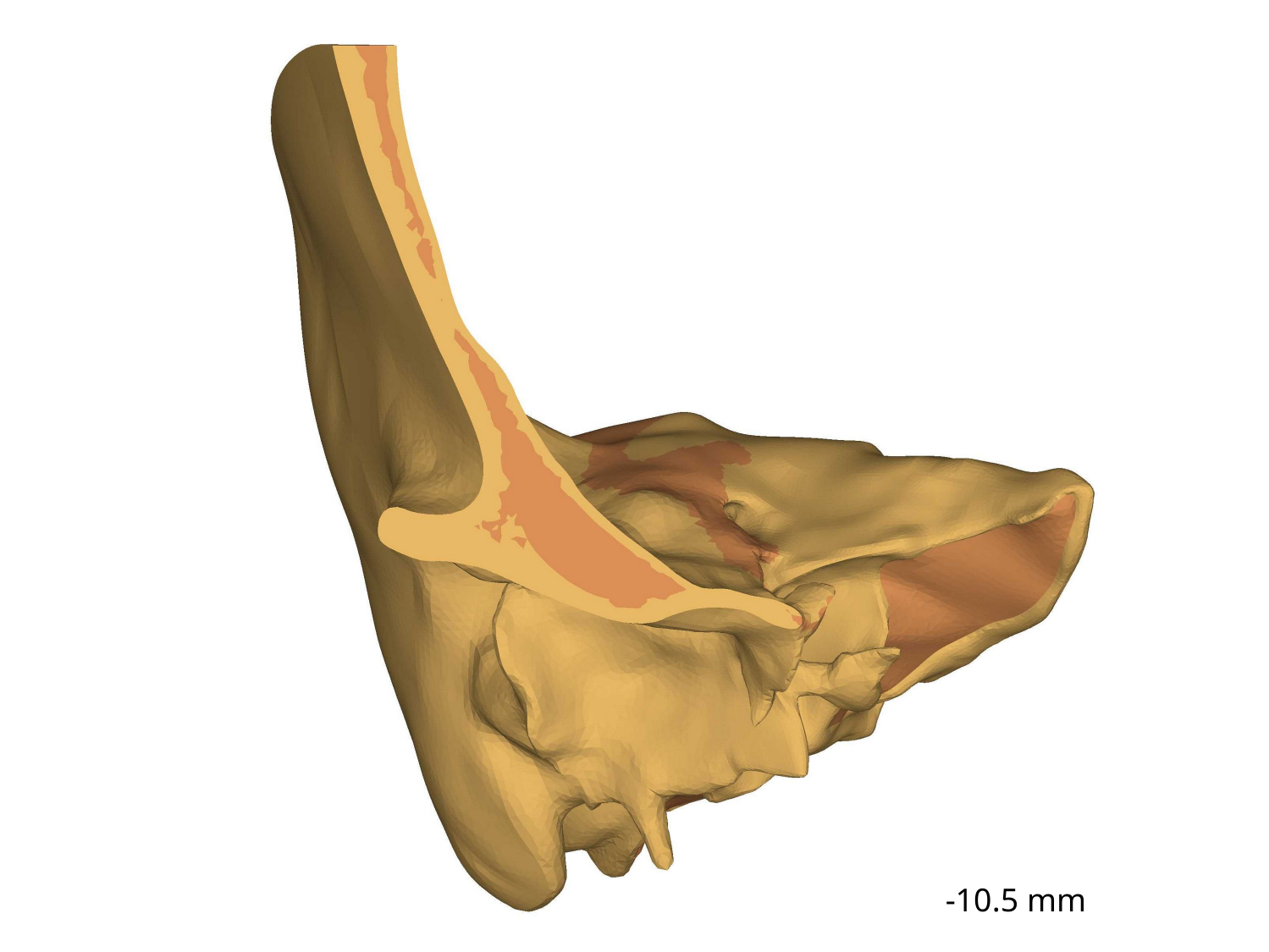

-10.5 mm

## Slide 58
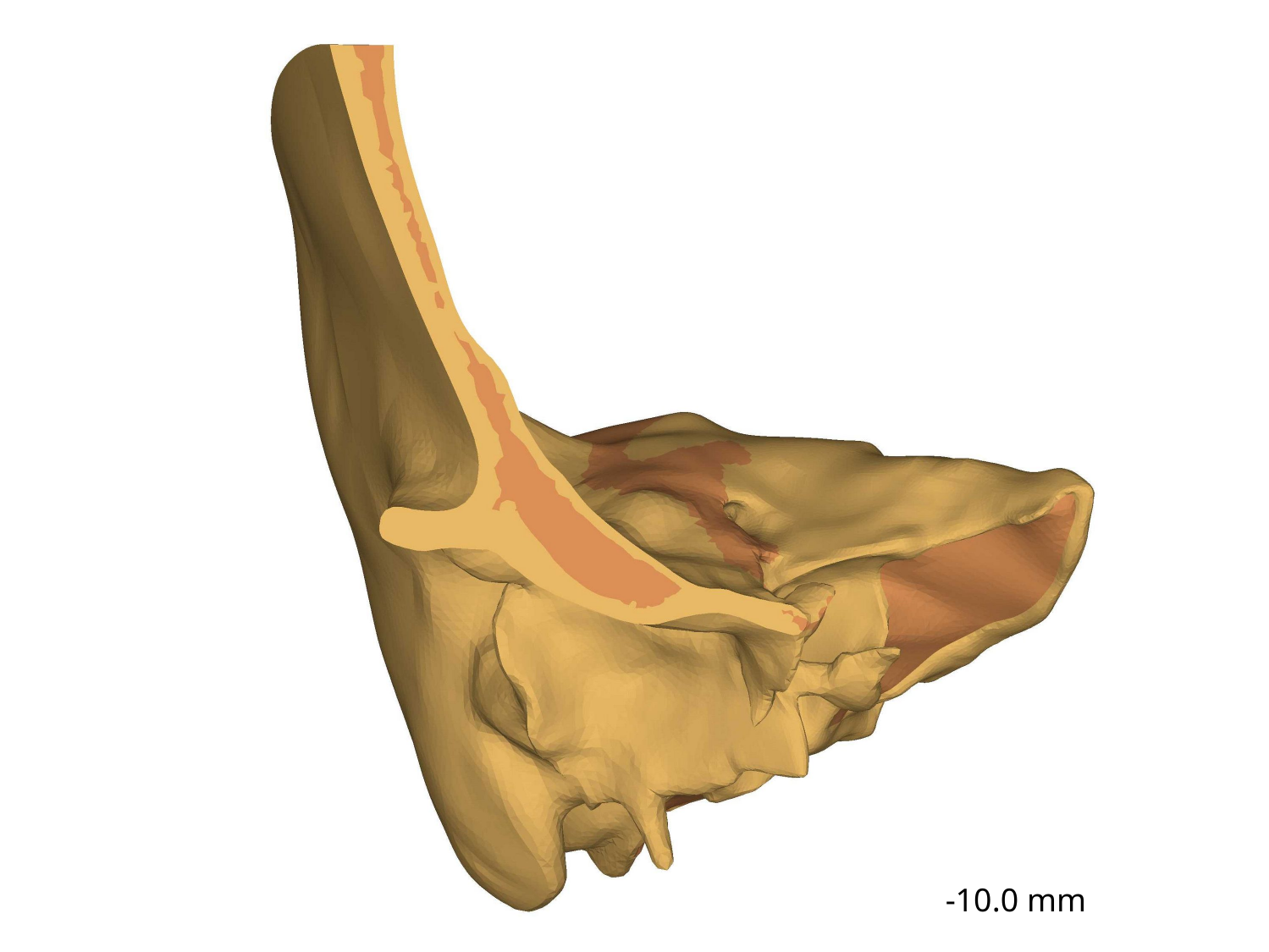

-10.0 mm

## Slide 59
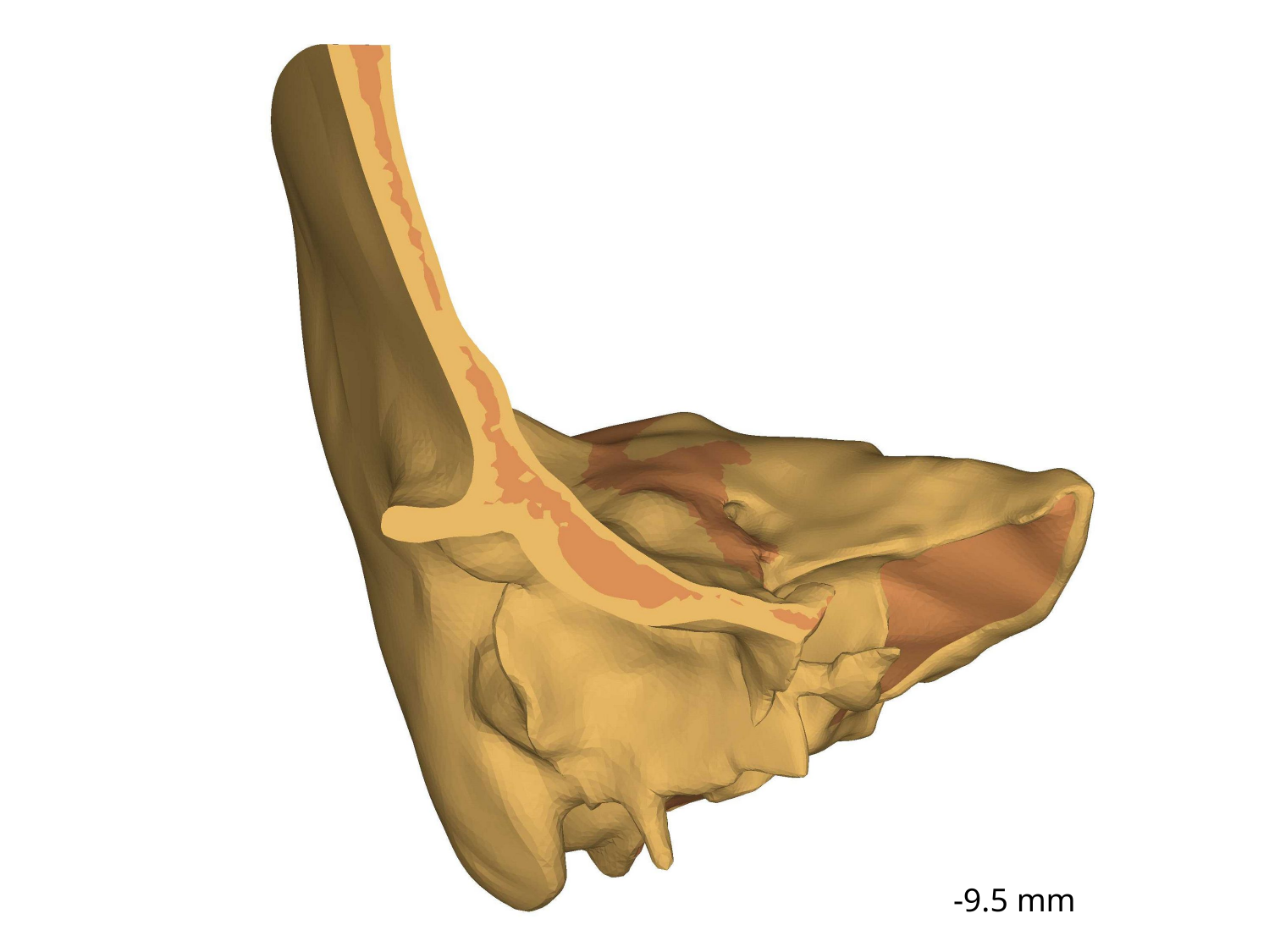

-9.5 mm

## Slide 60
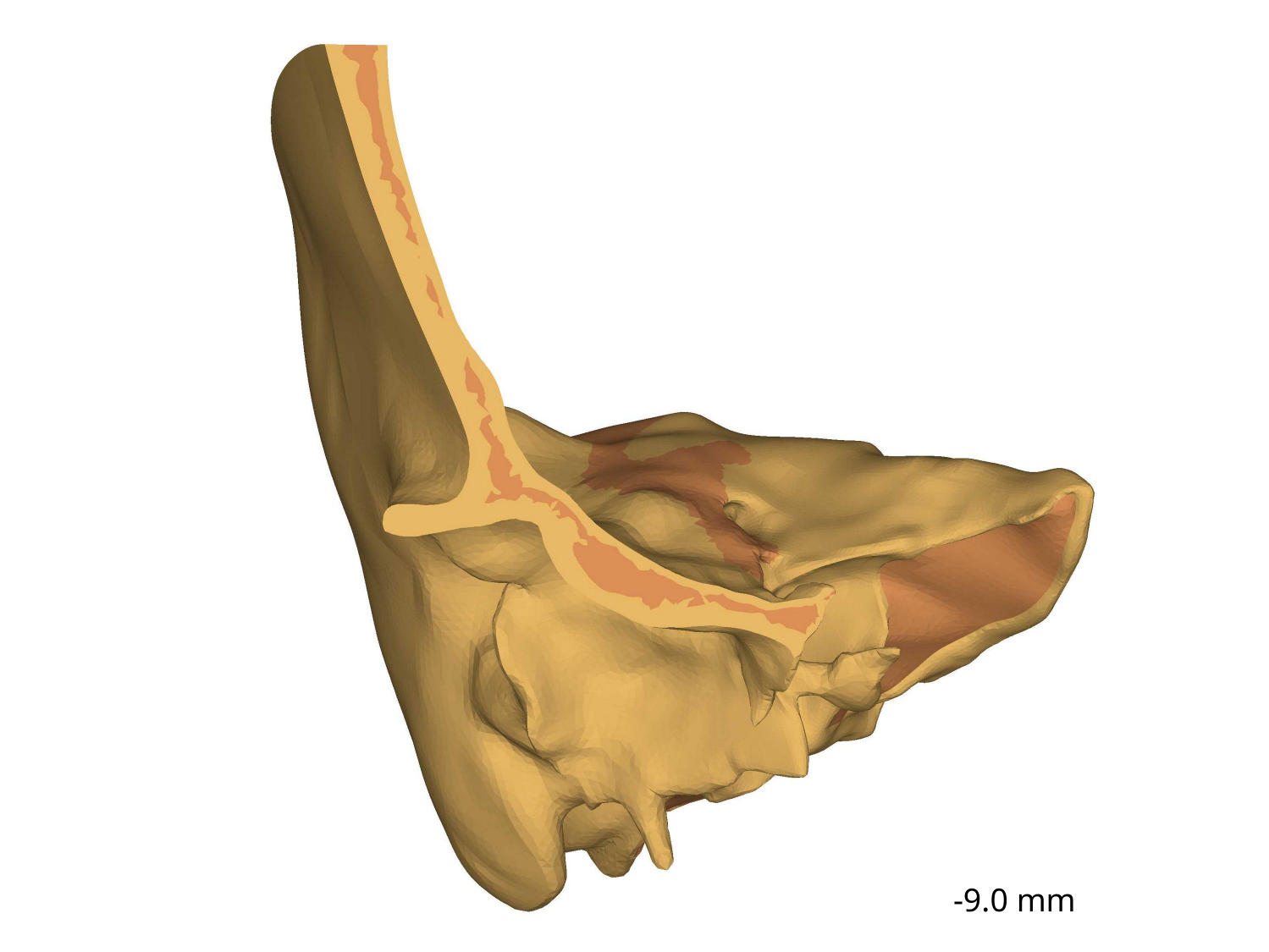

-9.0 mm

## Slide 61
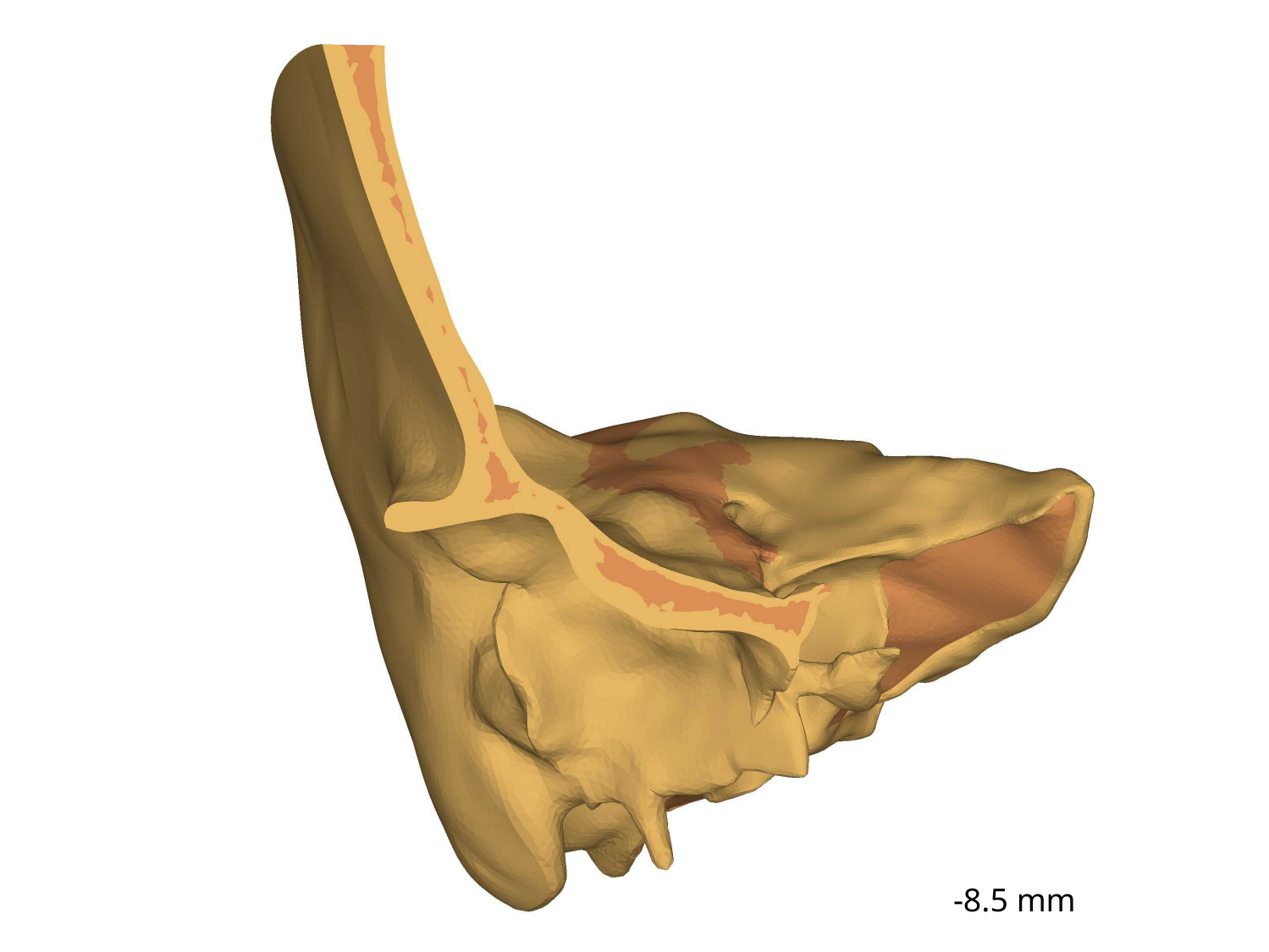

-8.5 mm

## Slide 62
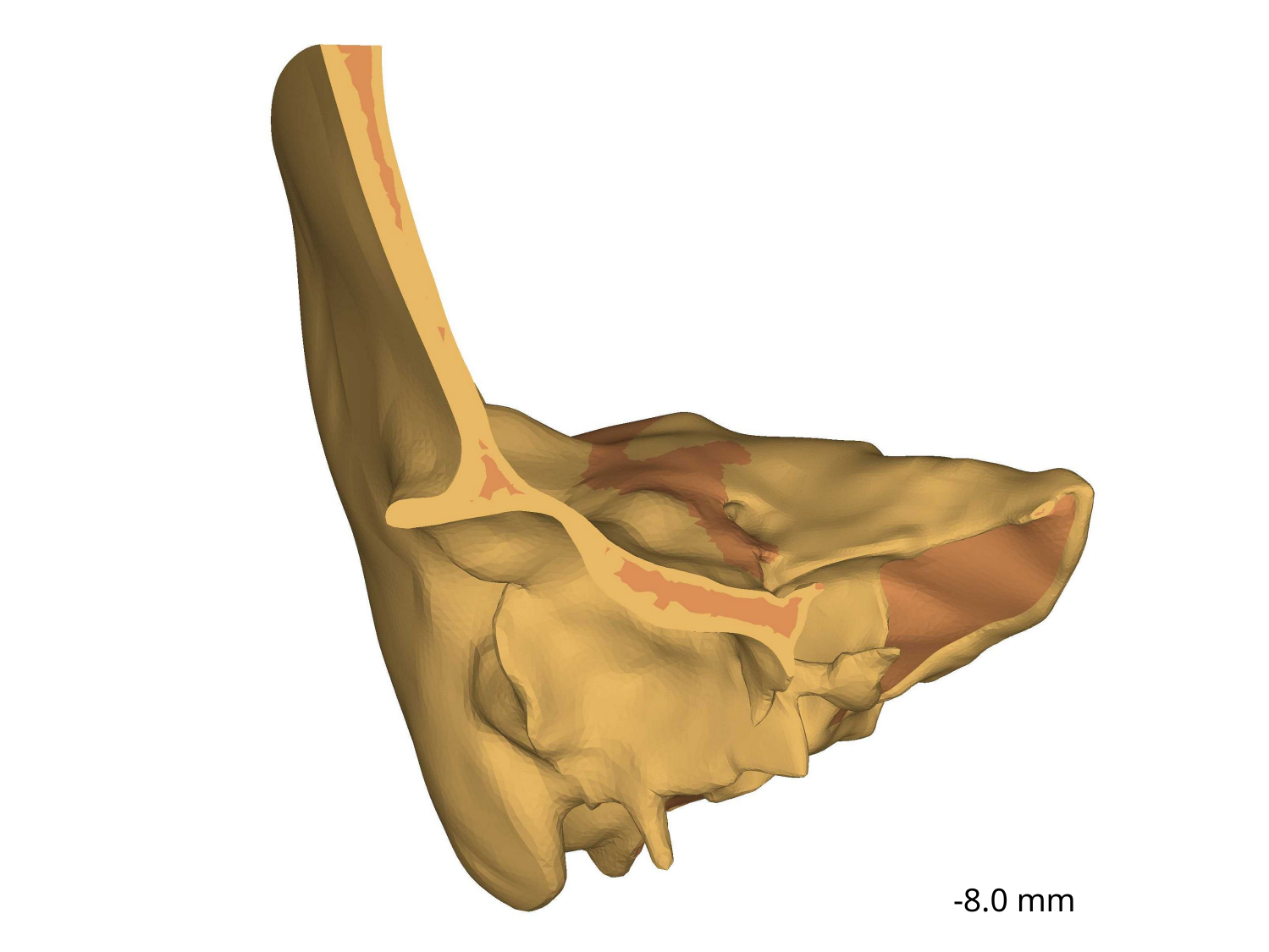

-8.0 mm

## Slide 63
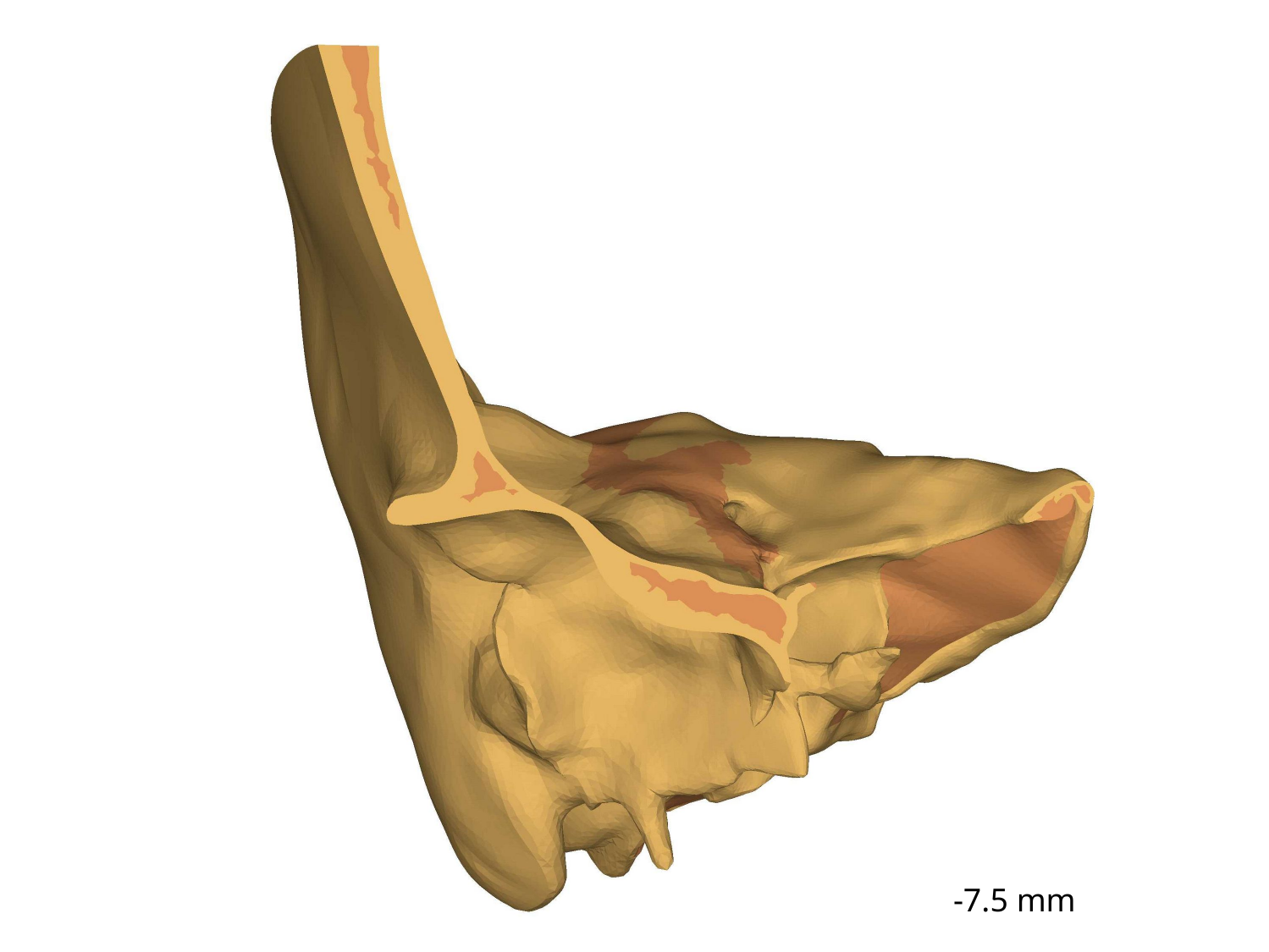

-7.5 mm

## Slide 64
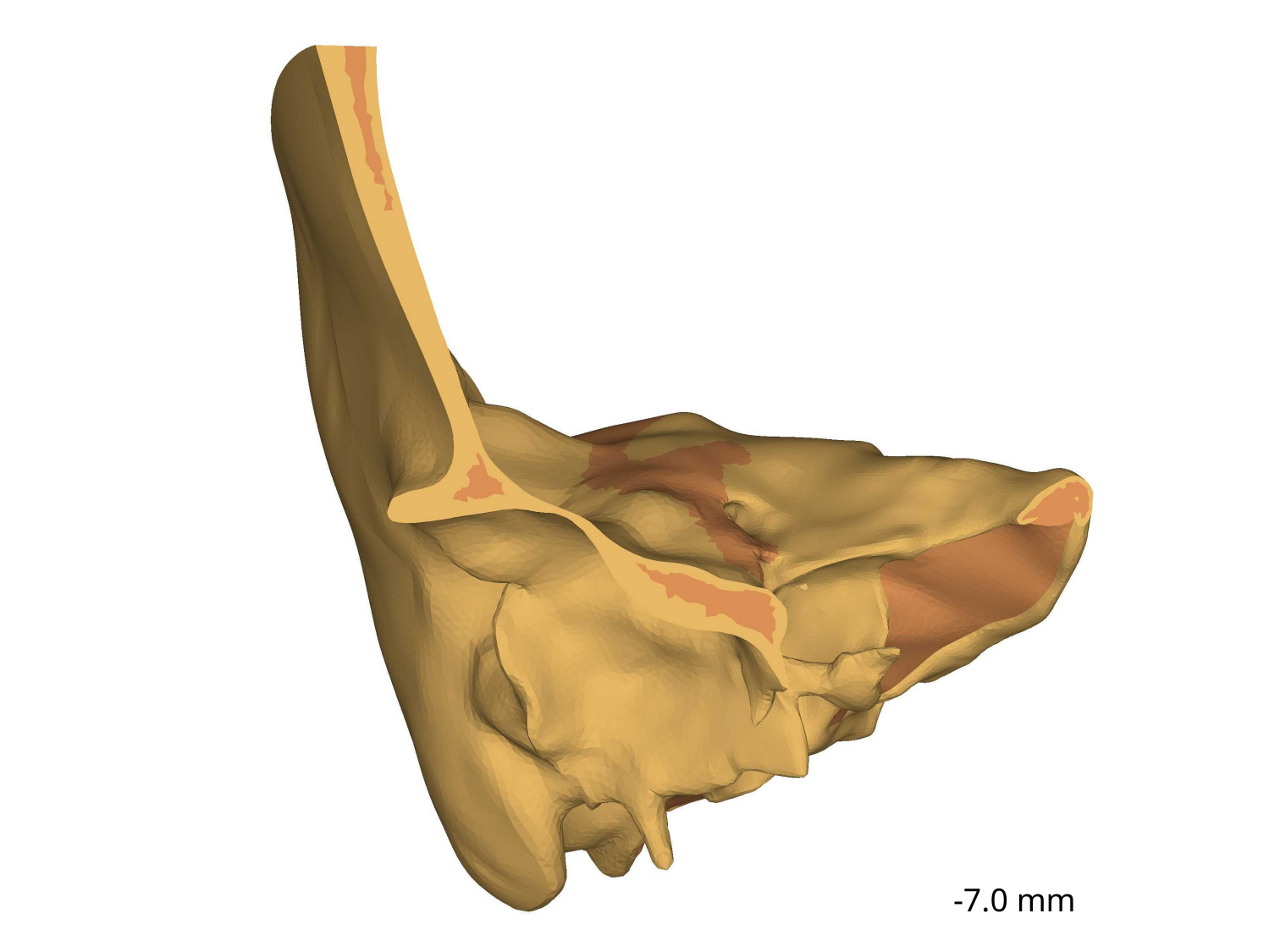

-7.0 mm

## Slide 65
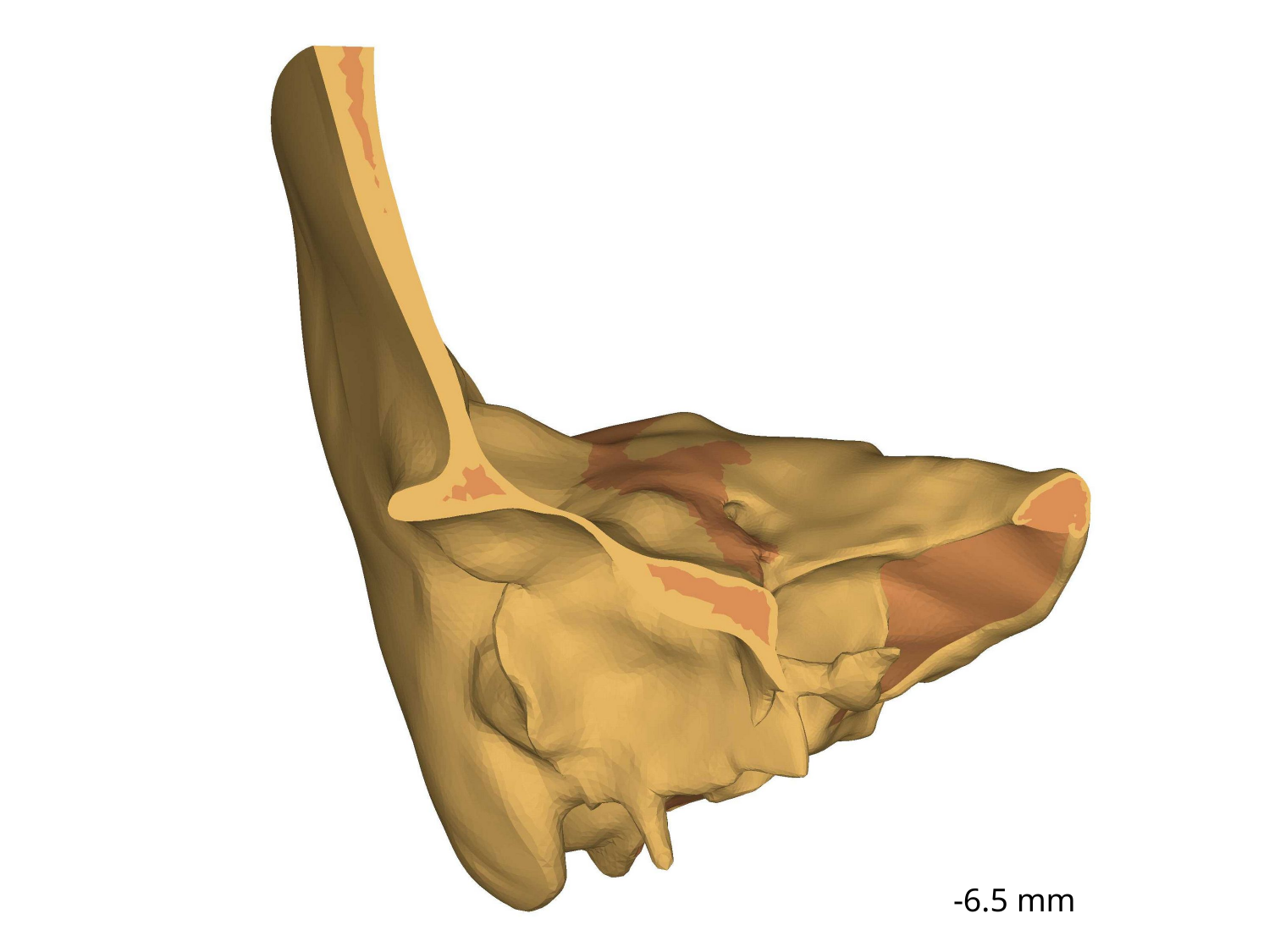

-6.5 mm

## Slide 66
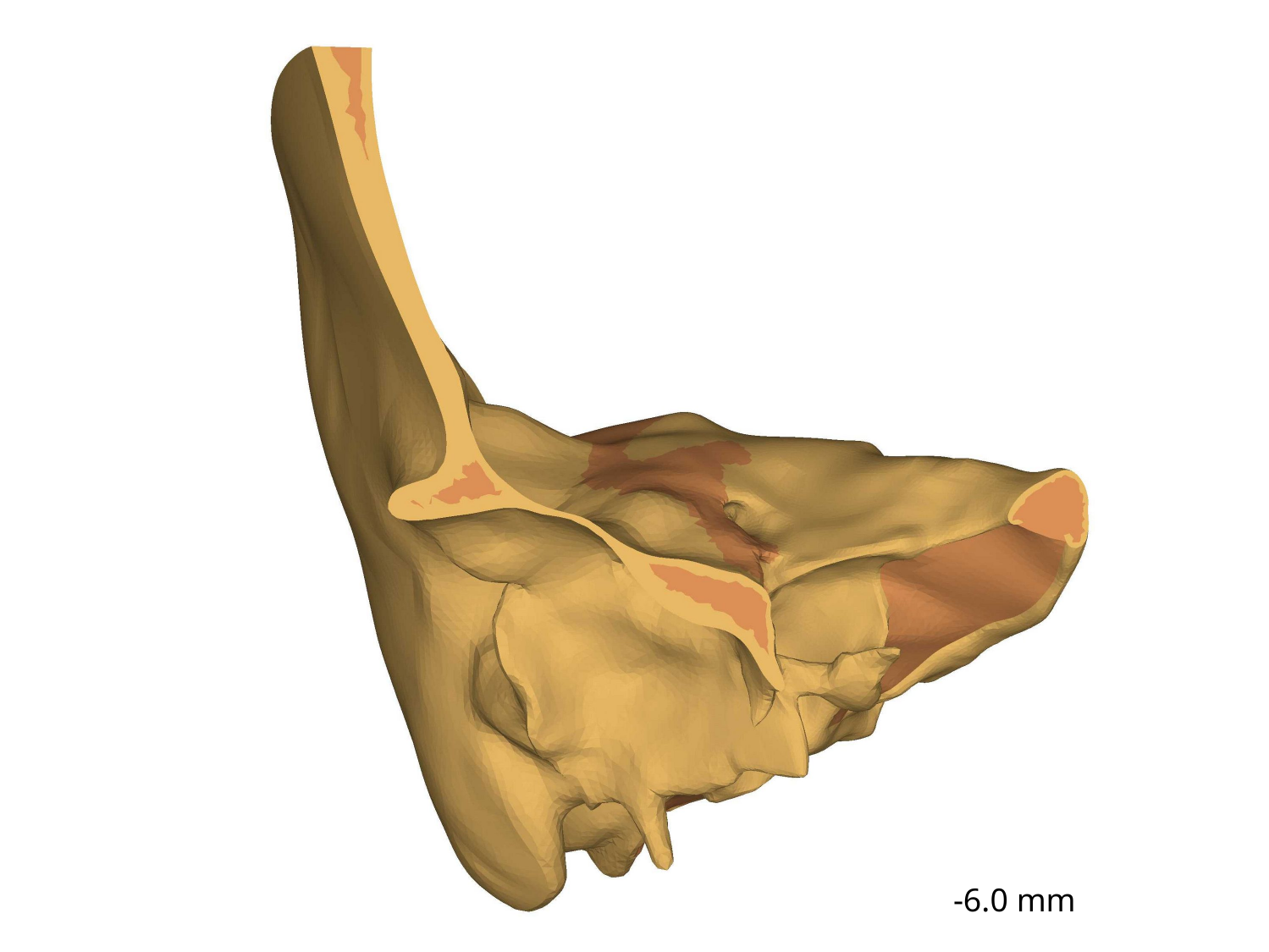

-6.0 mm

## Slide 67
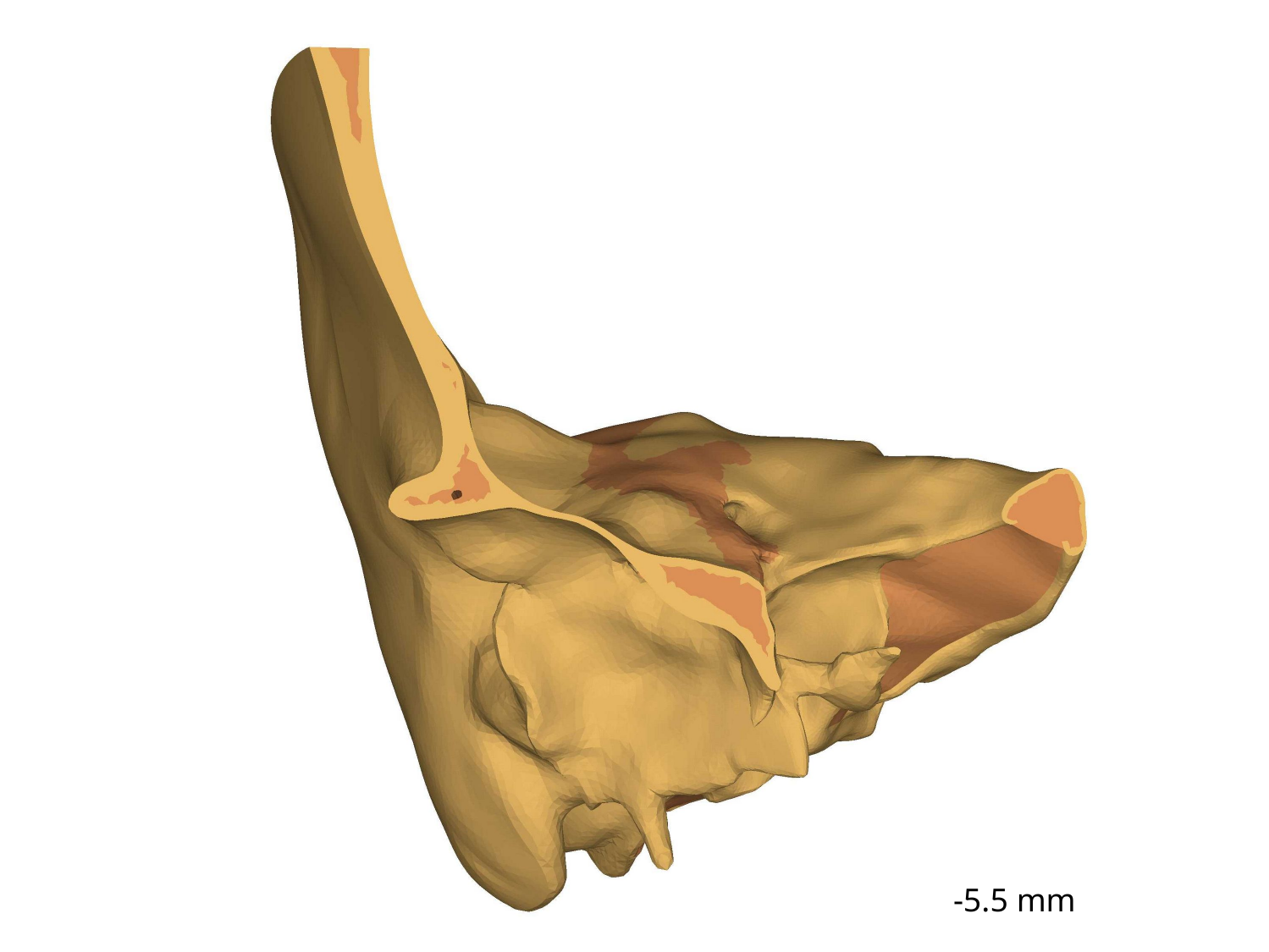

-5.5 mm

## Slide 68
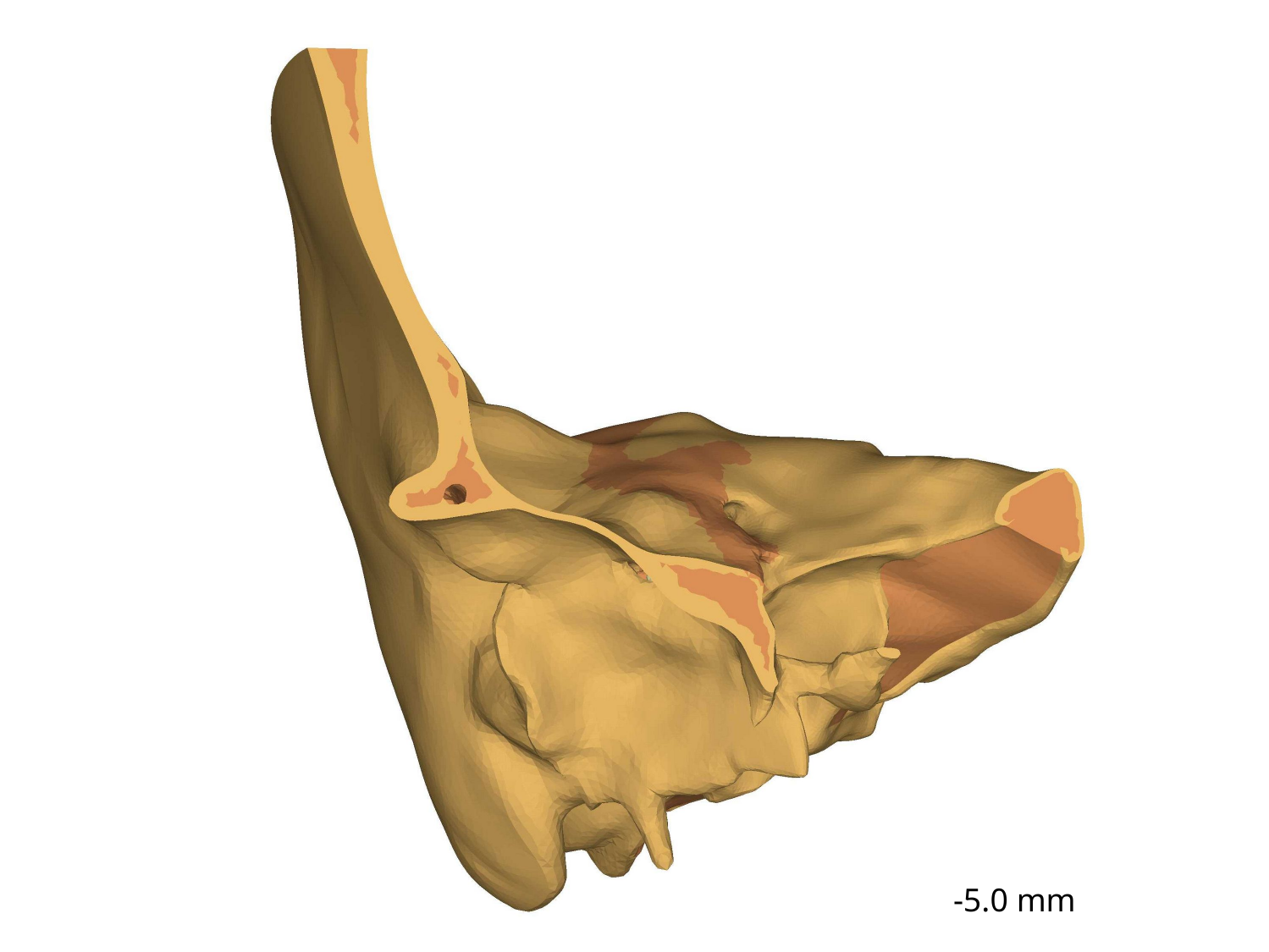

-5.0 mm

## Slide 69
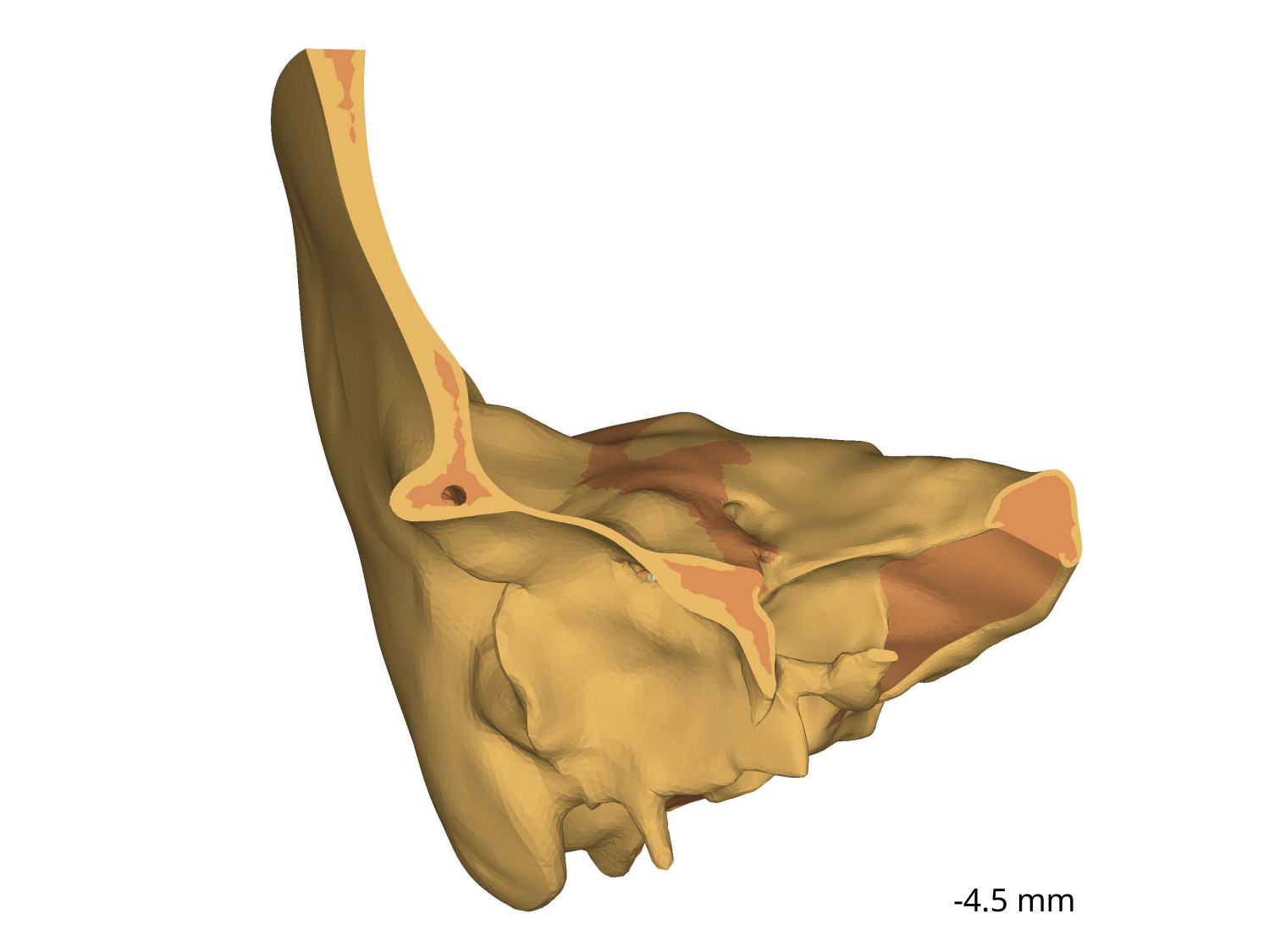

-4.5 mm

## Slide 70
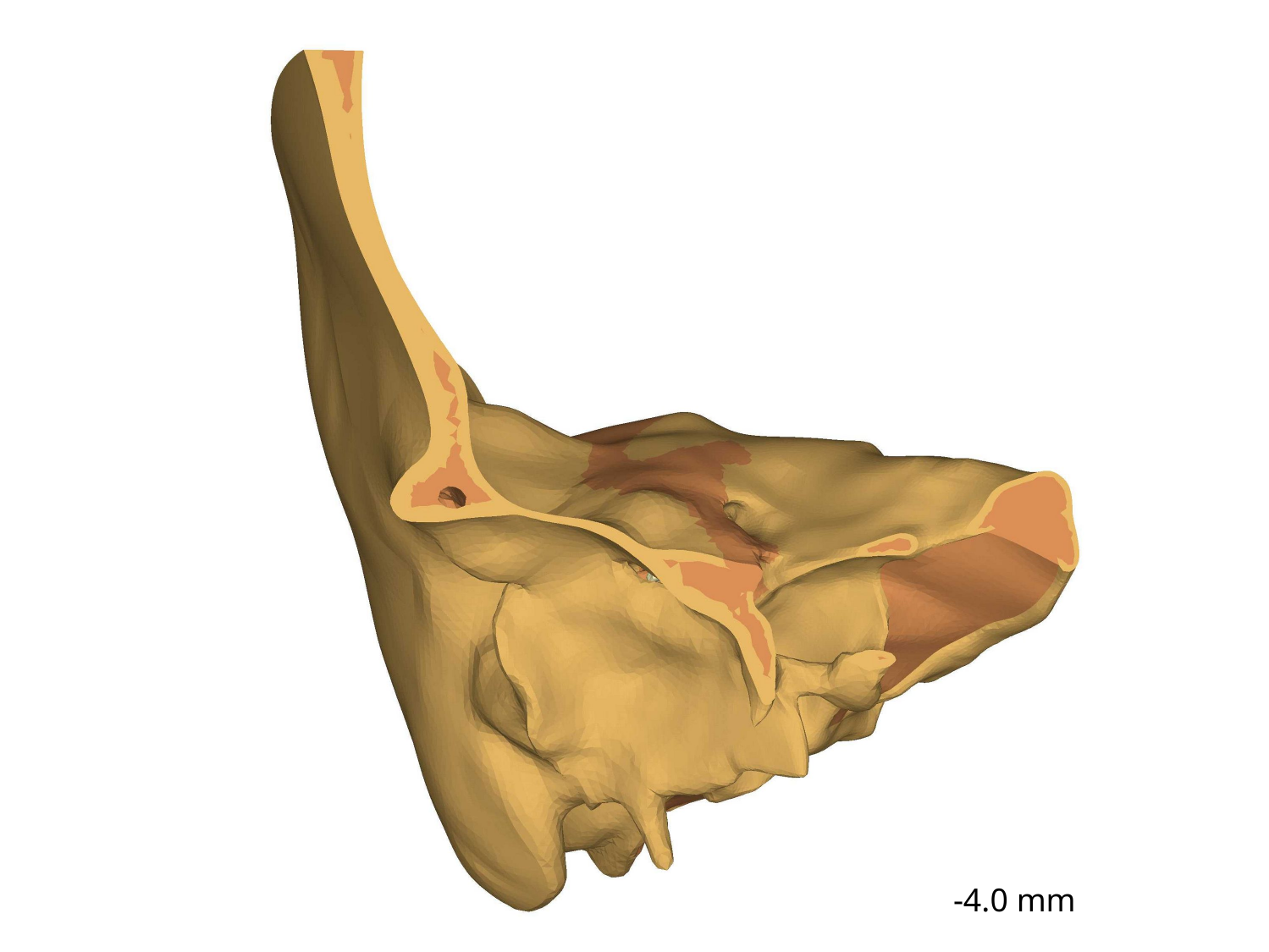

-4.0 mm

## Slide 71
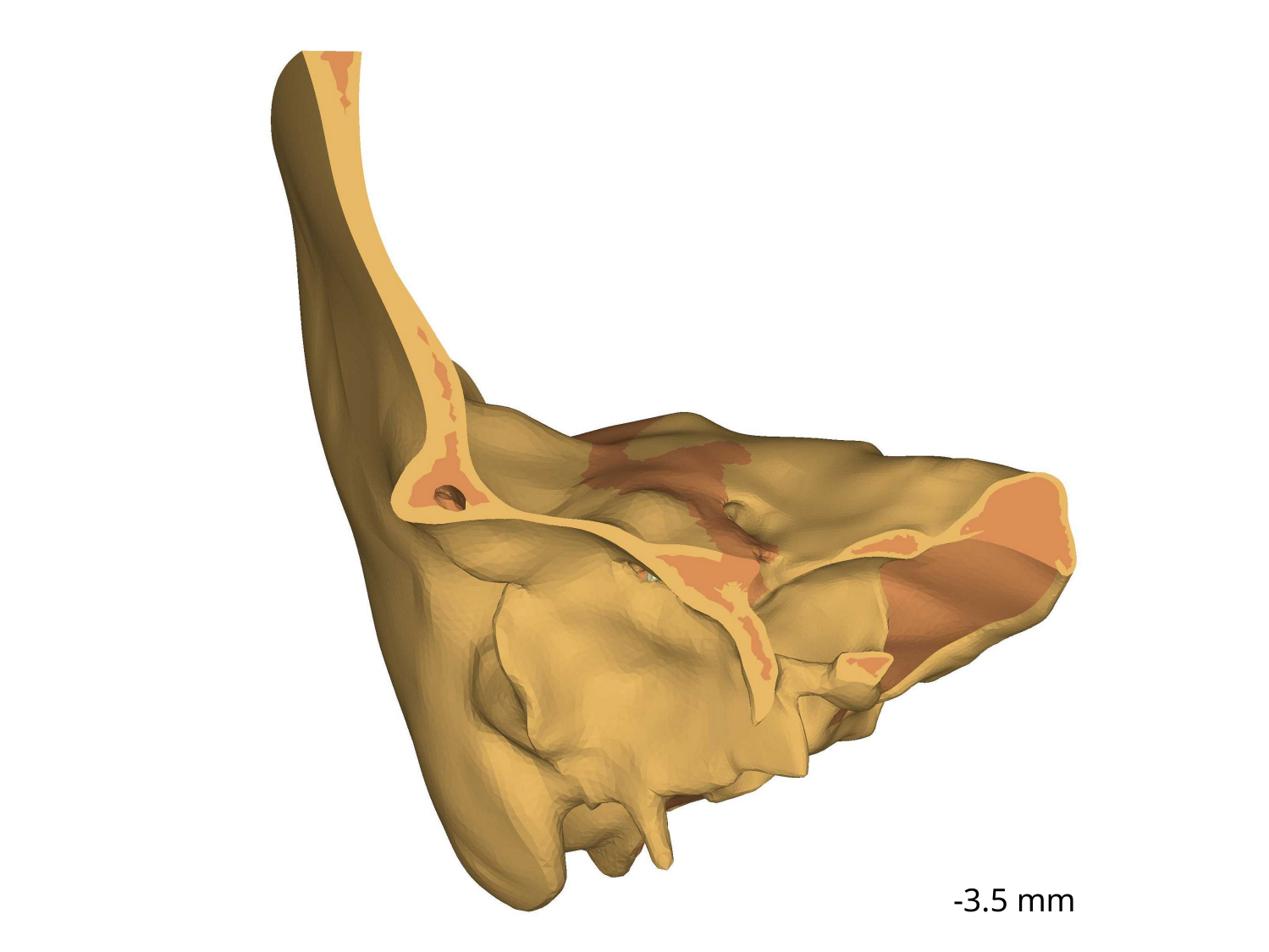

-3.5 mm

## Slide 72
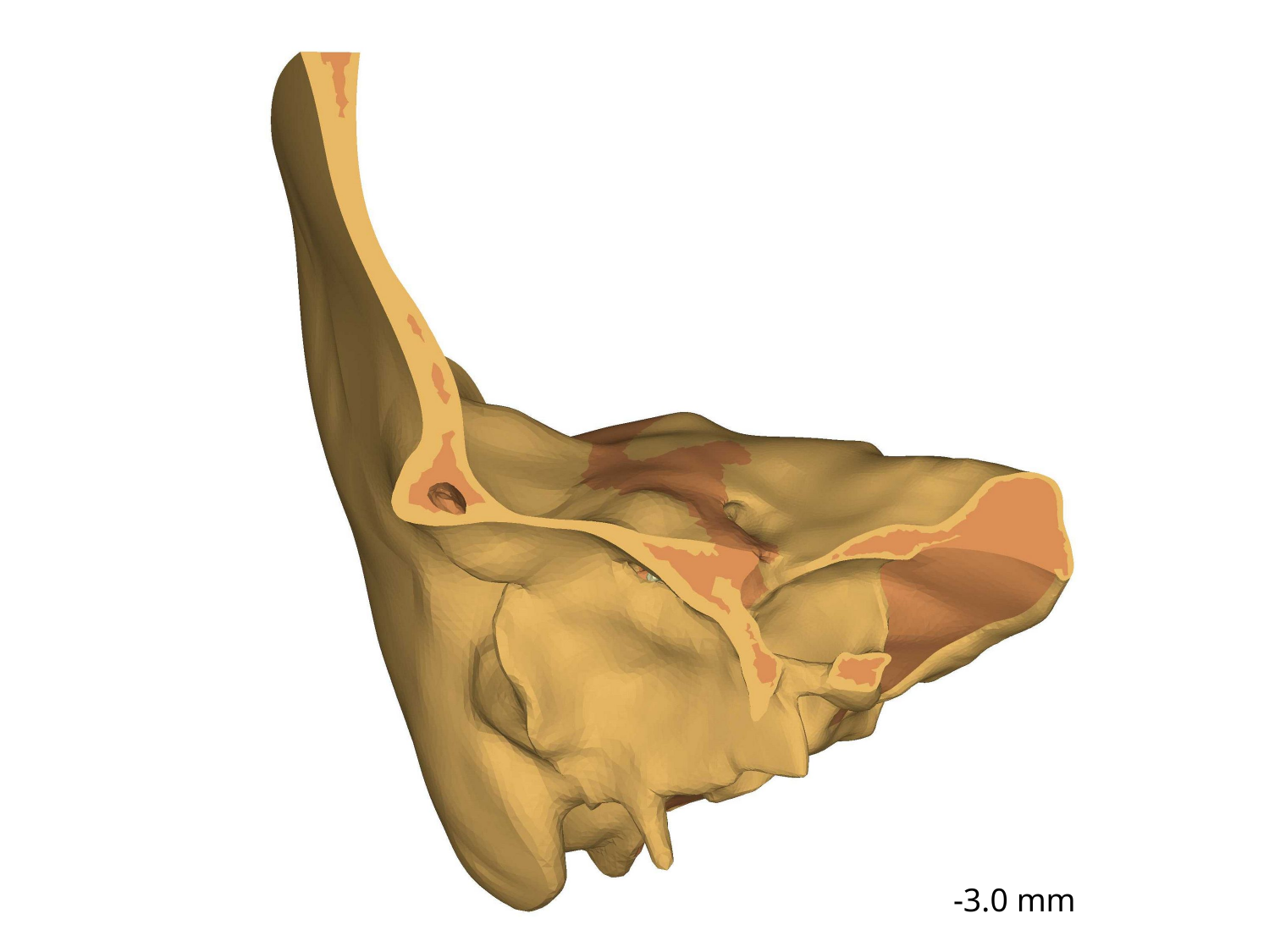

-3.0 mm

## Slide 73
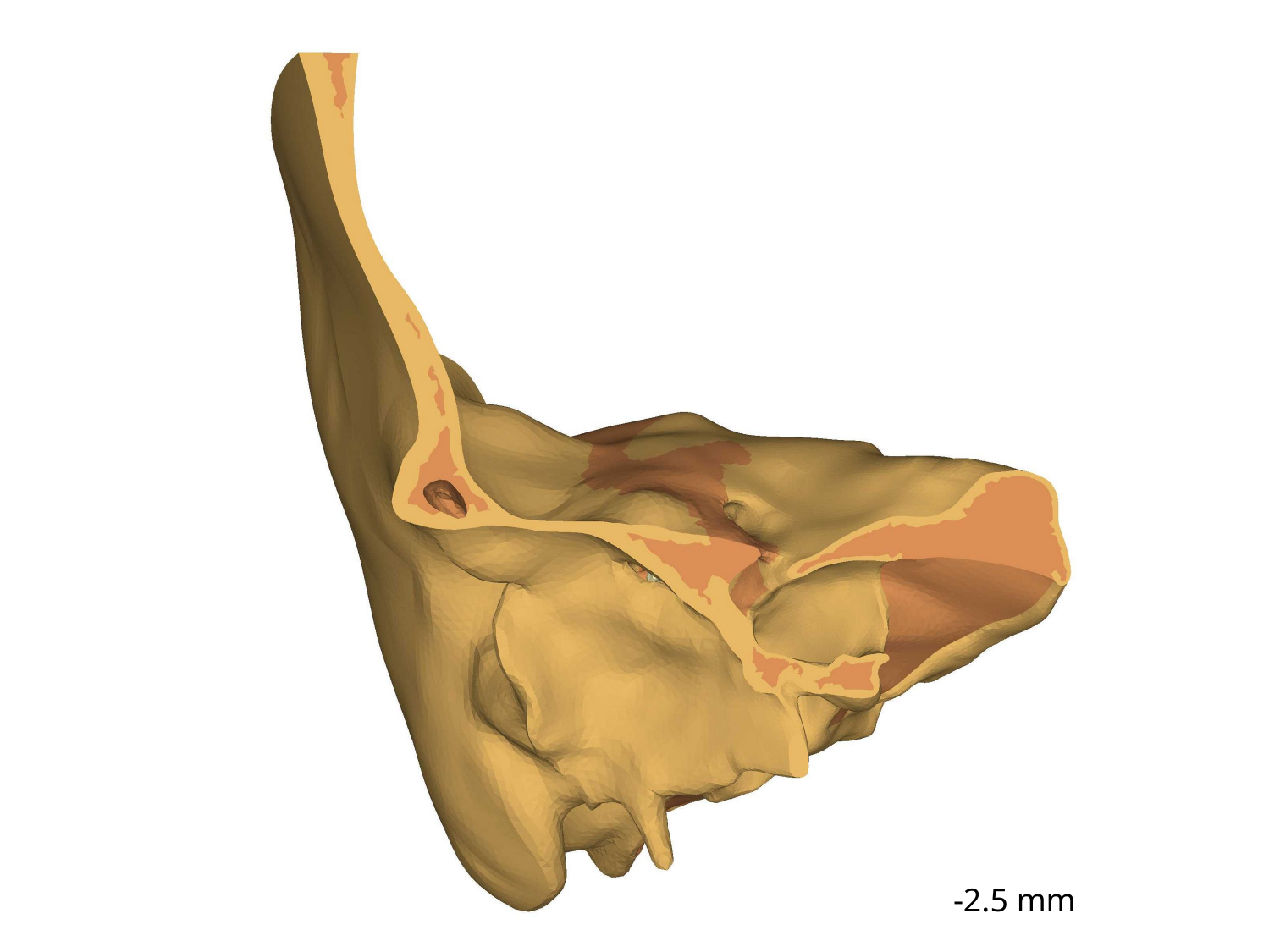

-2.5 mm

## Slide 74
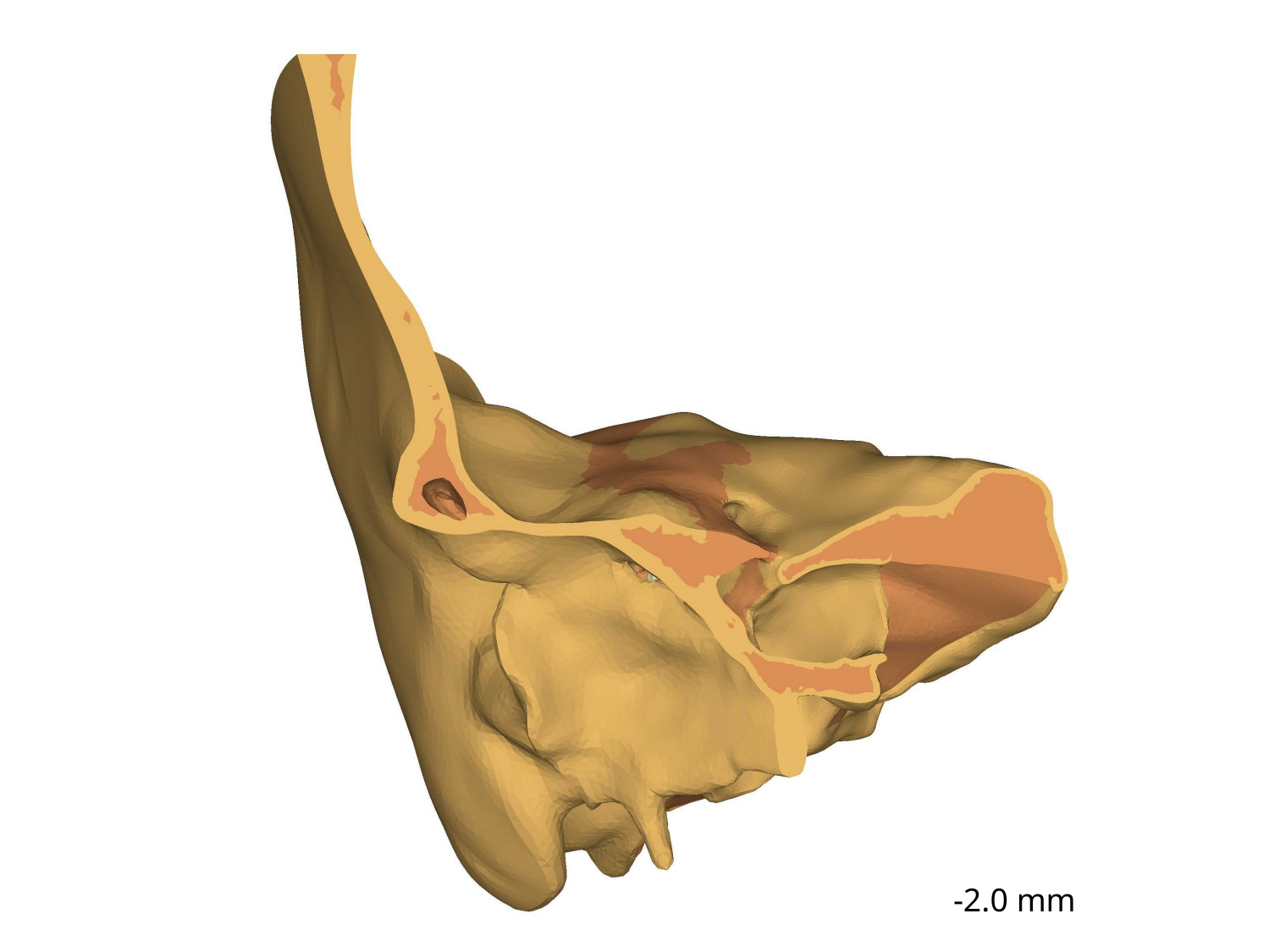

-2.0 mm

## Slide 75
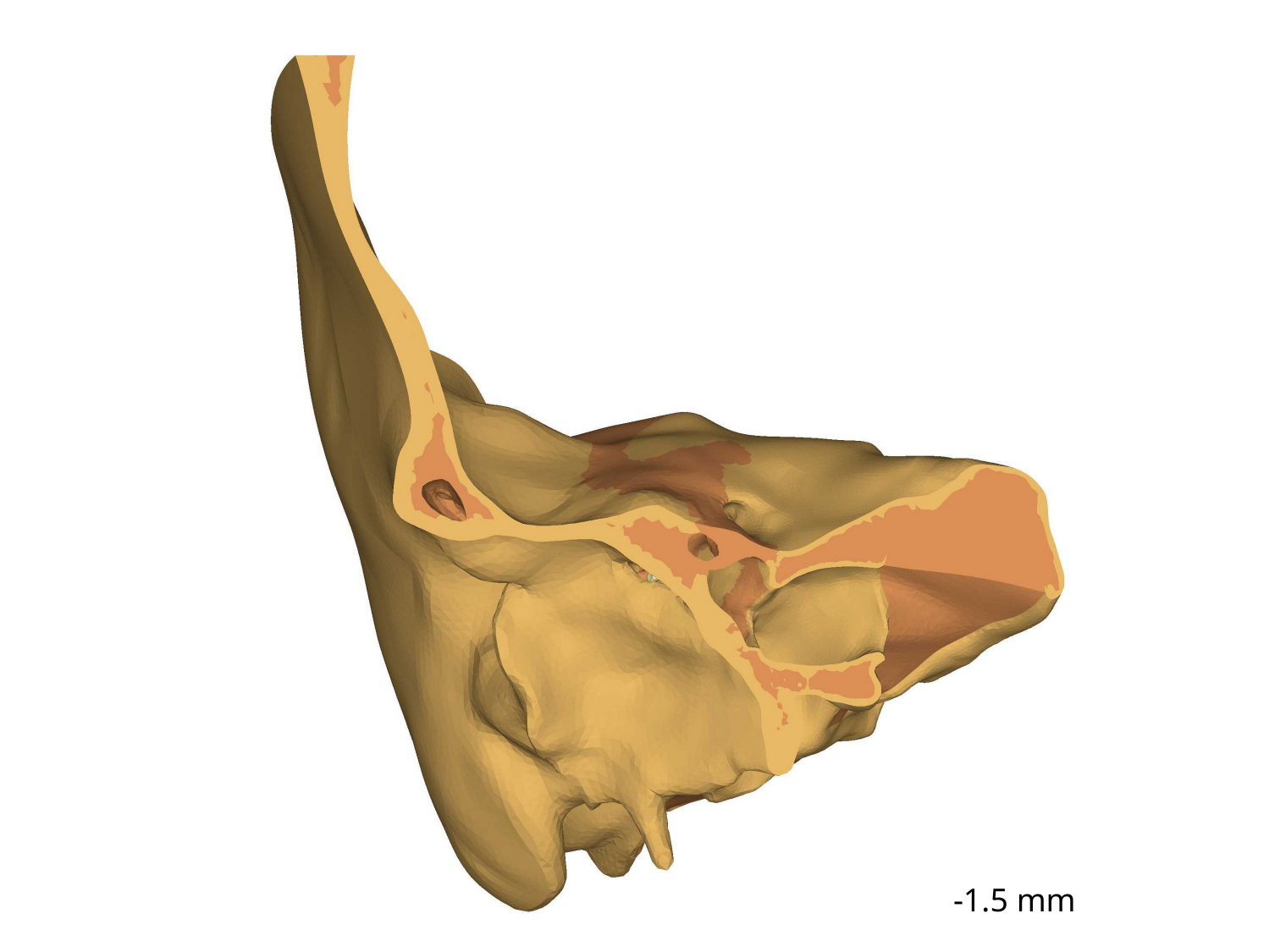

-1.5 mm

## Slide 76
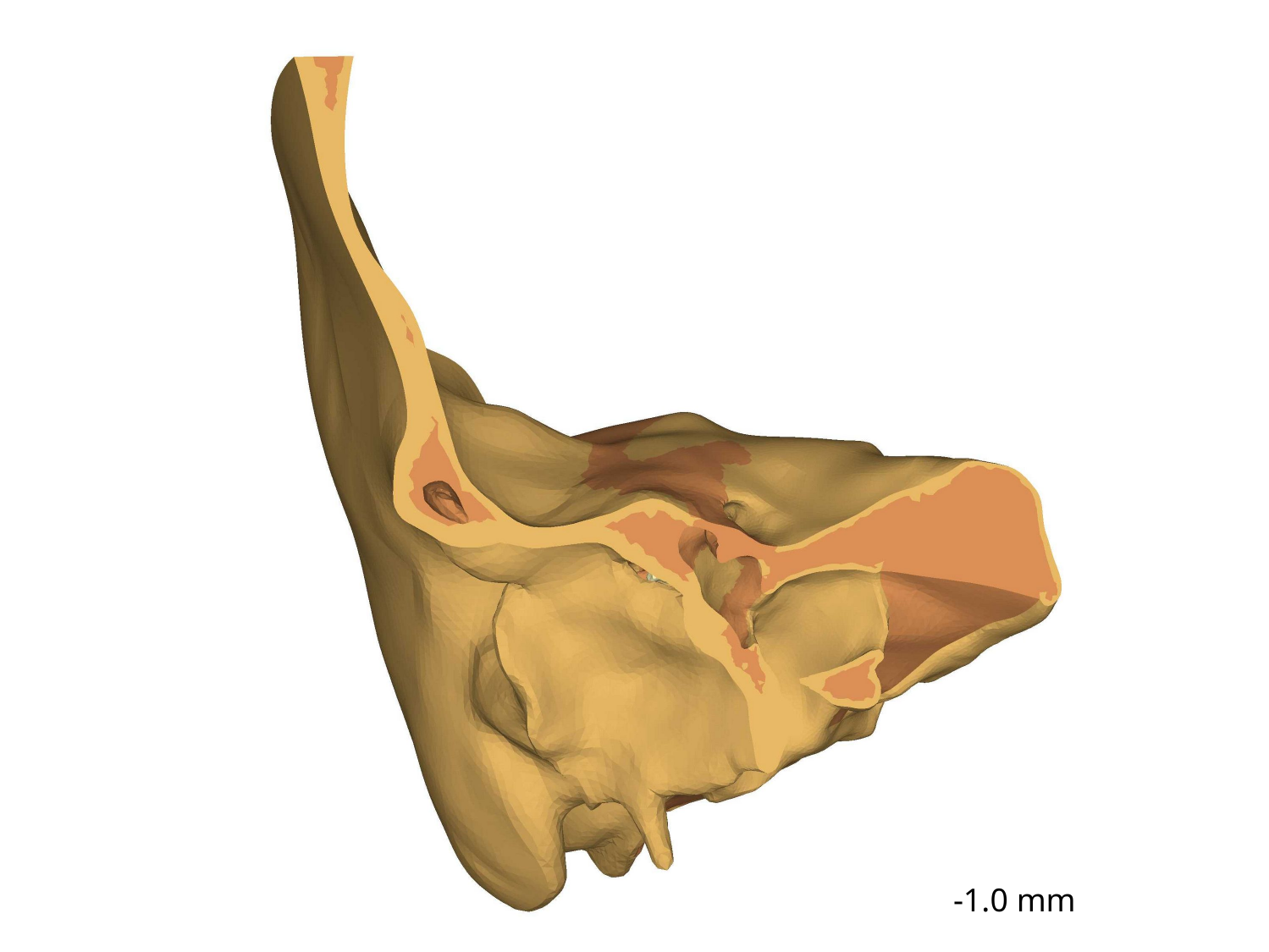

-1.0 mm

## Slide 77
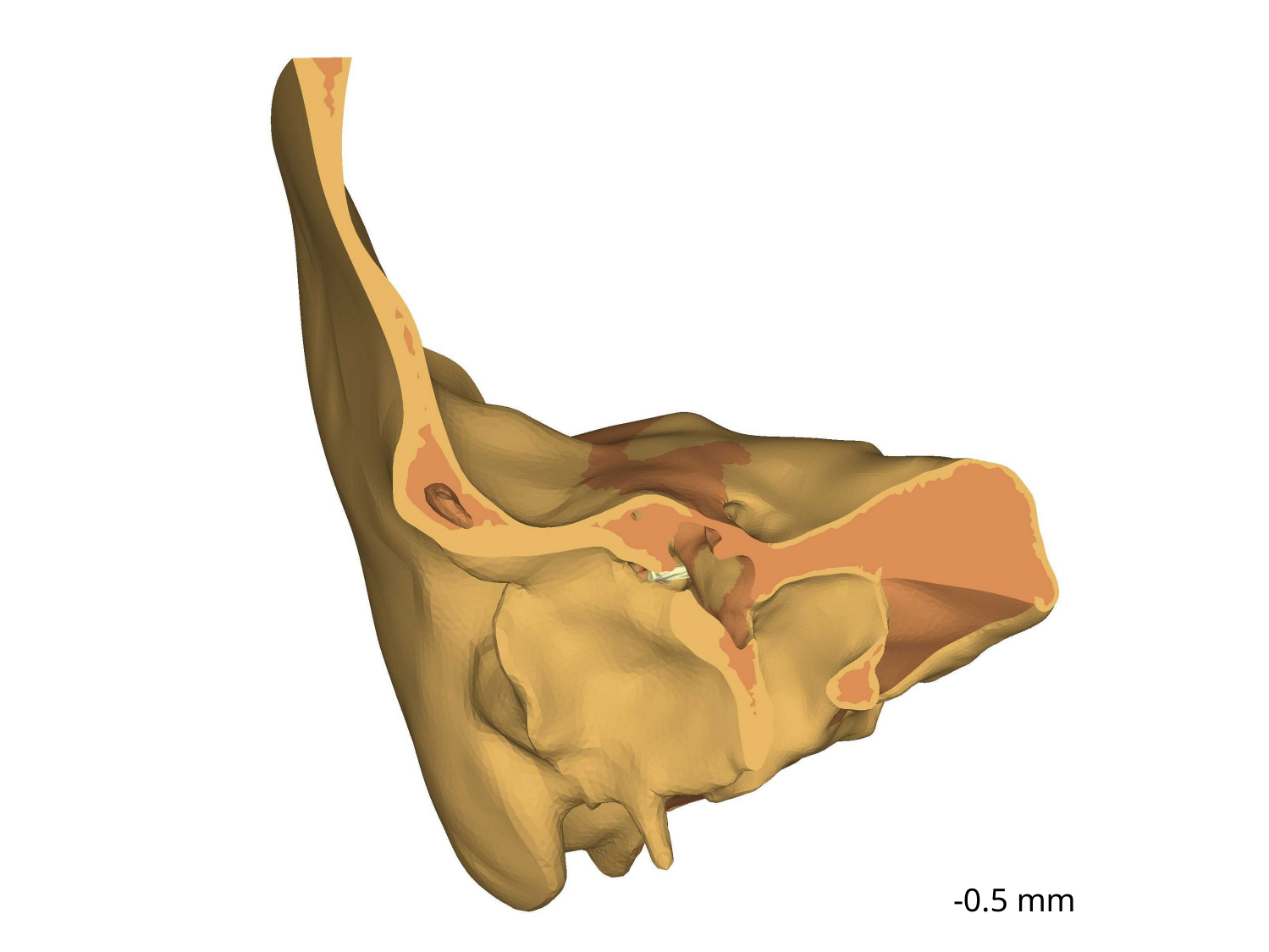

-0.5 mm

## Slide 78
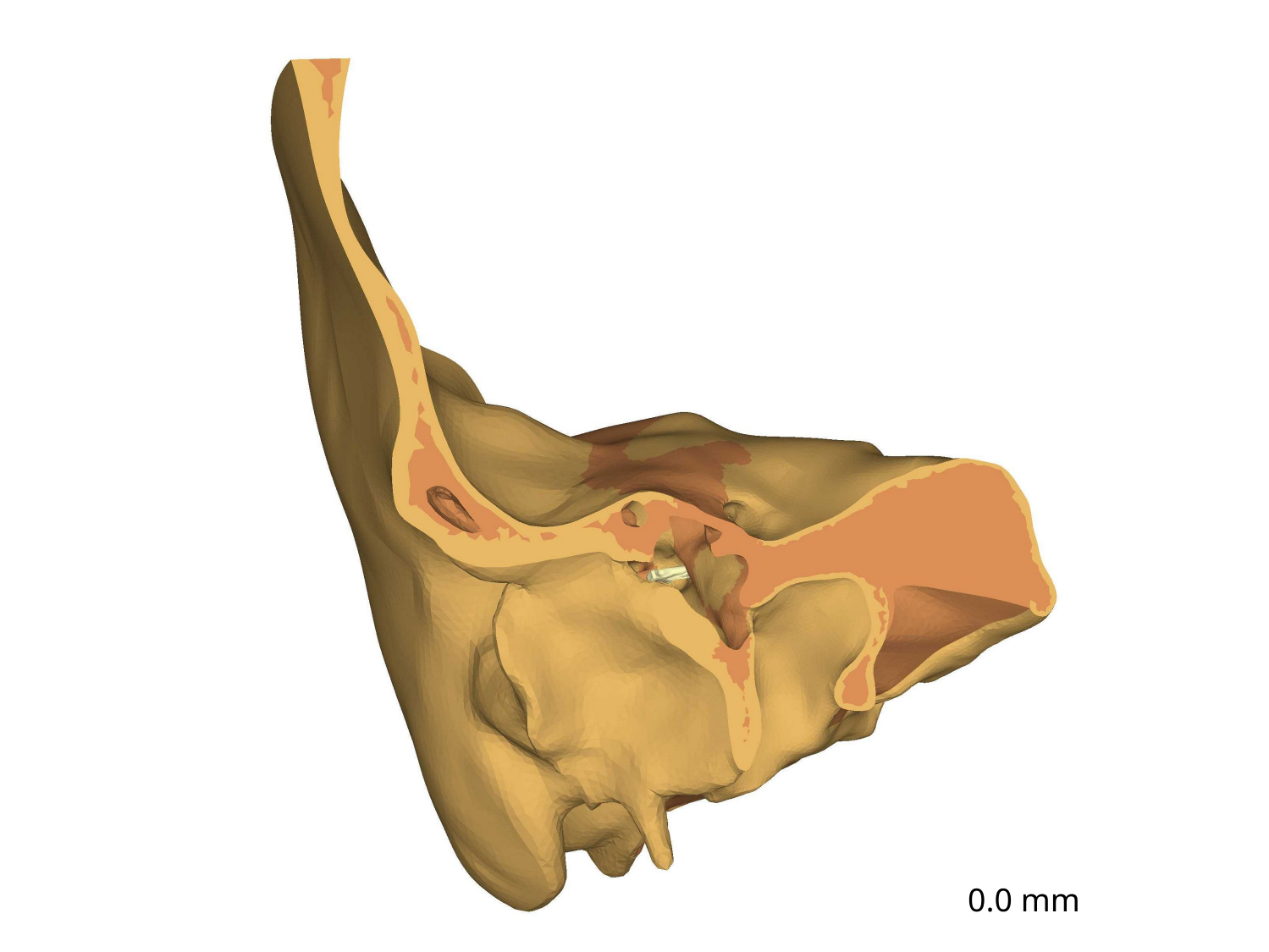

0.0 mm

## Slide 79
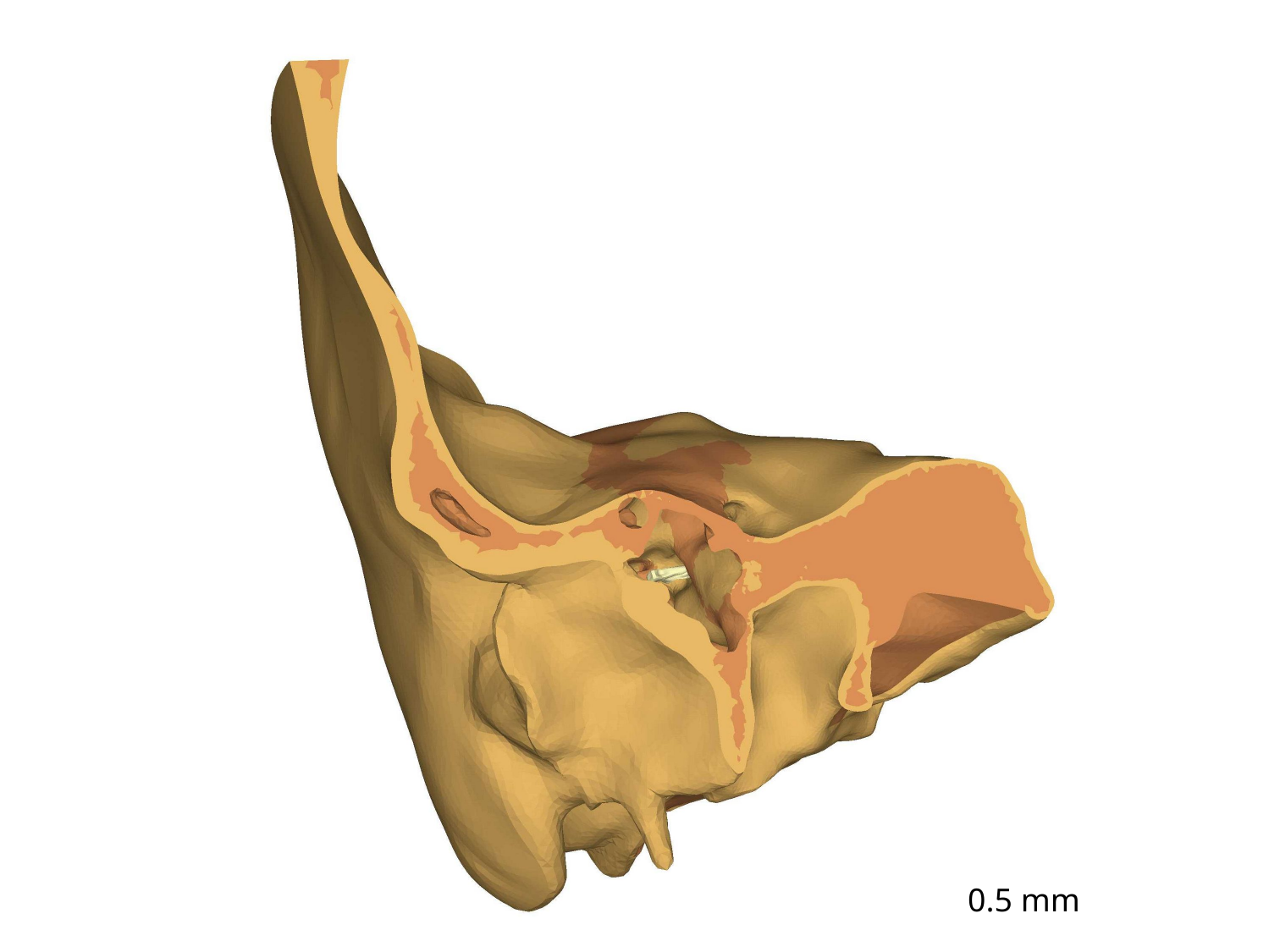

0.5 mm

## Slide 80
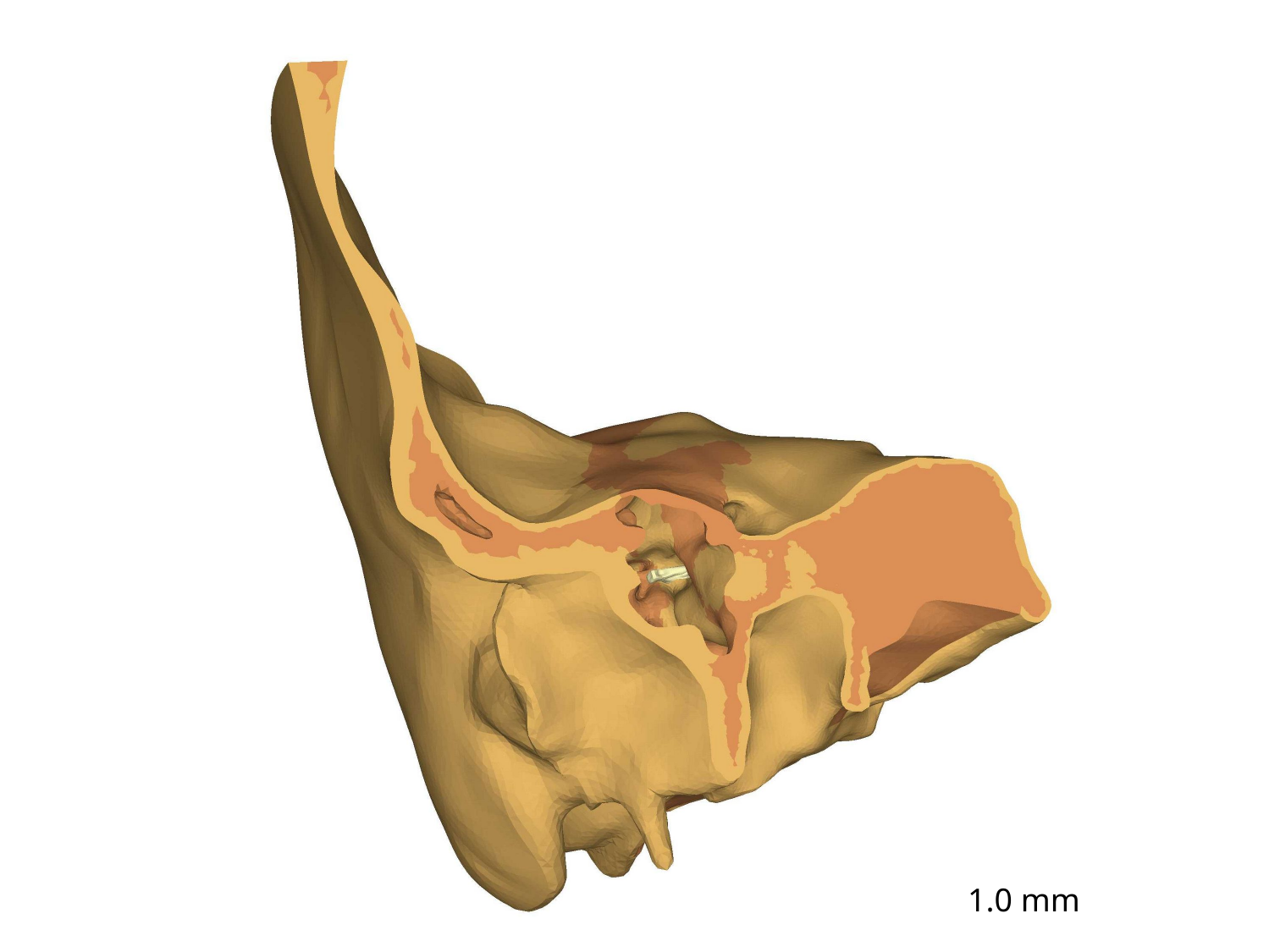

1.0 mm

## Slide 81
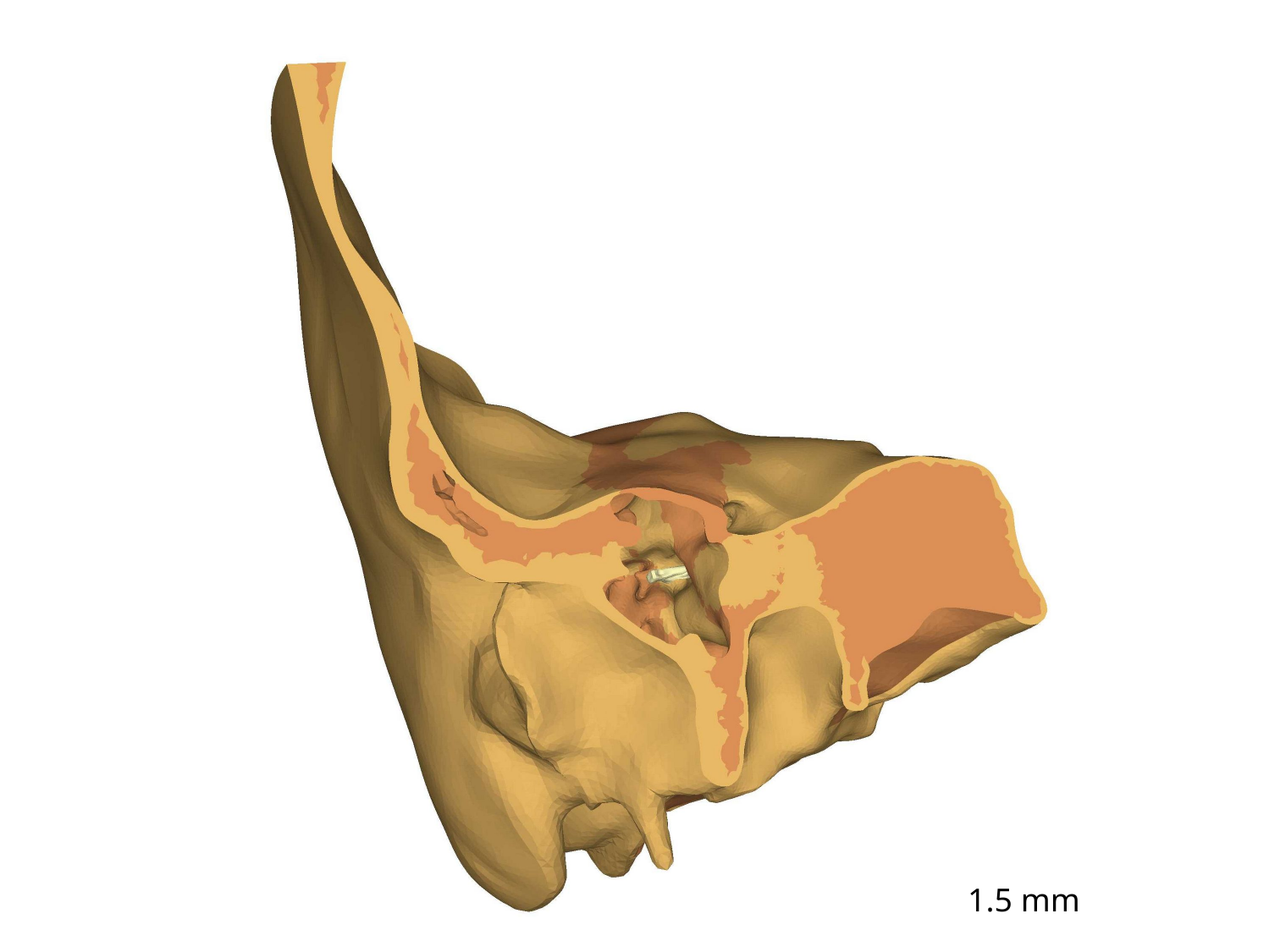

1.5 mm

## Slide 82
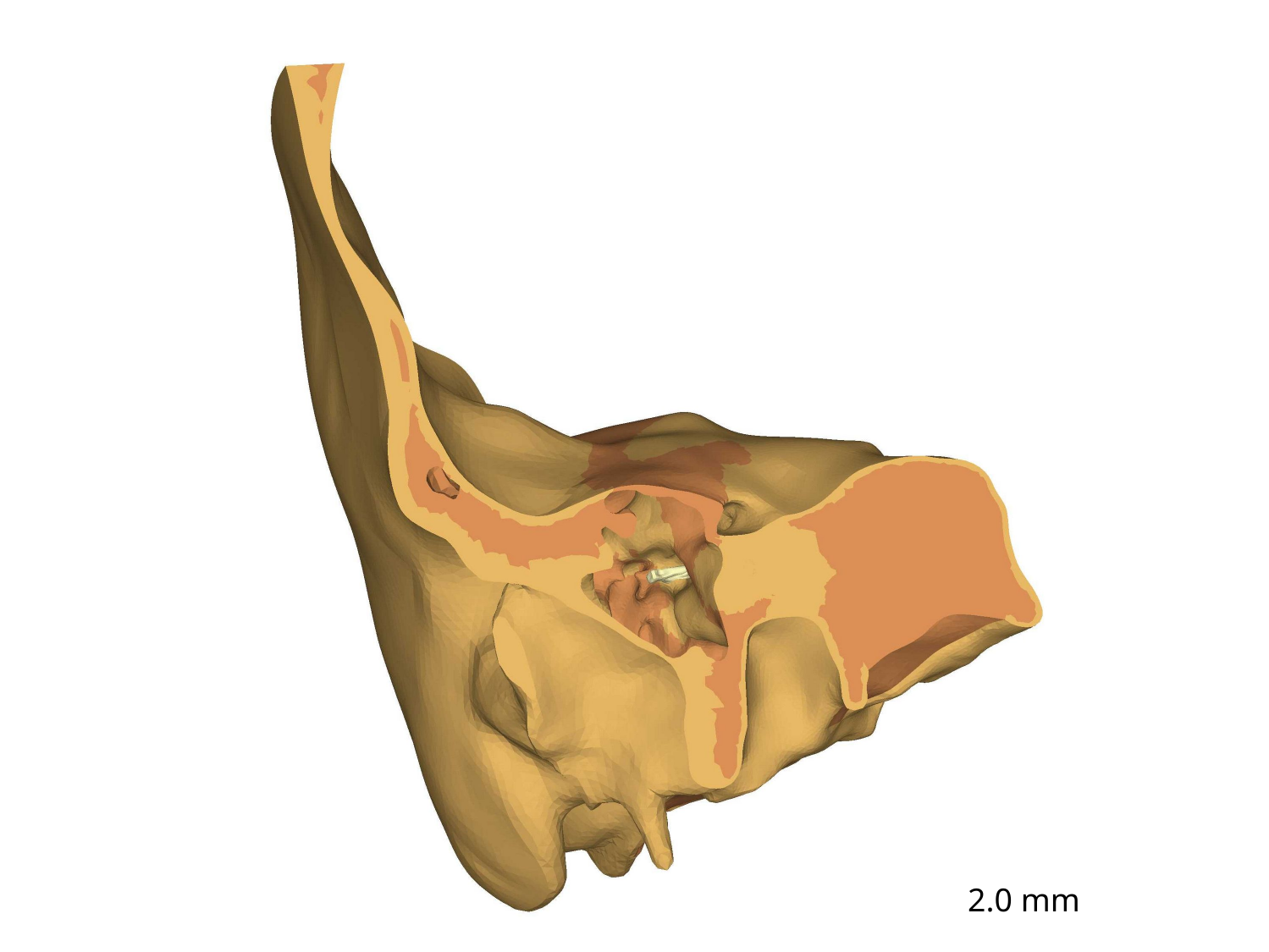

2.0 mm

## Slide 83
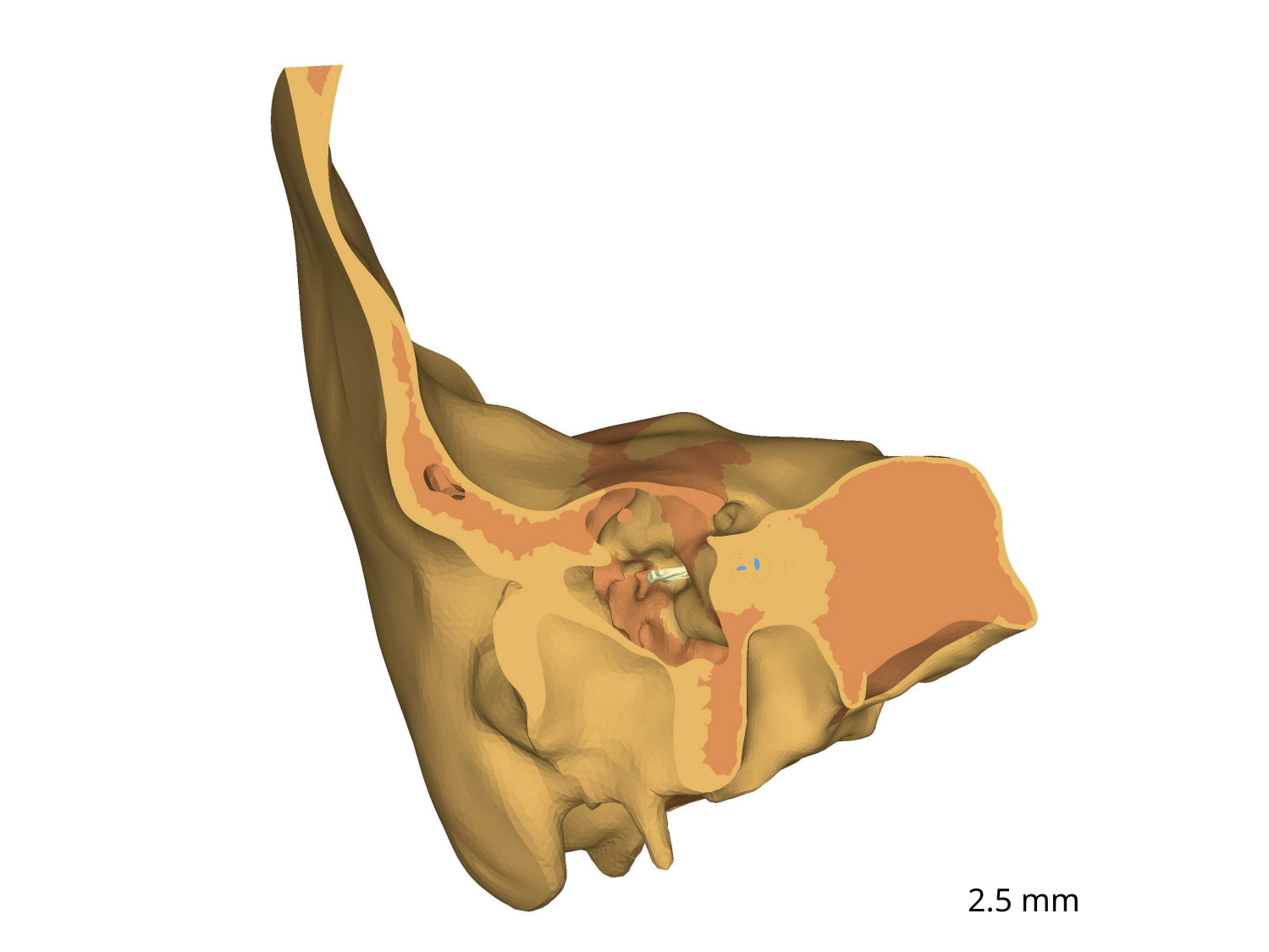

2.5 mm

## Slide 84
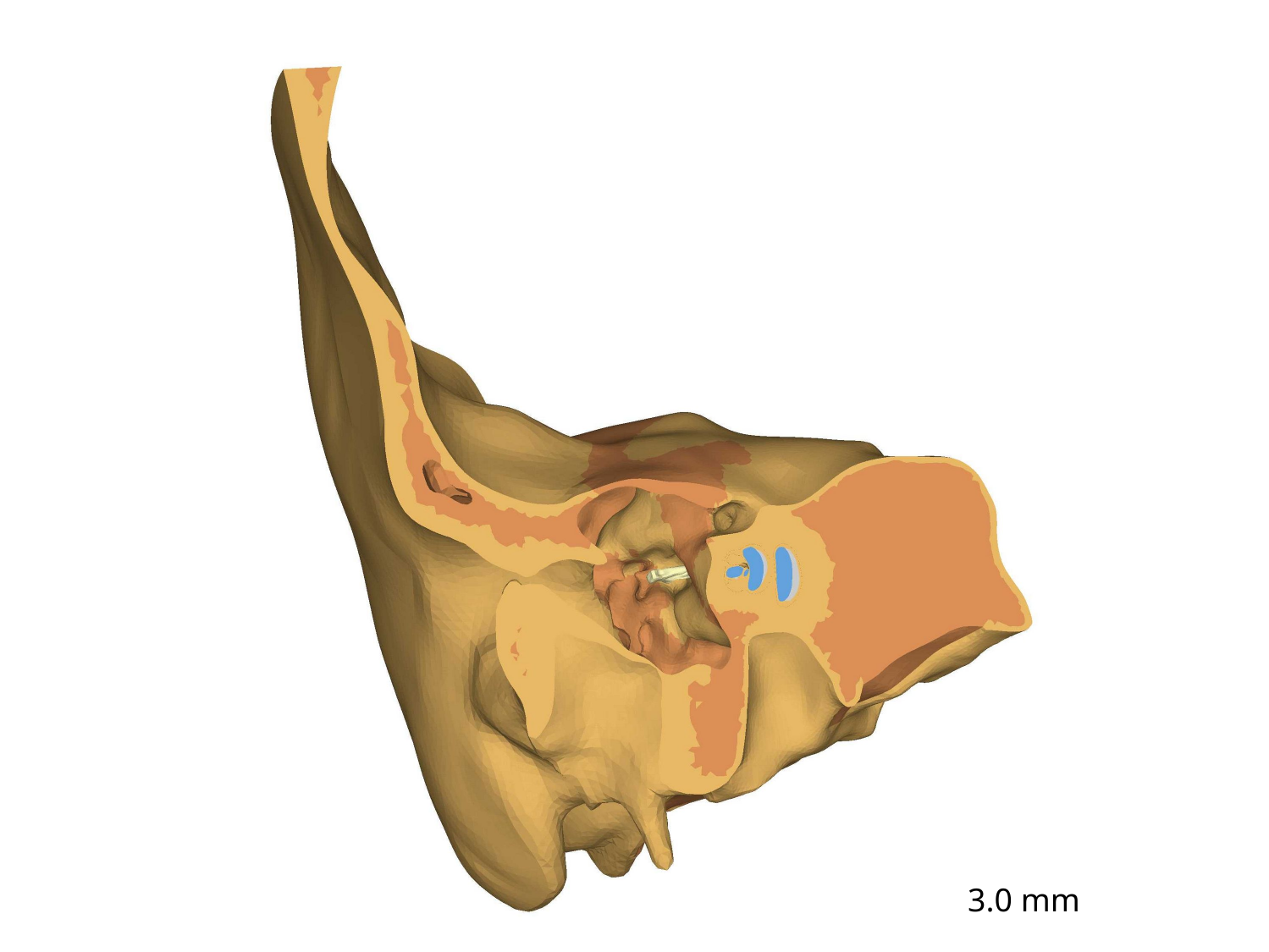

3.0 mm

## Slide 85
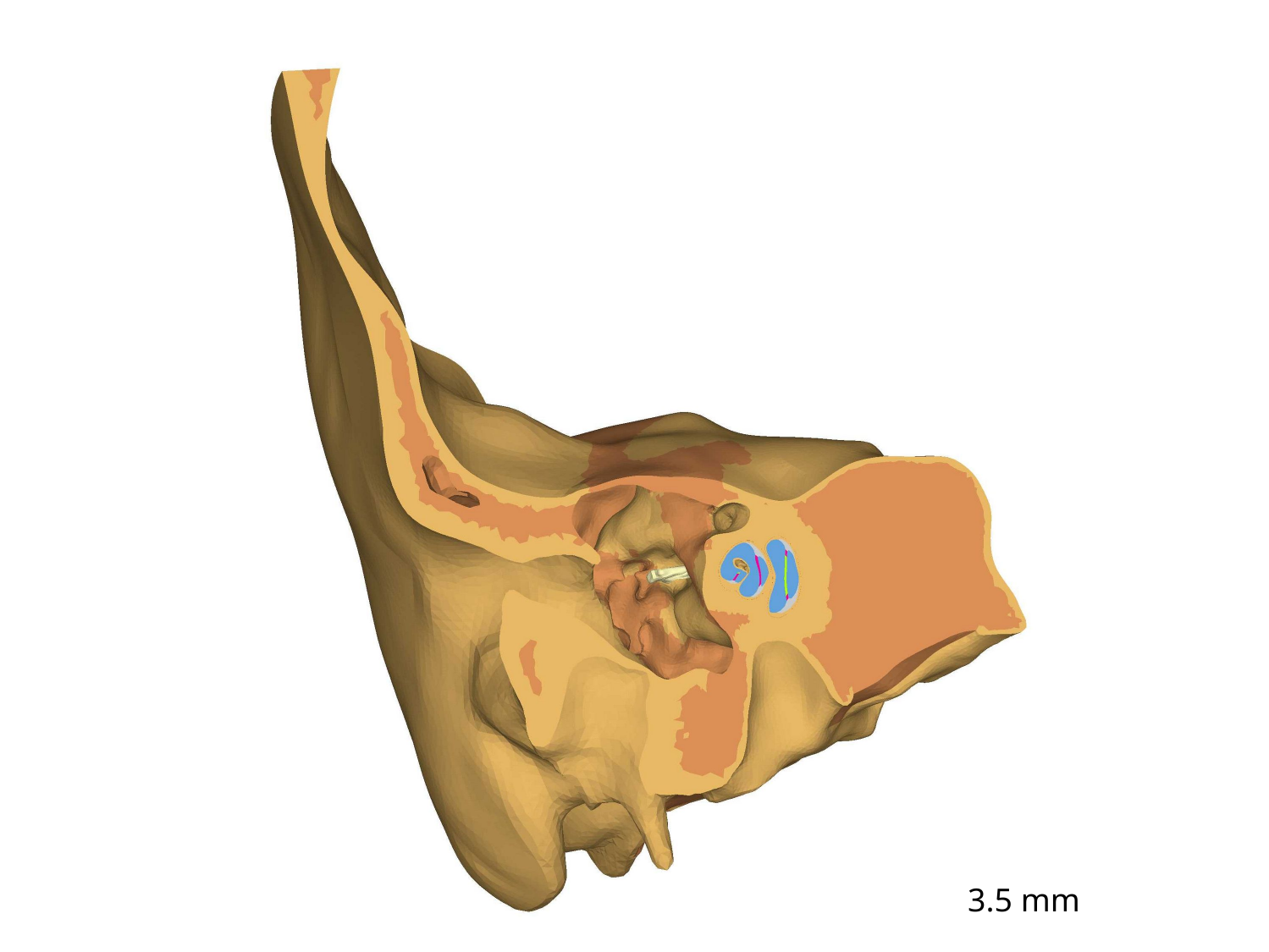

3.5 mm

## Slide 86
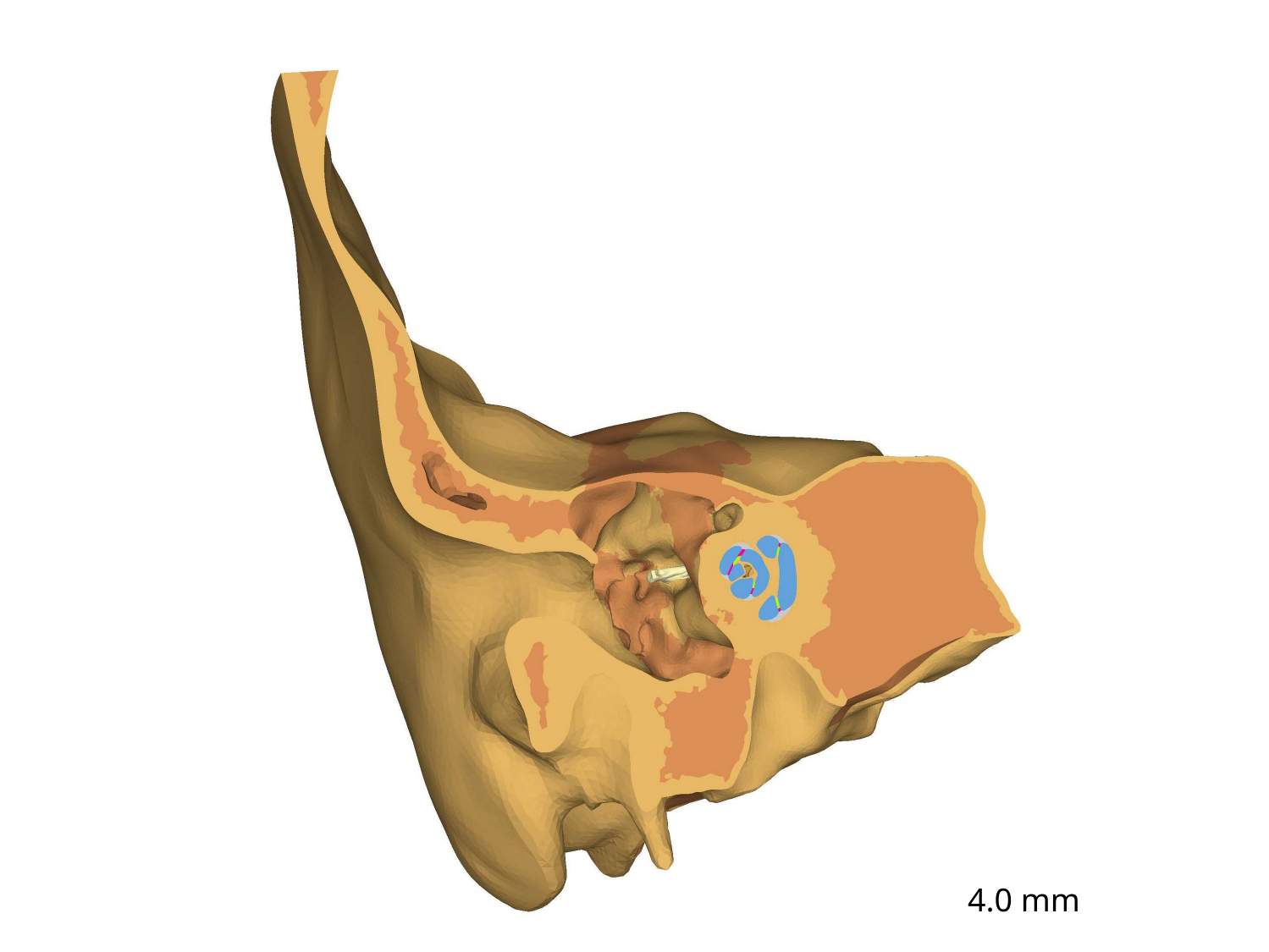

4.0 mm

## Slide 87
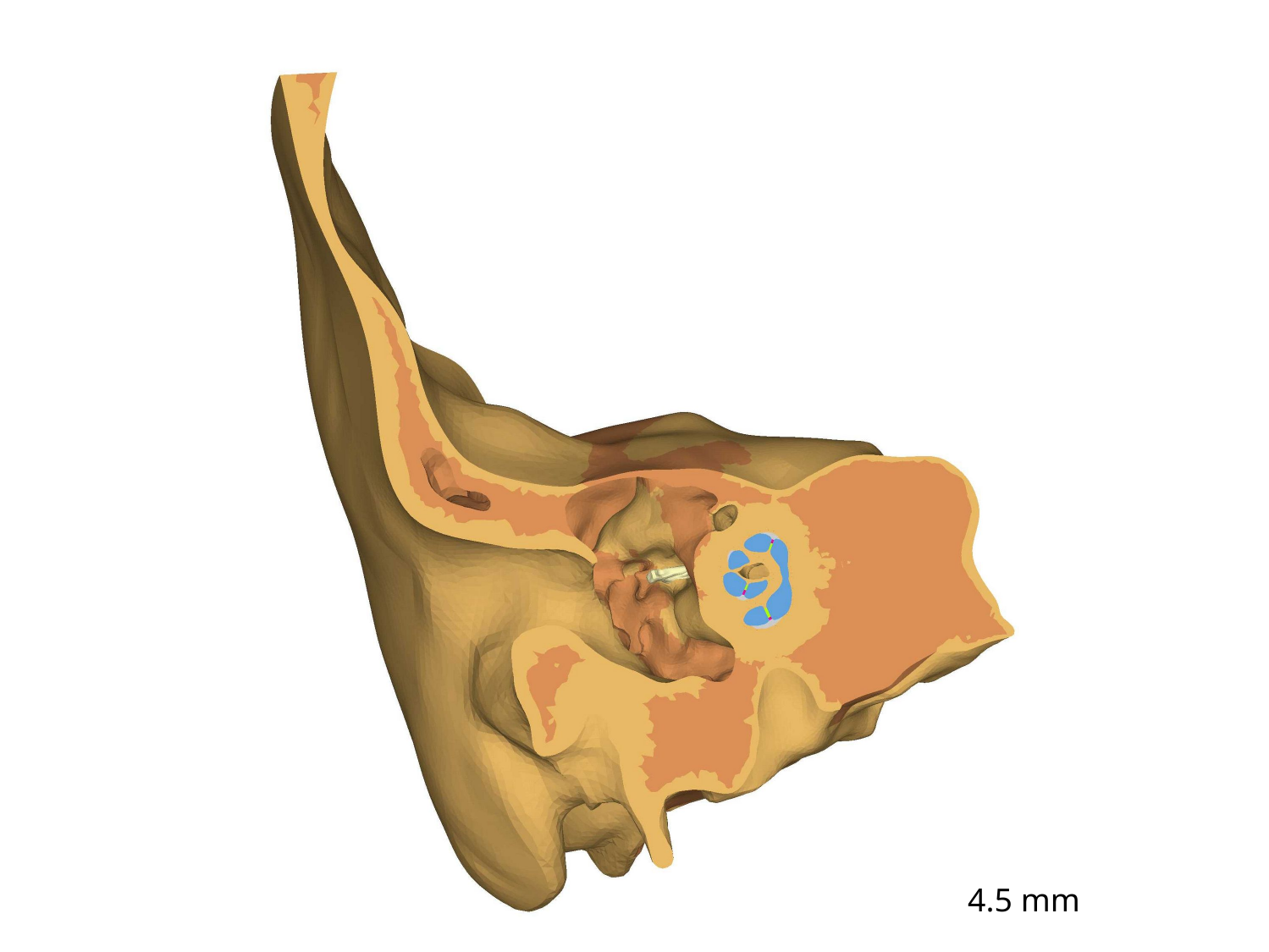

4.5 mm

## Slide 88
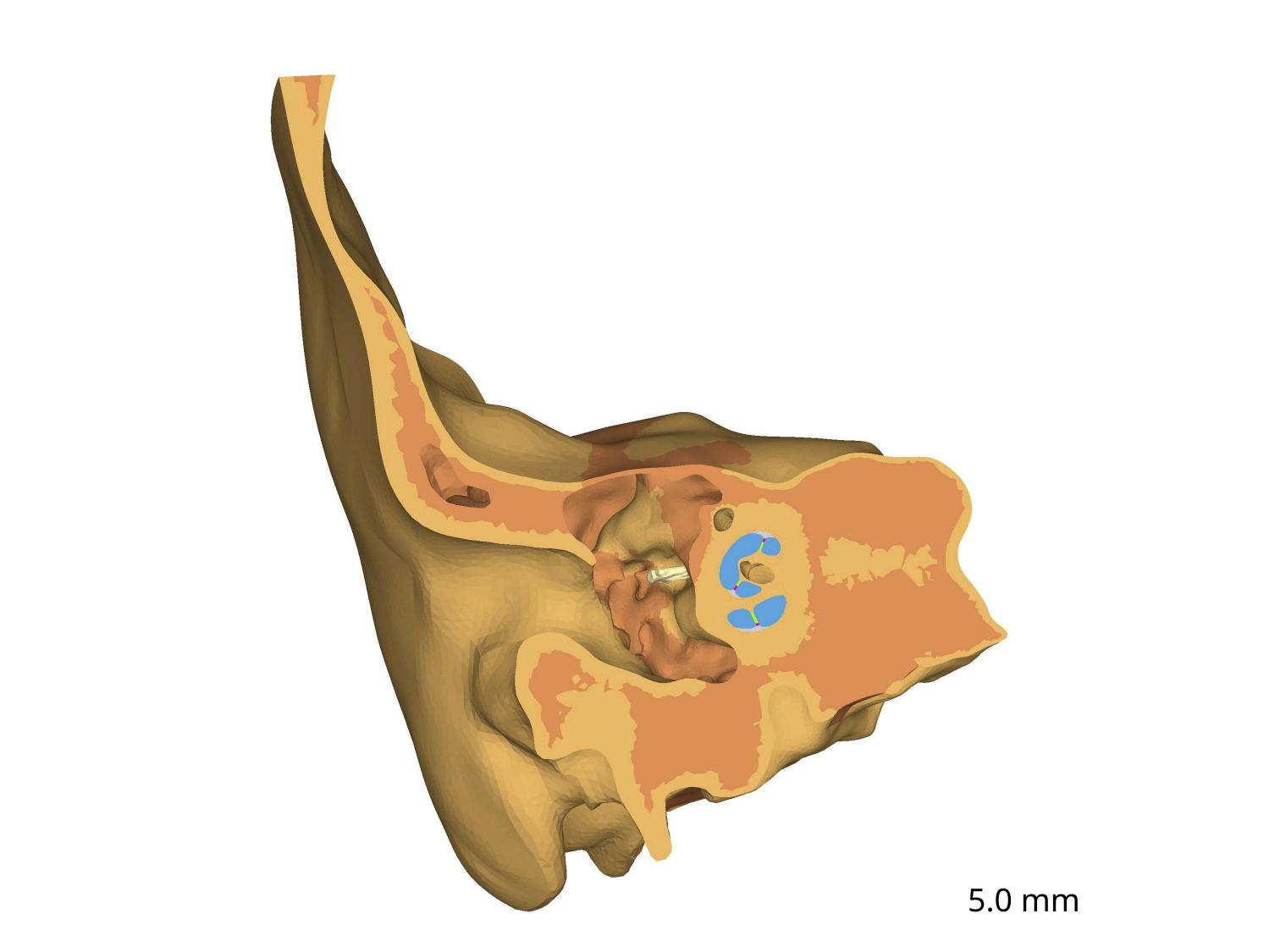

5.0 mm

## Slide 89
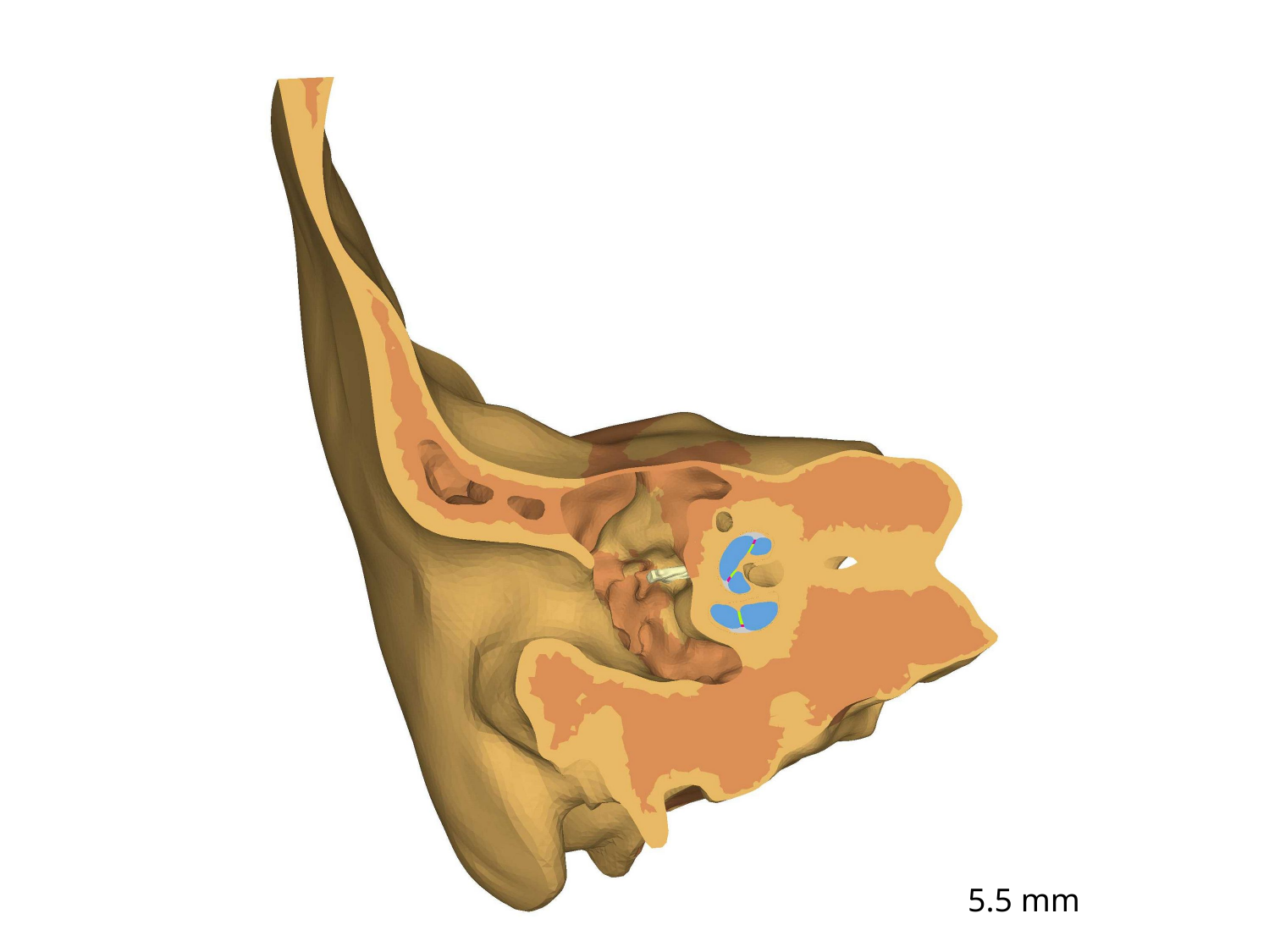

5.5 mm

## Slide 90
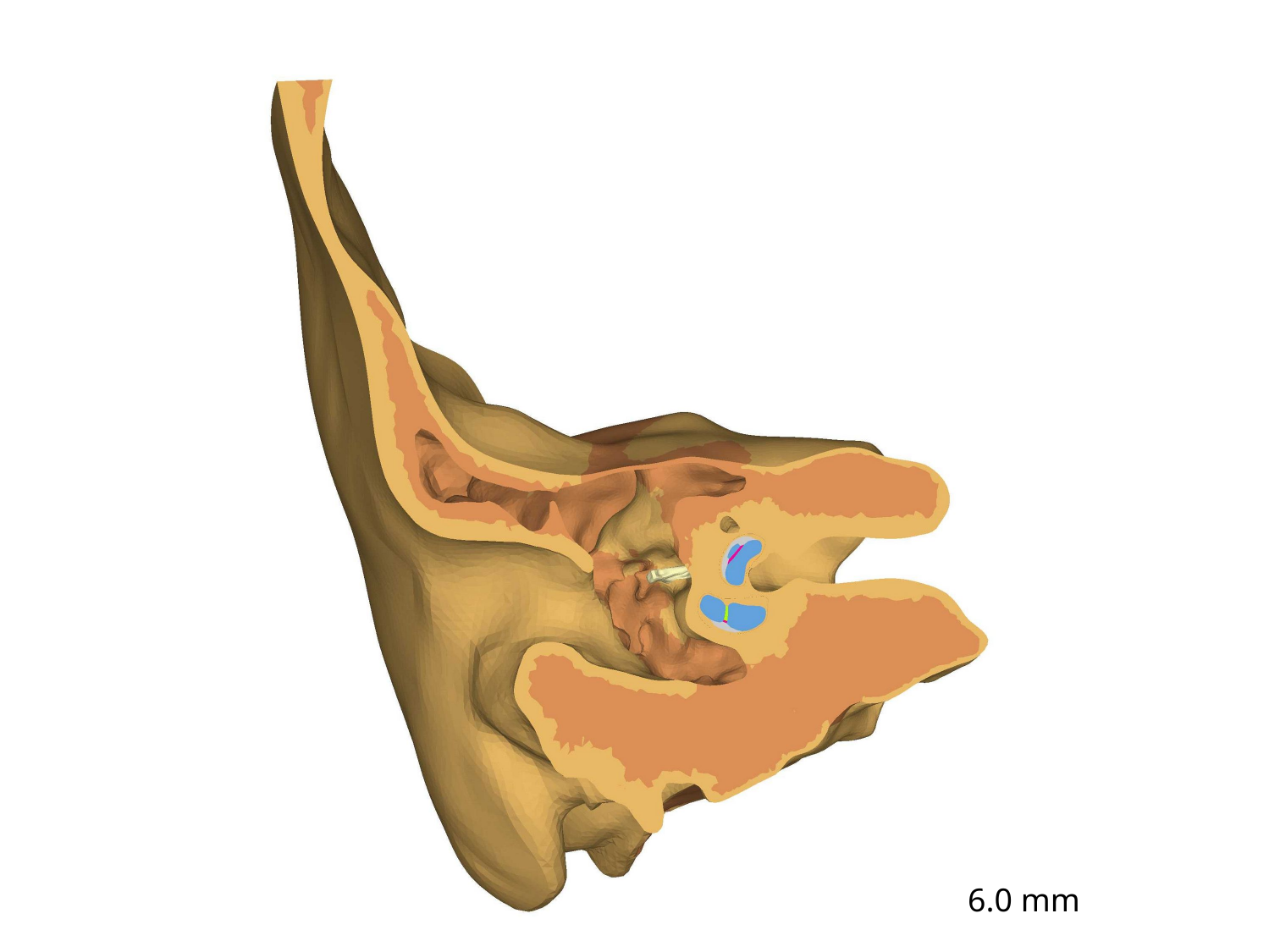

6.0 mm

## Slide 91
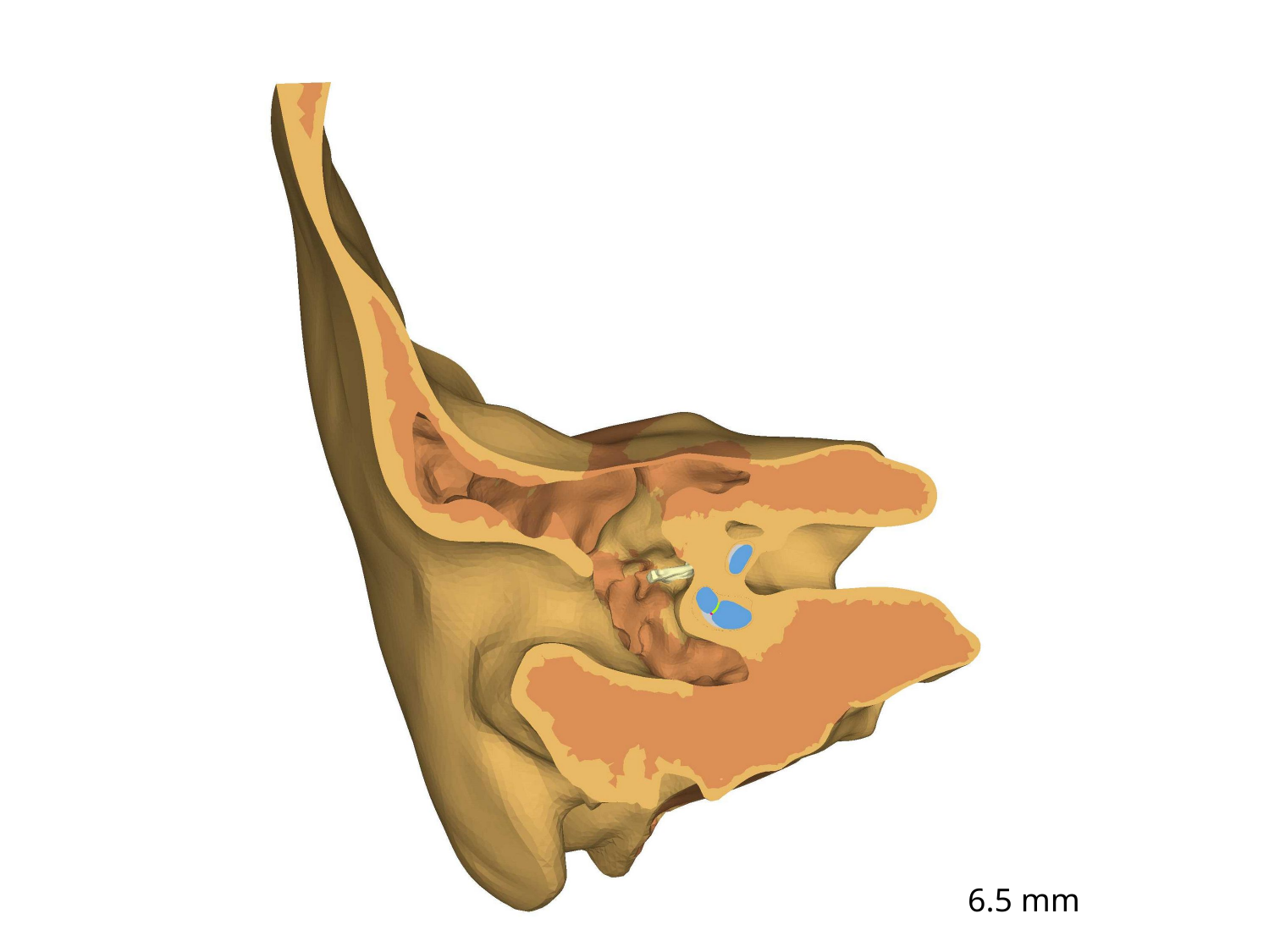

6.5 mm

## Slide 92
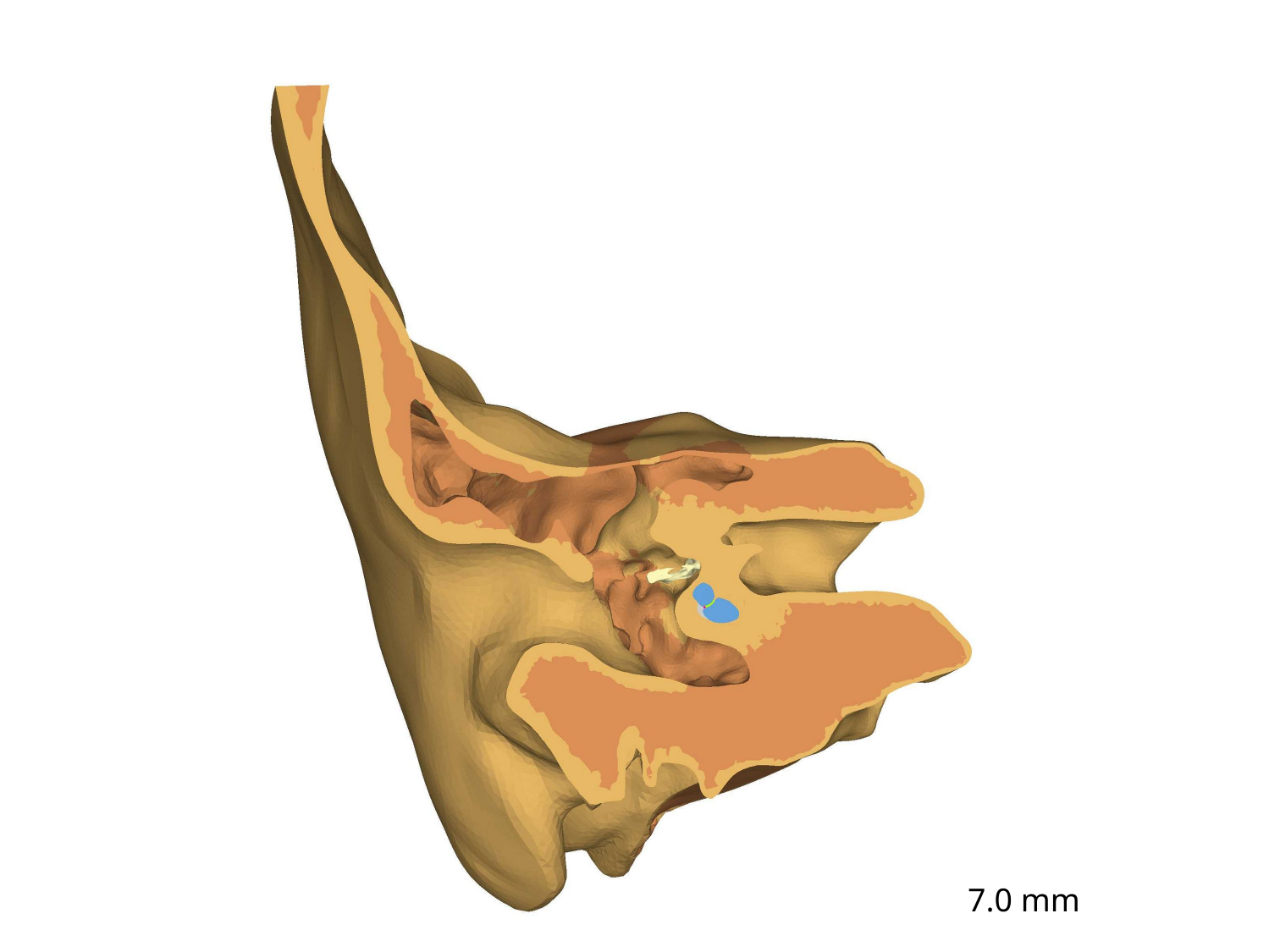

7.0 mm

## Slide 93
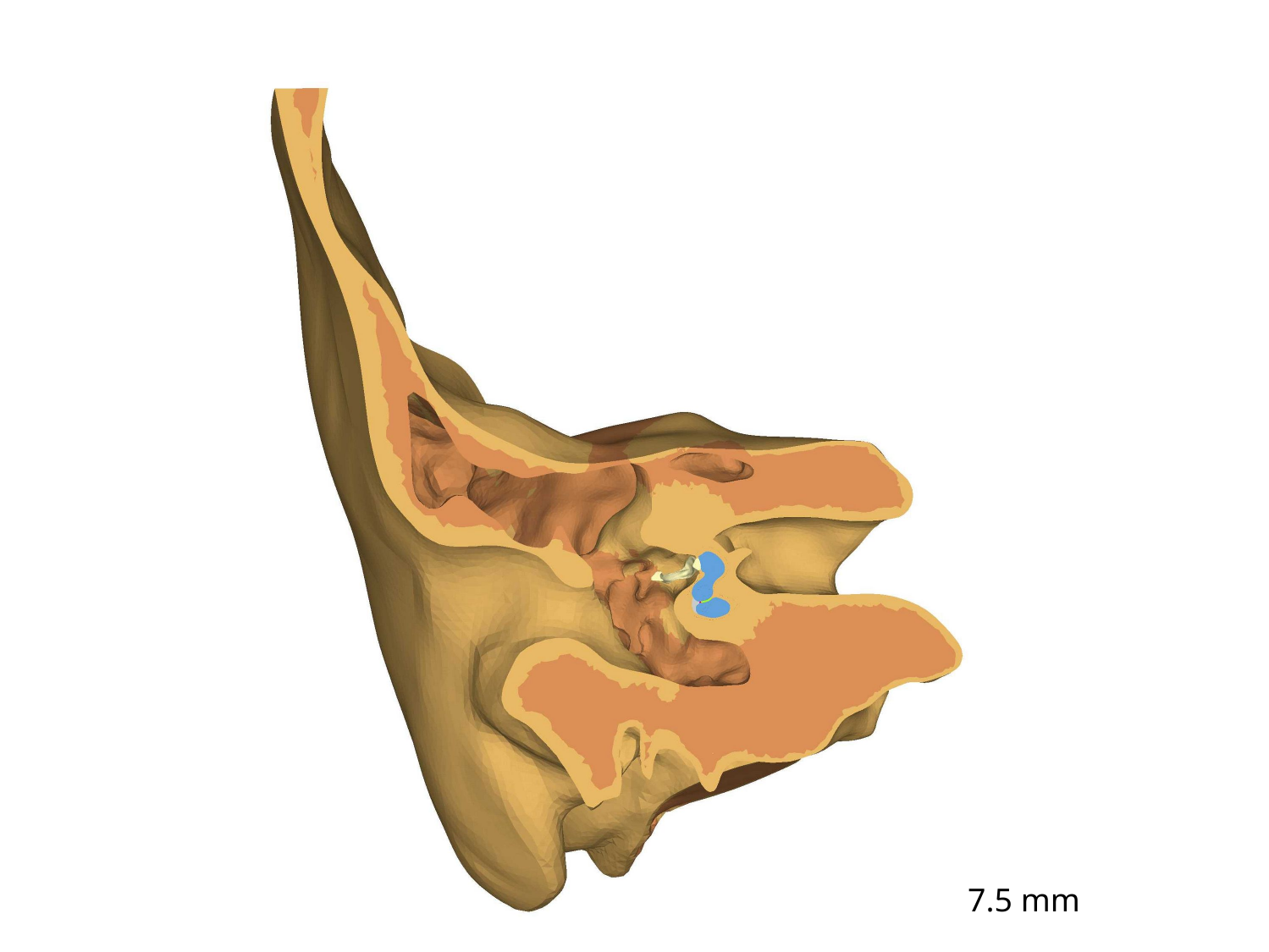

7.5 mm

## Slide 94
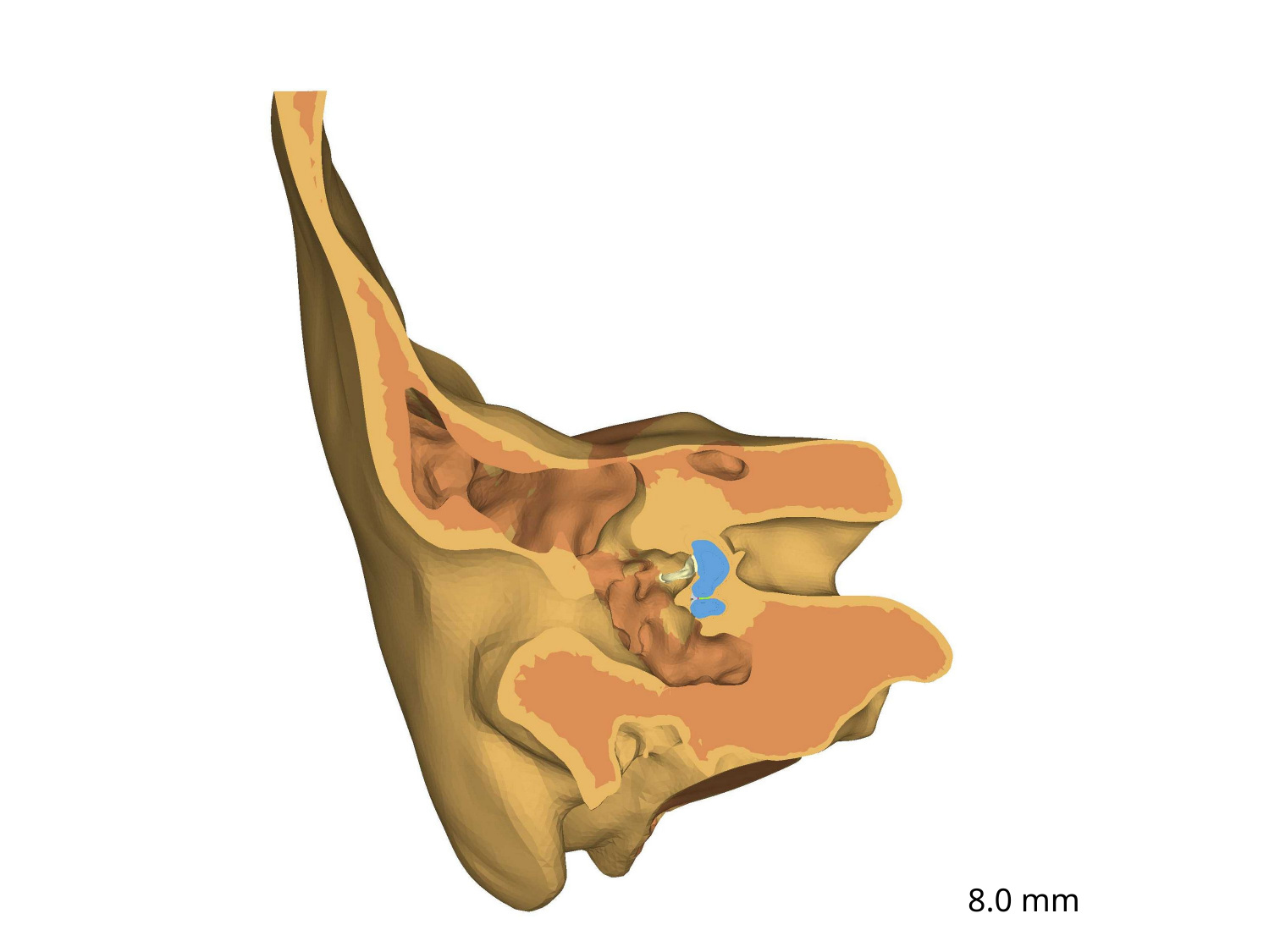

8.0 mm

## Slide 95
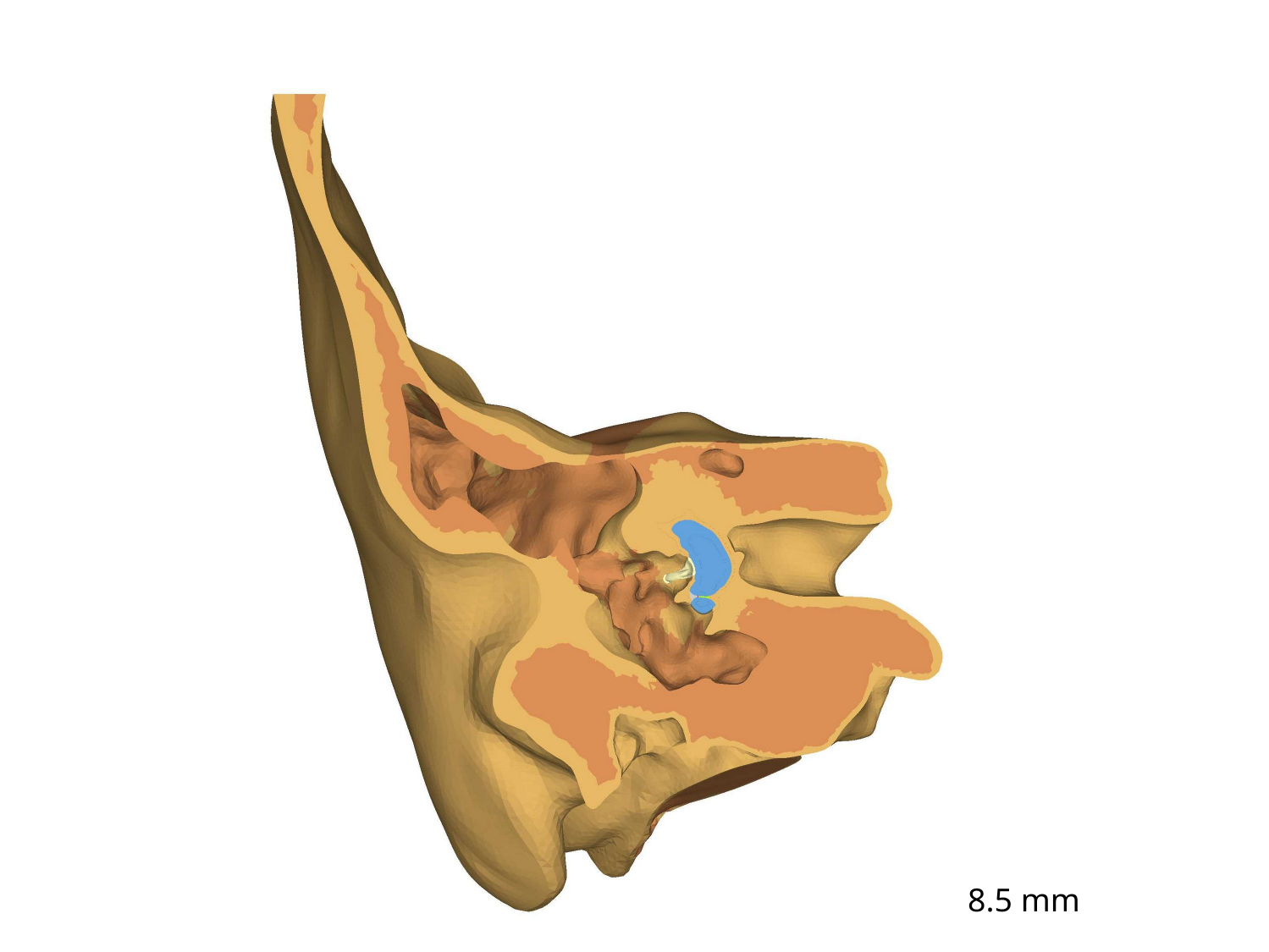

8.5 mm

## Slide 96
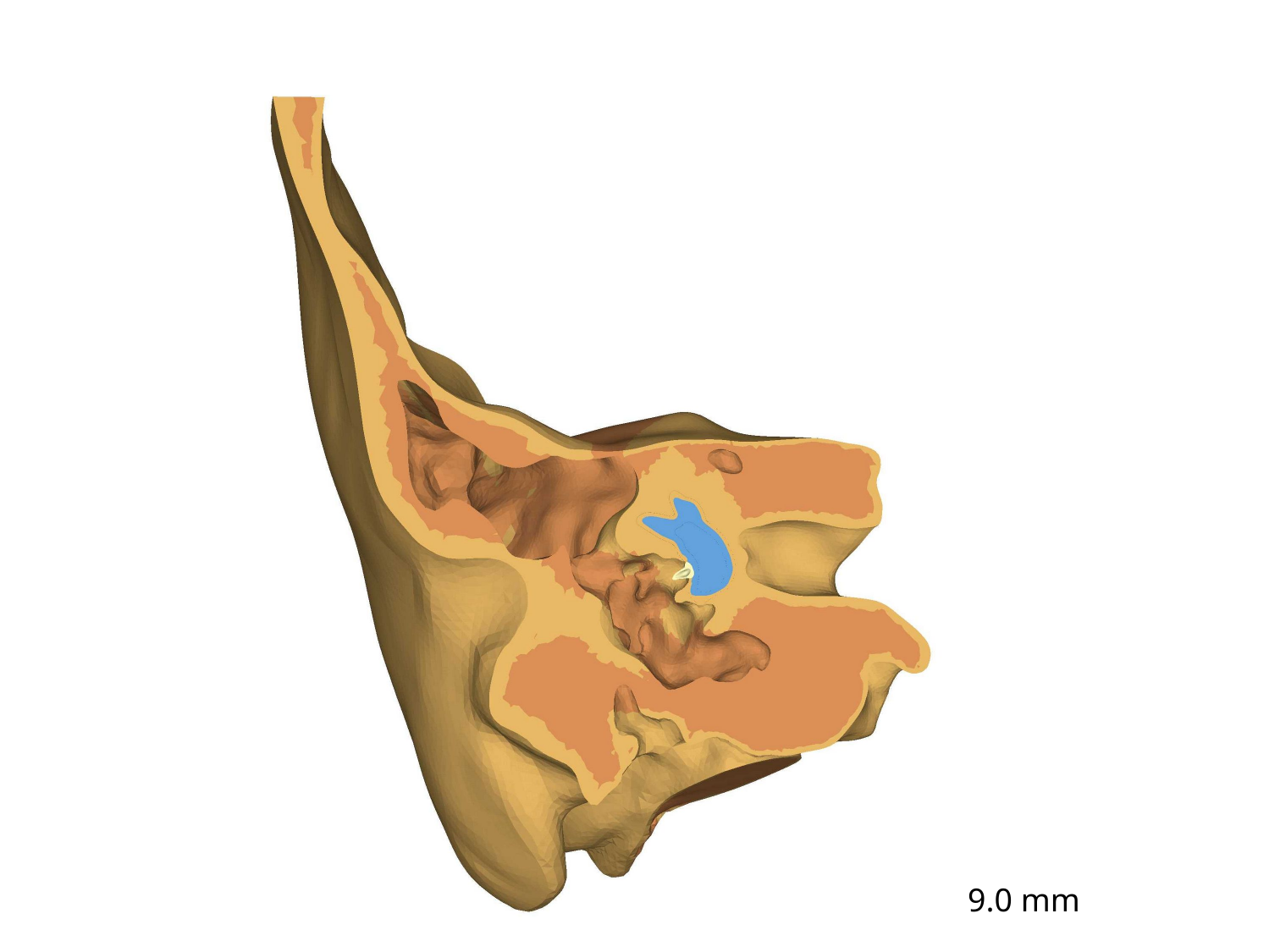

9.0 mm

## Slide 97
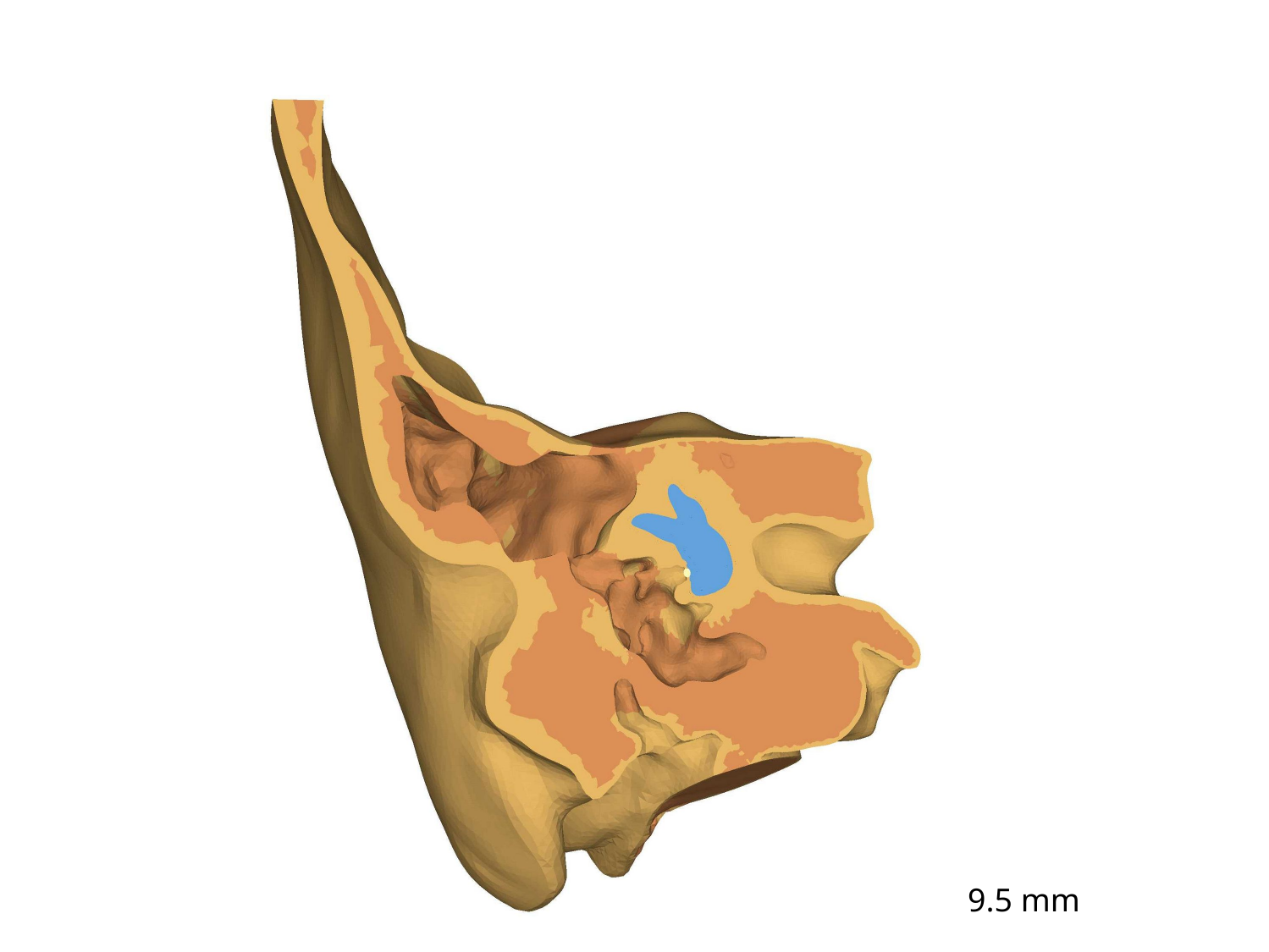

9.5 mm

## Slide 98
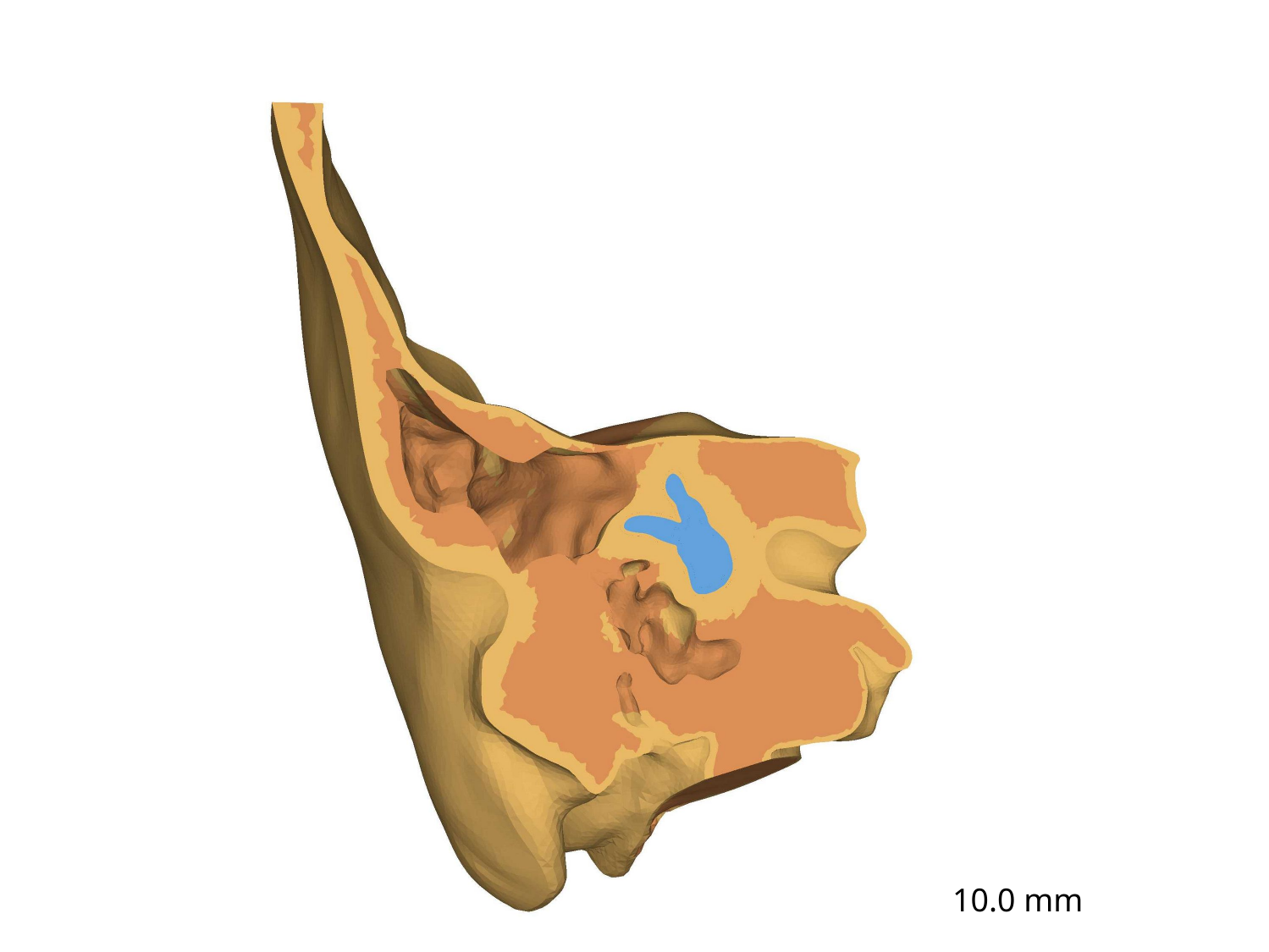

10.0 mm

## Slide 99
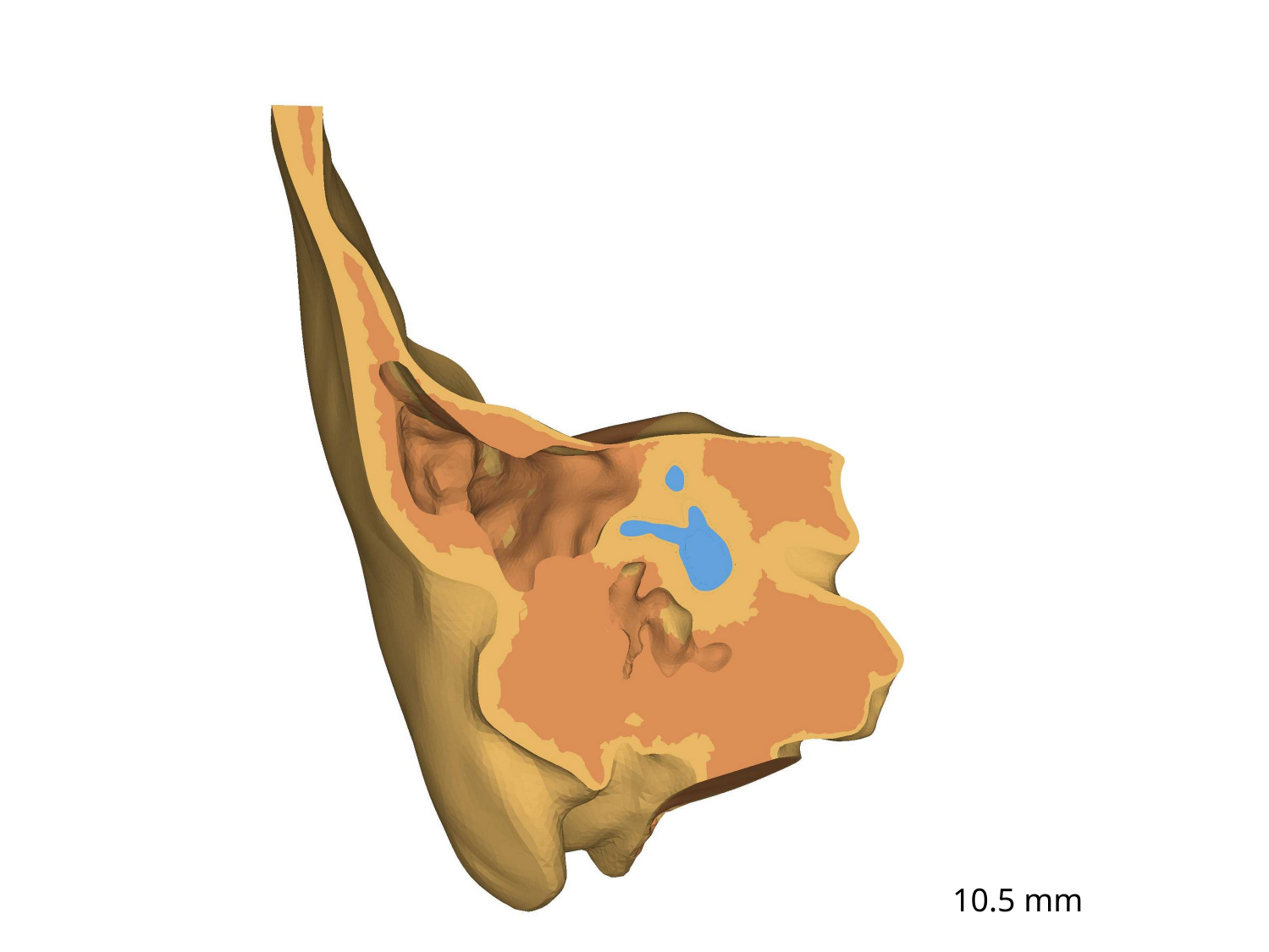

10.5 mm

## Slide 100
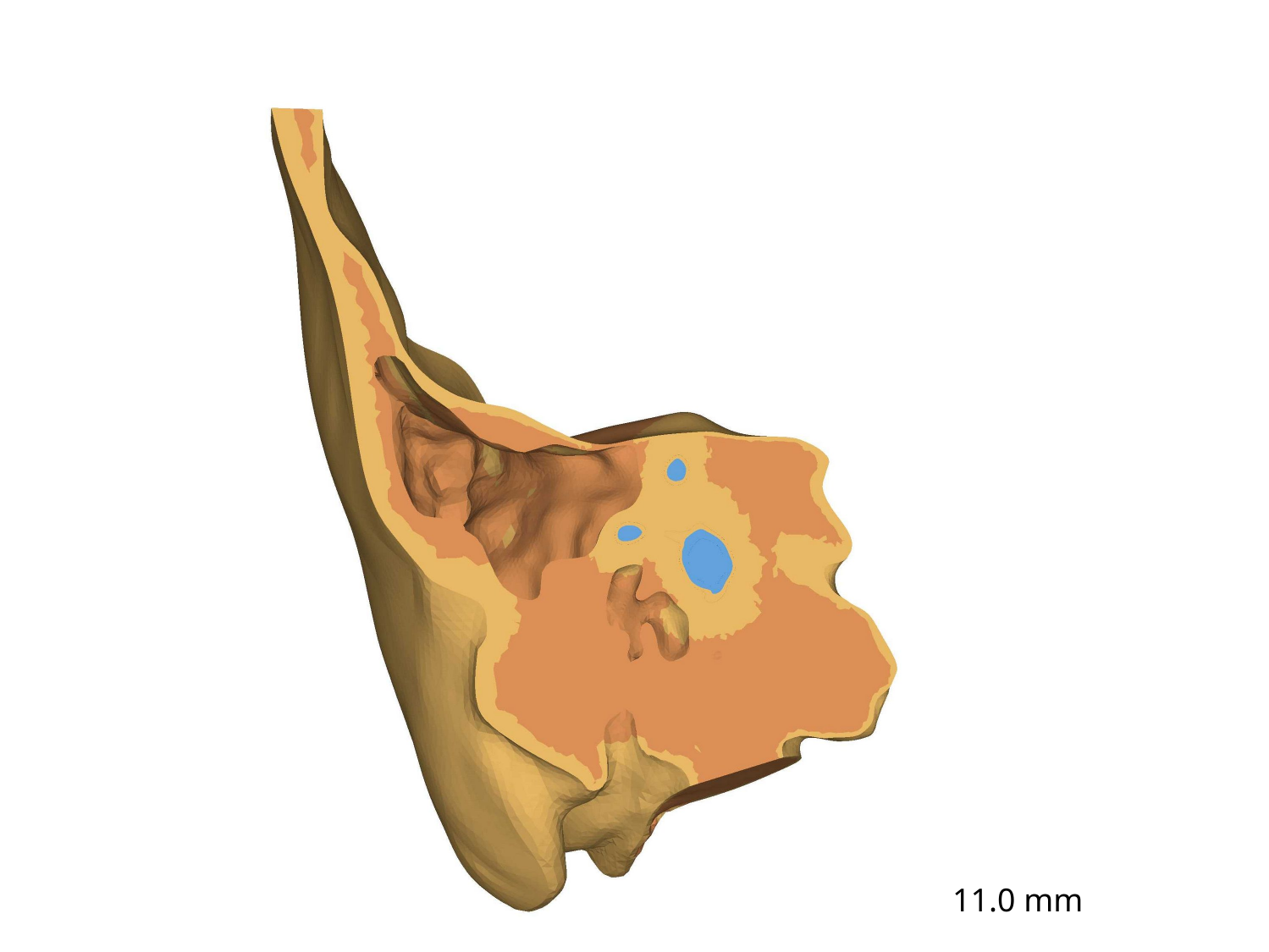

11.0 mm

## Slide 101
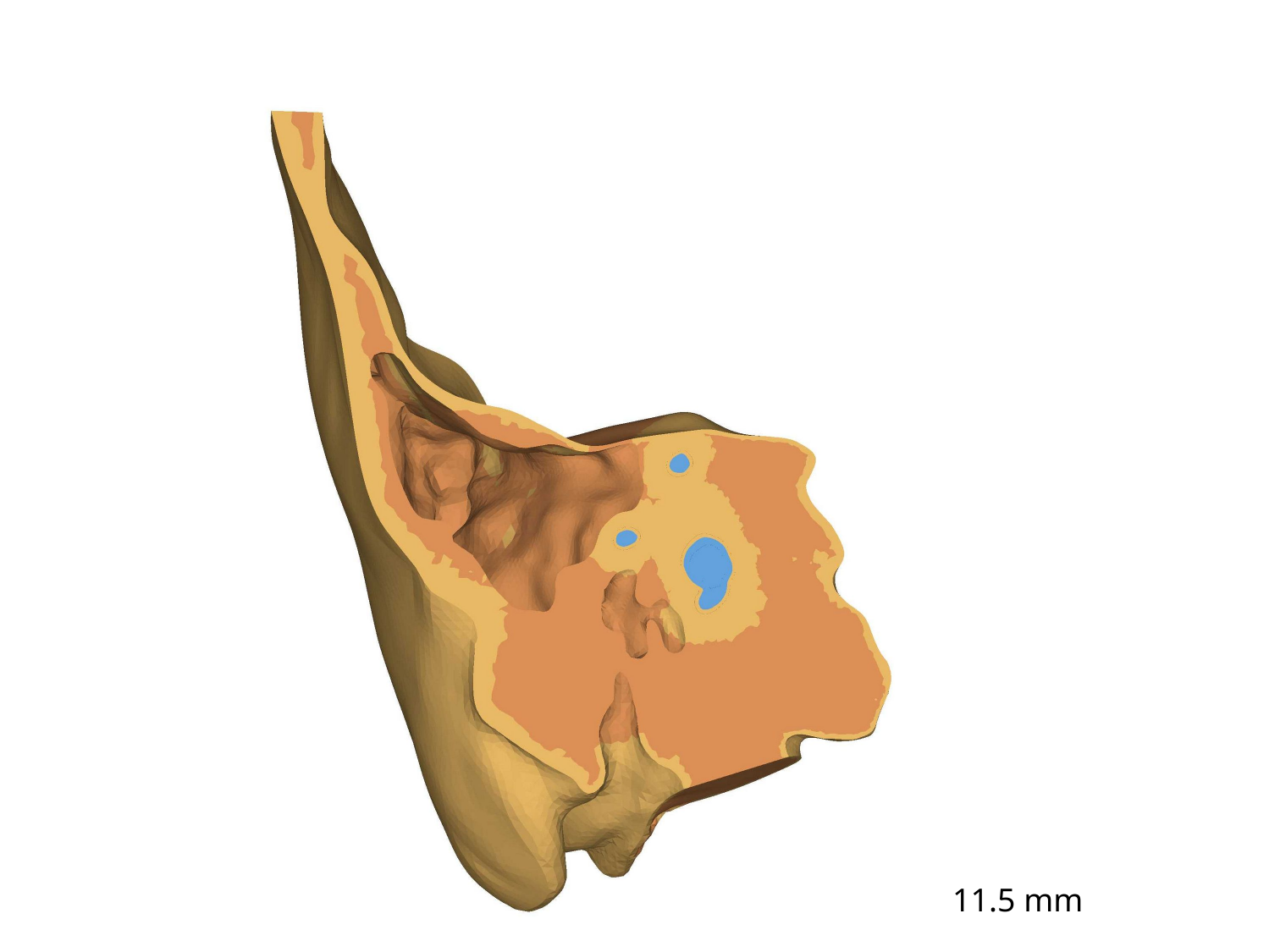

11.5 mm

## Slide 102
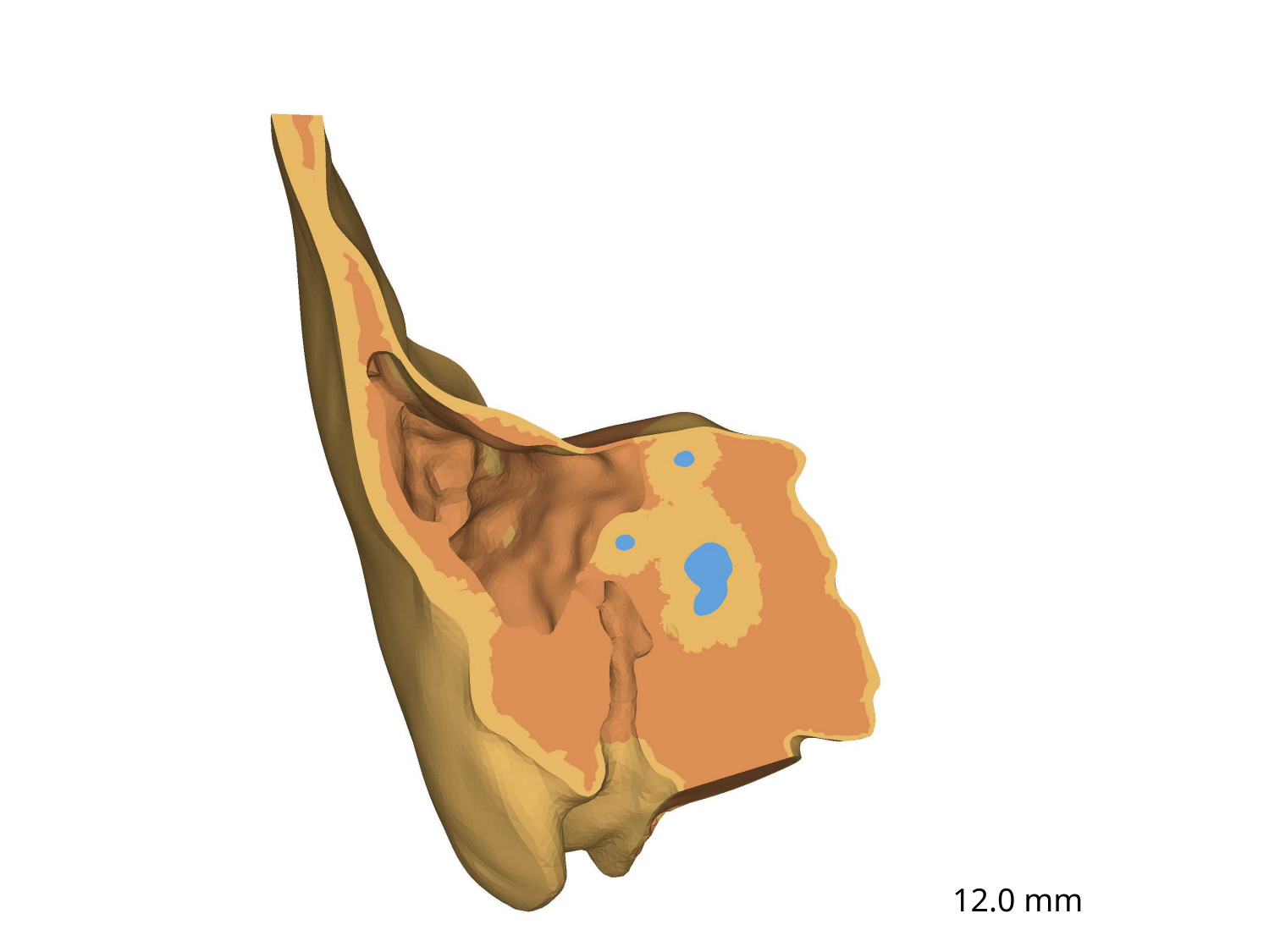

12.0 mm

## Slide 103
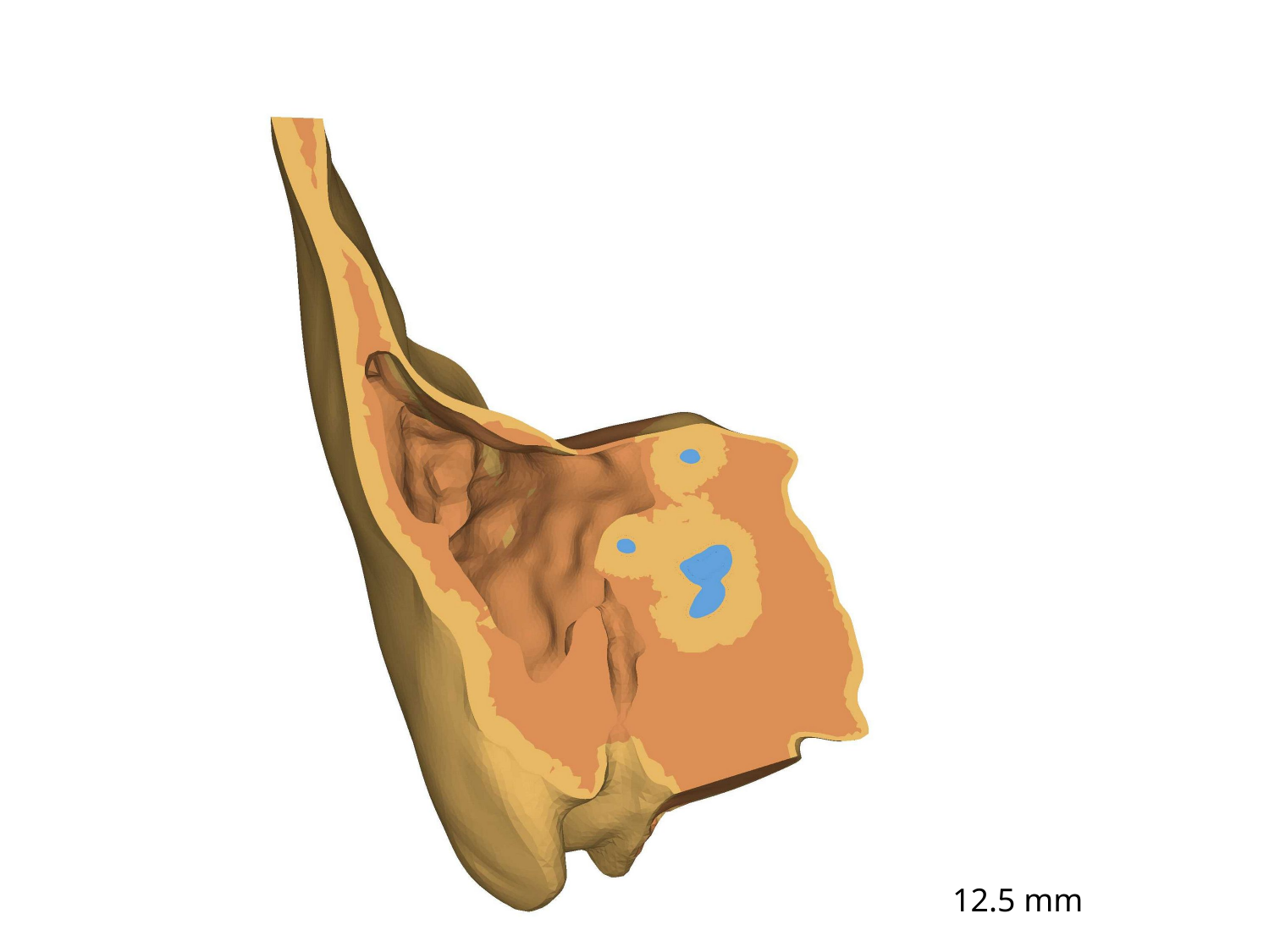

12.5 mm

## Slide 104
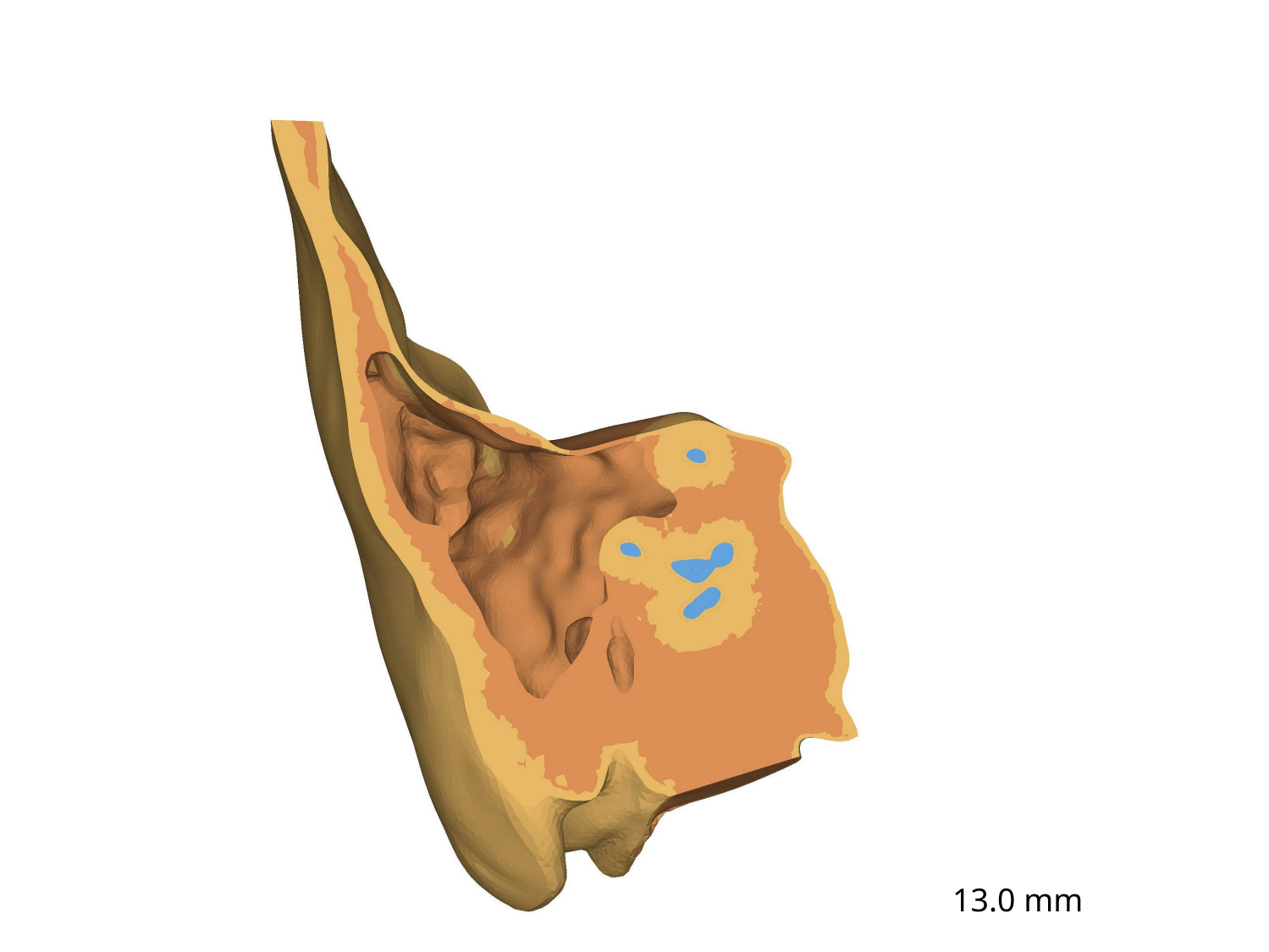

13.0 mm

## Slide 105
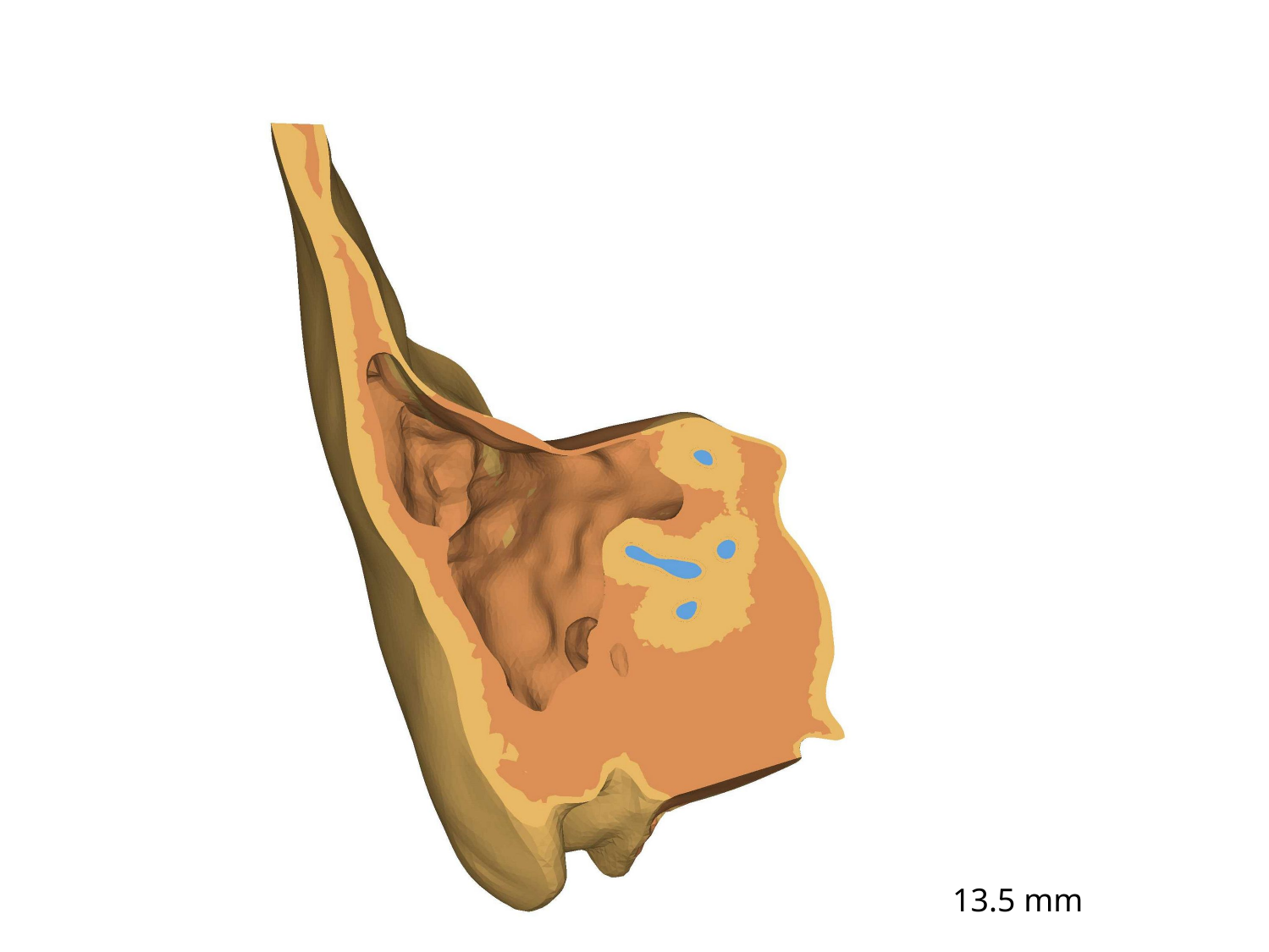

13.5 mm

## Slide 106
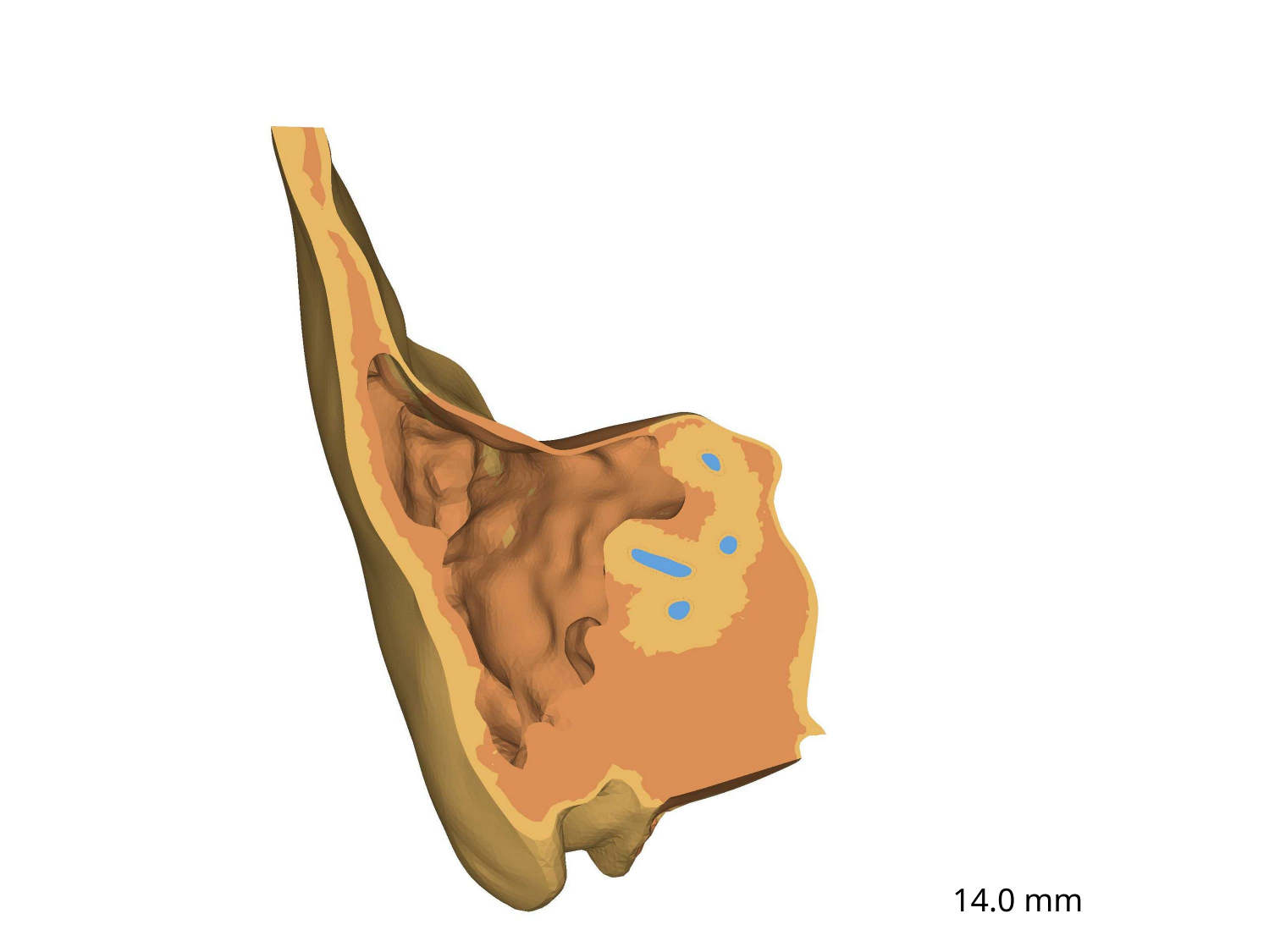

14.0 mm

## Slide 107
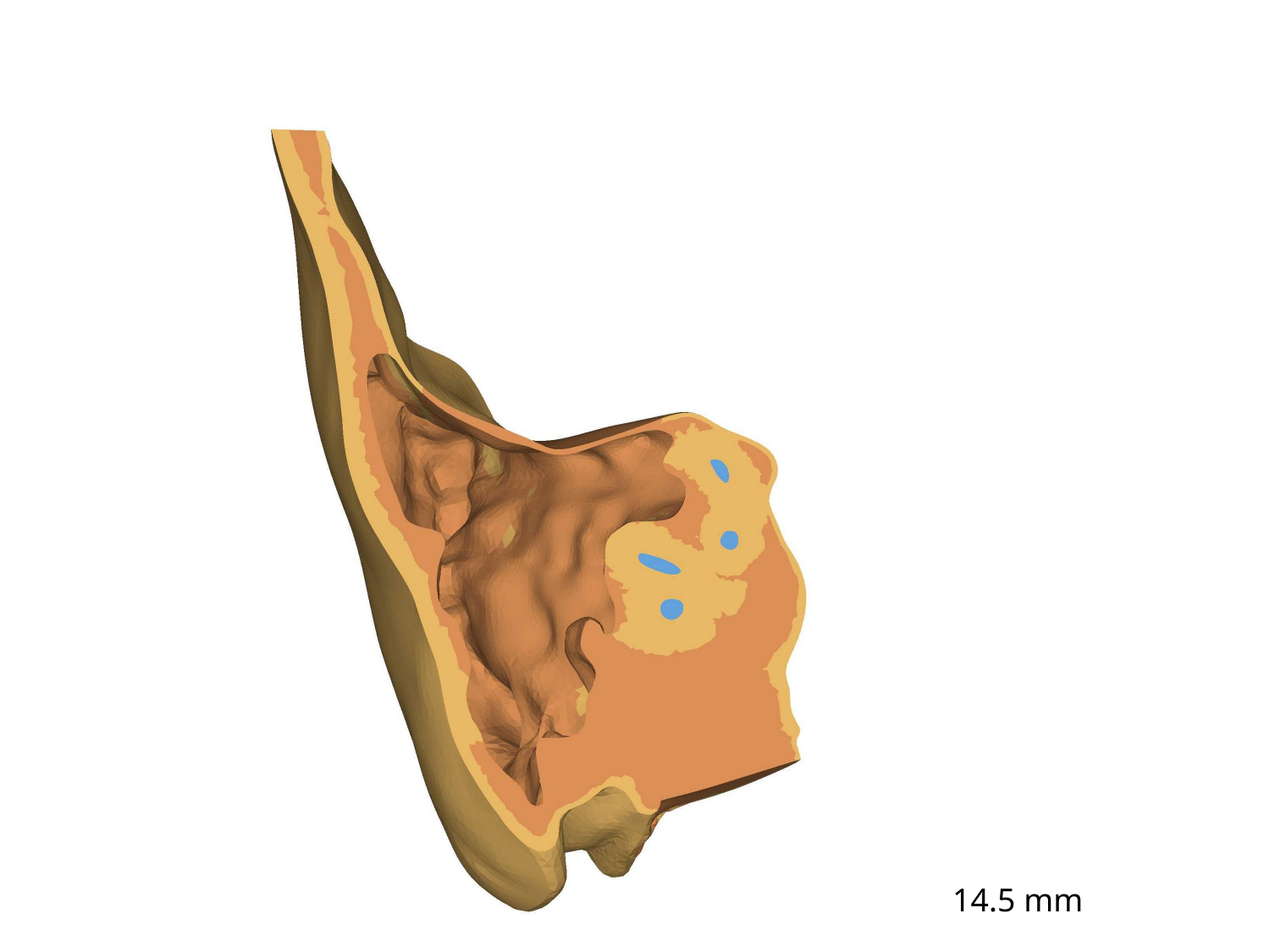

14.5 mm

## Slide 108
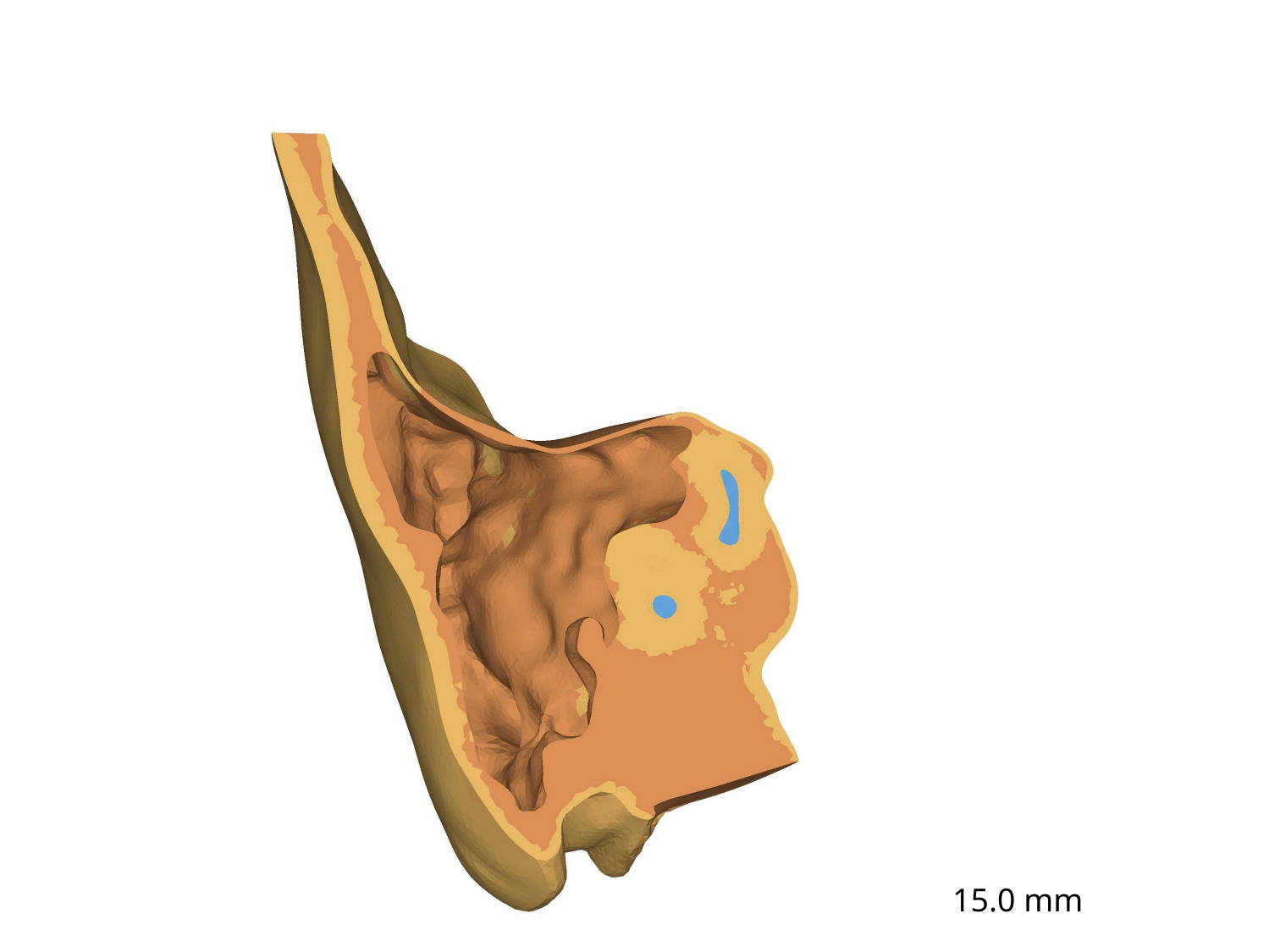

15.0 mm

## Slide 109
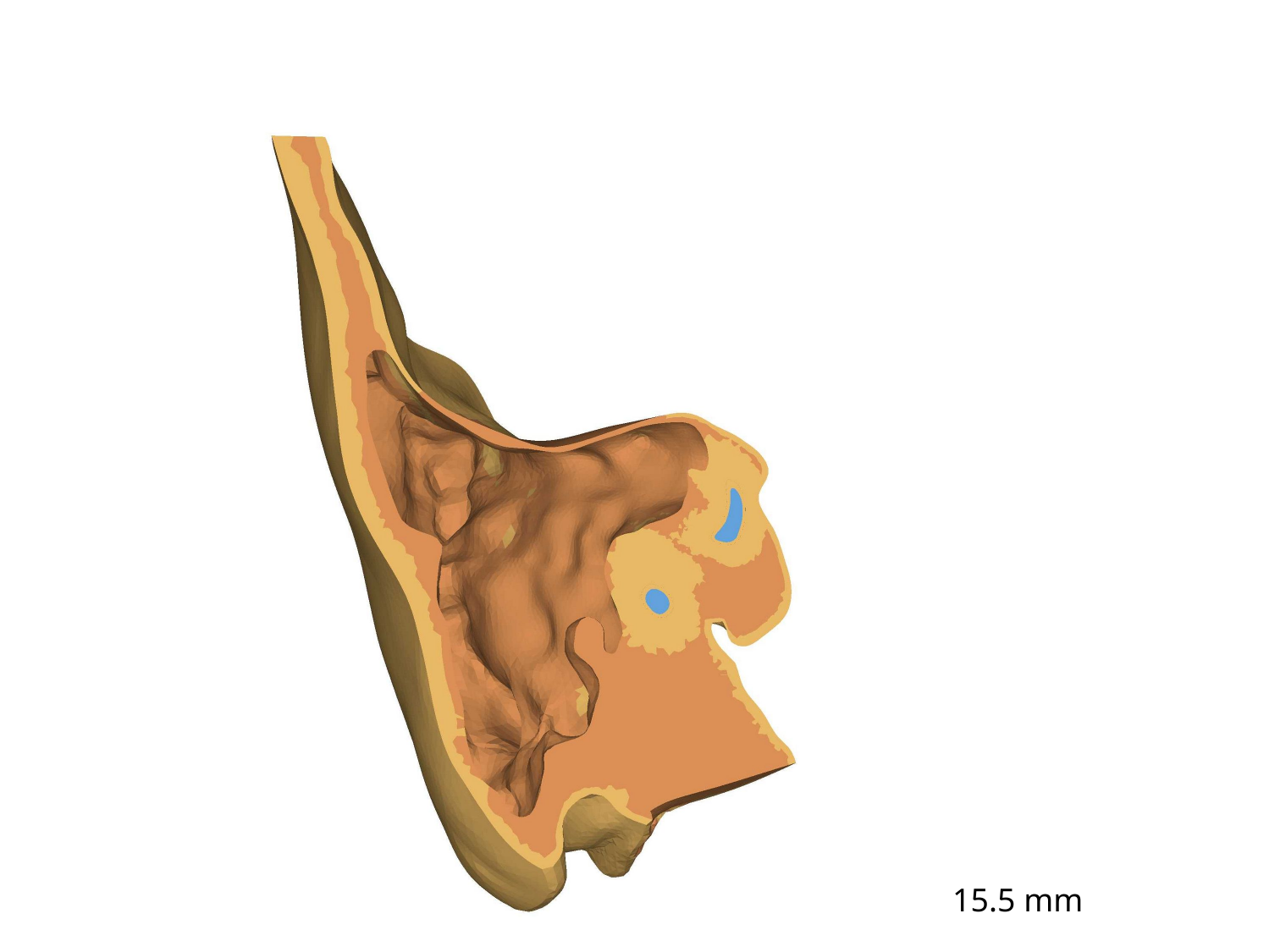

15.5 mm

## Slide 110
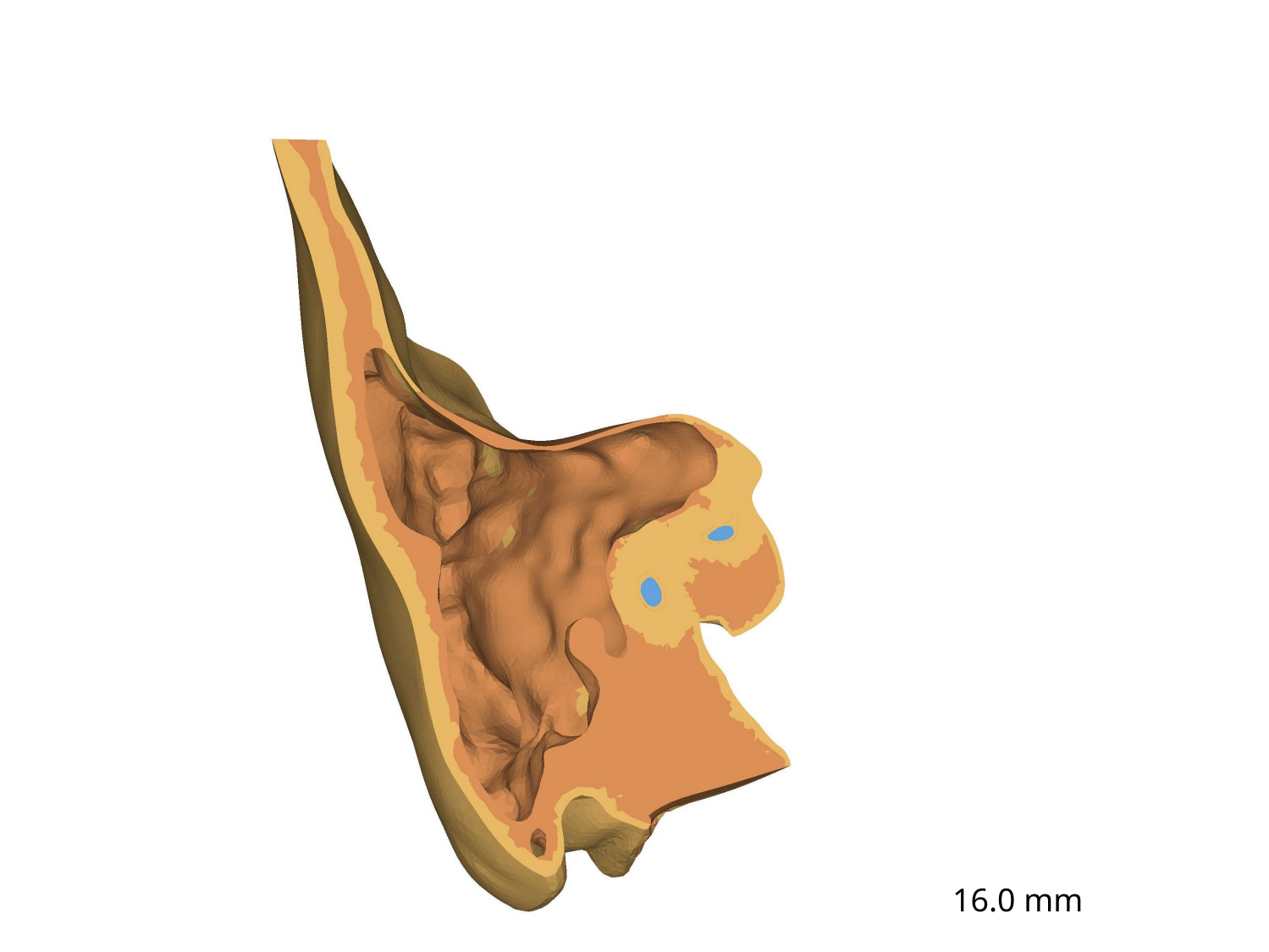

16.0 mm

## Slide 111
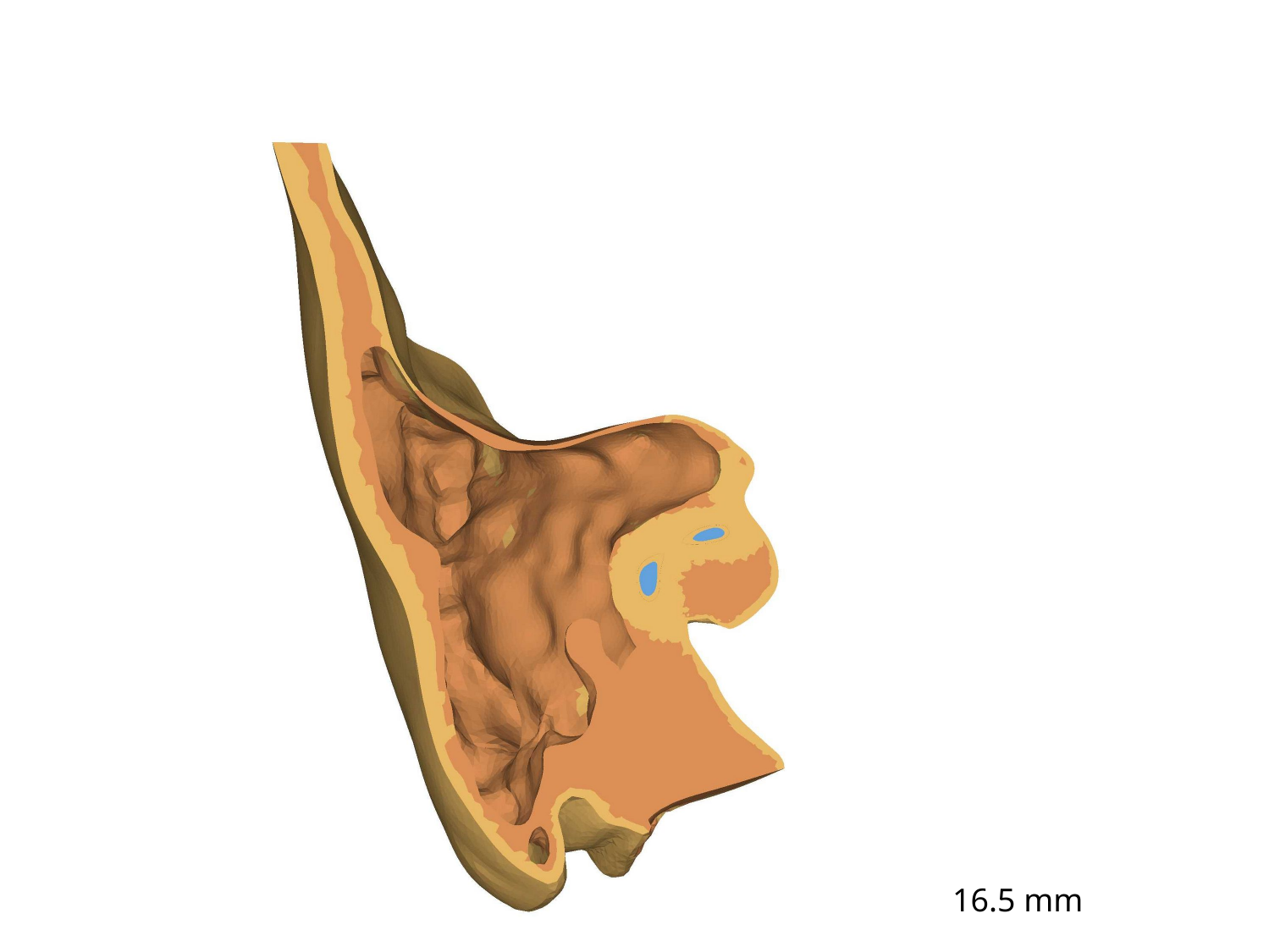

16.5 mm

## Slide 112
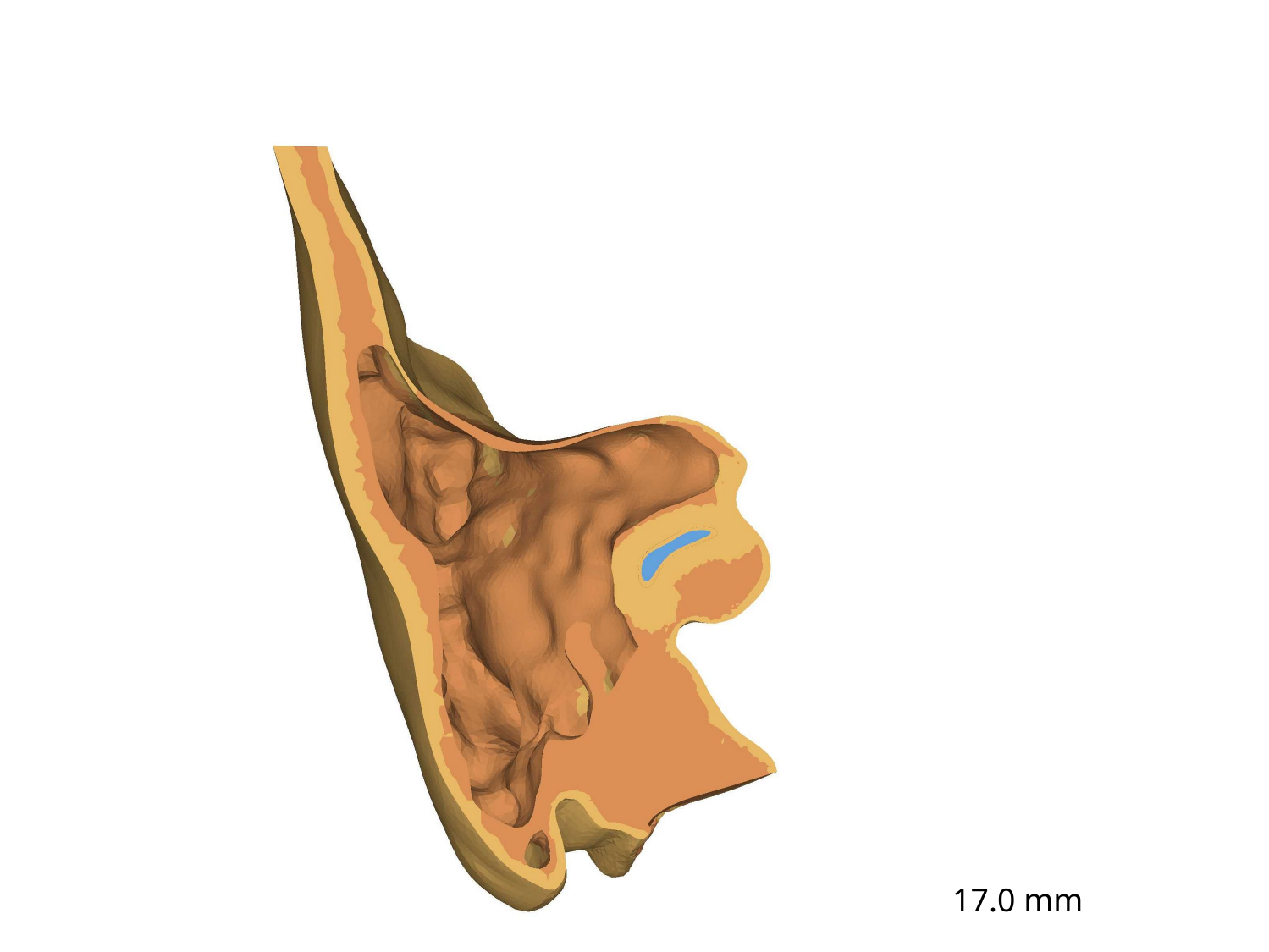

17.0 mm

## Slide 113
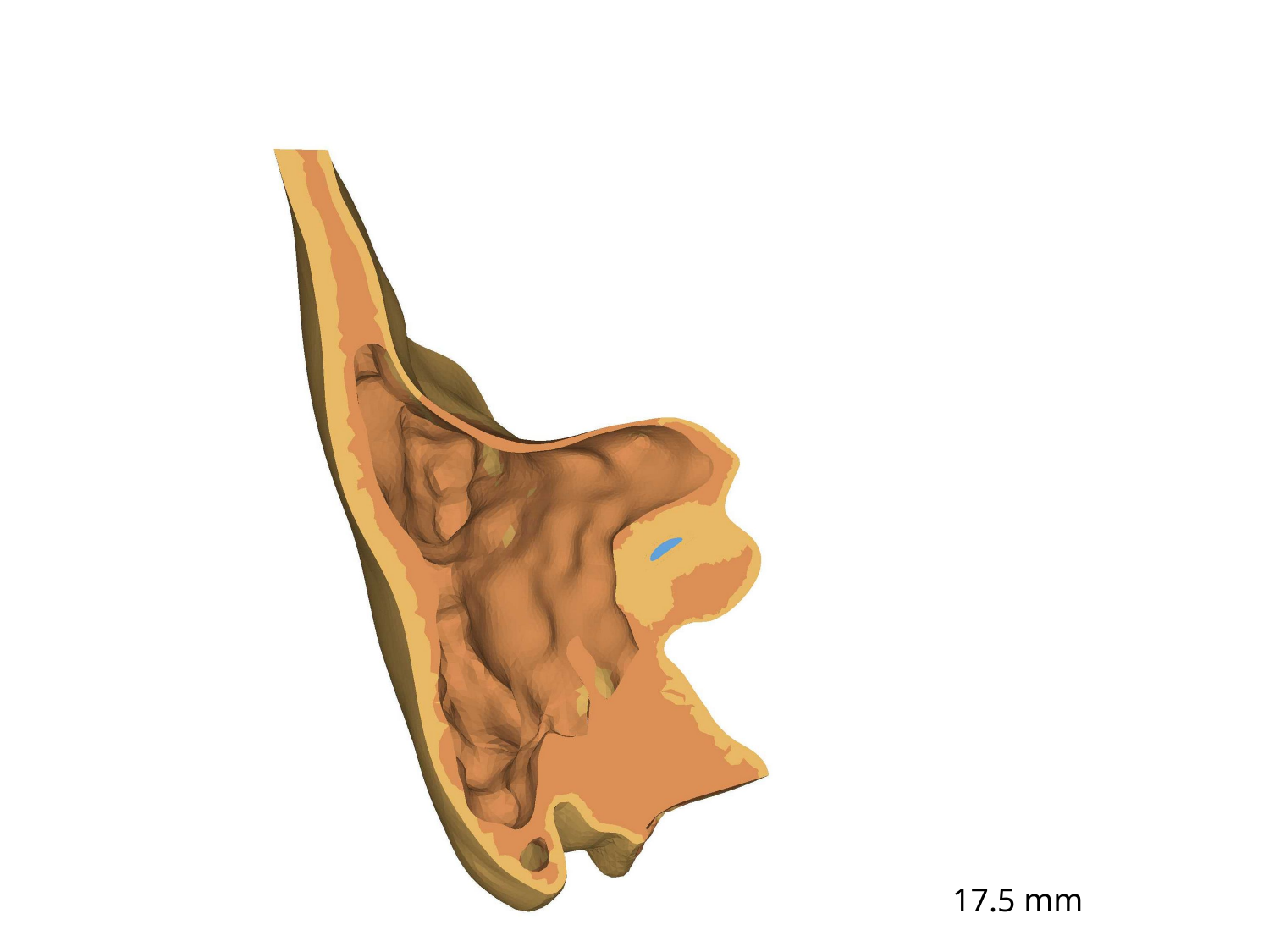

17.5 mm

## Slide 114
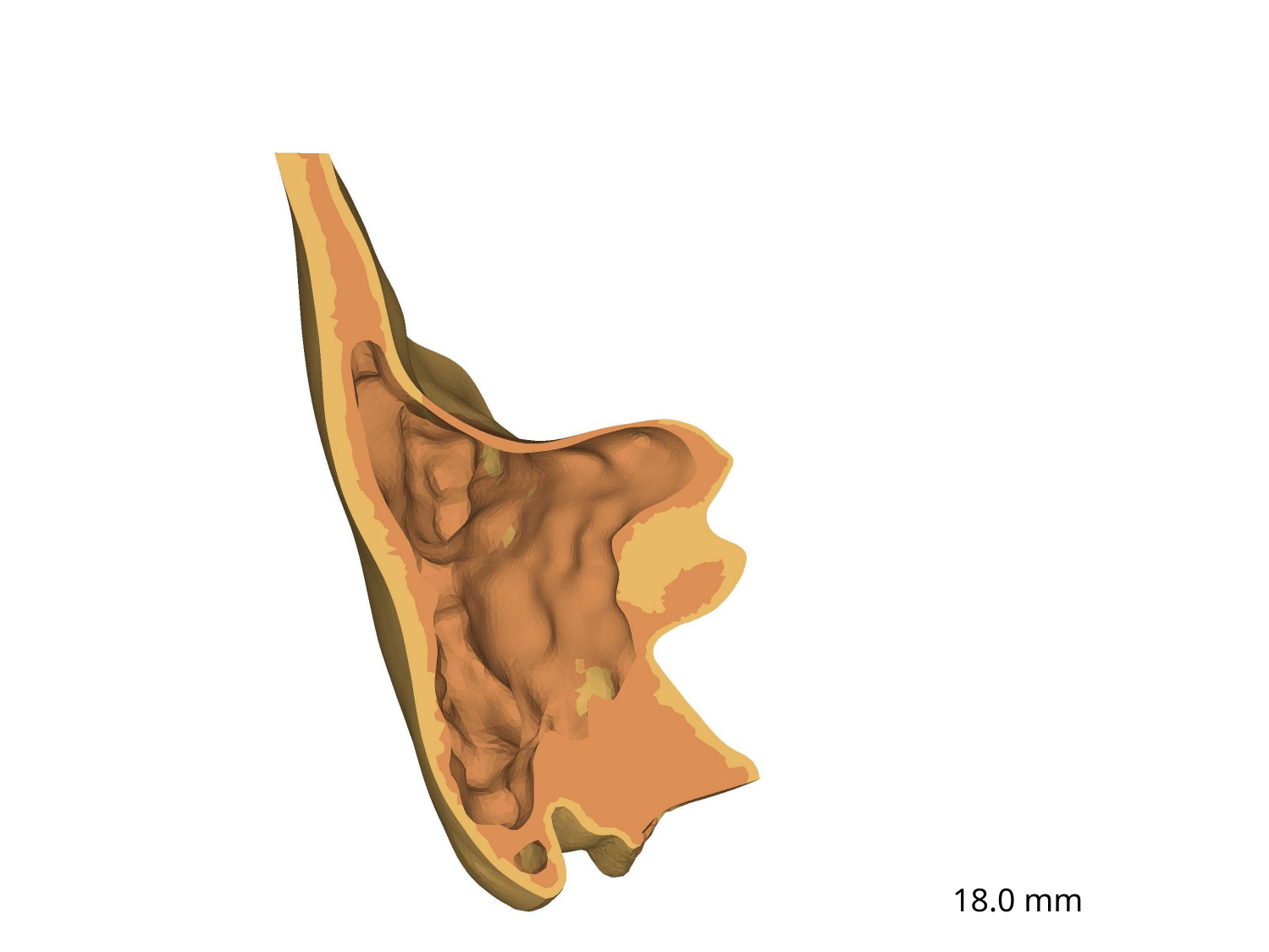

18.0 mm

## Slide 115
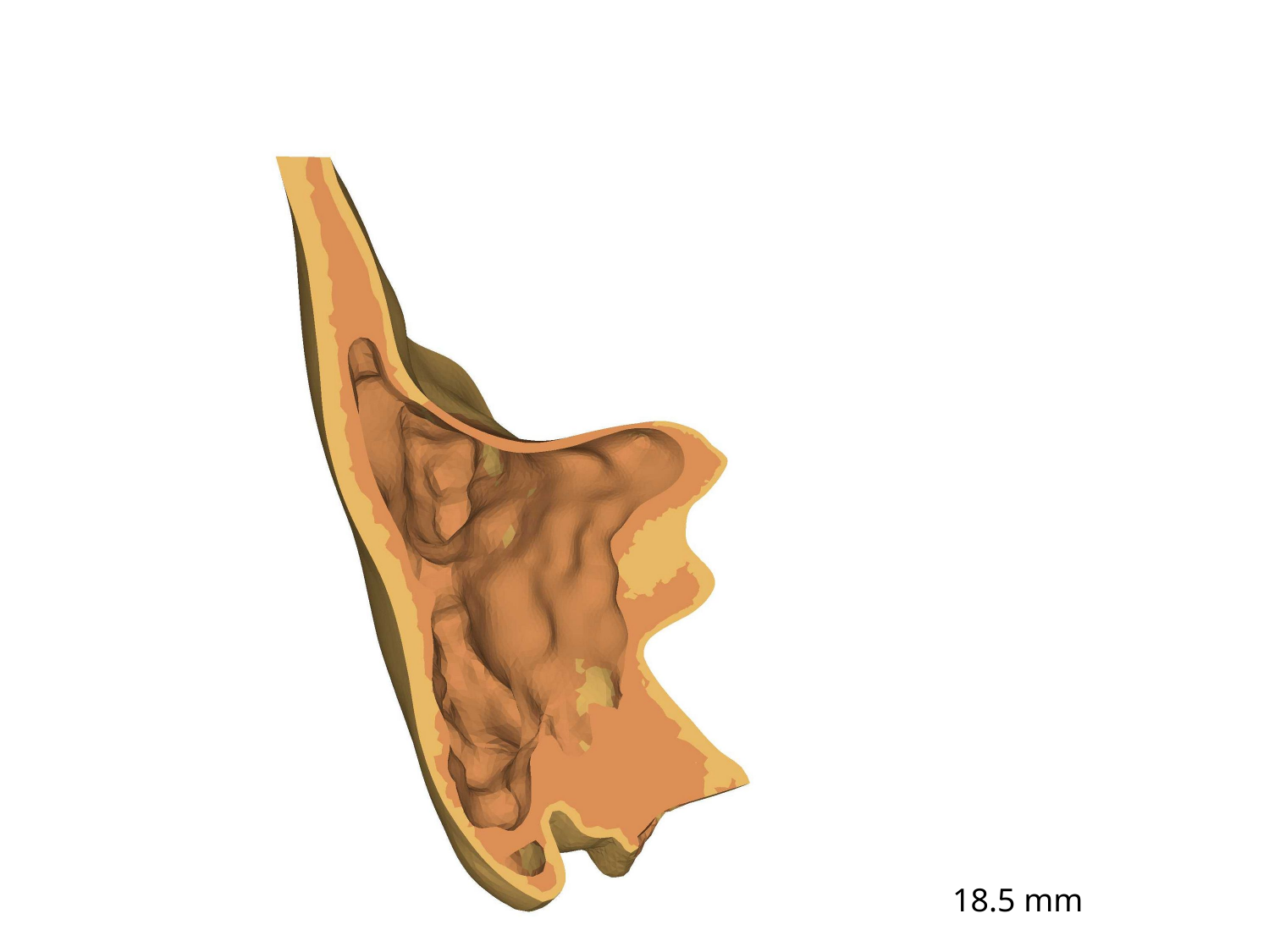

18.5 mm

## Slide 116
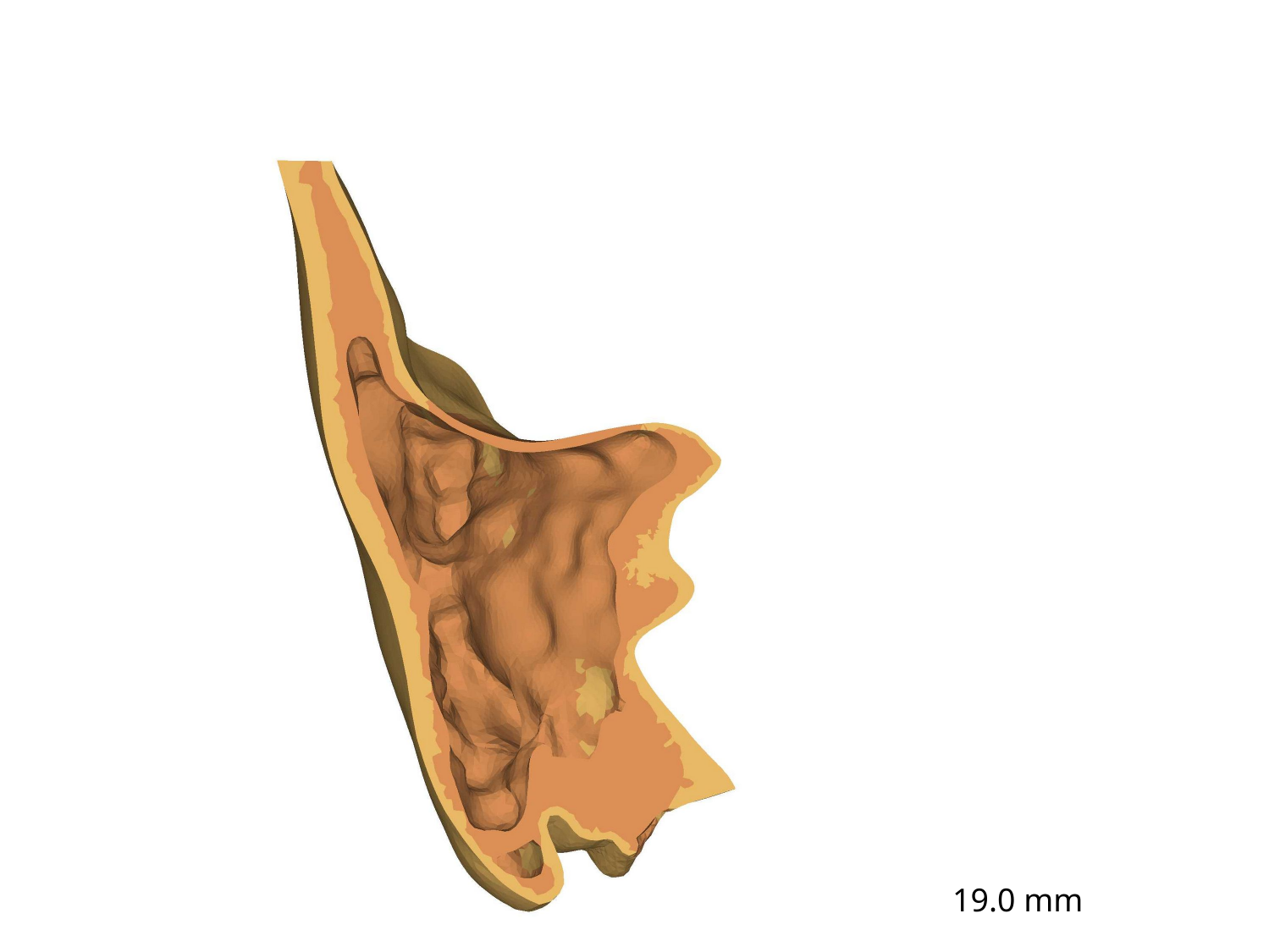

19.0 mm

## Slide 117
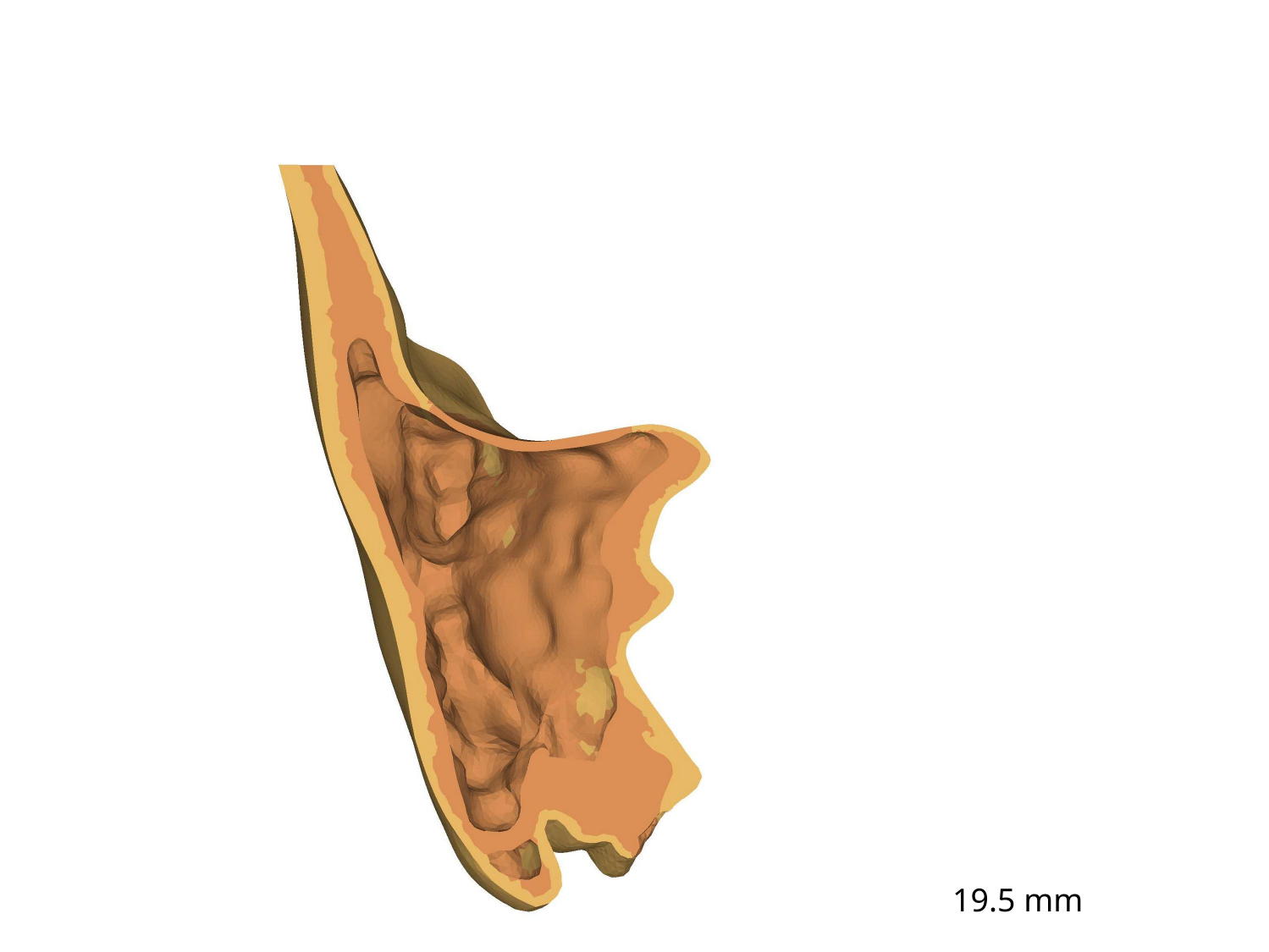

19.5 mm

## Slide 118
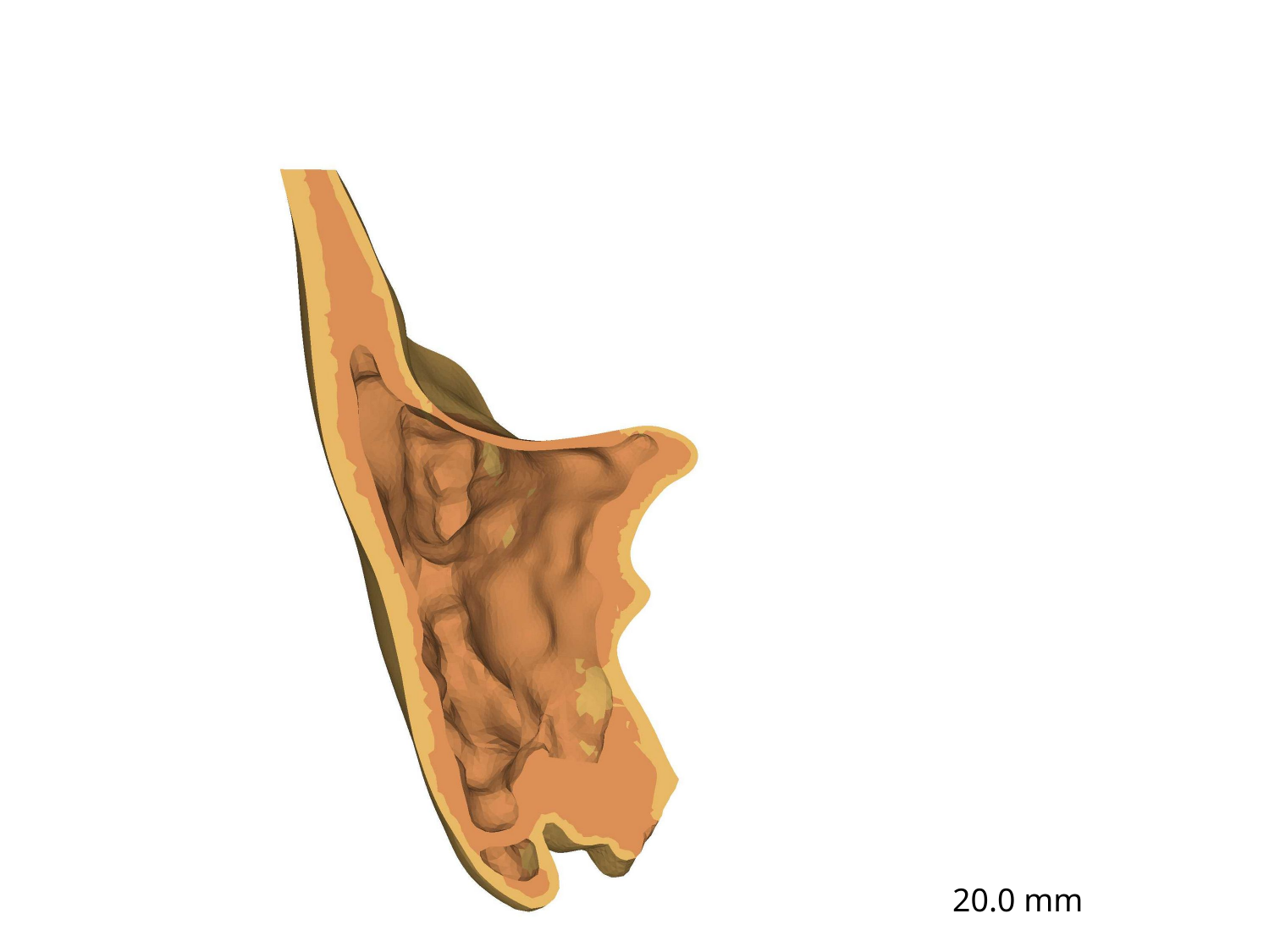

20.0 mm

## Slide 119
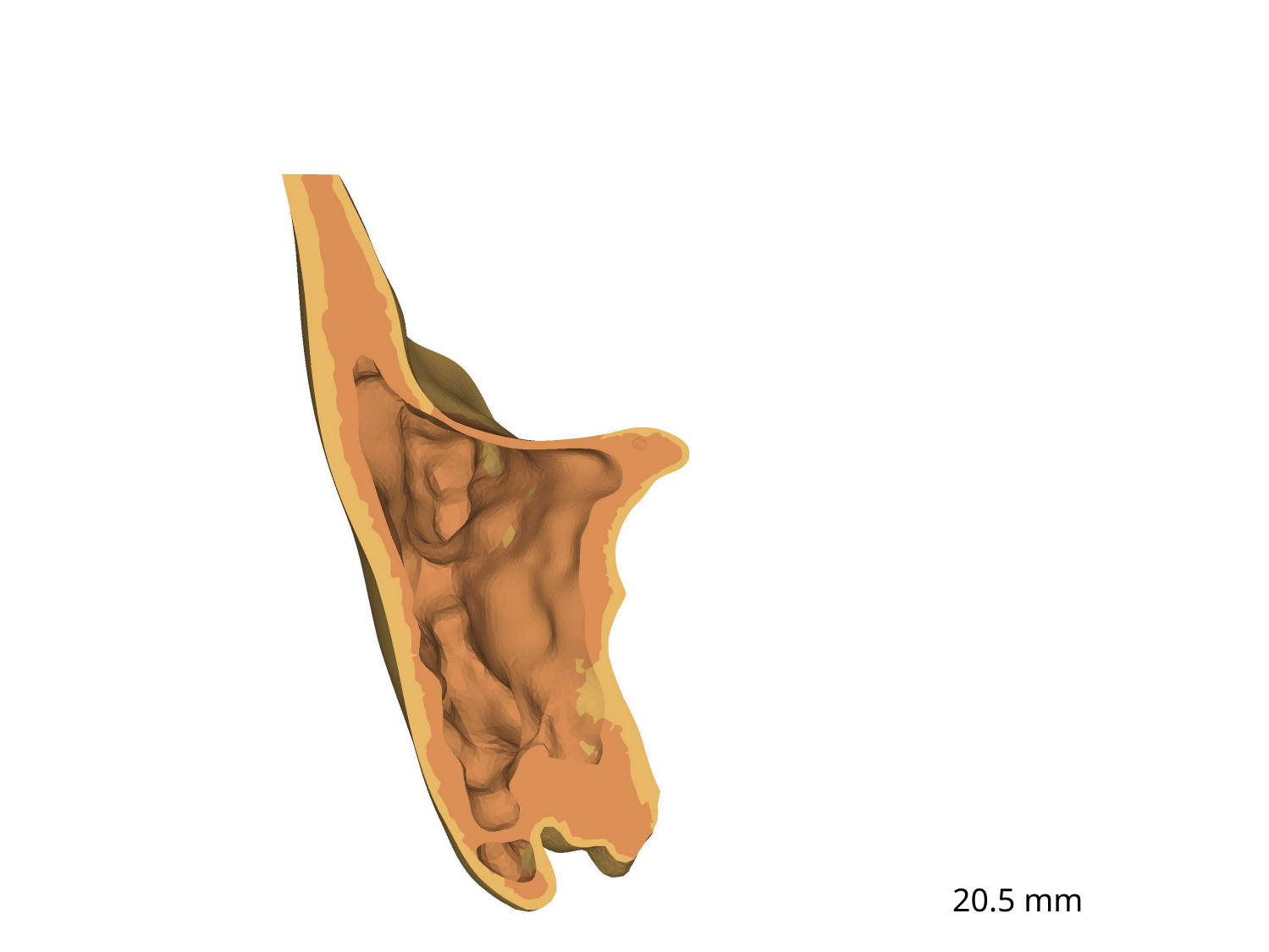

20.5 mm

## Slide 120
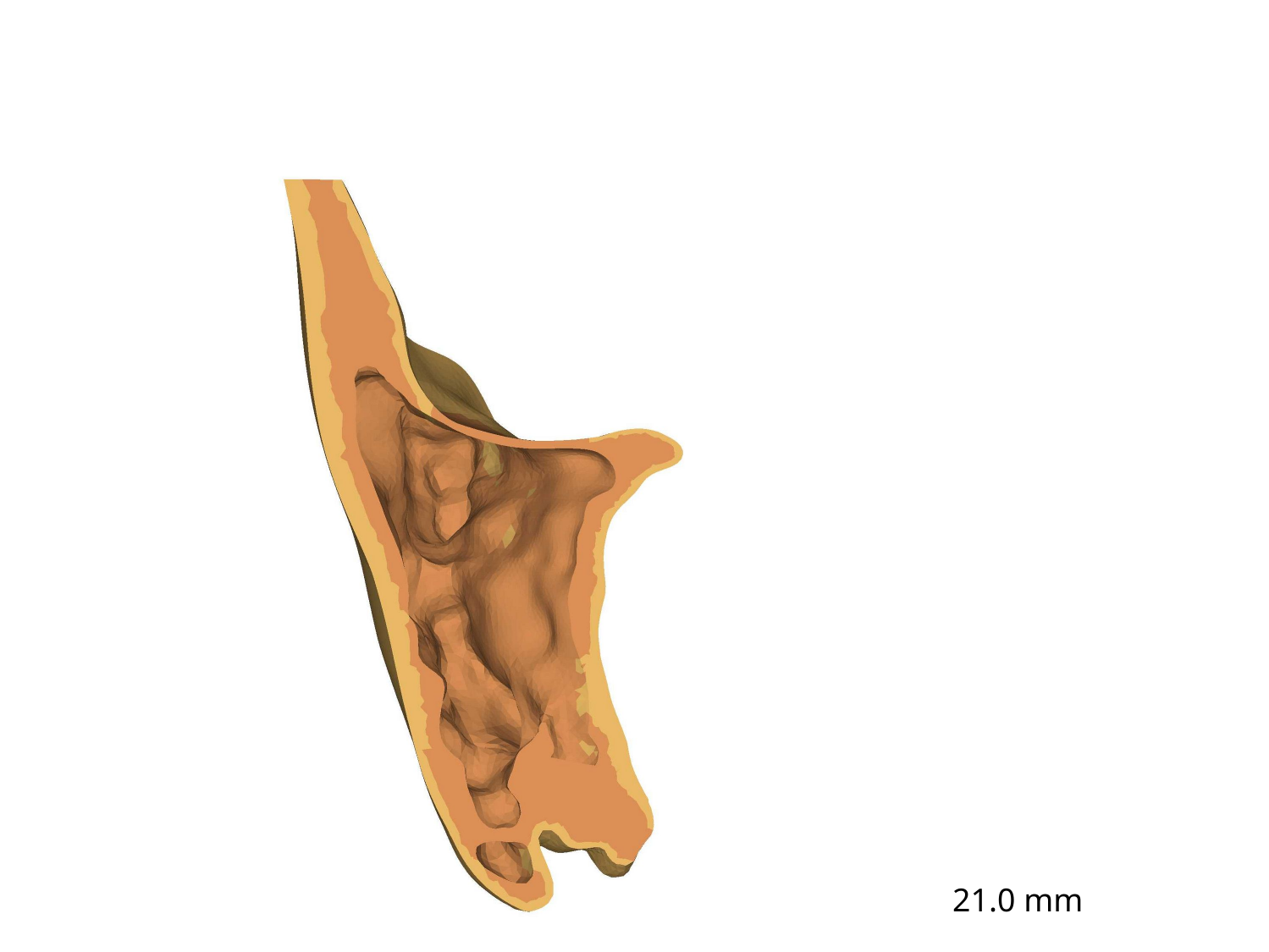

21.0 mm

## Slide 121
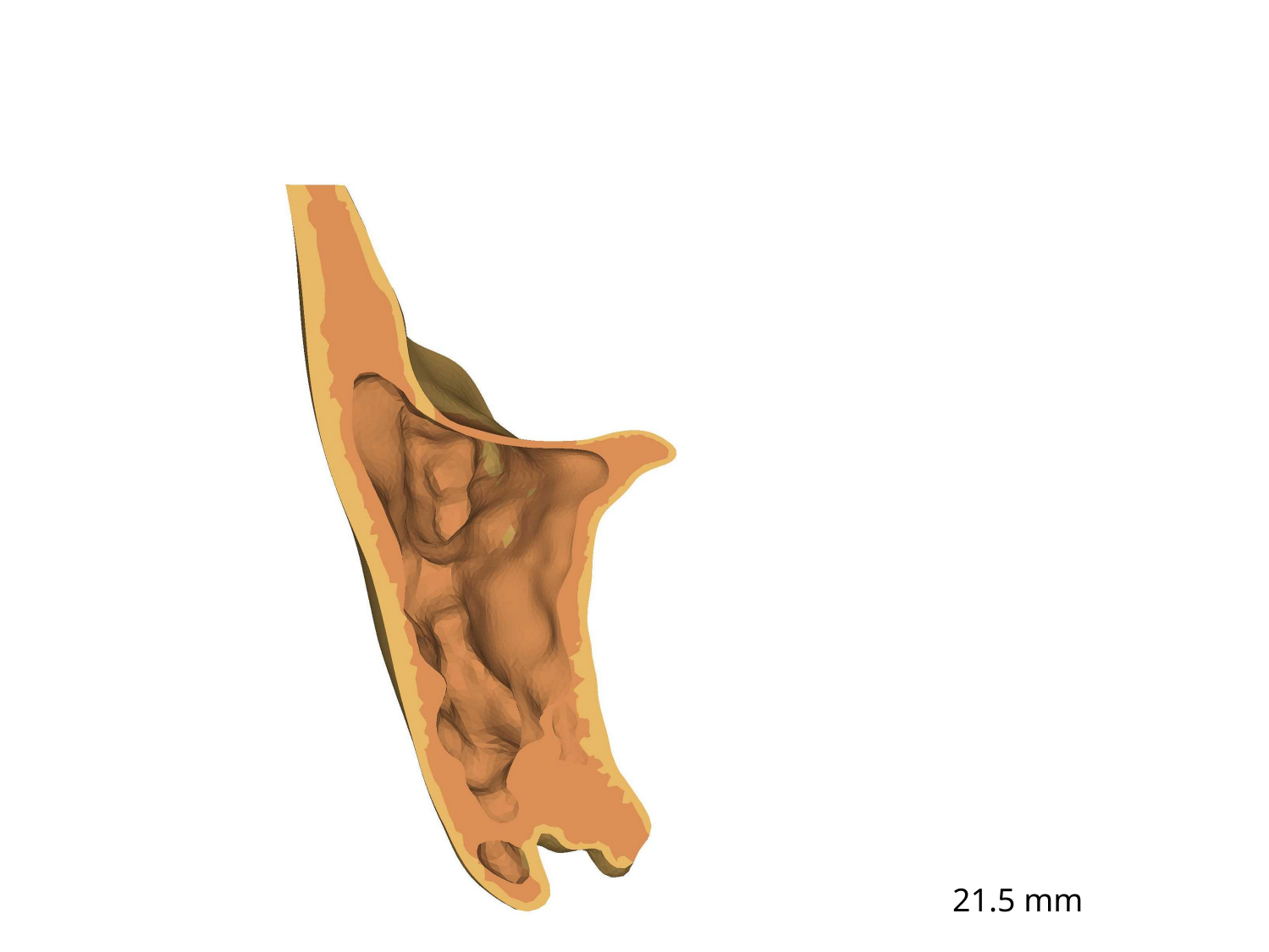

21.5 mm

## Slide 122
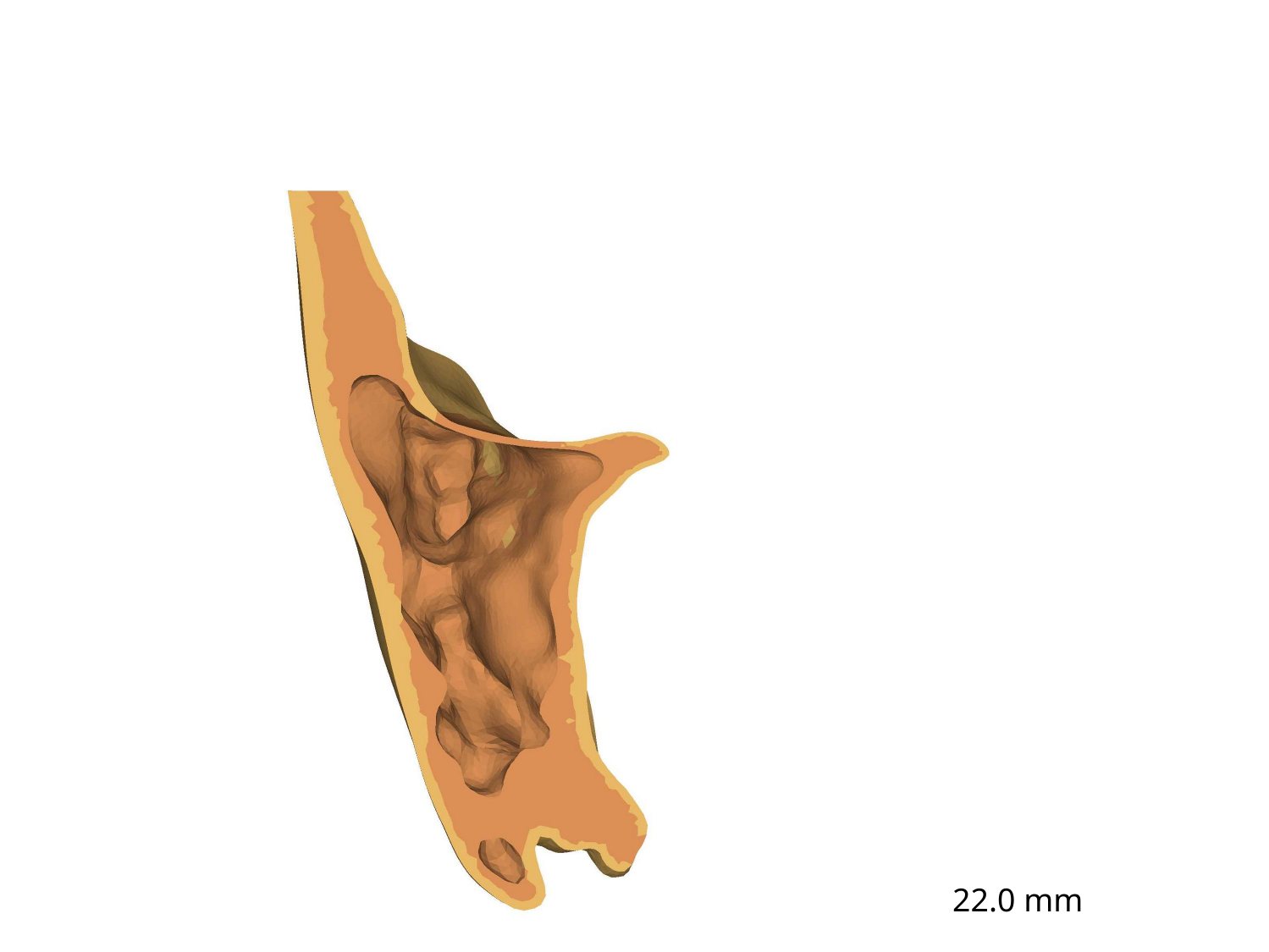

22.0 mm

## Slide 123
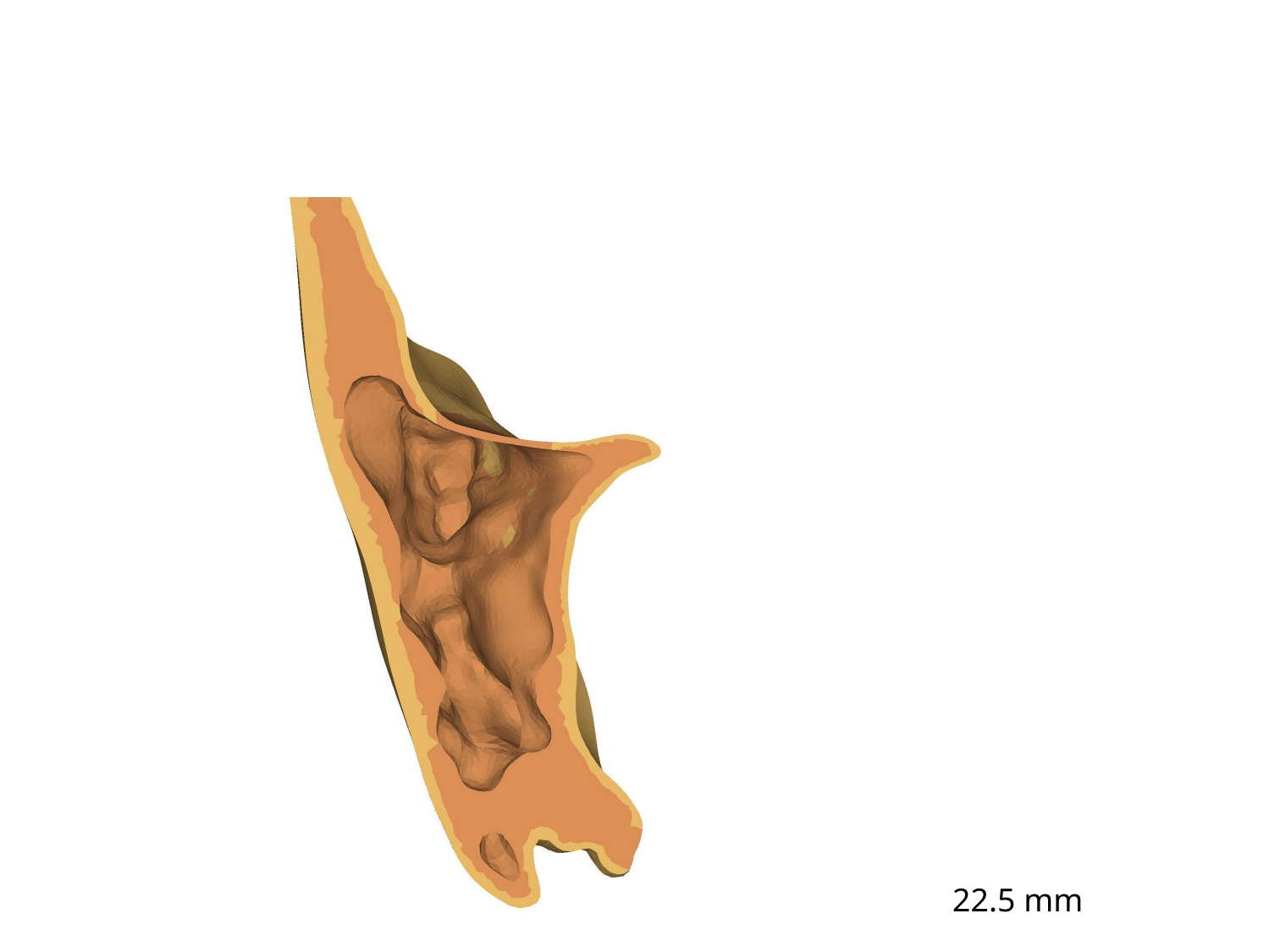

22.5 mm

## Slide 124
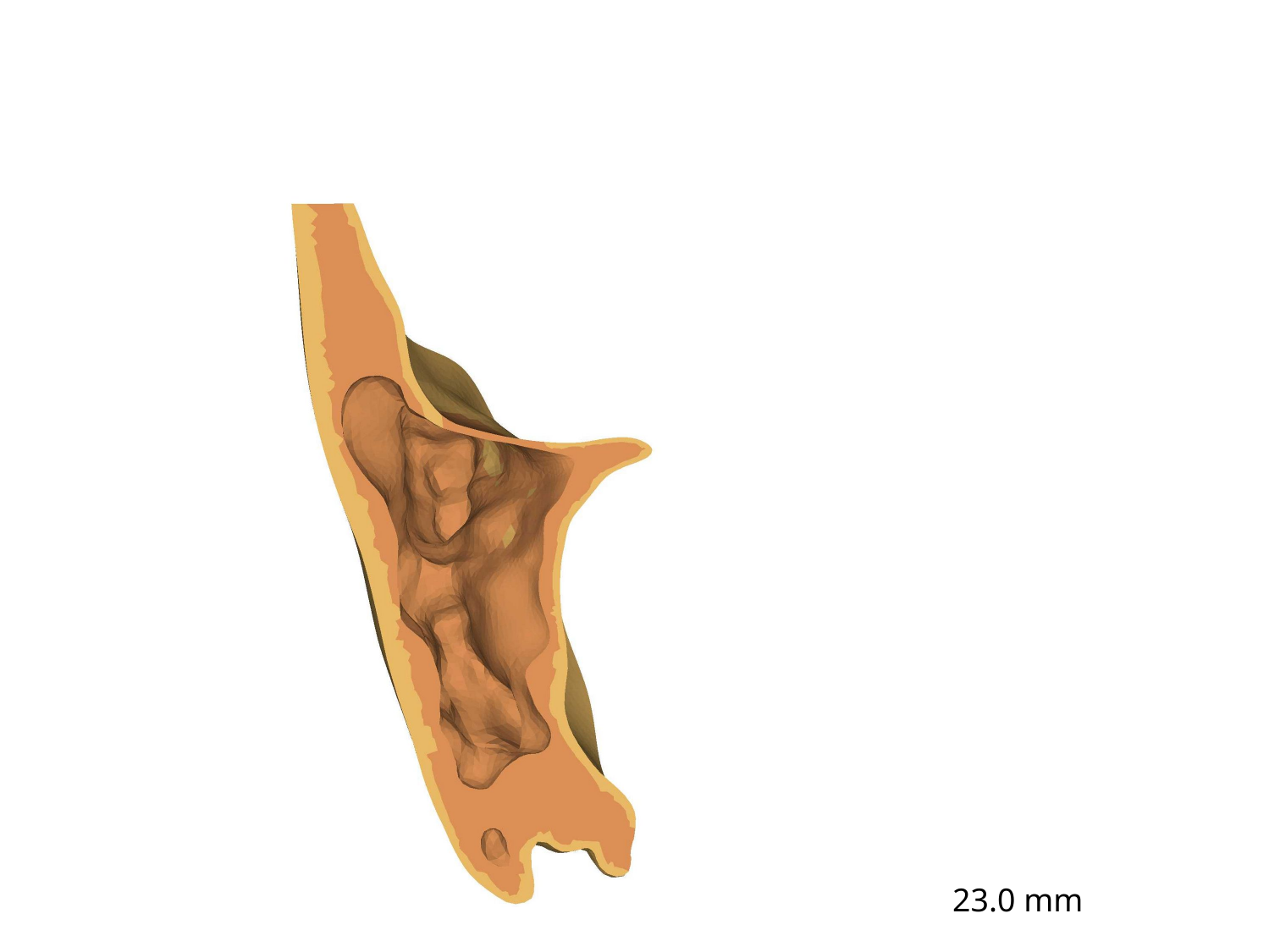

23.0 mm

## Slide 125
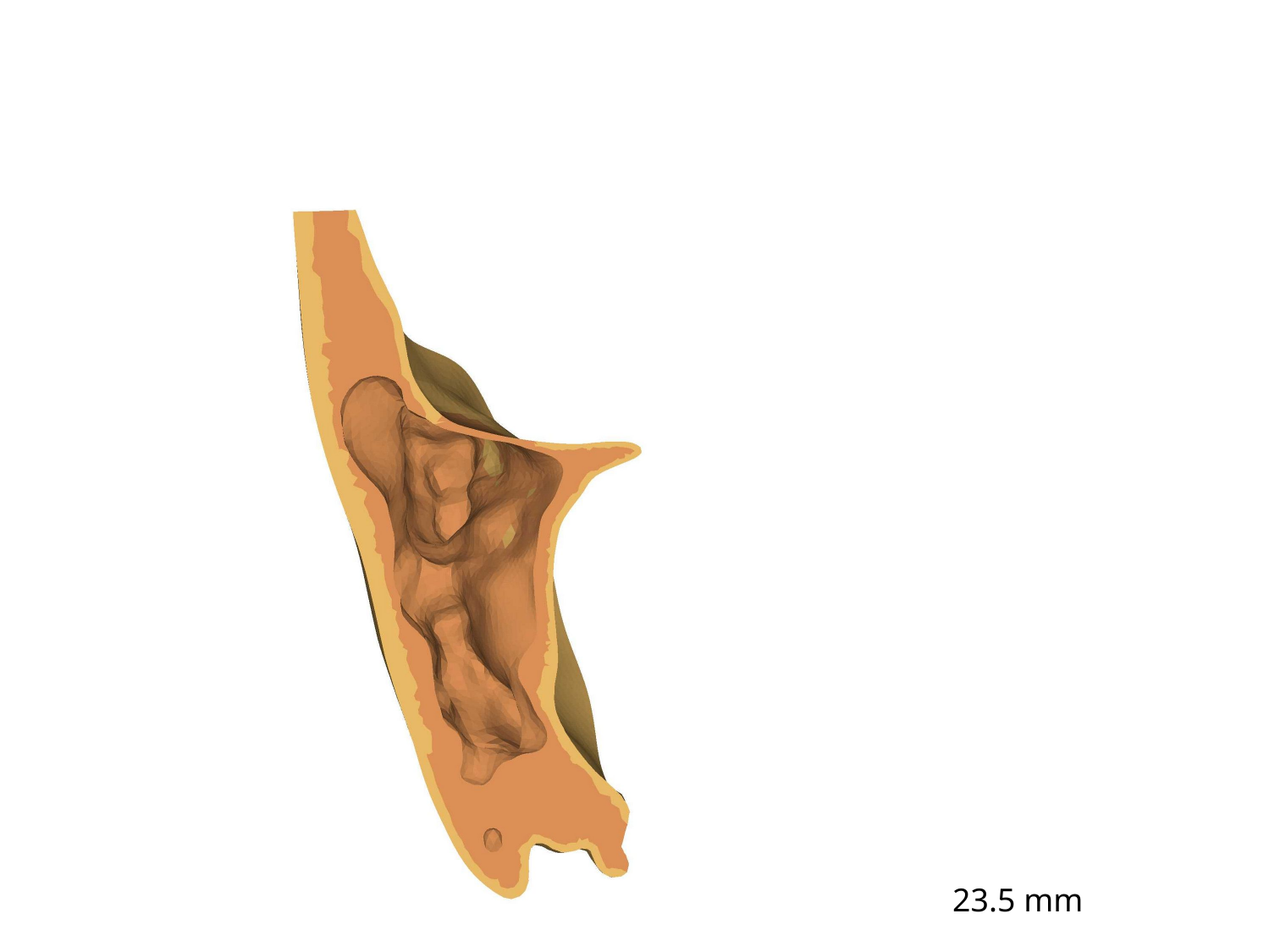

23.5 mm

## Slide 126
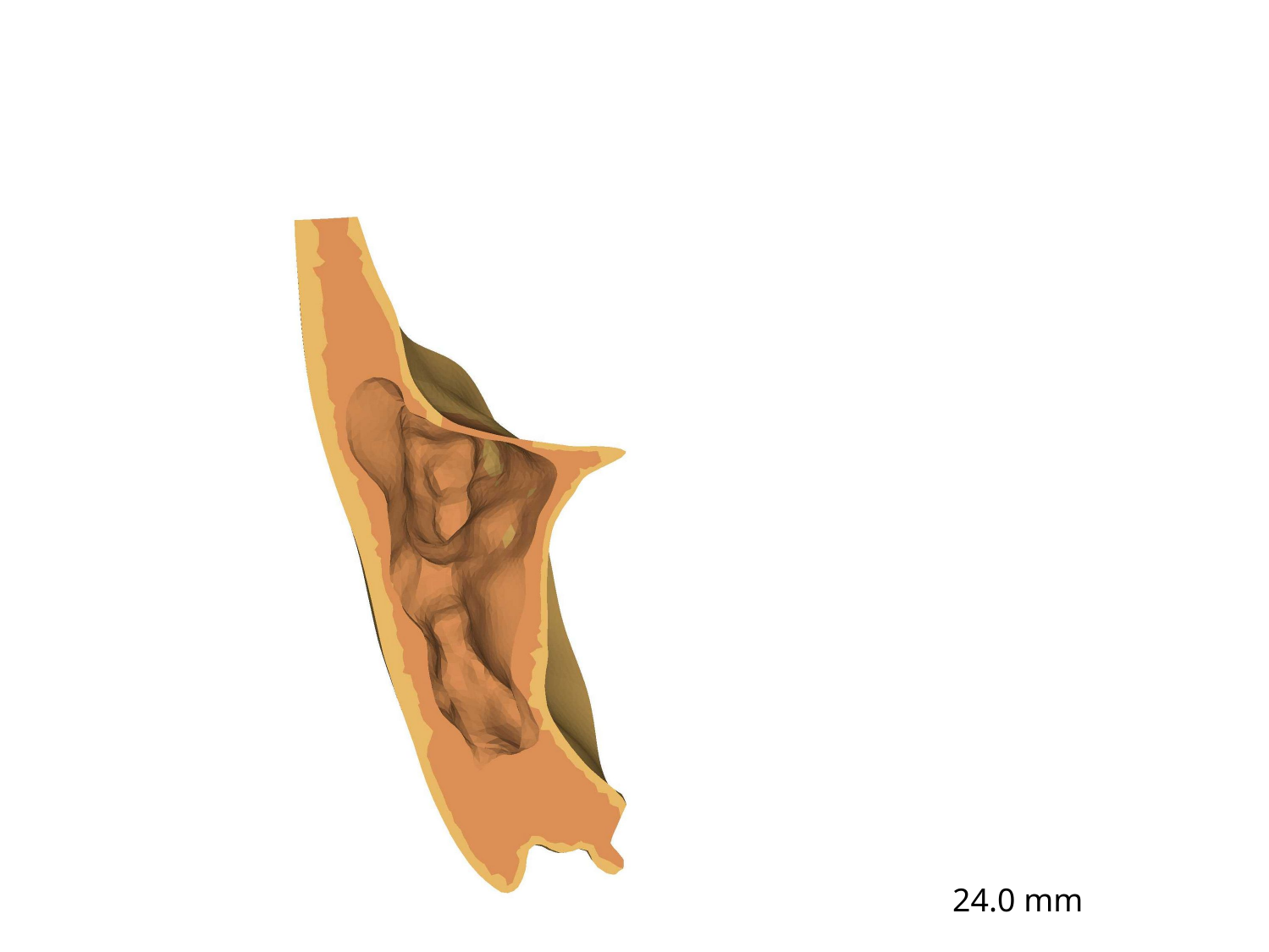

24.0 mm

## Slide 127
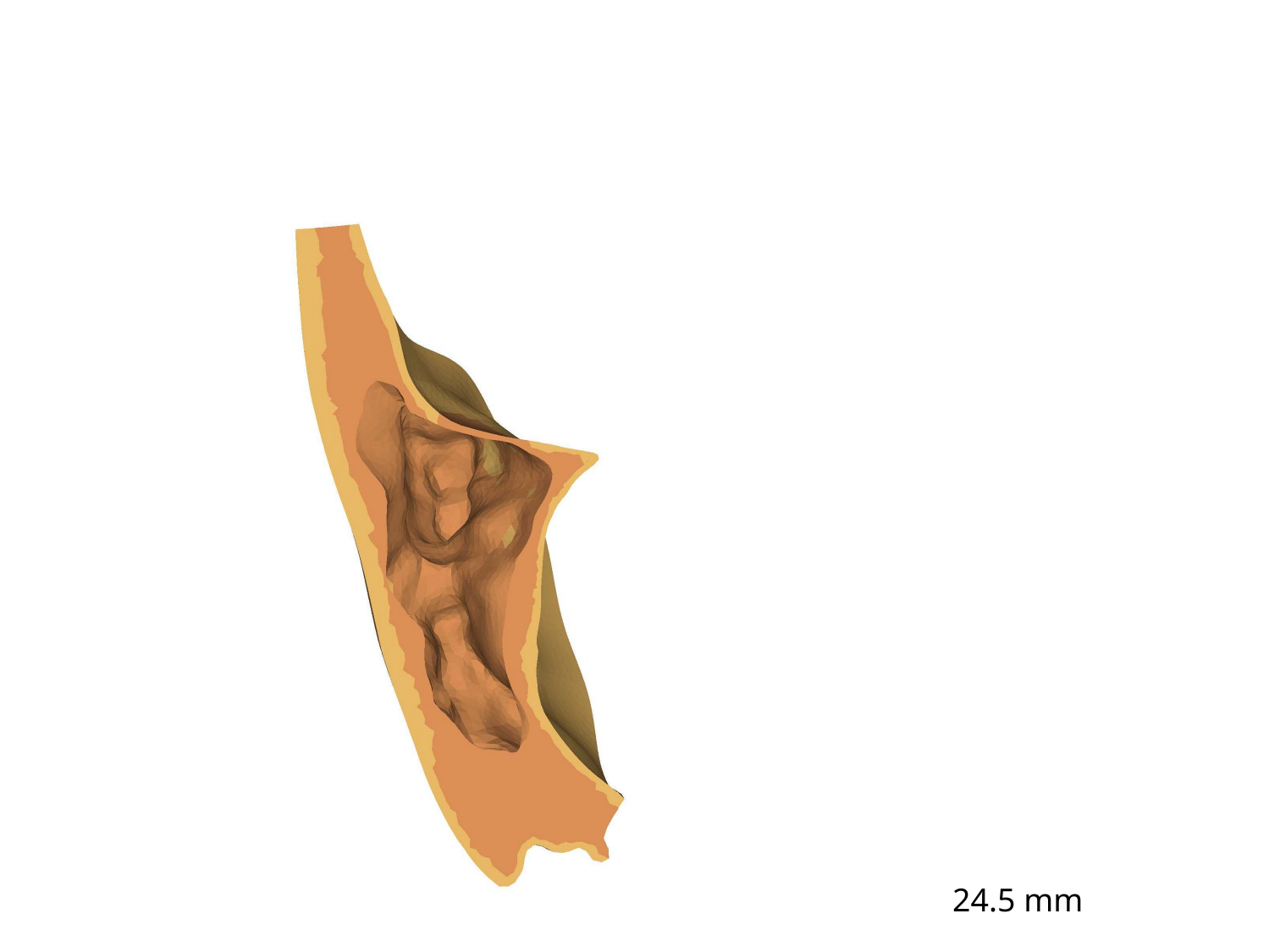

24.5 mm

## Slide 128
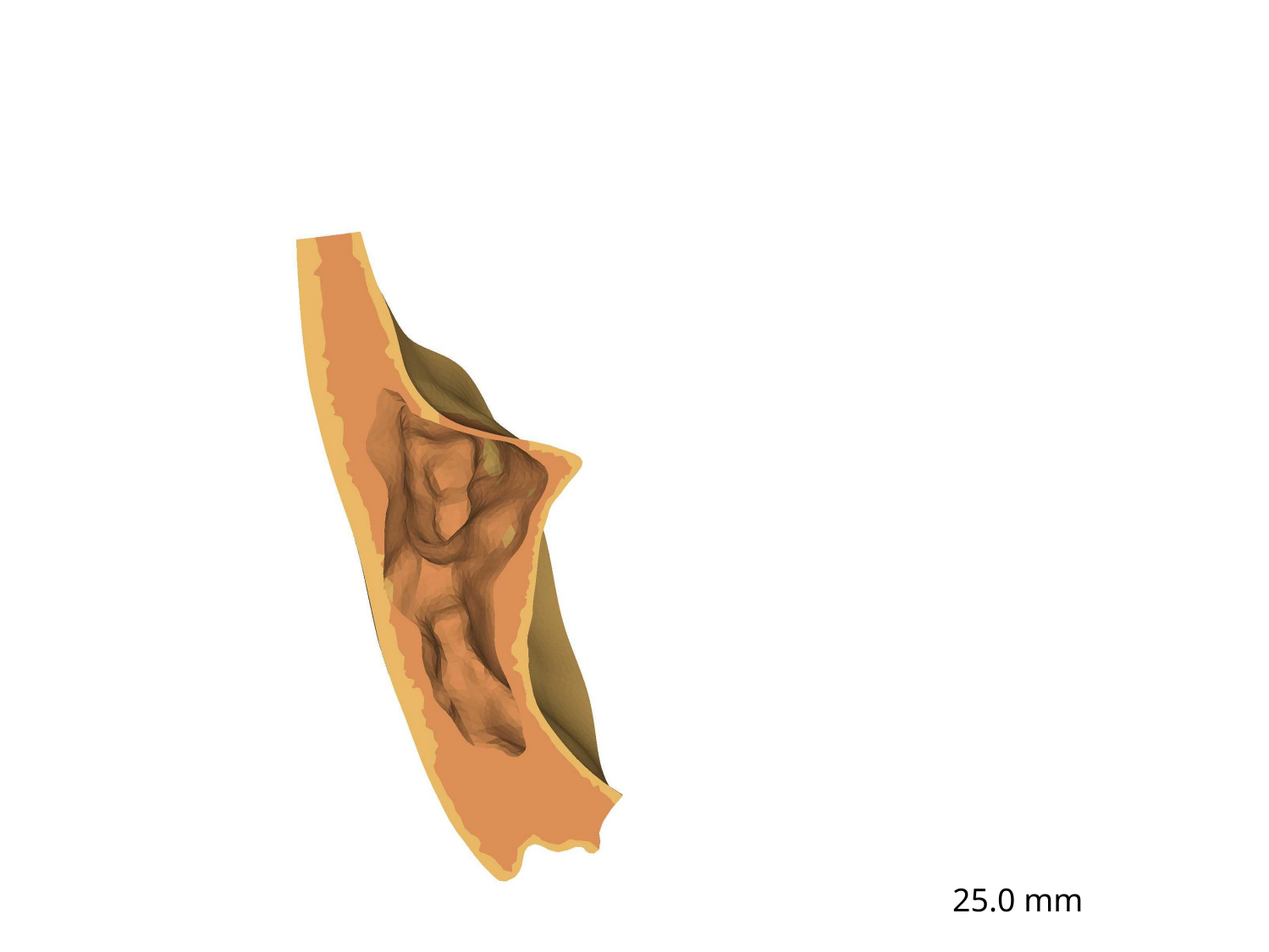

25.0 mm

## Slide 129
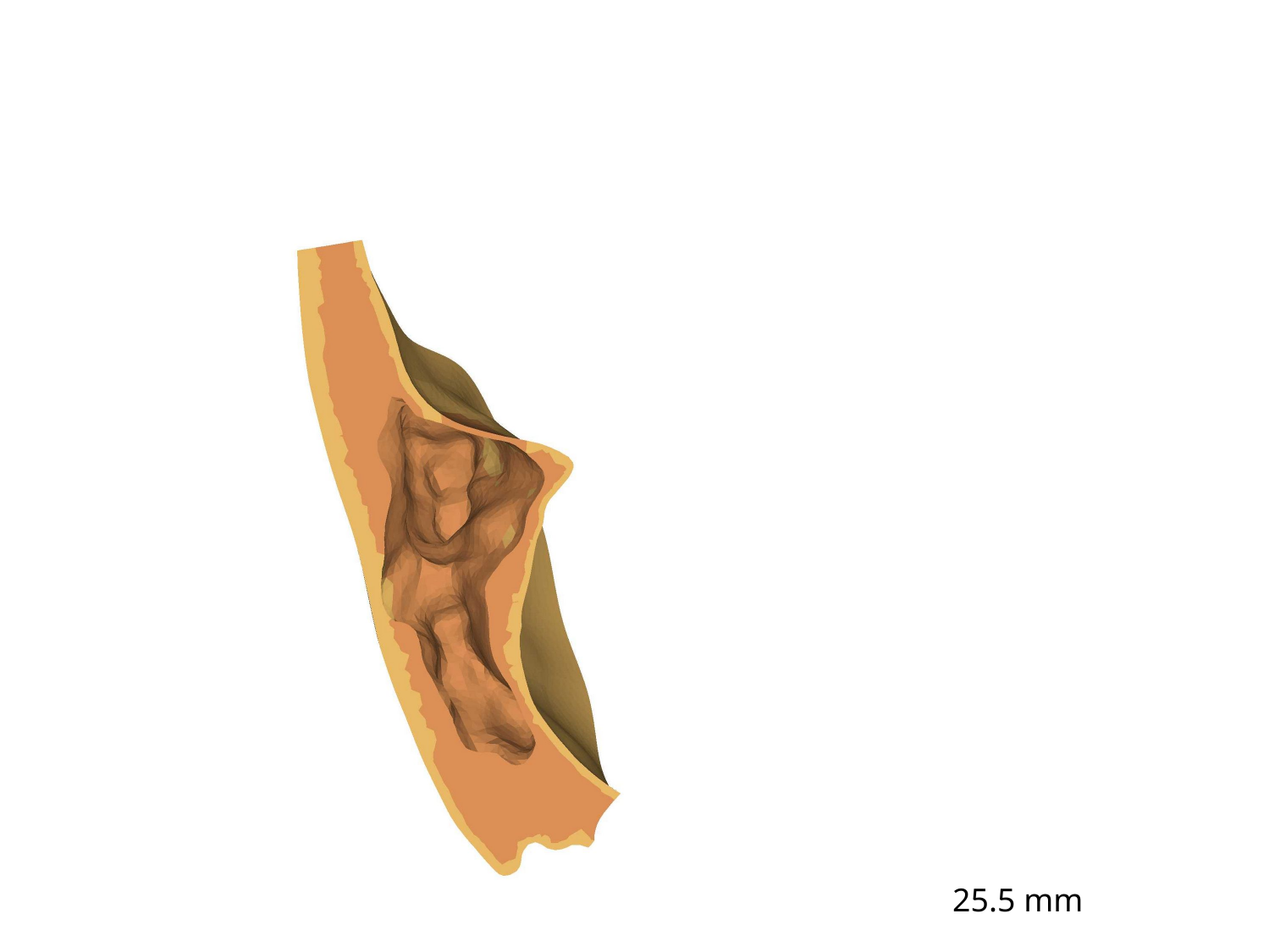

25.5 mm

## Slide 130
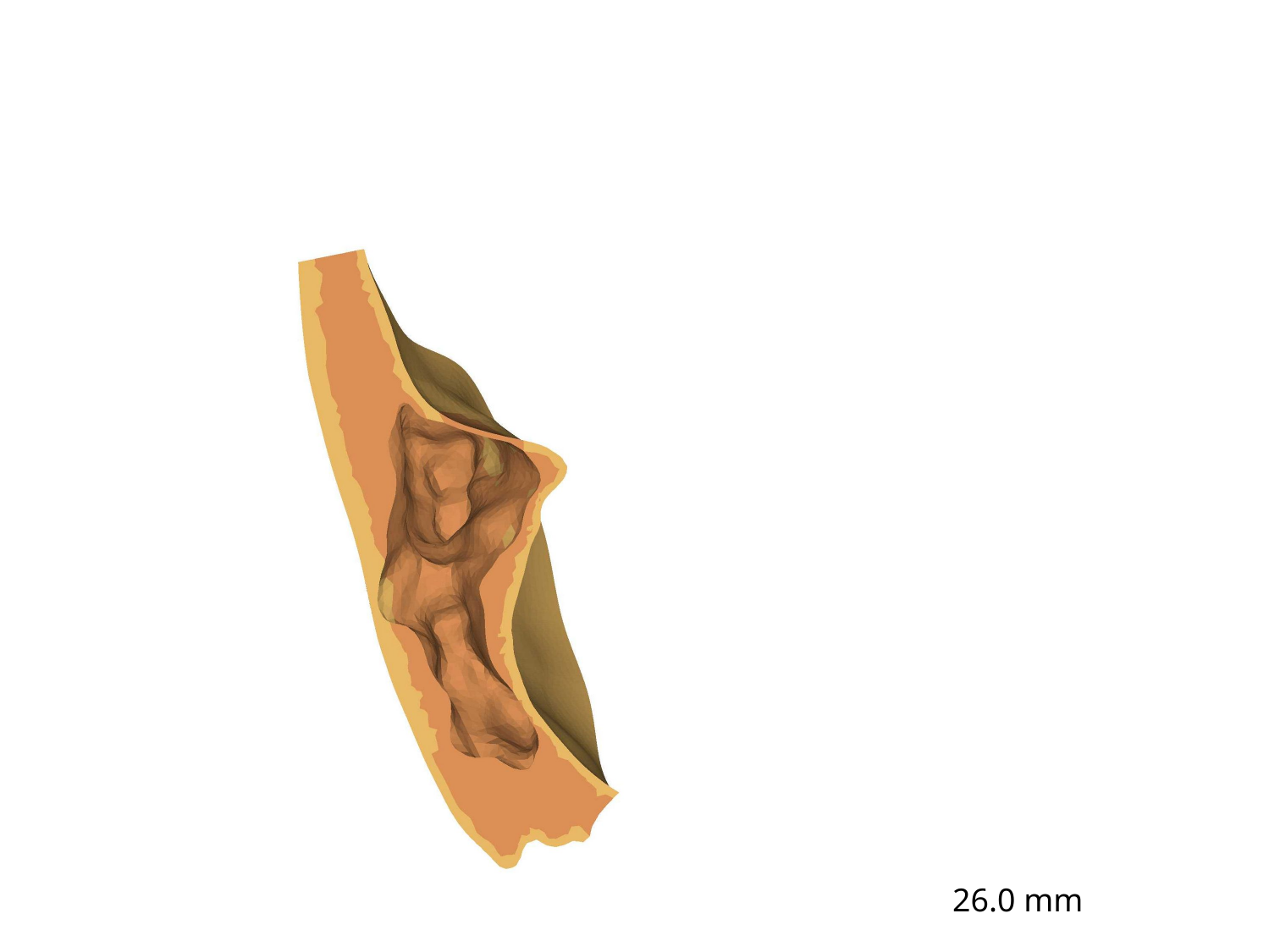

26.0 mm

## Slide 131
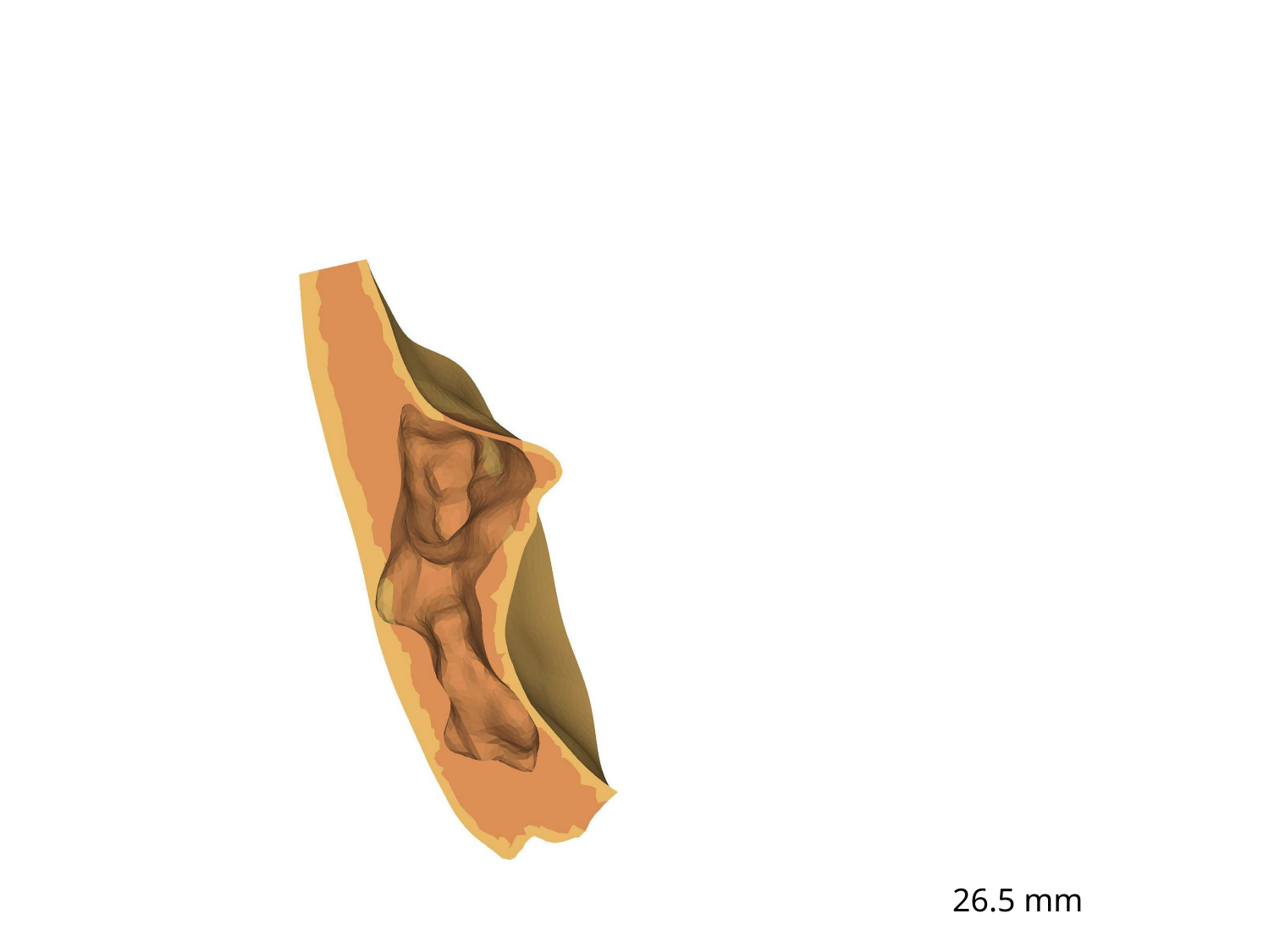

26.5 mm

## Slide 132
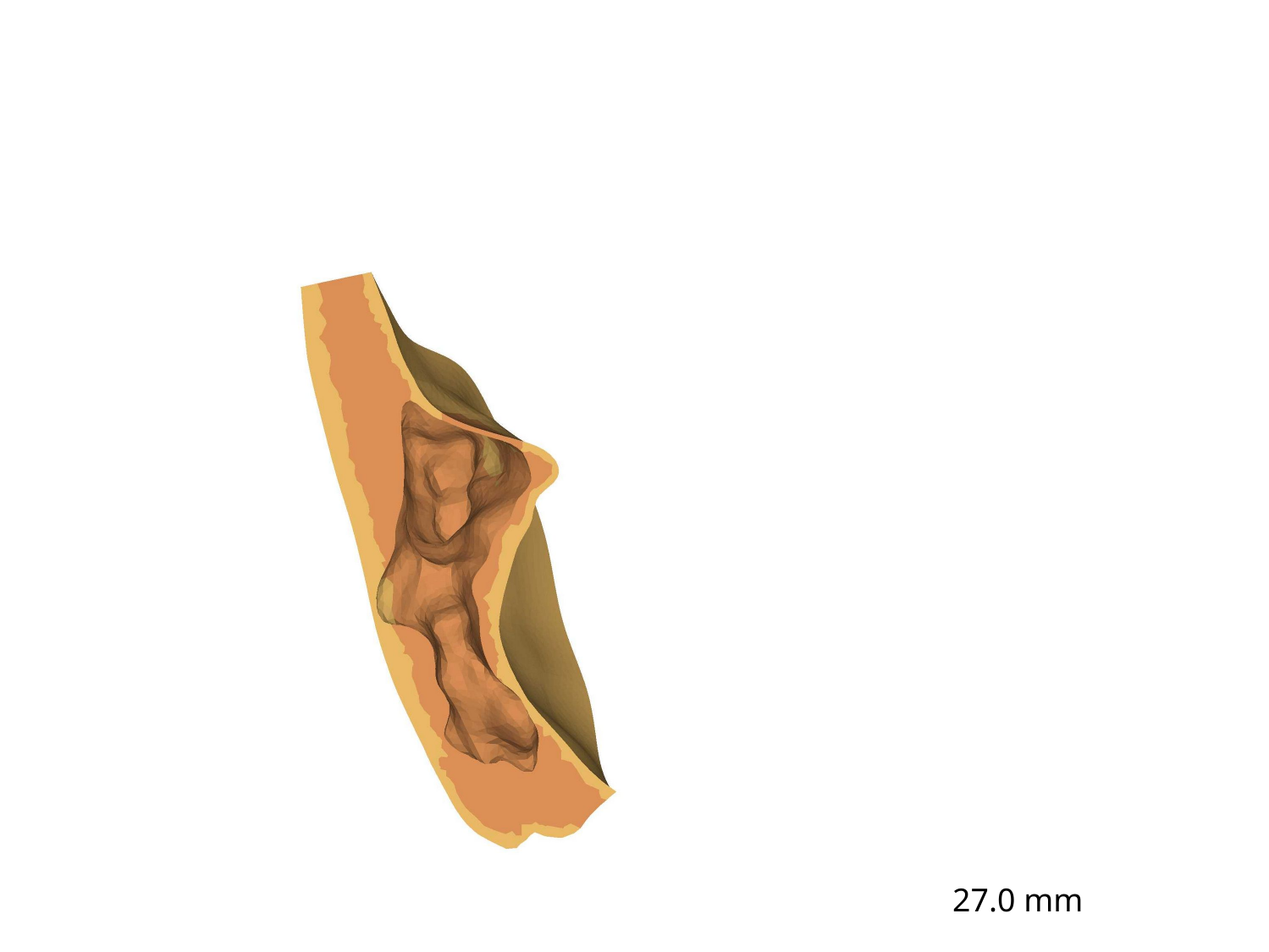

27.0 mm

## Slide 133
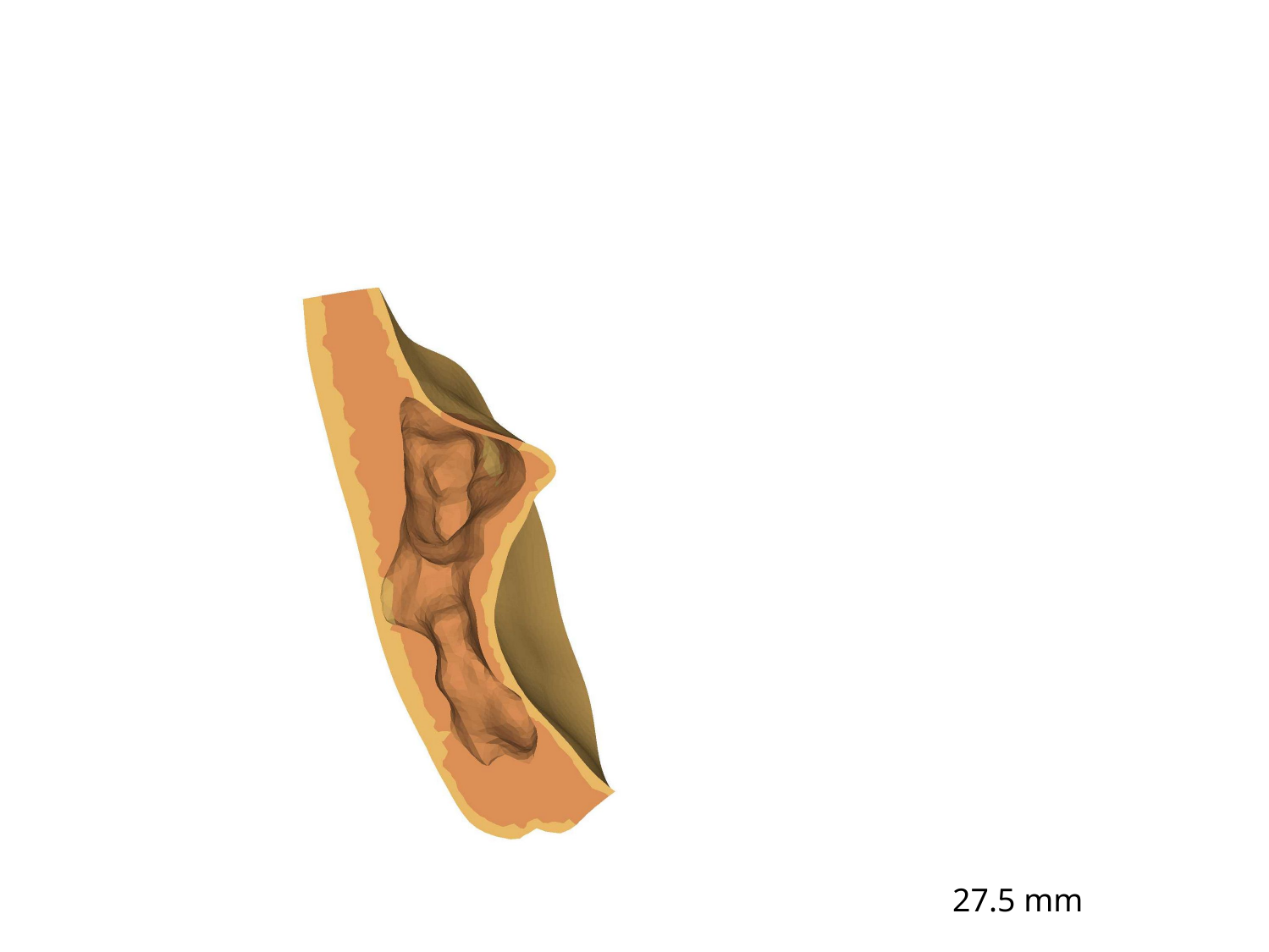

27.5 mm

## Slide 134
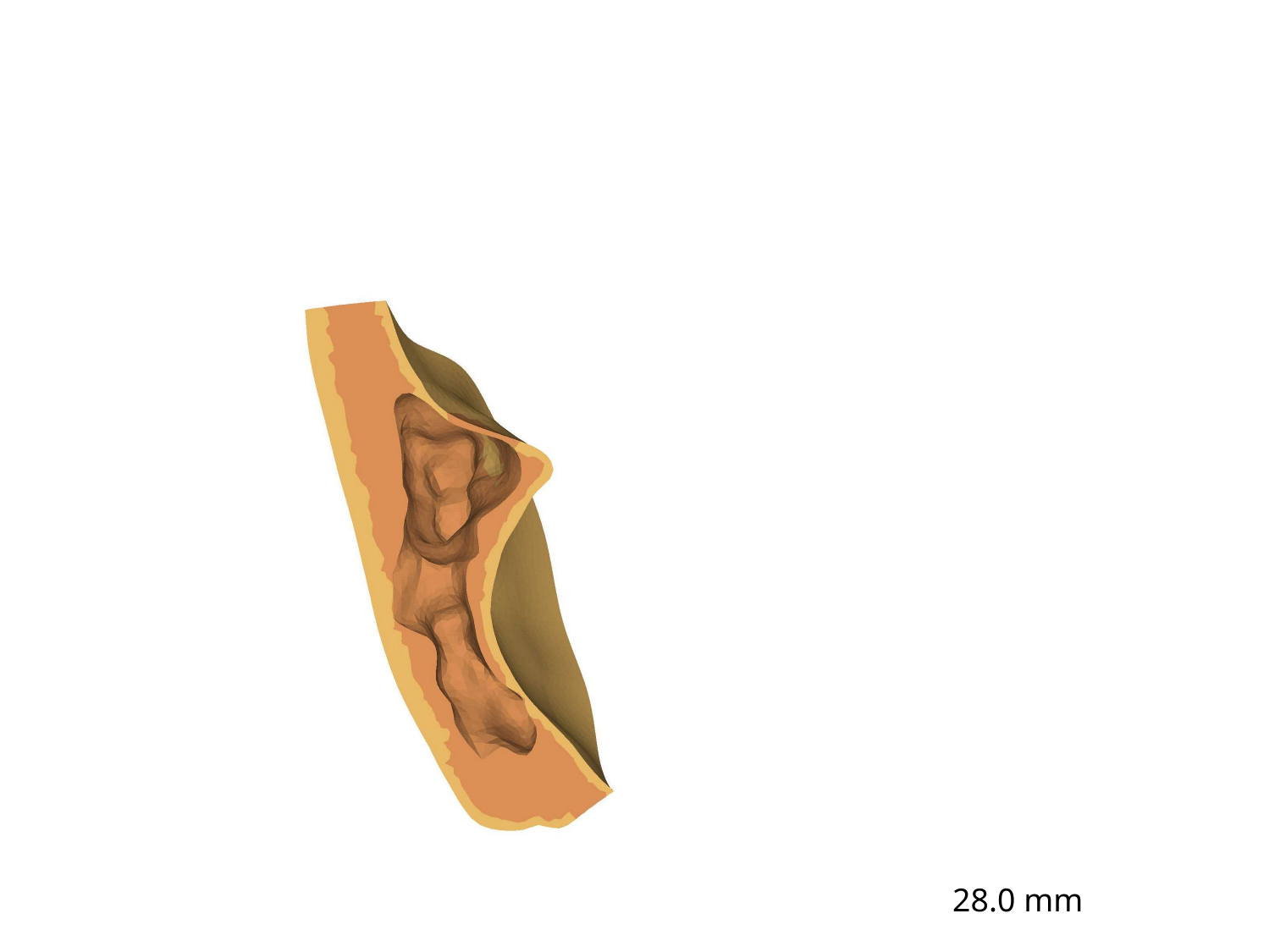

28.0 mm

## Slide 135
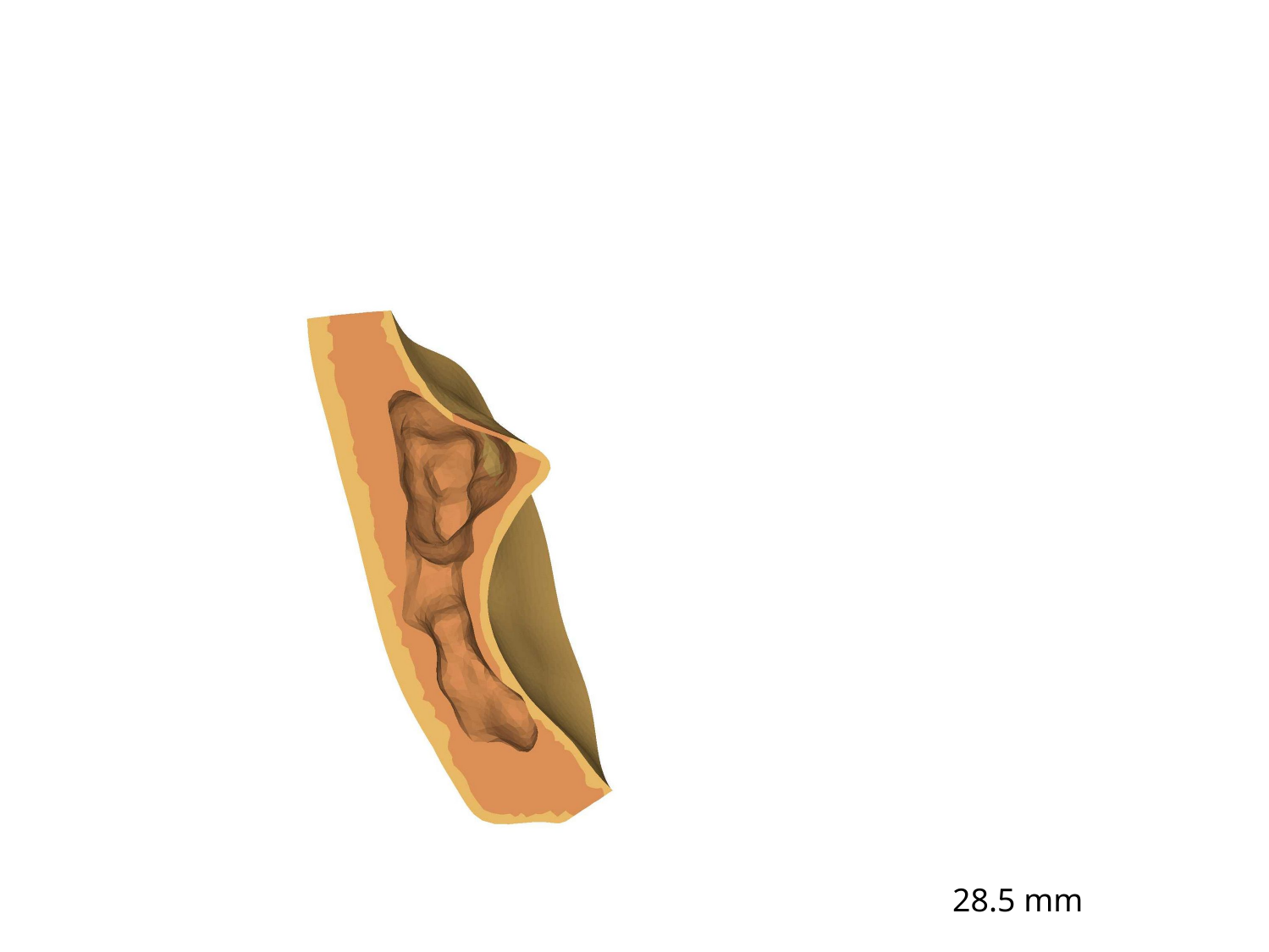

28.5 mm

## Slide 136
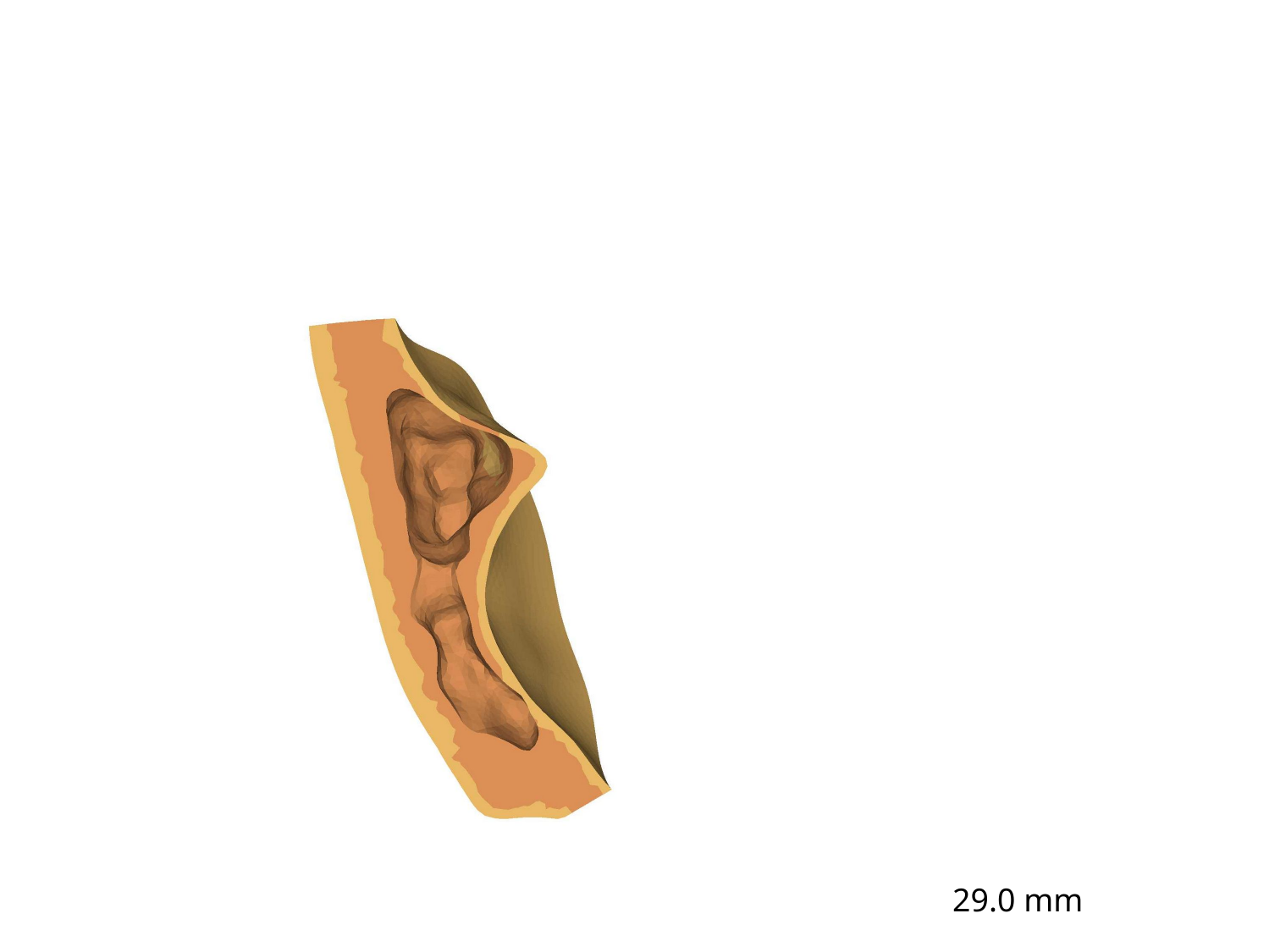

29.0 mm

## Slide 137
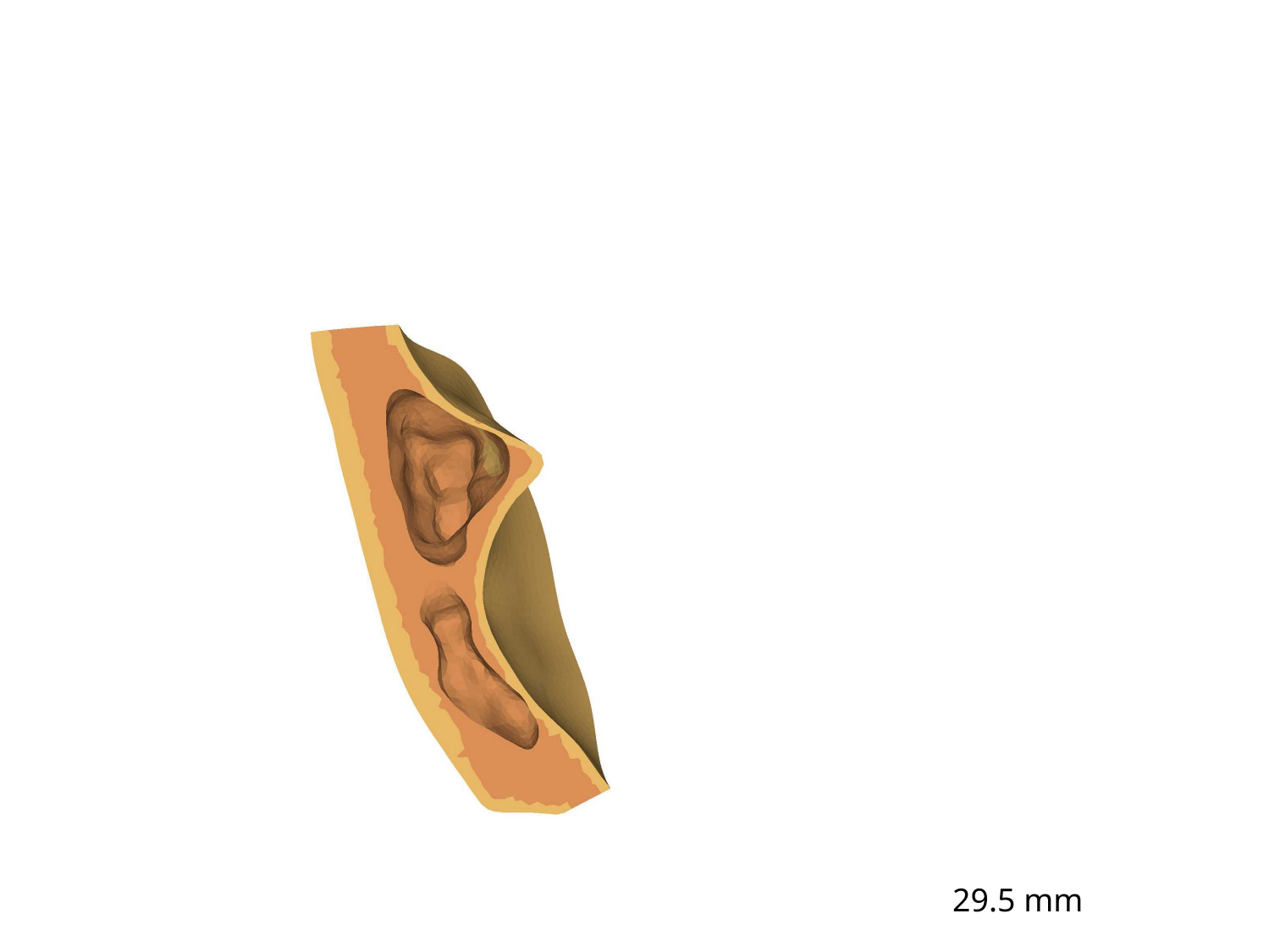

29.5 mm

## Slide 138
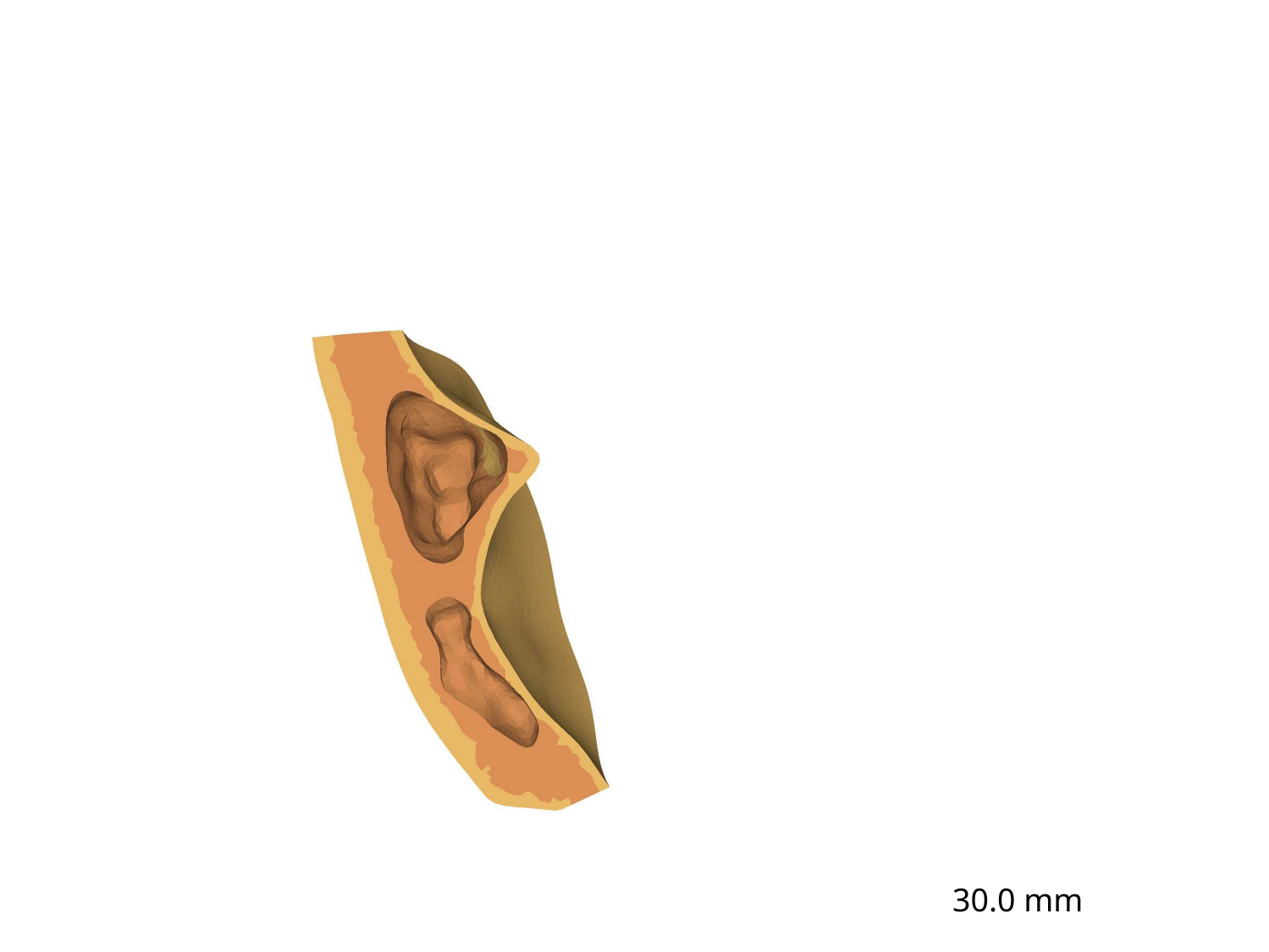

30.0 mm

## Slide 139
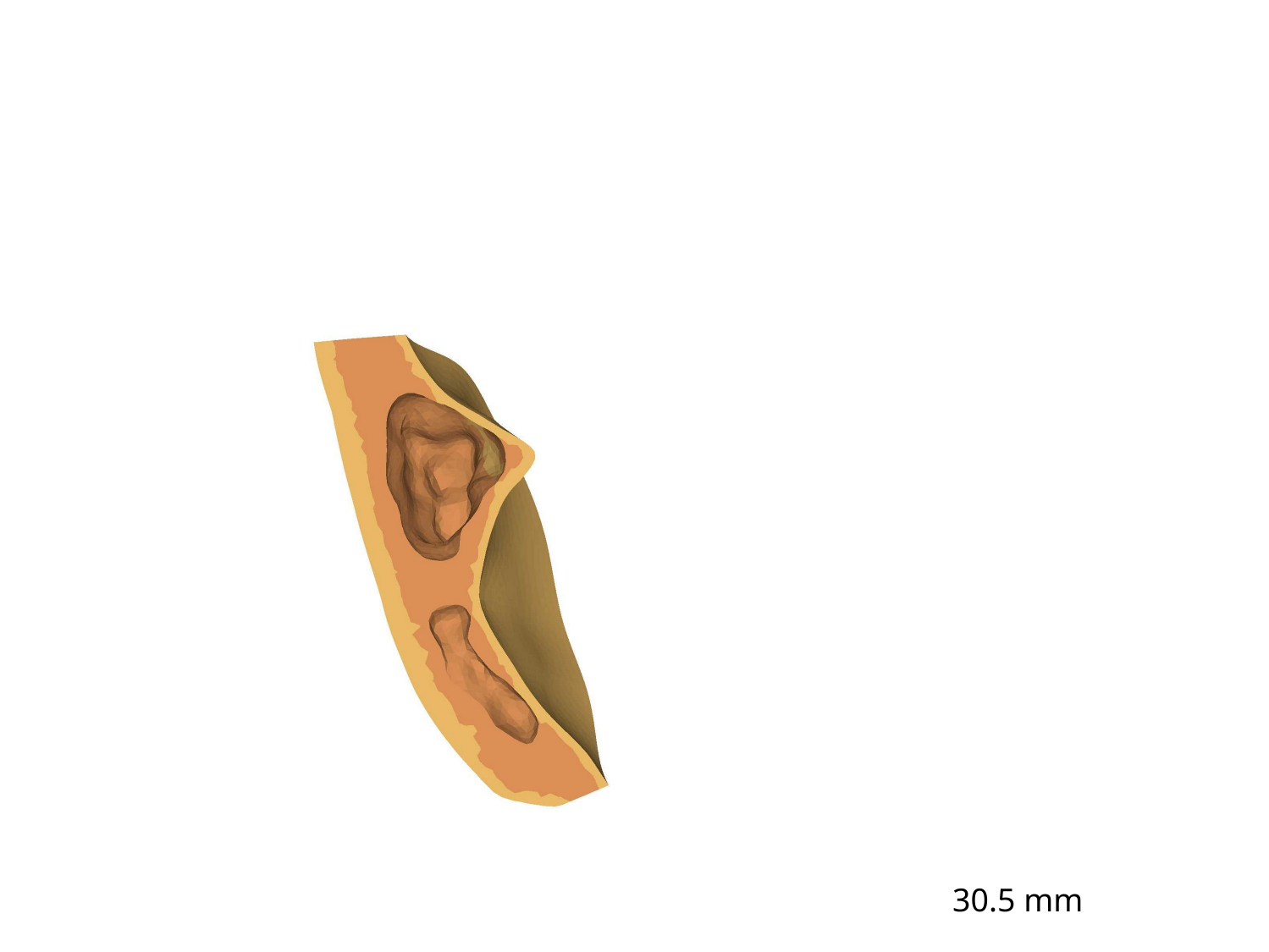

30.5 mm

## Slide 140
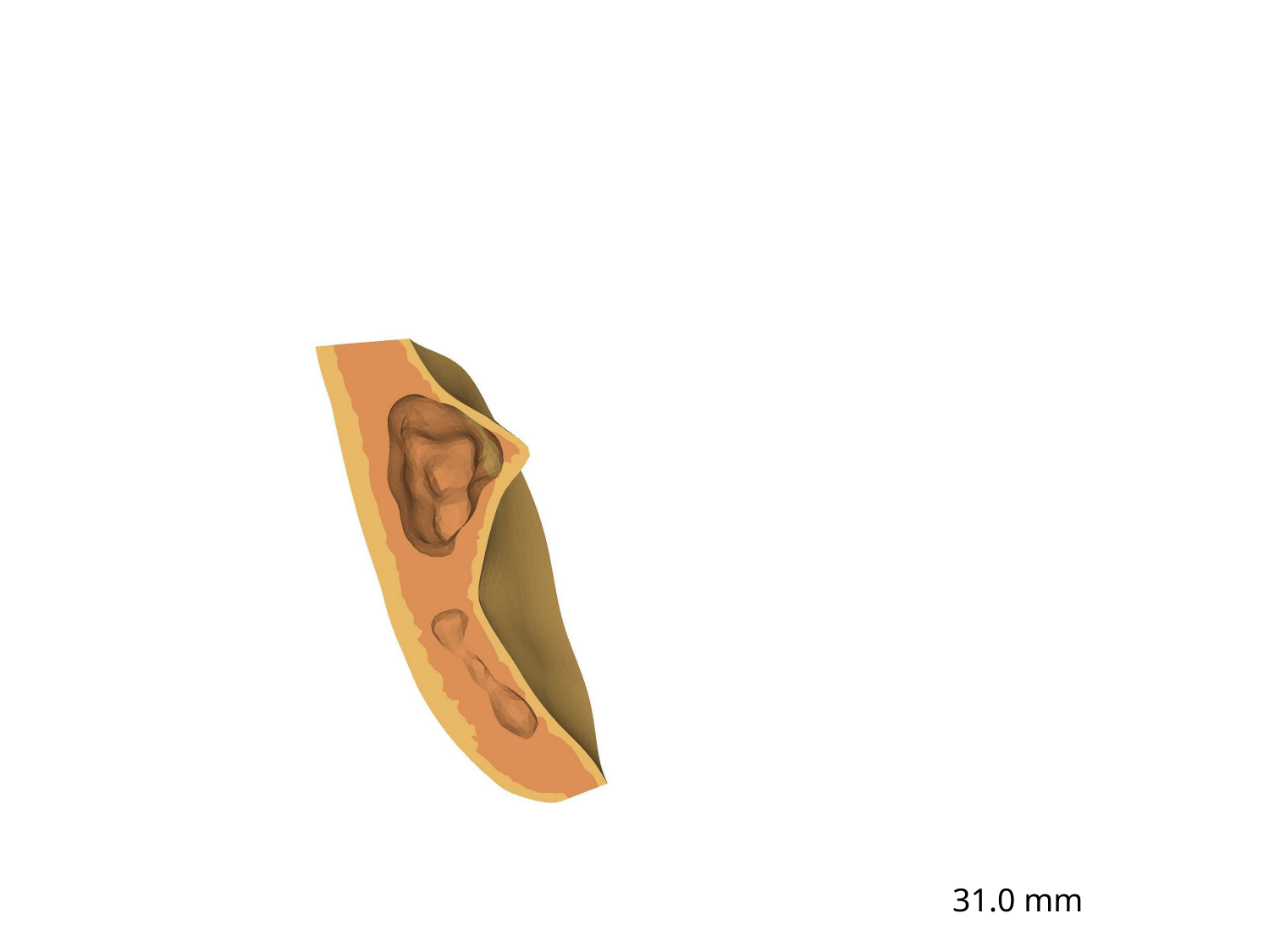

31.0 mm

## Slide 141
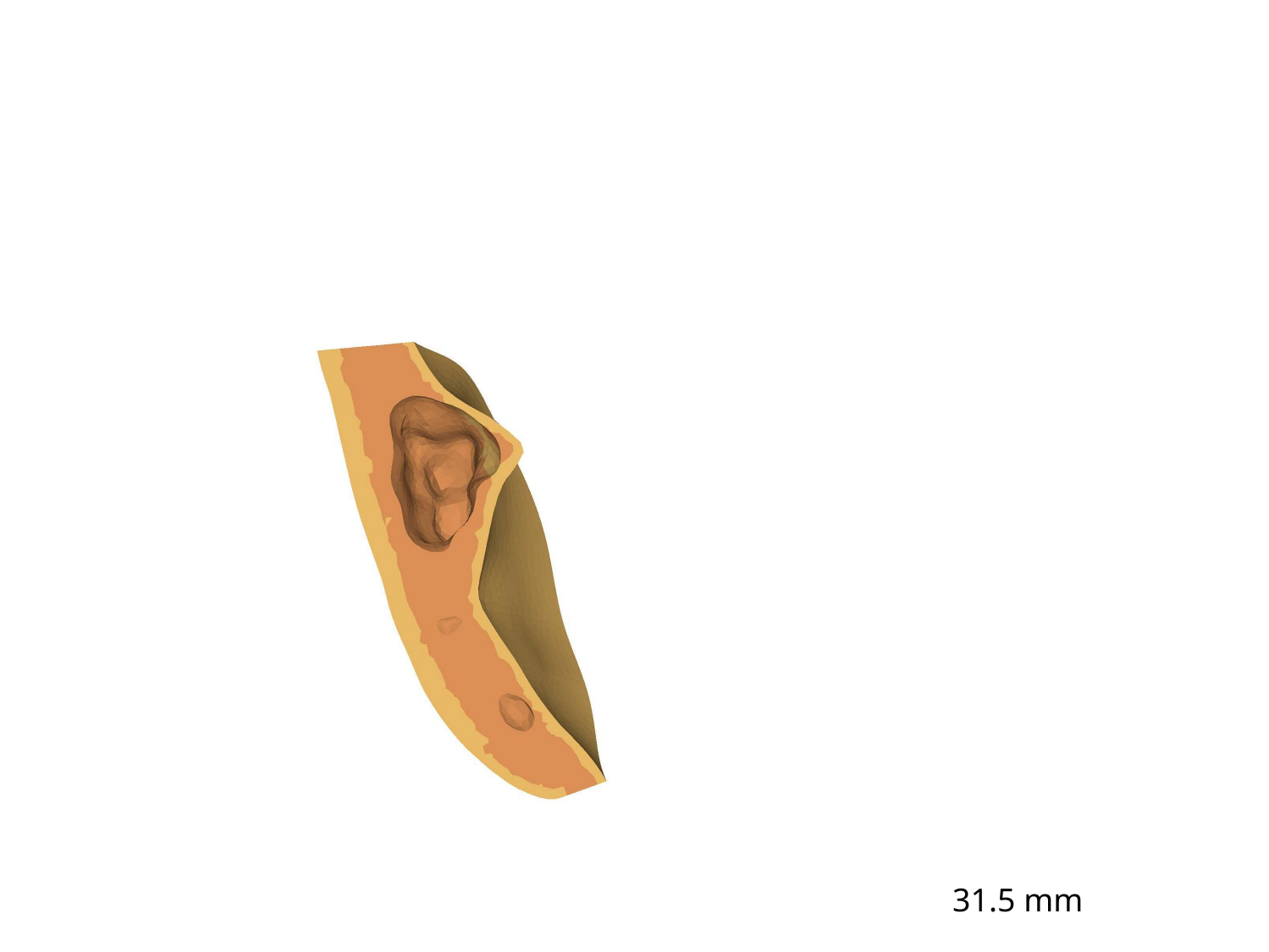

31.5 mm

## Slide 142
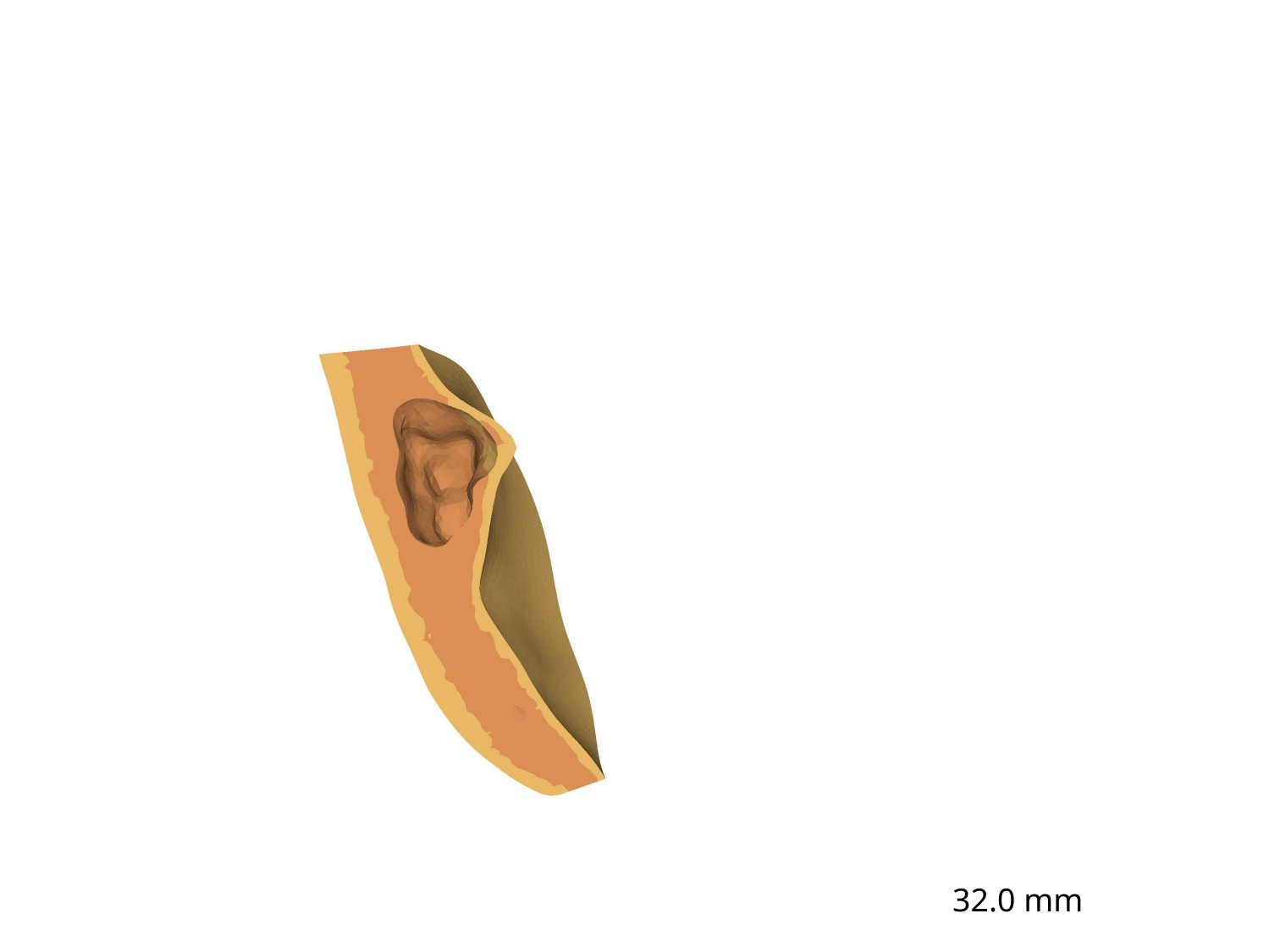

32.0 mm

## Slide 143
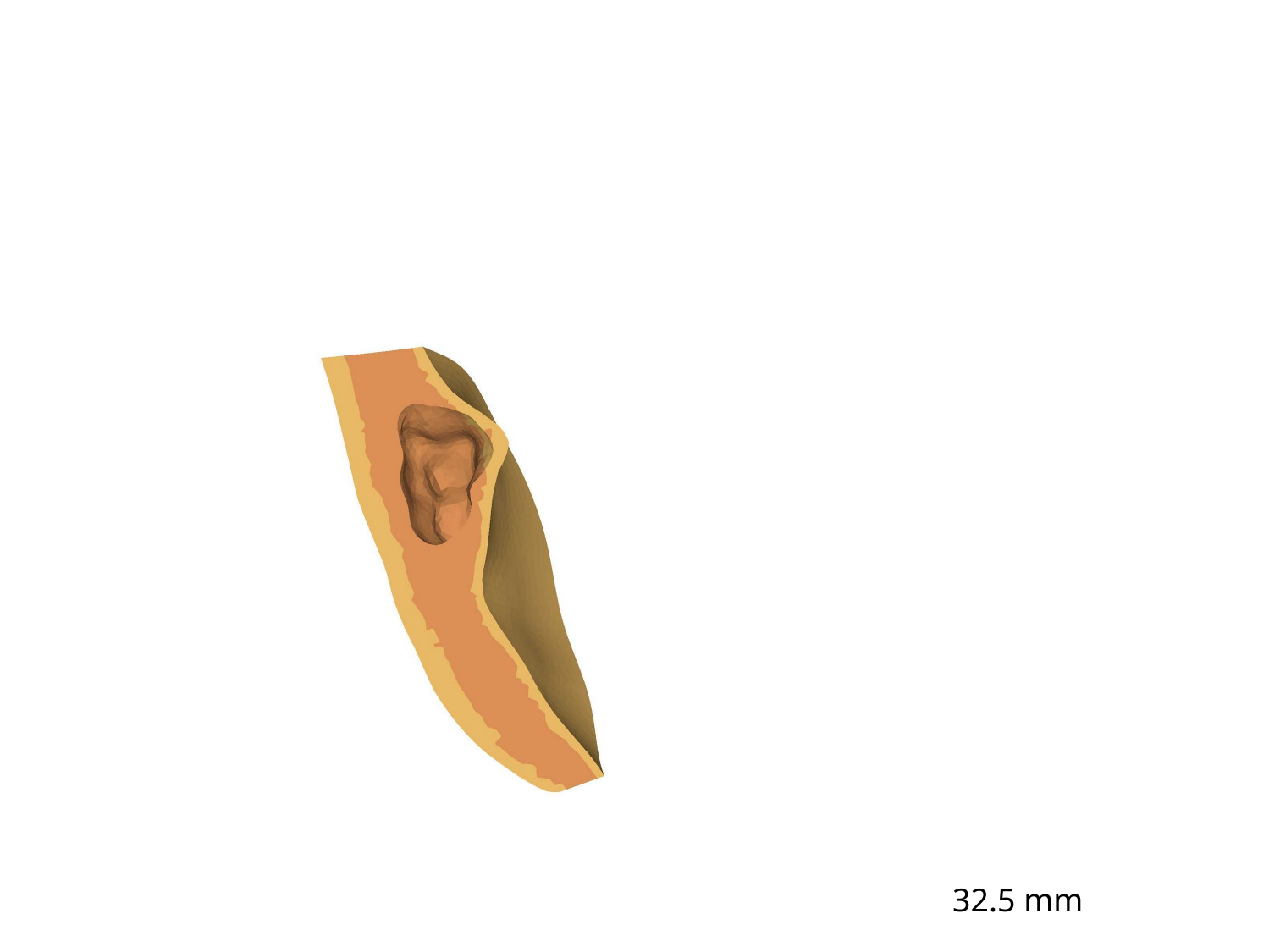

32.5 mm

## Slide 144
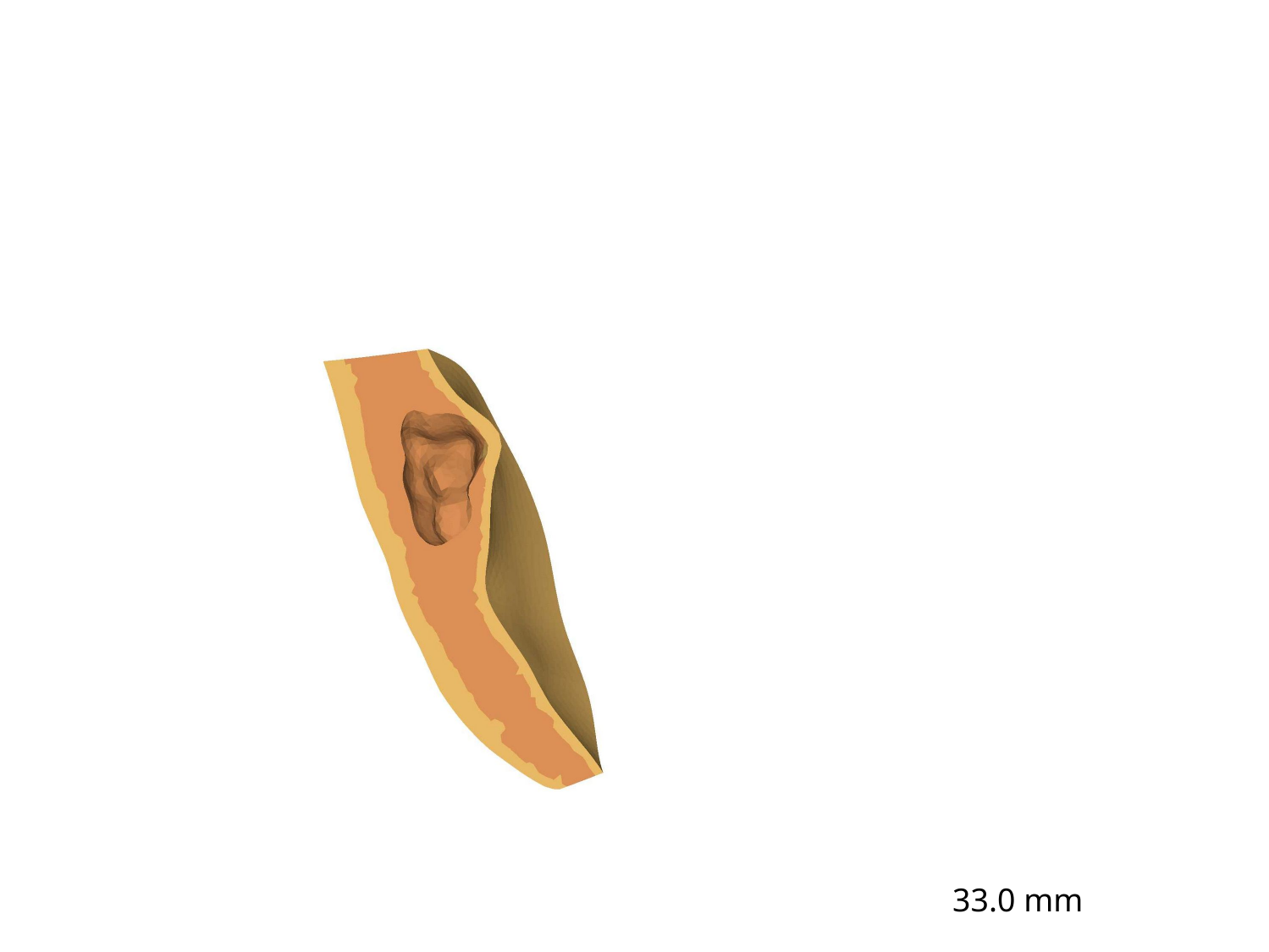

33.0 mm

## Slide 145
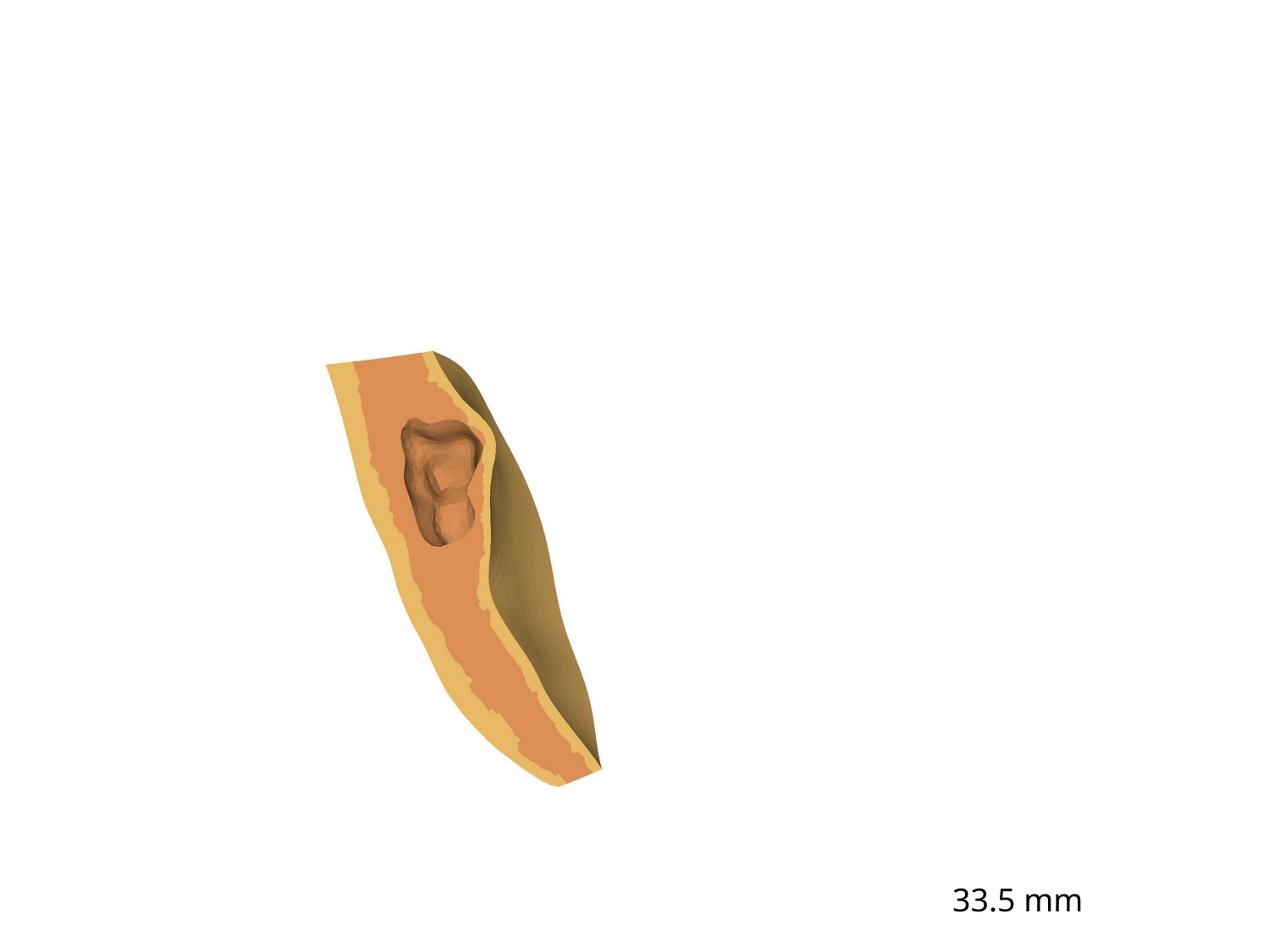

33.5 mm

## Slide 146
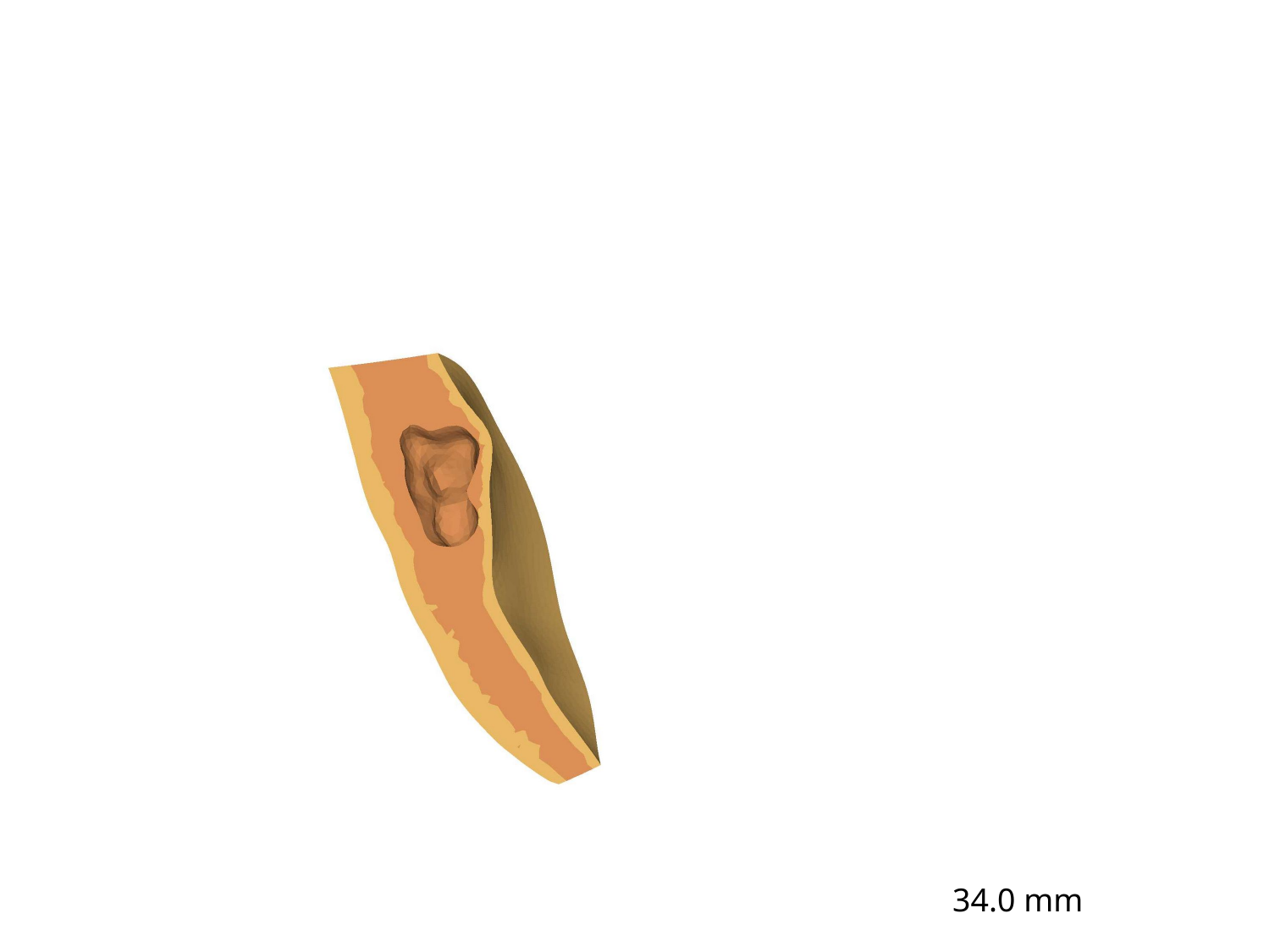

34.0 mm

## Slide 147
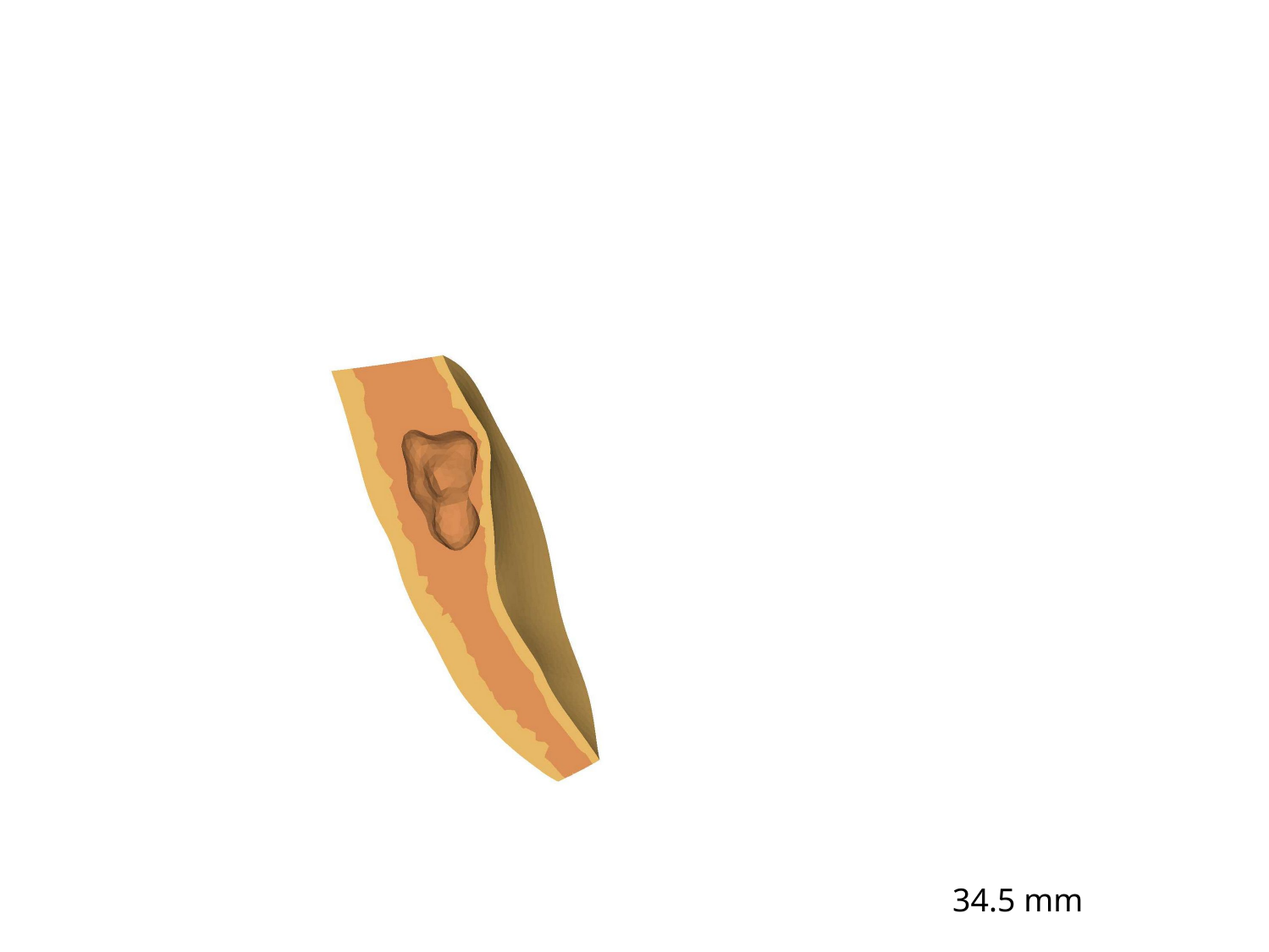

34.5 mm

## Slide 148
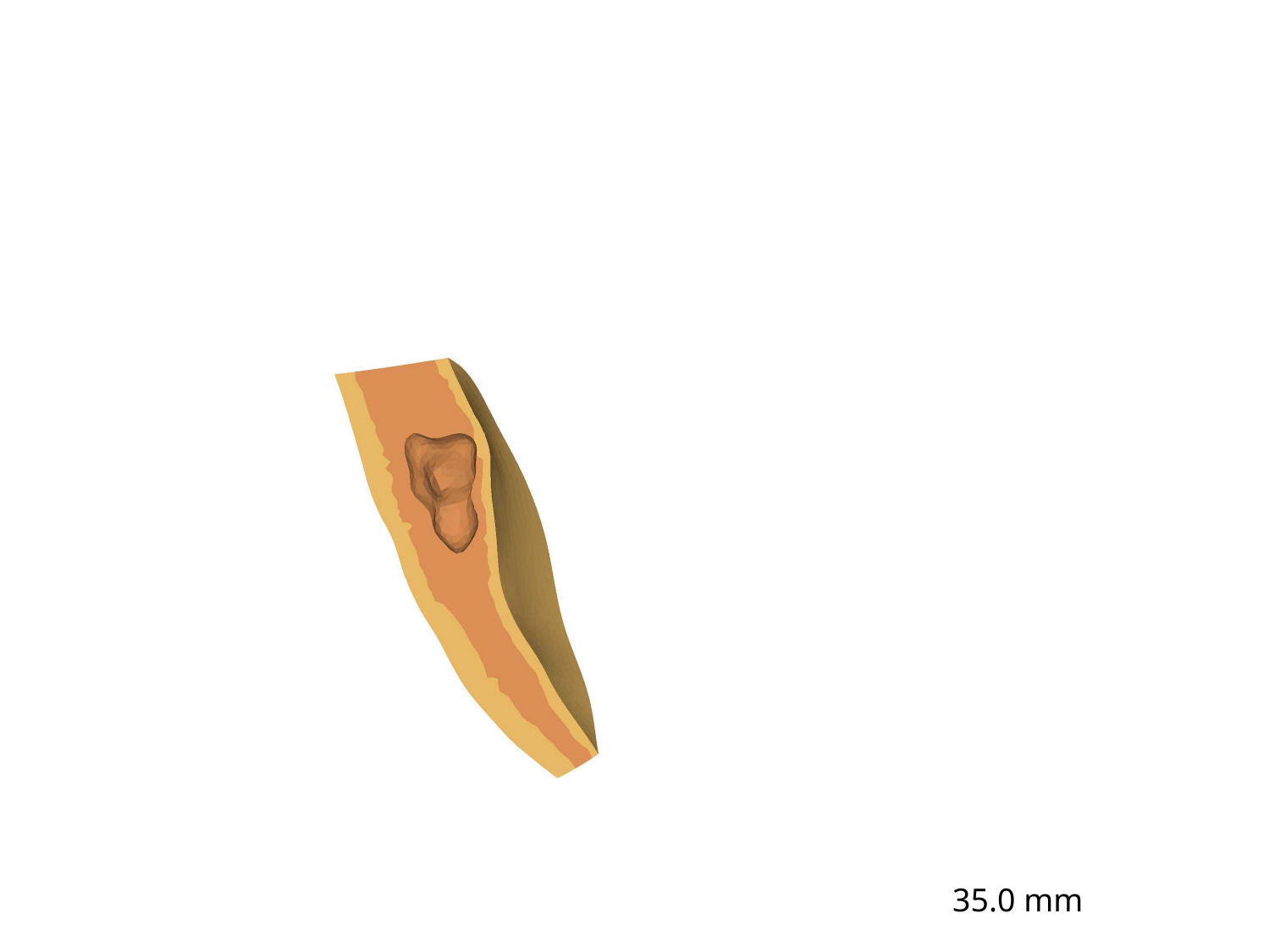

35.0 mm

## Slide 149
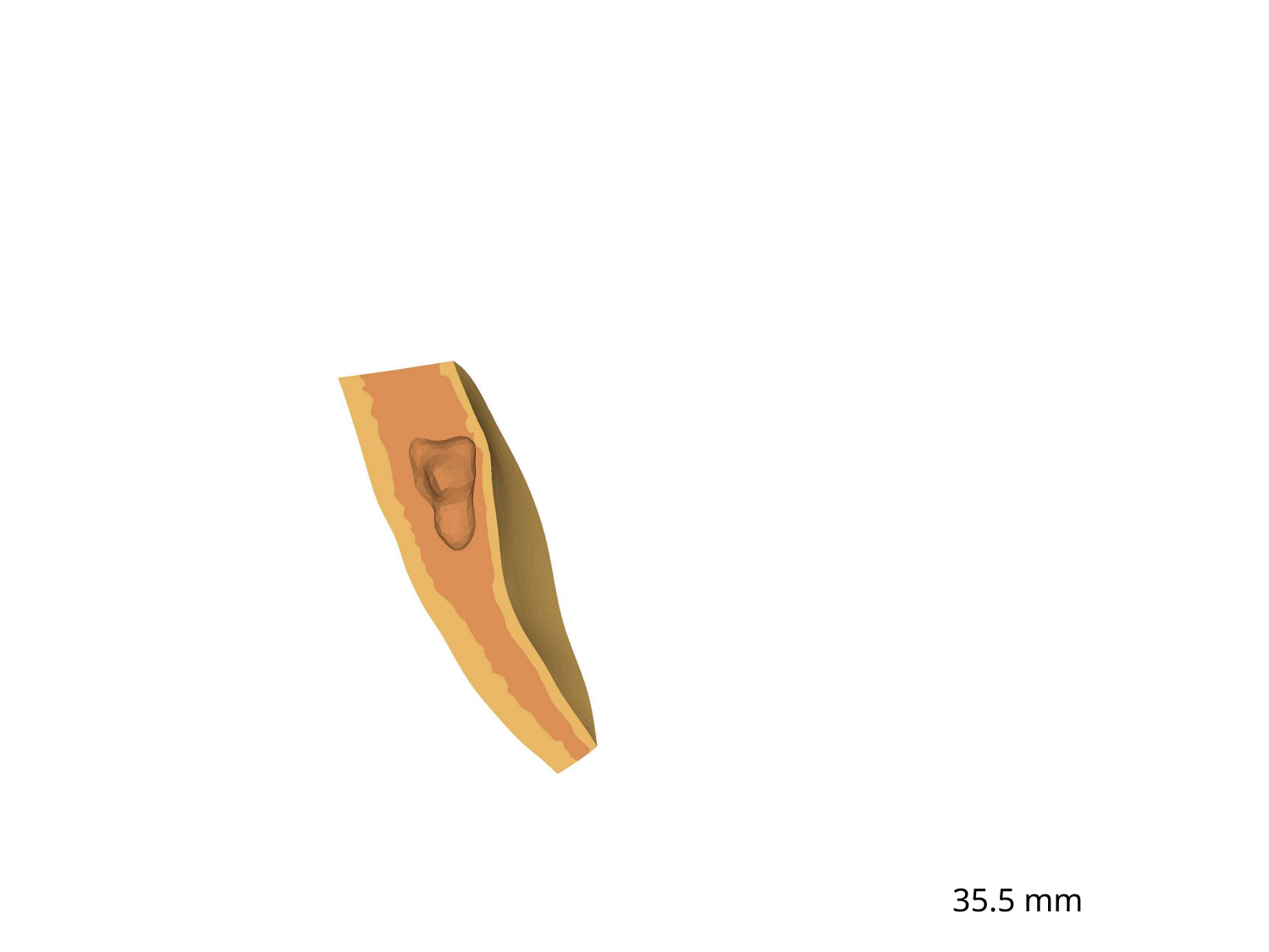

35.5 mm

## Slide 150
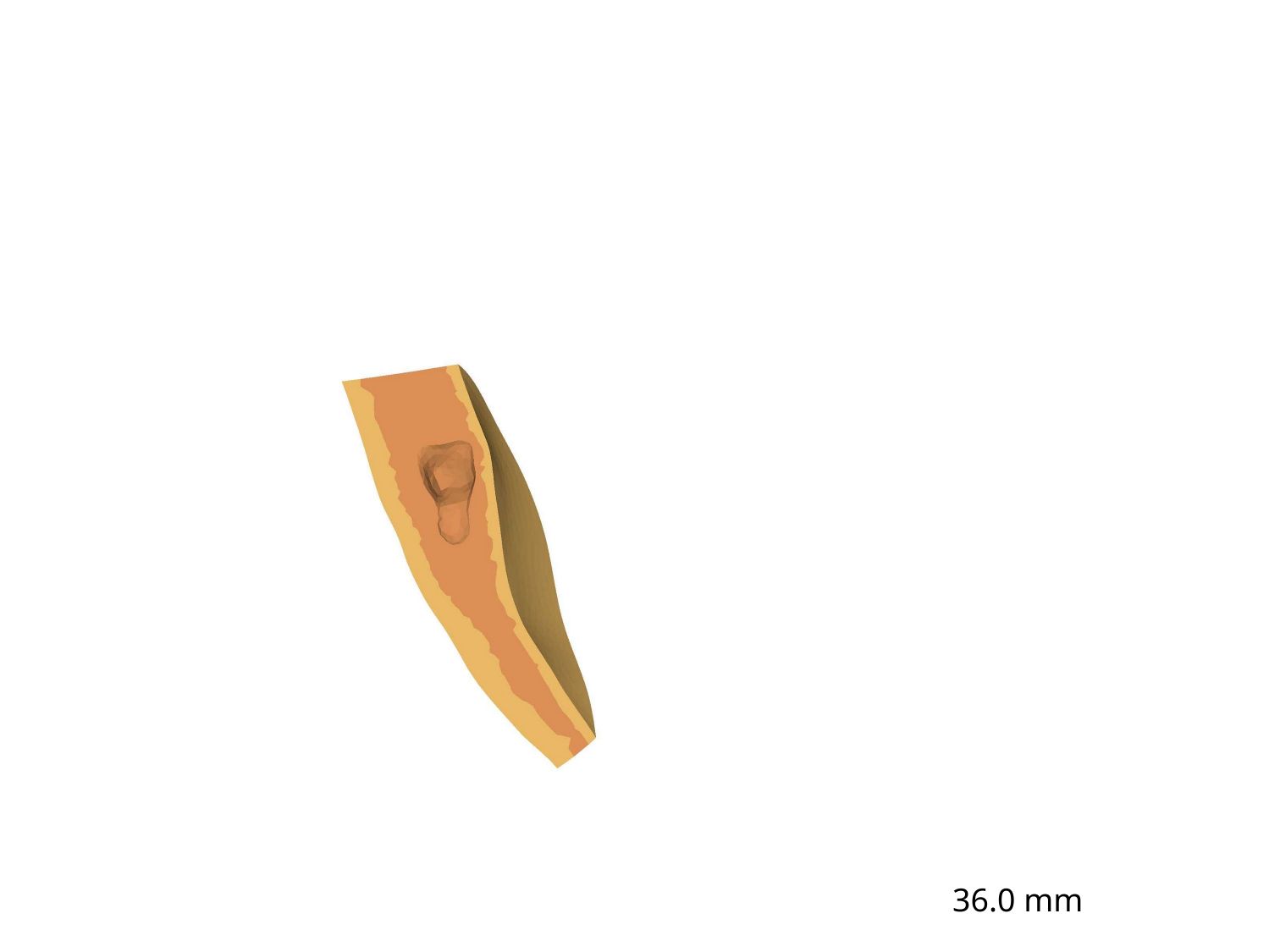

36.0 mm

## Slide 151
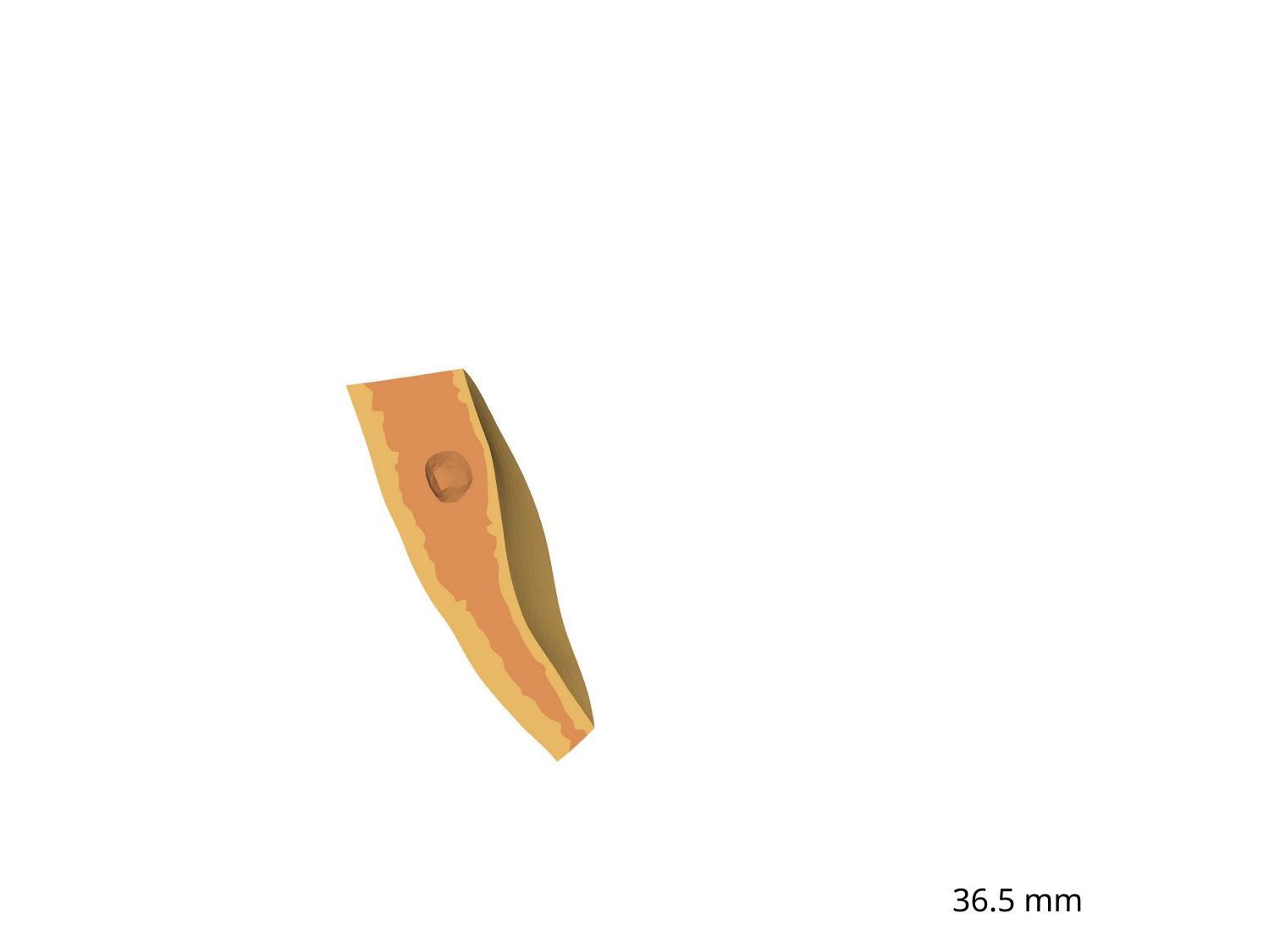

36.5 mm

## Slide 152
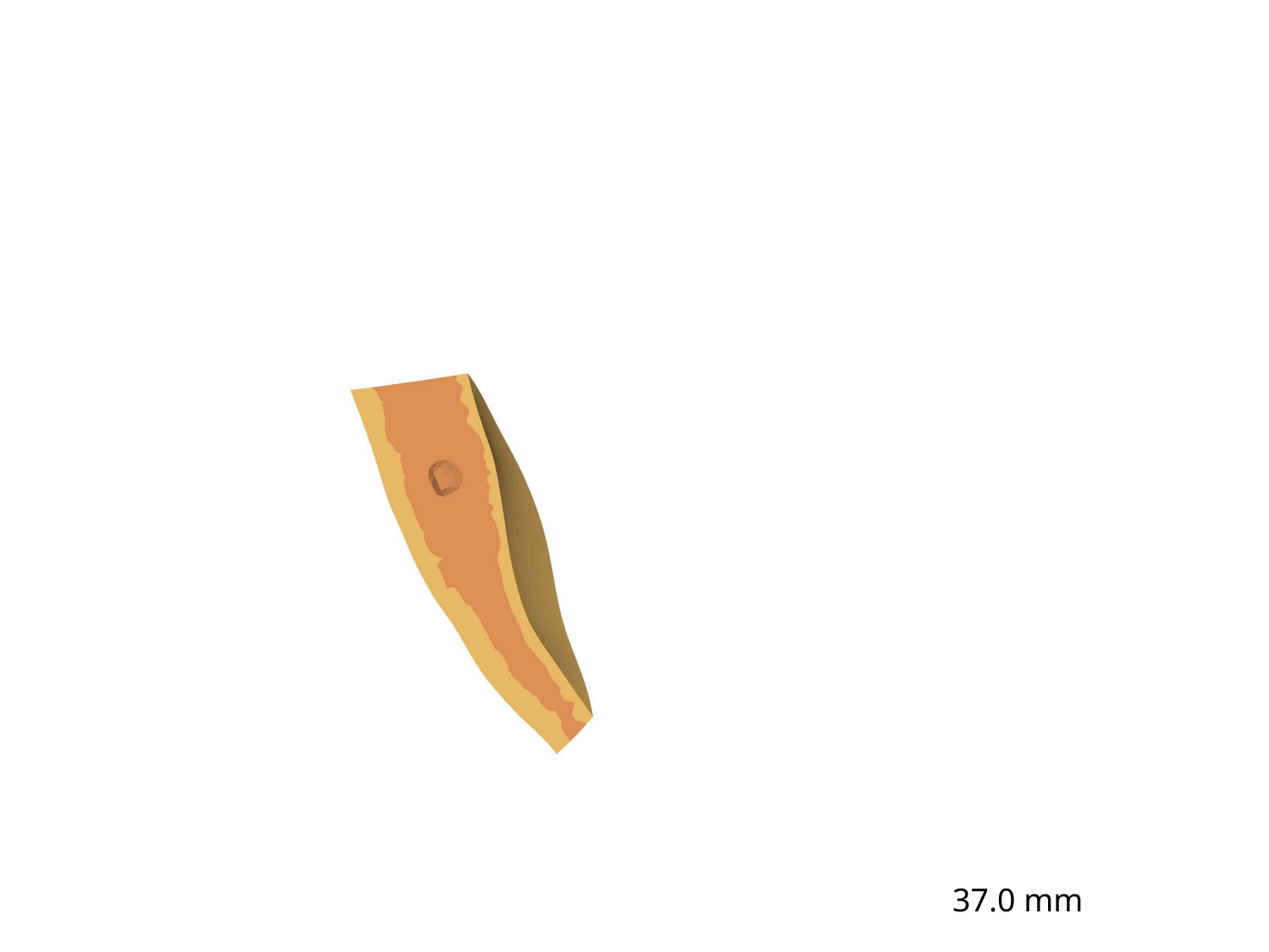

37.0 mm

## Slide 153
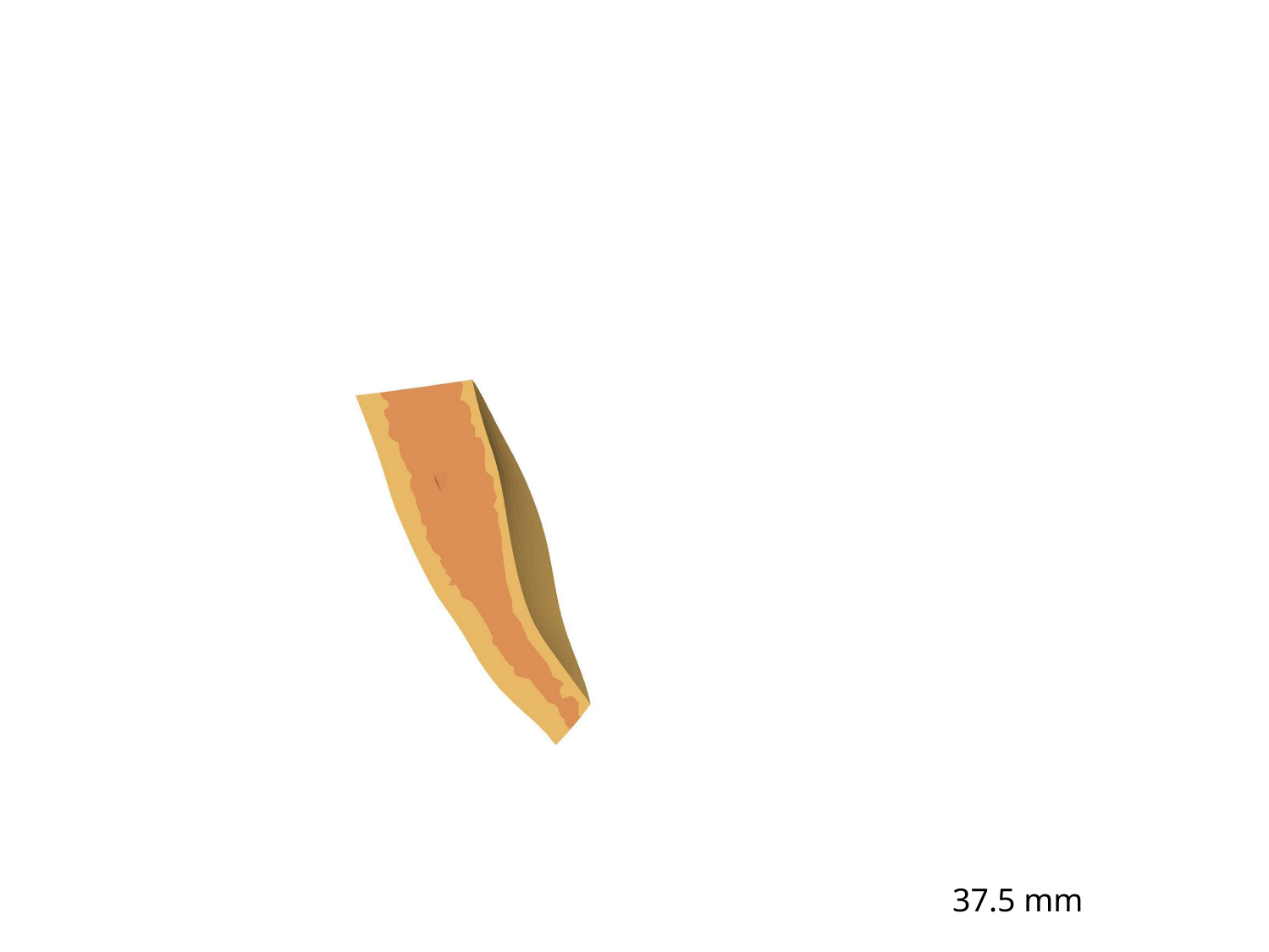

37.5 mm

## Slide 154
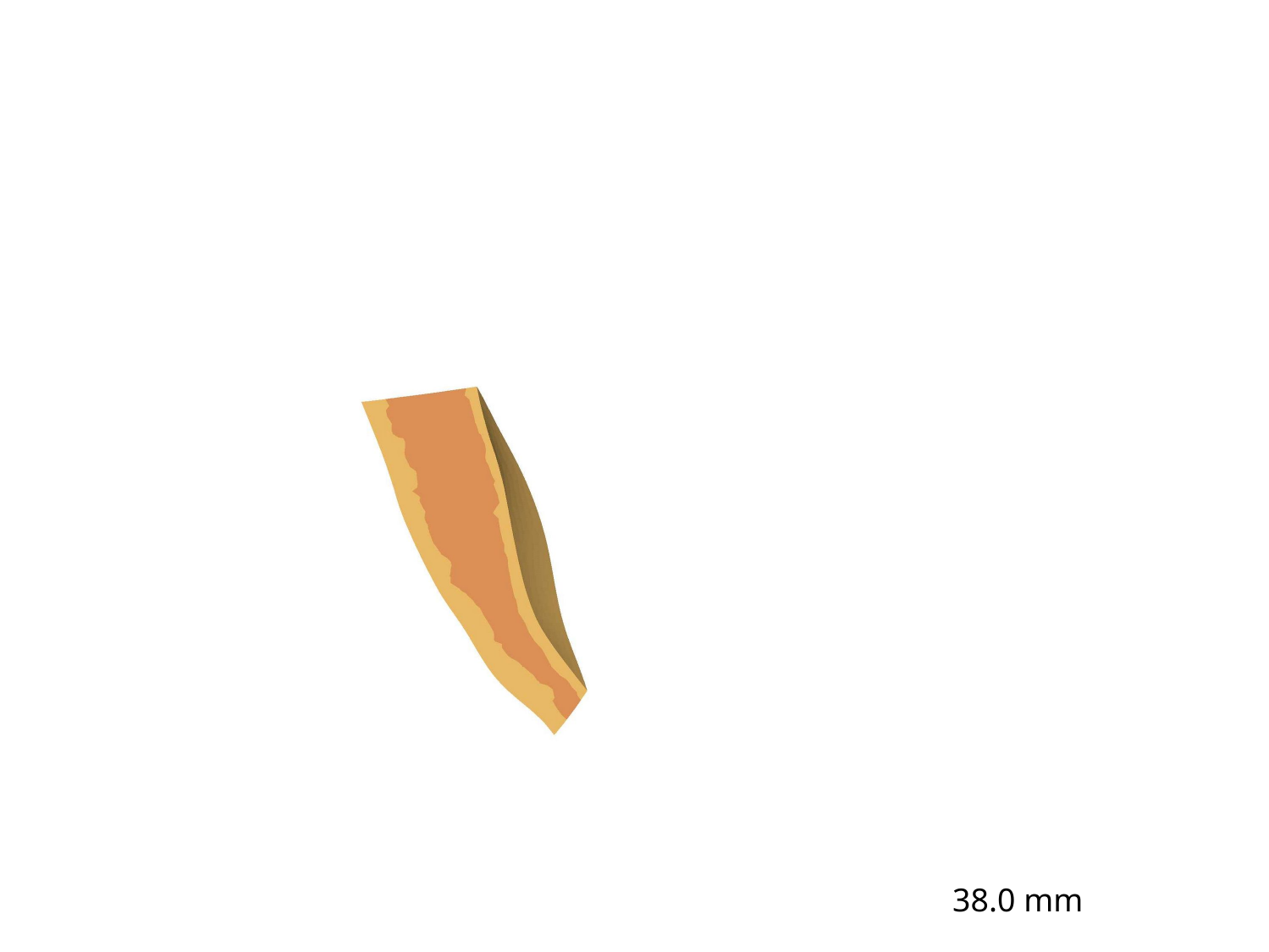

38.0 mm

## Slide 155
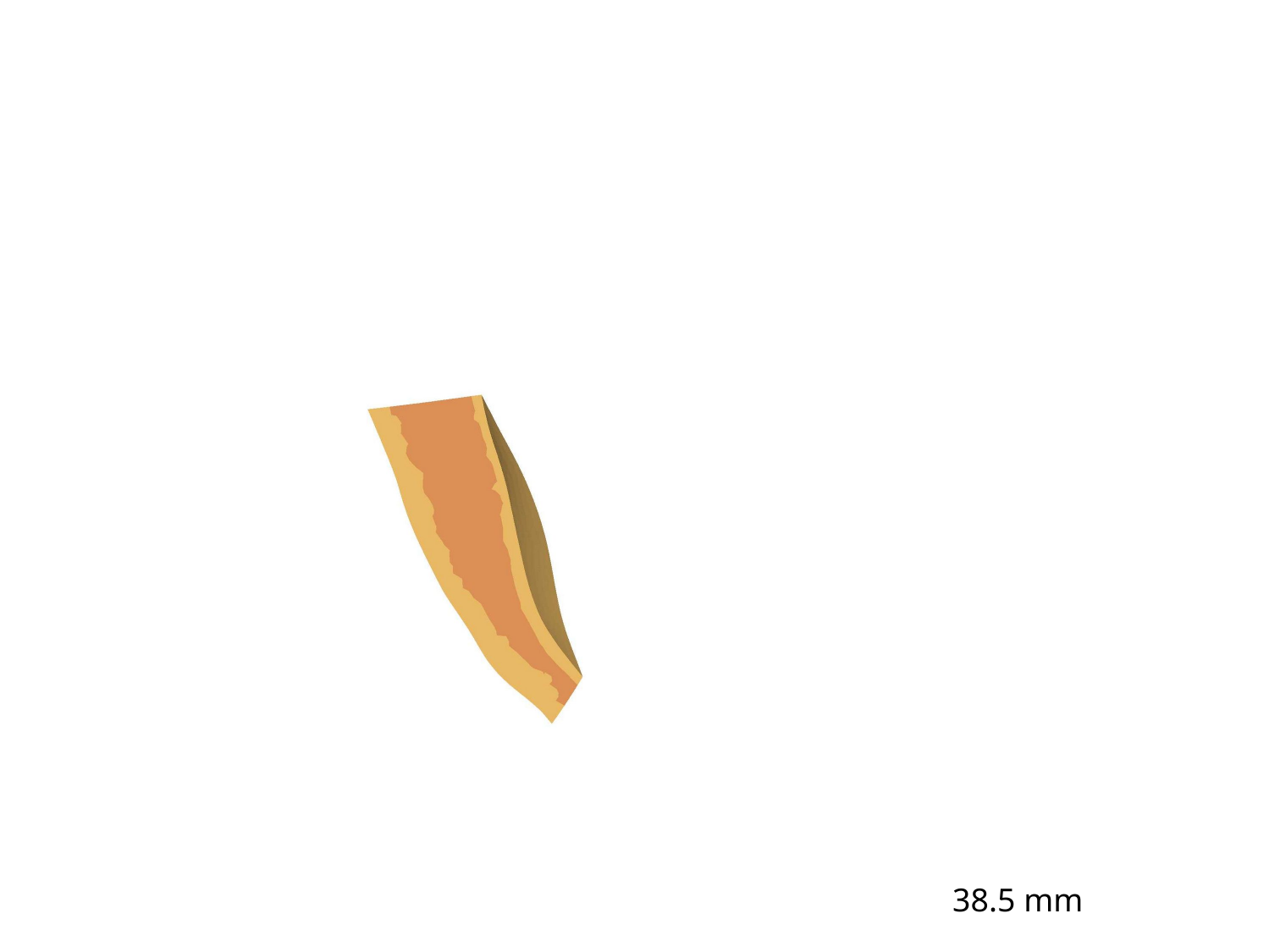

38.5 mm

## Slide 156
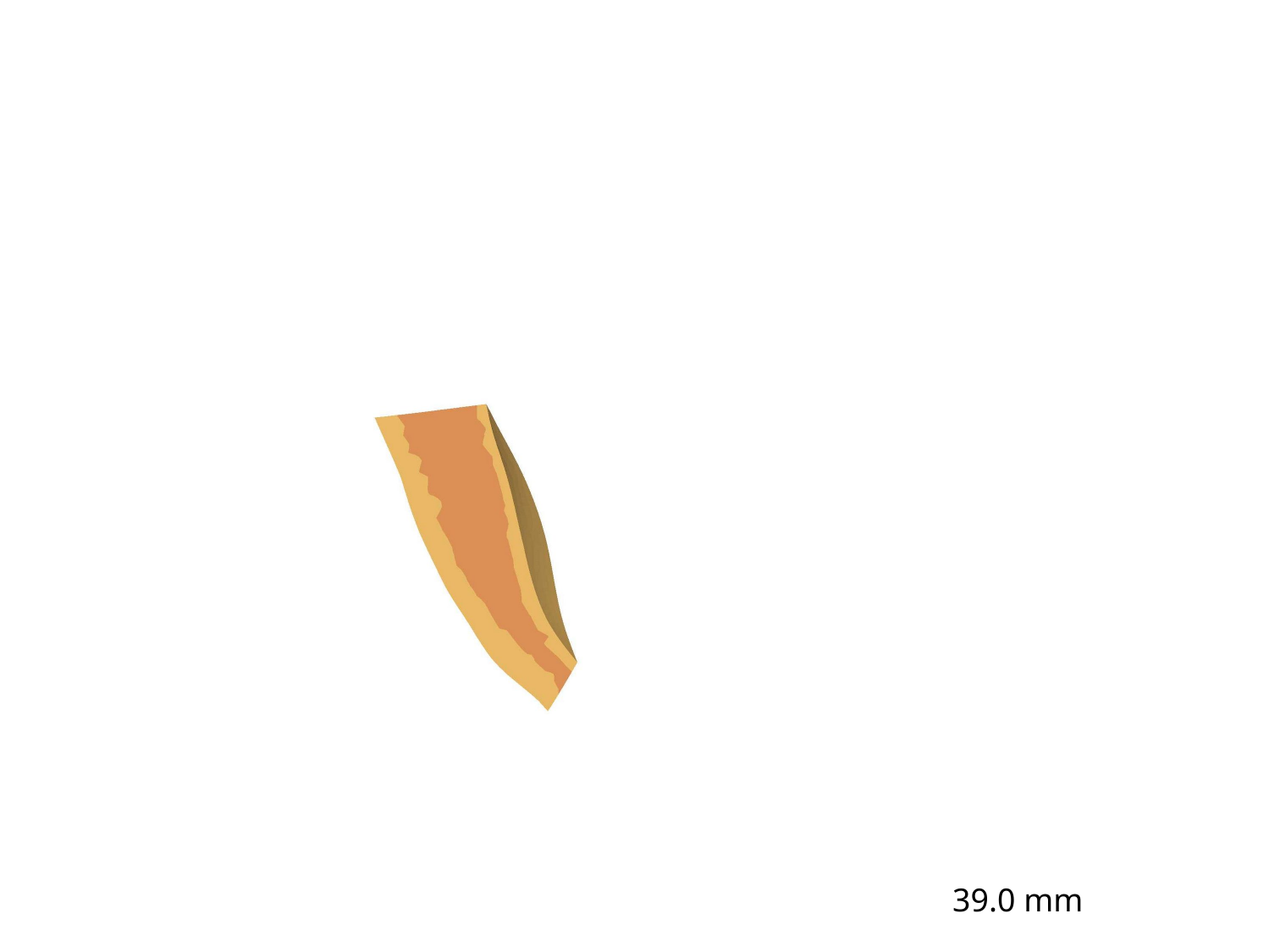

39.0 mm

## Slide 157
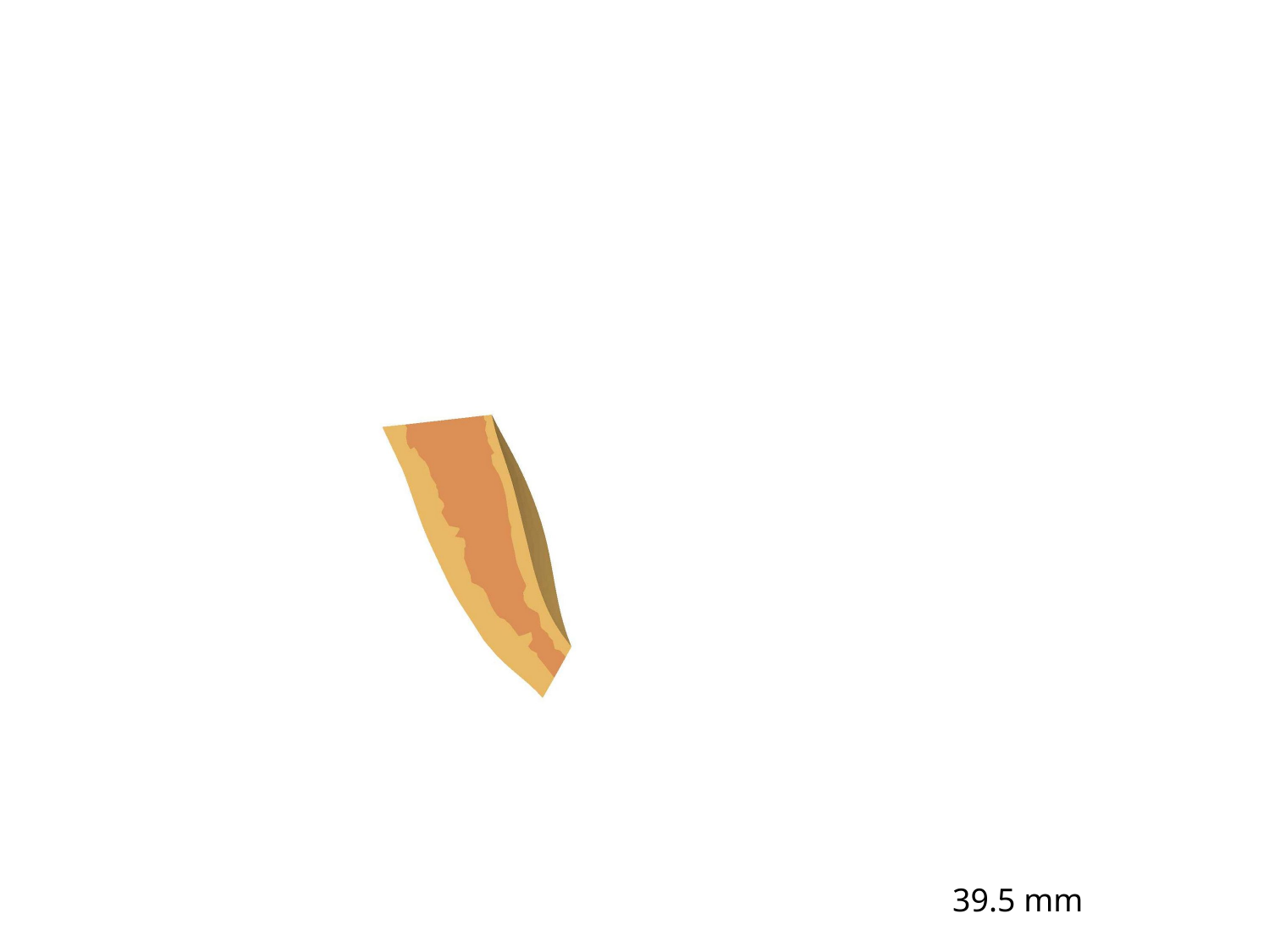

39.5 mm

## Slide 158
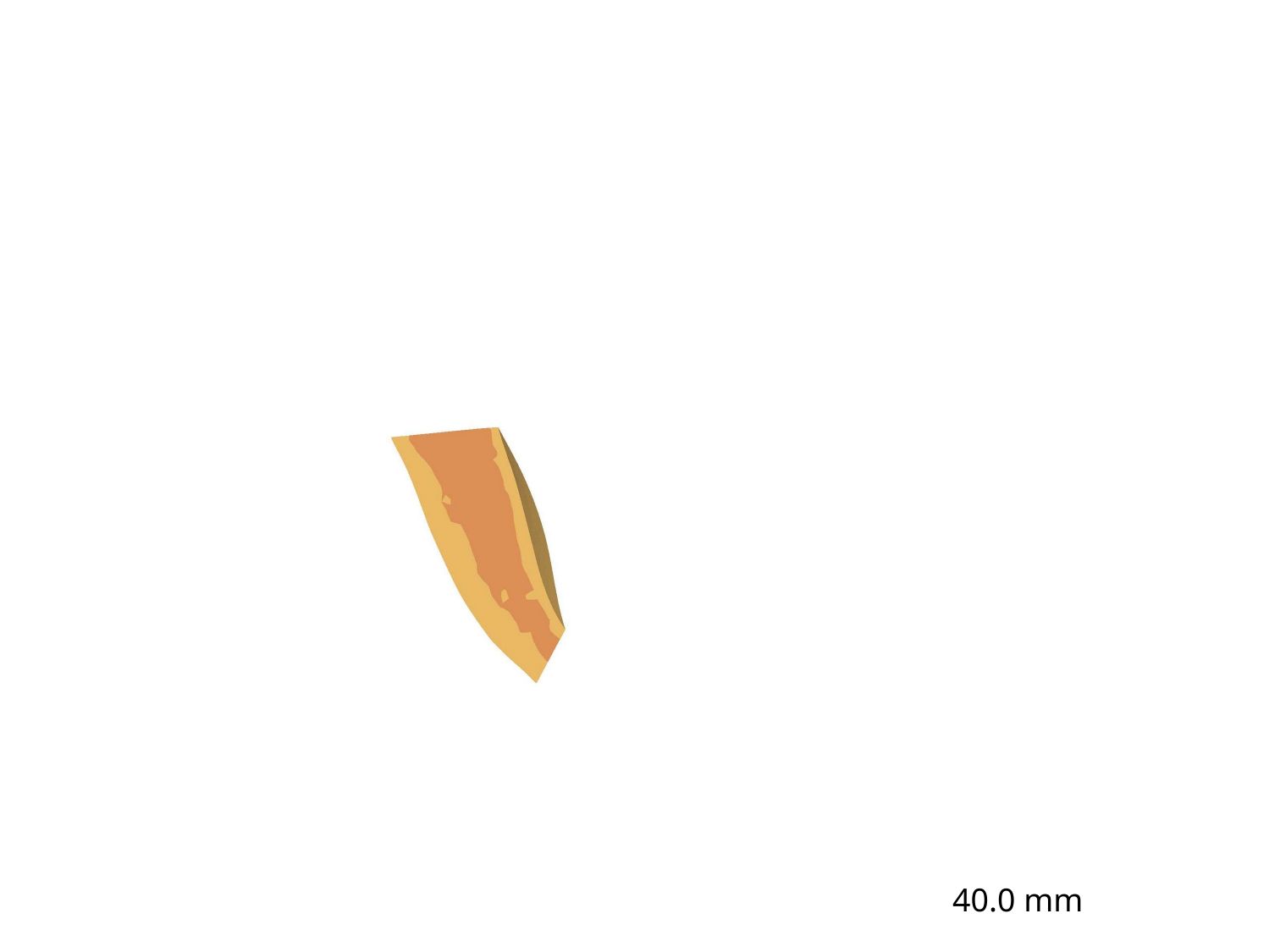

40.0 mm

## Slide 159
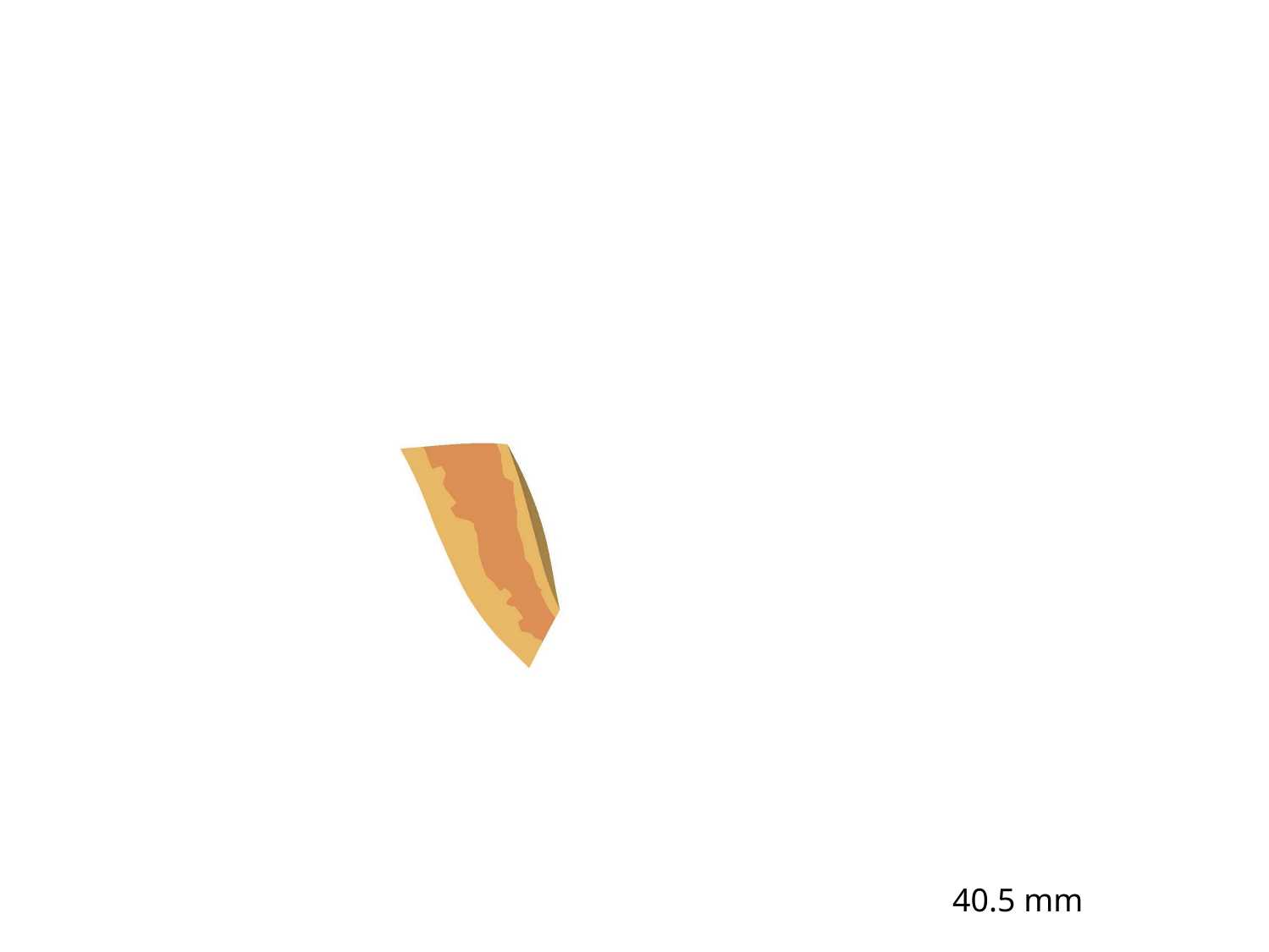

40.5 mm

## Slide 160
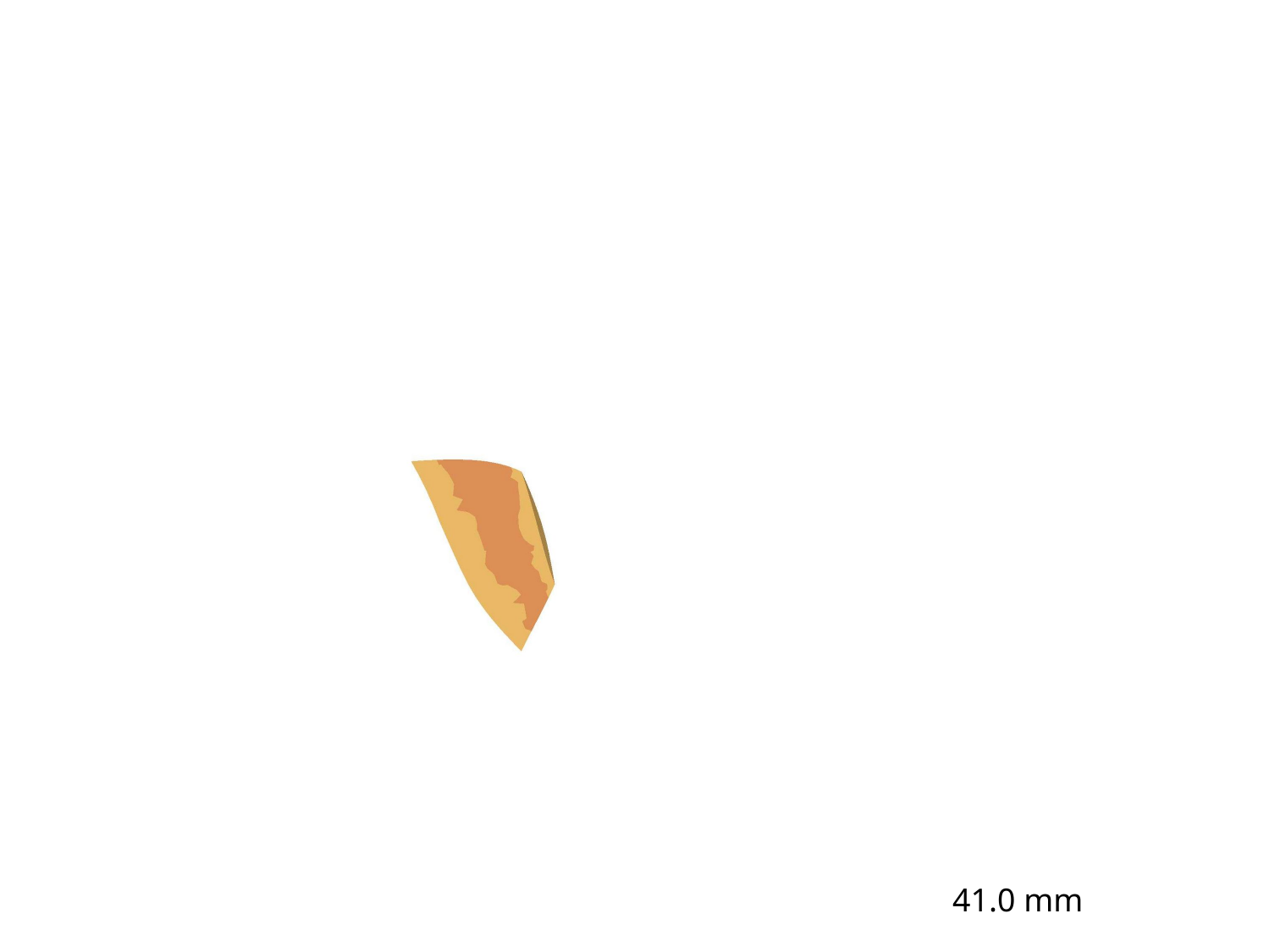

41.0 mm

## Slide 161
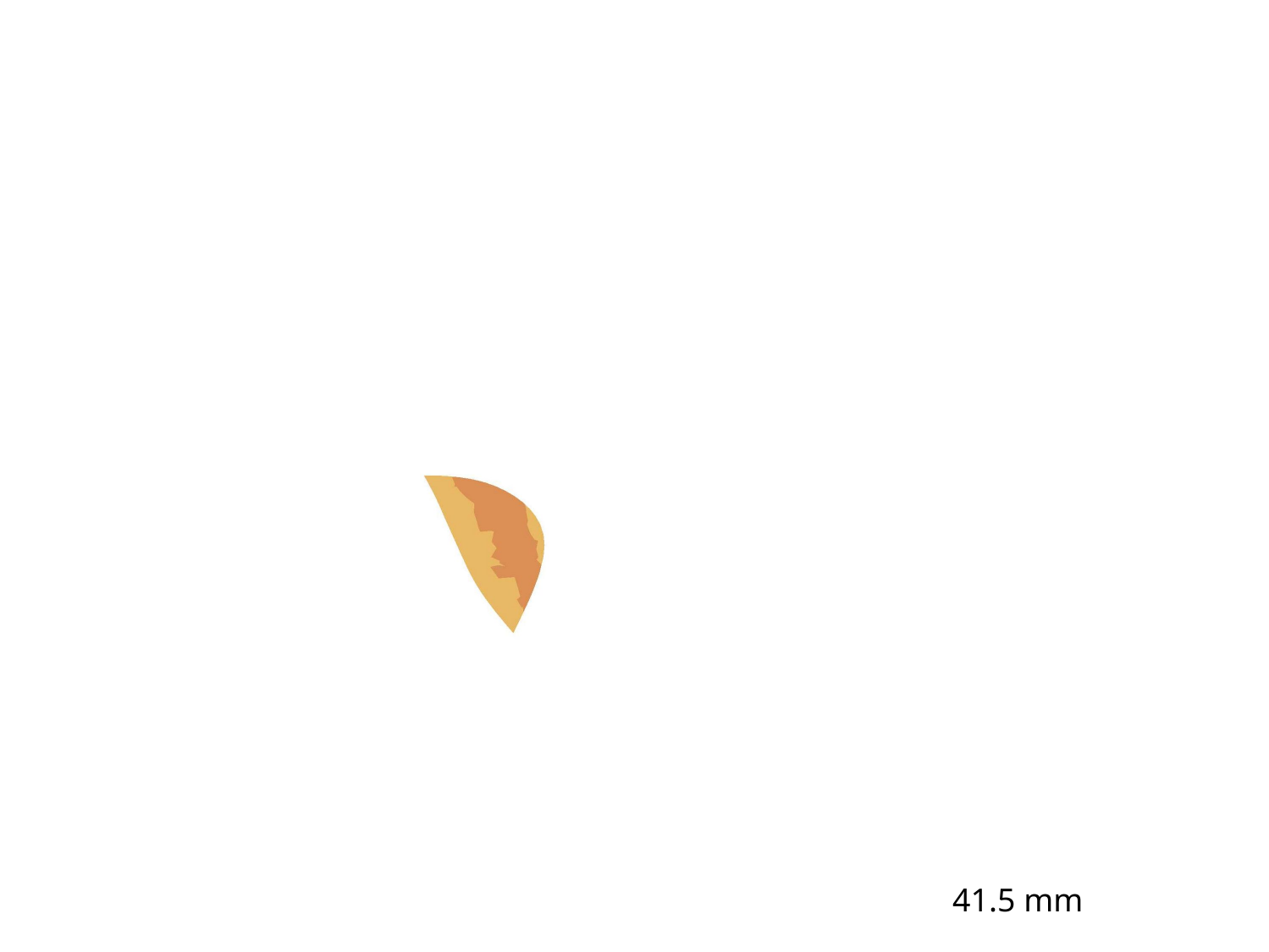

41.5 mm

## Slide 162
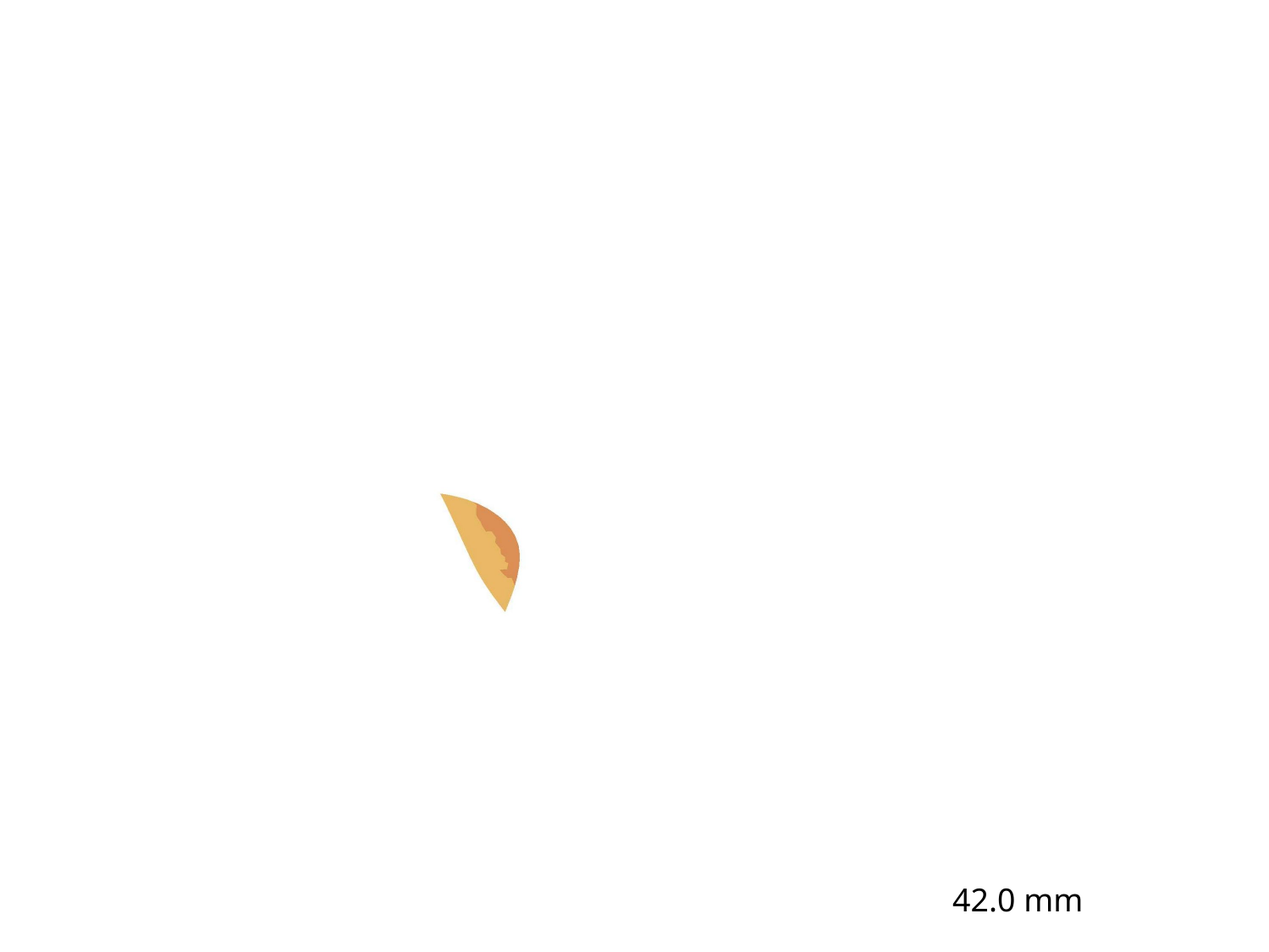

42.0 mm

## Slide 163
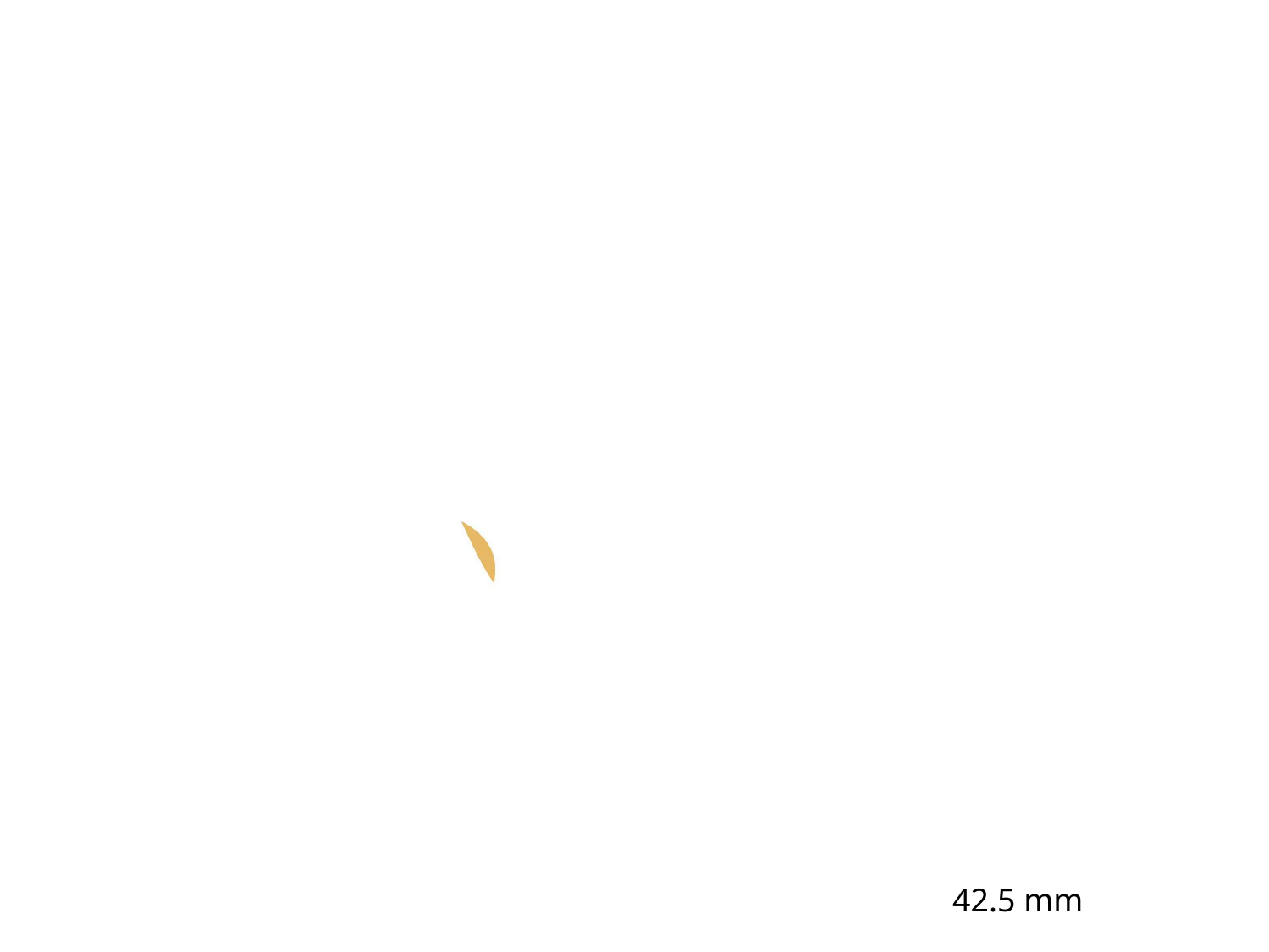

42.5 mm
