# Supplementary material for: Directional sensitivity of bone conduction stimulation on the otic capsule in a finite element model of the human temporal bone
Source: Sci Rep. 2024 Jun 14;14:13768. doi: 10.1038/s41598-024-64377-x (PMC11178818; doi:10.1038/s41598-024-64377-x)
Supplement: Supplementary file 2 — Supplementary Information 2. [file 41598_2024_64377_MOESM2_ESM.pptx]

## Slide 1
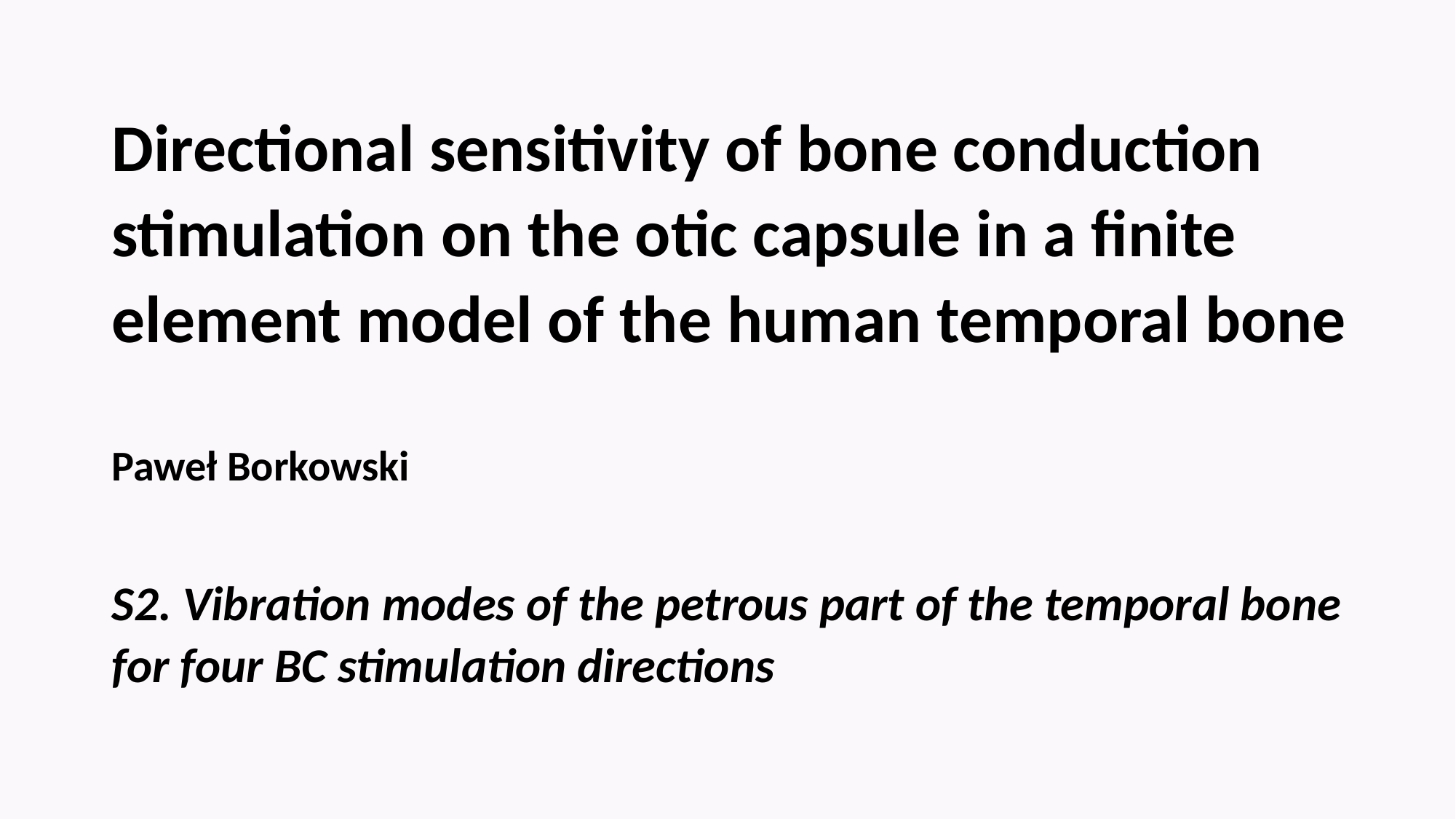

Directional sensitivity of bone conduction stimulation on the otic capsule in a finite element model of the human temporal bone
Paweł Borkowski
S2. Vibration modes of the petrous part of the temporal bone for four BC stimulation directions

## Slide 2
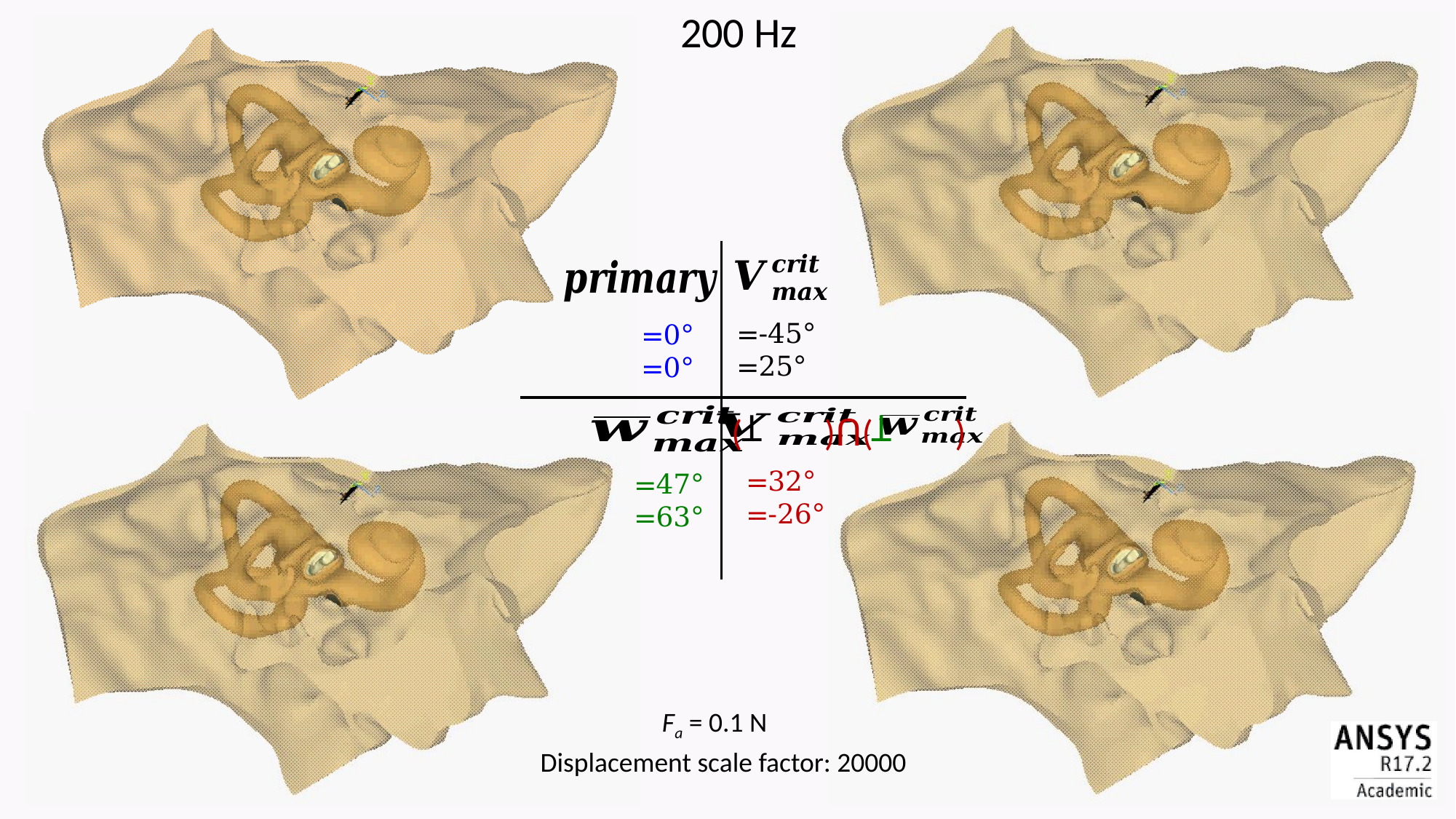

200 Hz
( )ꓵ( )
Ʇ
Ʇ
Fa = 0.1 N
Displacement scale factor: 20000

## Slide 3
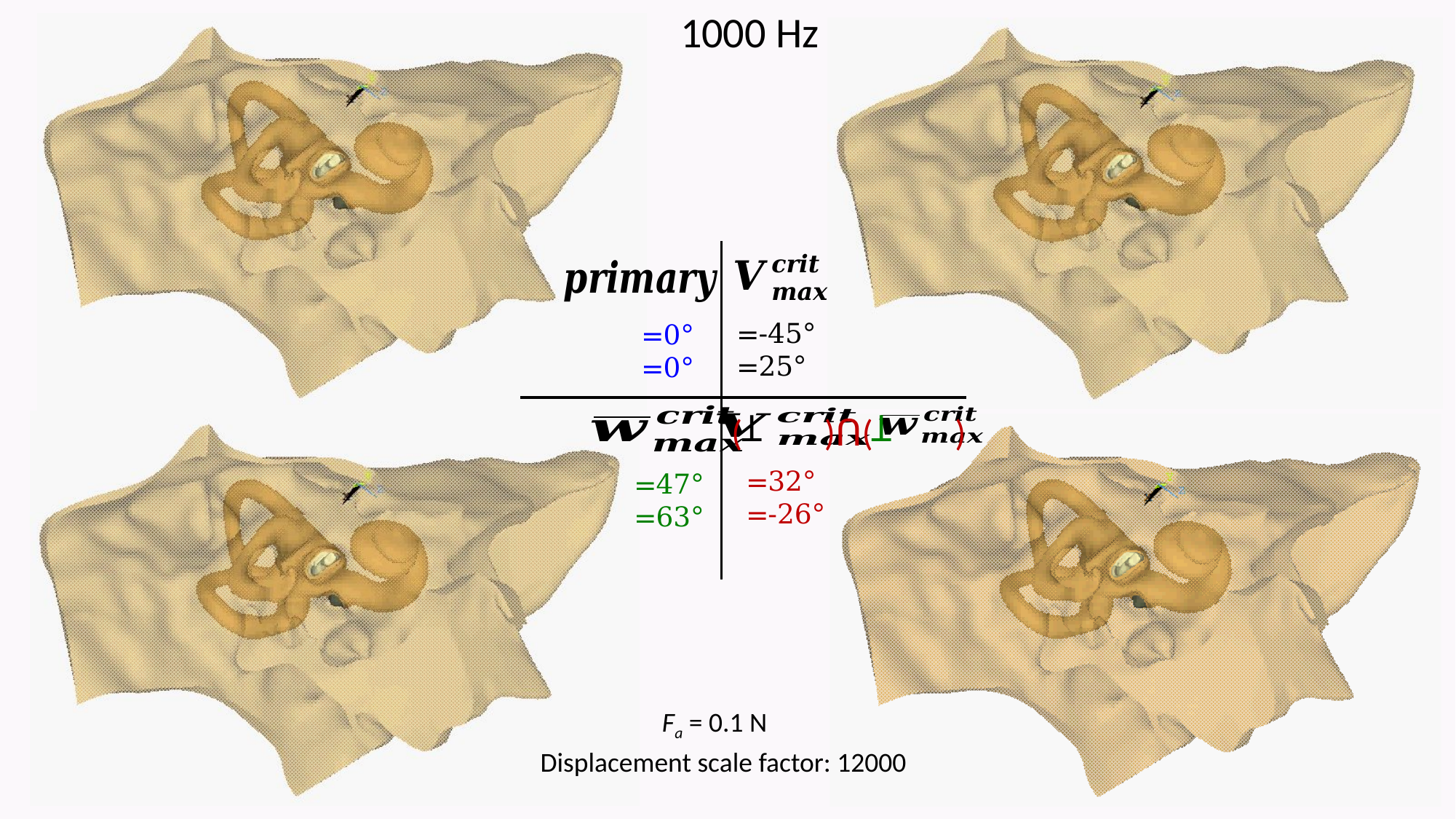

1000 Hz
( )ꓵ( )
Ʇ
Ʇ
Fa = 0.1 N
Displacement scale factor: 12000

## Slide 4
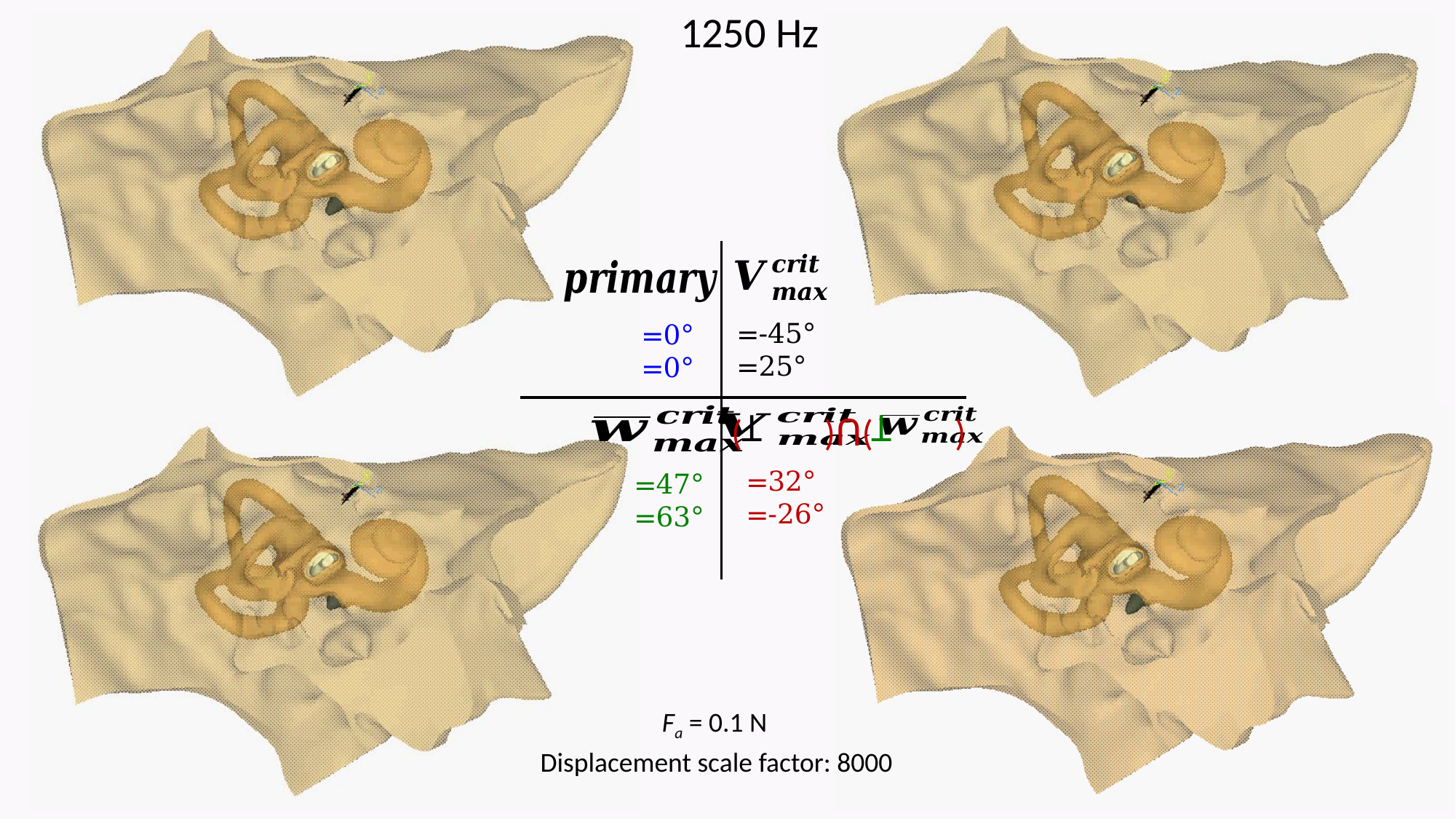

1250 Hz
( )ꓵ( )
Ʇ
Ʇ
Fa = 0.1 N
Displacement scale factor: 8000

## Slide 5
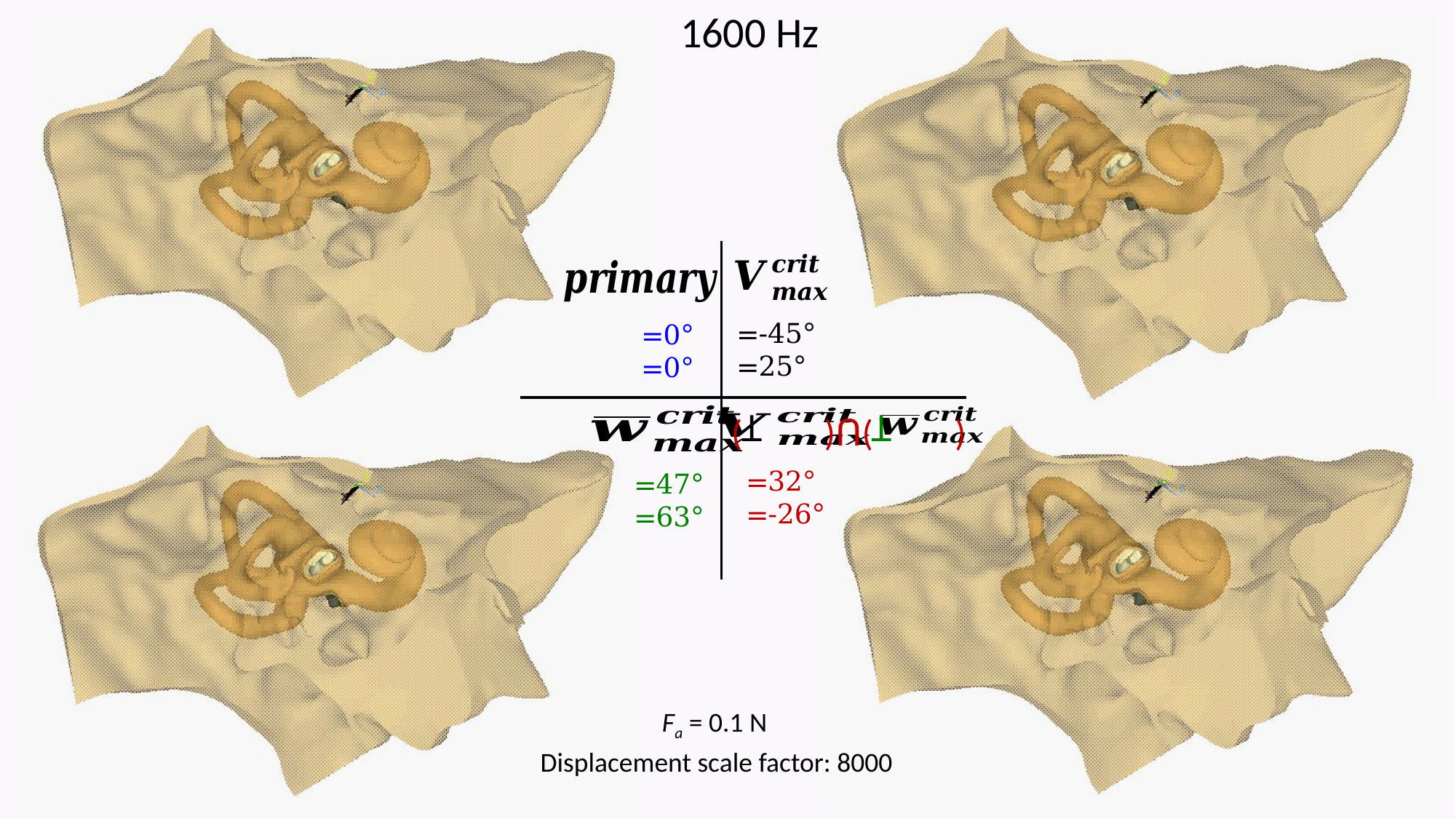

1600 Hz
( )ꓵ( )
Ʇ
Ʇ
Fa = 0.1 N
Displacement scale factor: 8000

## Slide 6
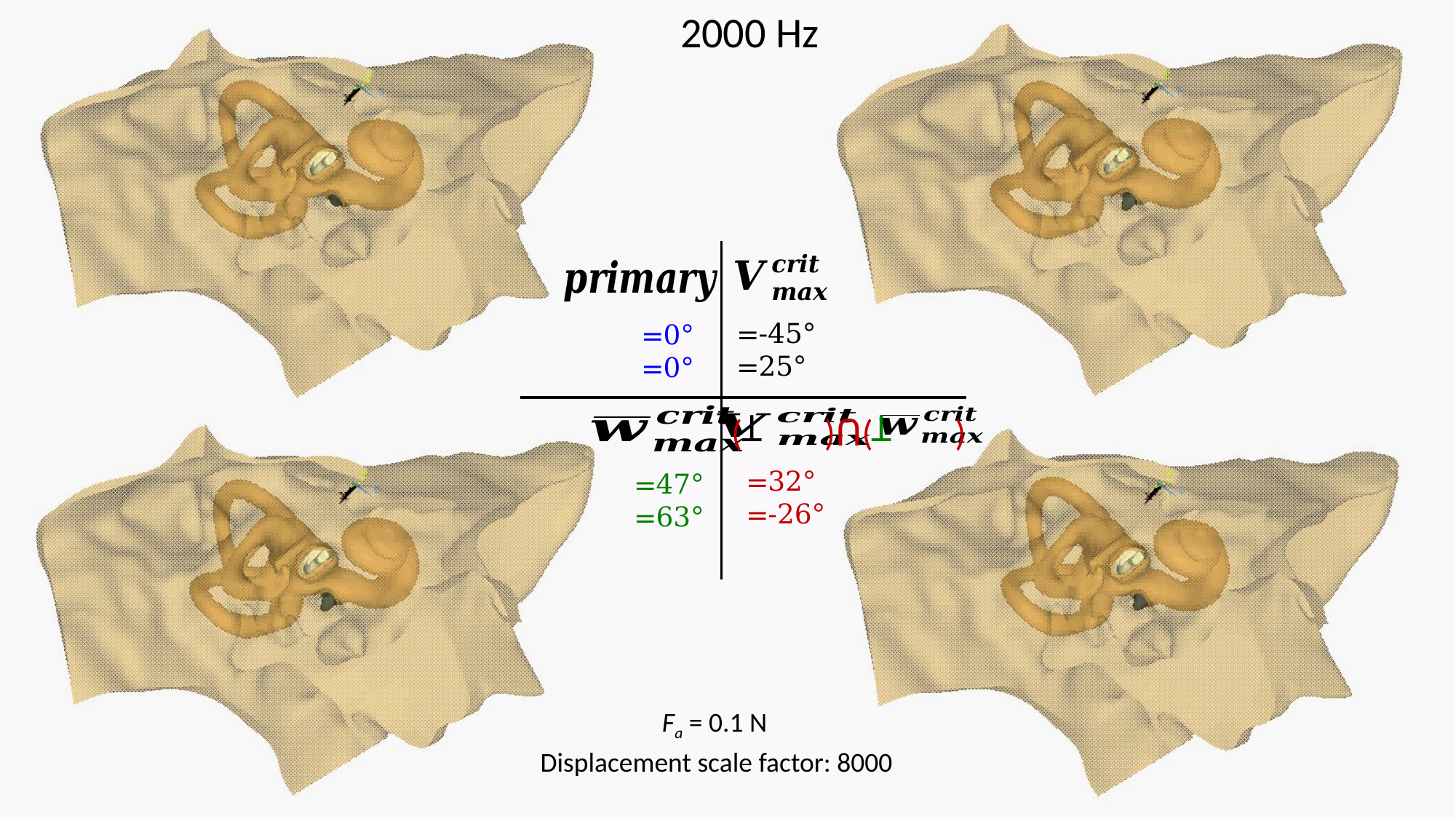

2000 Hz
( )ꓵ( )
Ʇ
Ʇ
Fa = 0.1 N
Displacement scale factor: 8000

## Slide 7
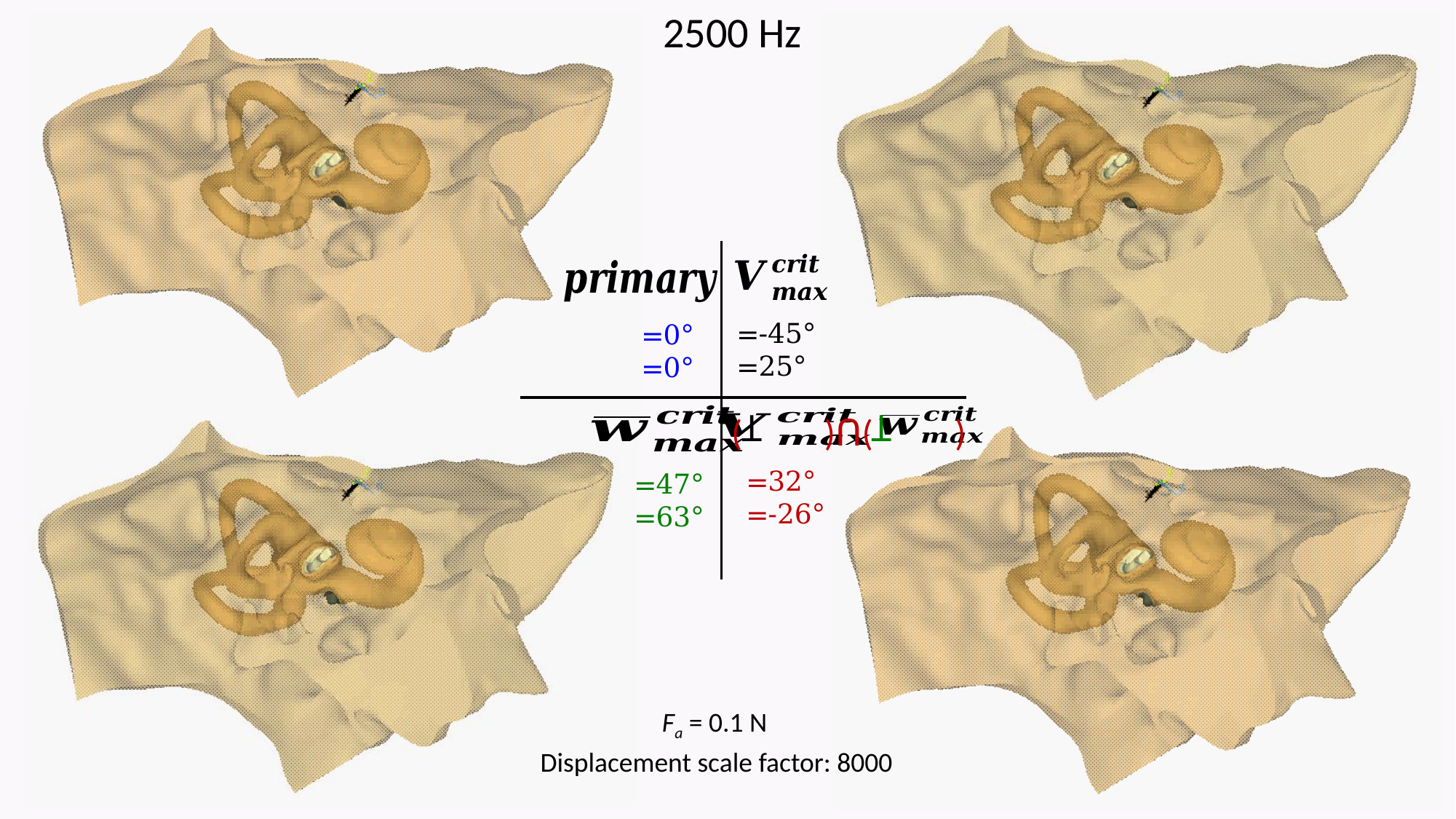

2500 Hz
( )ꓵ( )
Ʇ
Ʇ
Fa = 0.1 N
Displacement scale factor: 8000

## Slide 8
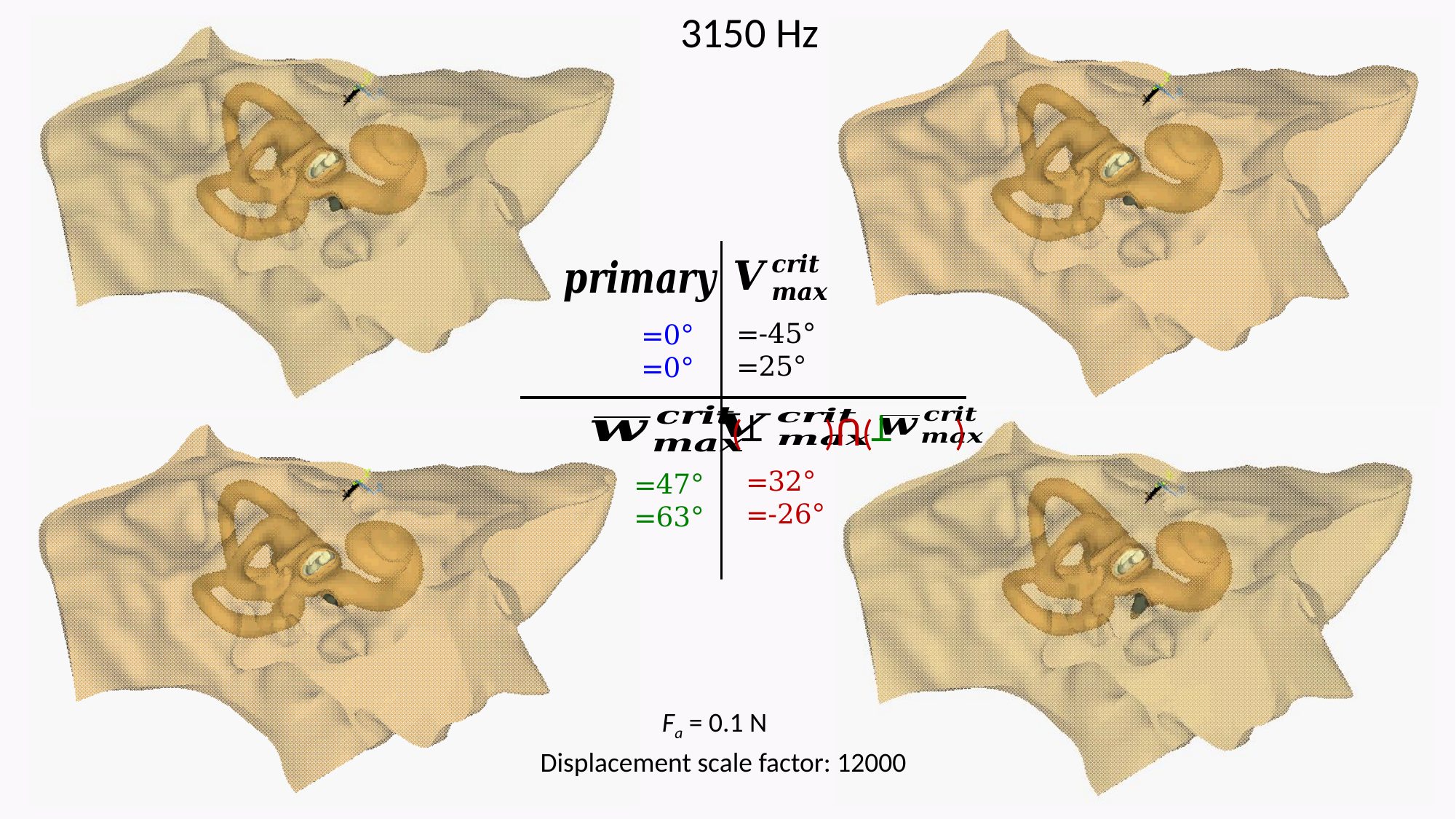

3150 Hz
( )ꓵ( )
Ʇ
Ʇ
Fa = 0.1 N
Displacement scale factor: 12000

## Slide 9
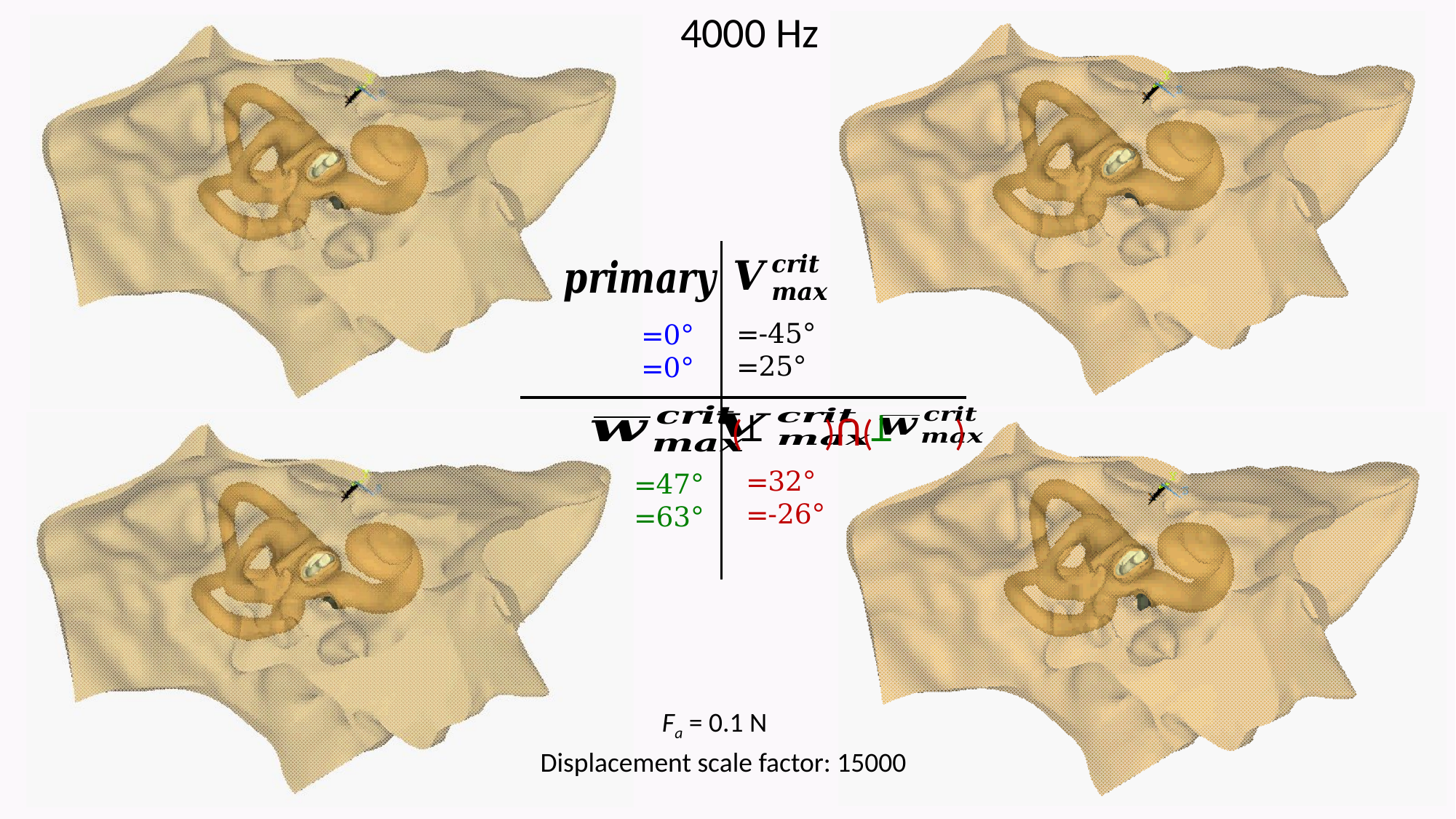

4000 Hz
( )ꓵ( )
Ʇ
Ʇ
Fa = 0.1 N
Displacement scale factor: 15000

## Slide 10
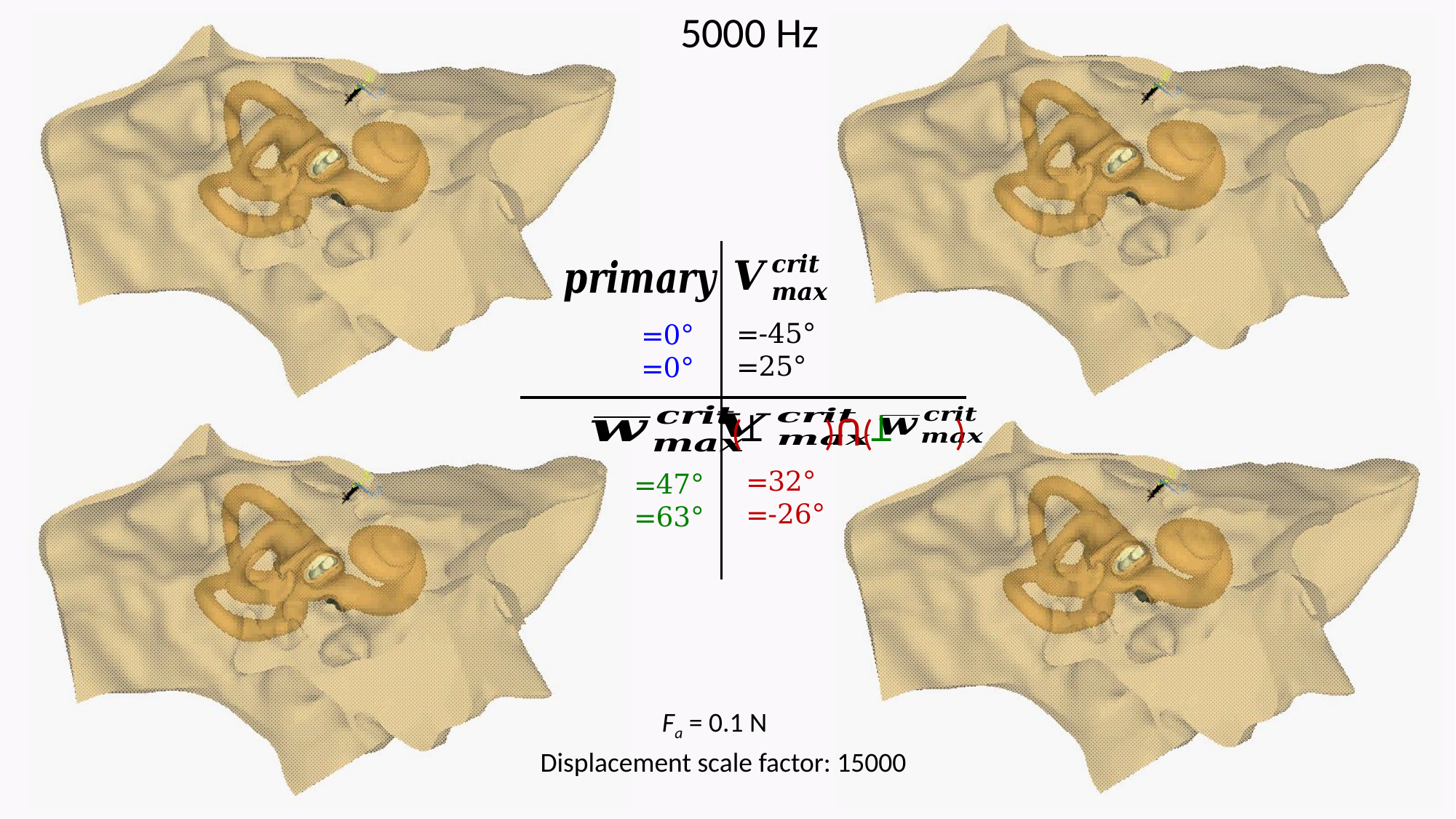

5000 Hz
( )ꓵ( )
Ʇ
Ʇ
Fa = 0.1 N
Displacement scale factor: 15000

## Slide 11
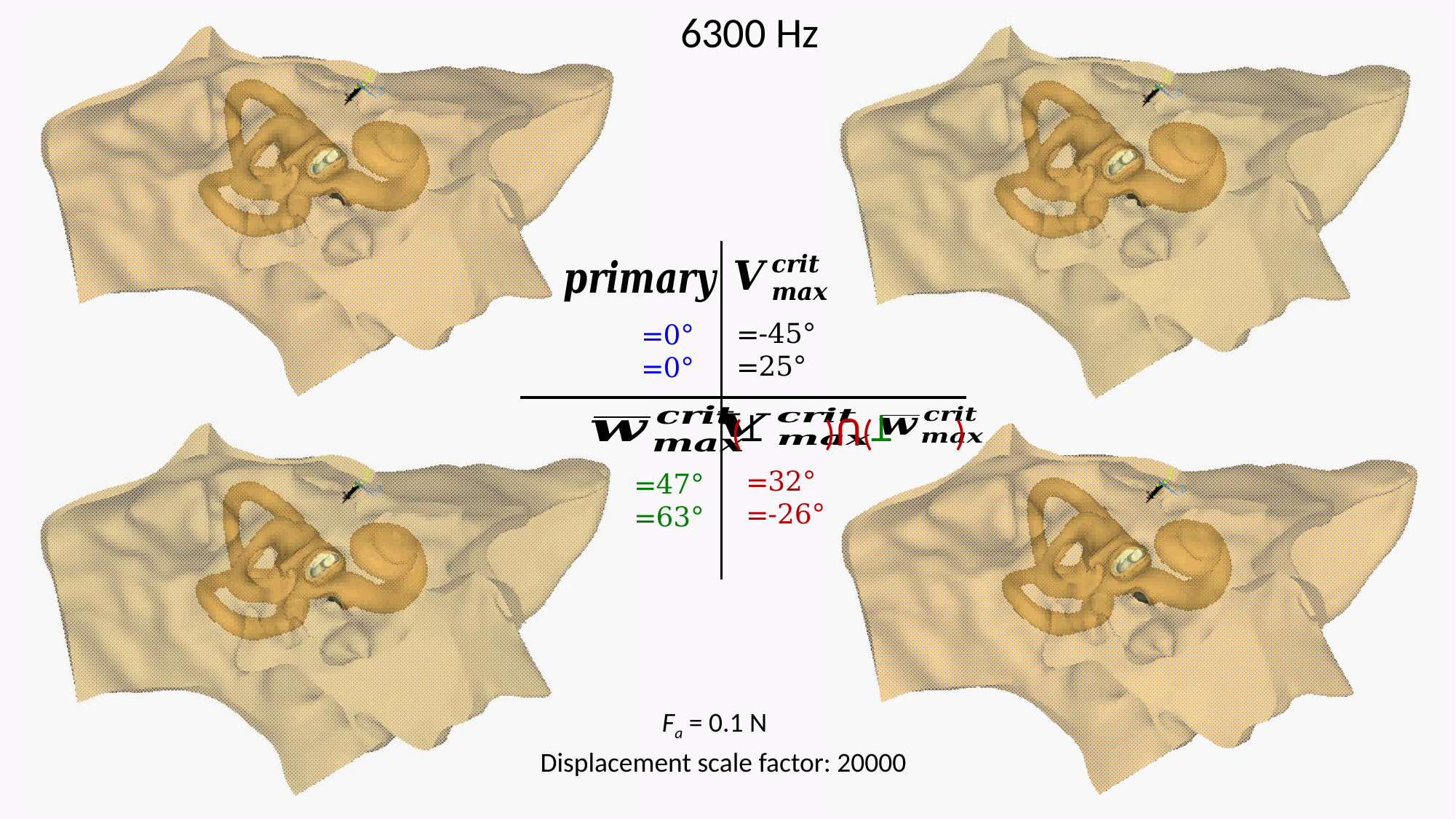

6300 Hz
( )ꓵ( )
Ʇ
Ʇ
Fa = 0.1 N
Displacement scale factor: 20000

## Slide 12
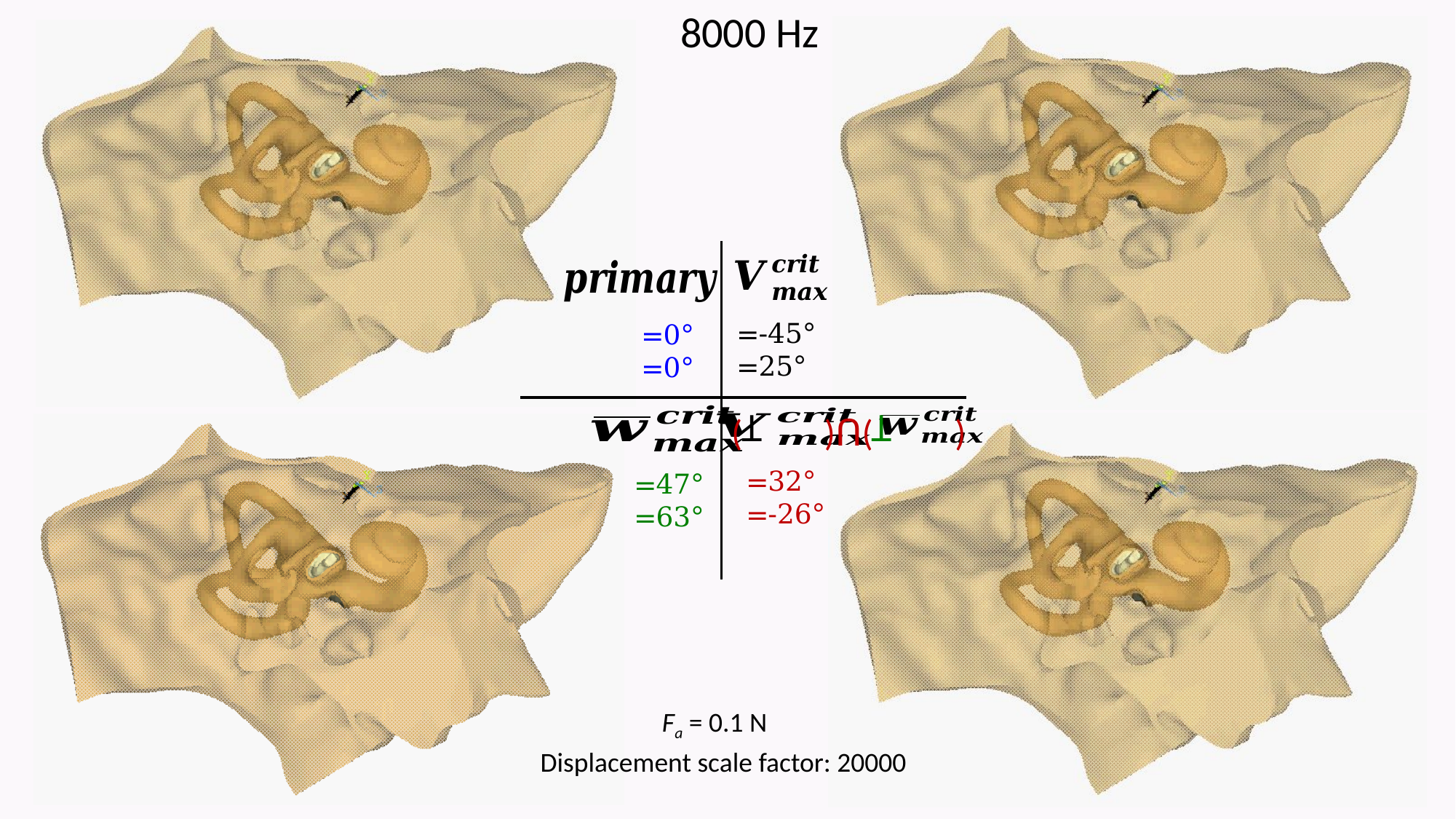

8000 Hz
( )ꓵ( )
Ʇ
Ʇ
Fa = 0.1 N
Displacement scale factor: 20000

## Slide 13
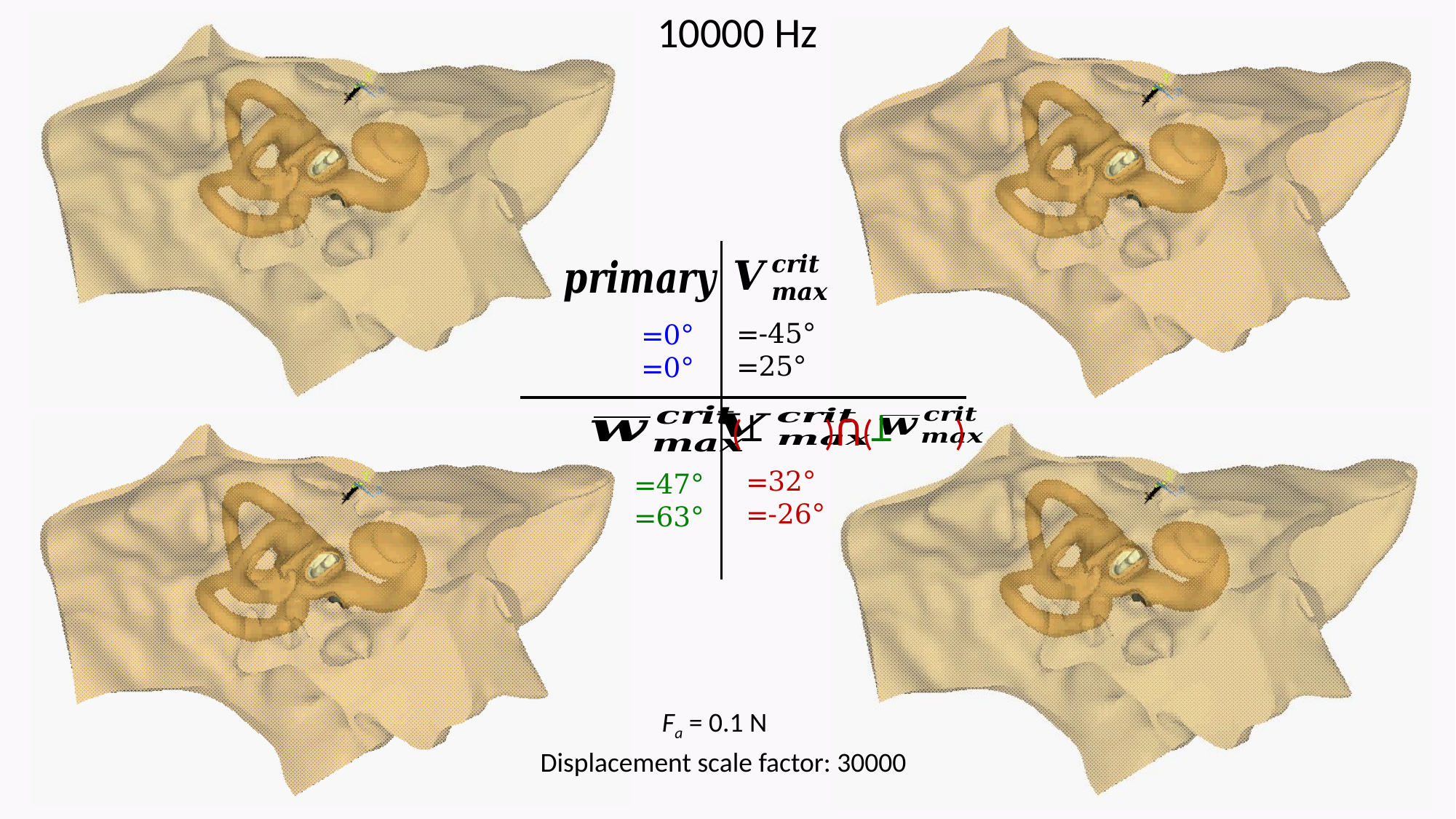

10000 Hz
( )ꓵ( )
Ʇ
Ʇ
Fa = 0.1 N
Displacement scale factor: 30000
